# Multicomponent Molecular Editing of Polybutadiene: From Design Space to Battery Function

**DOI:** 10.1002/advs.78468

**Published:** 2026-11-03

**Authors:** Janna Jeschke, Oghenegare Emmanuel Eyankware, Ioannis Moutsios, Loïc Vidal, Cyril Vaulot, Azra Kocaarslan, Angelina Sarapulova, Sonia Dsoke, Hatice Mutlu

**Affiliations:** ^1^ Institut de Science des Matériaux de Mulhouse (IS2M) UMR 7361 CNRS/Université de Haute Alsace (UHA) Mulhouse France; ^2^ Fraunhofer Institute for Solar Energy Systems Freiburg Germany; ^3^ FMF‐Freiburg Materials Research Center University of Freiburg Freiburg Germany; ^4^ Institute for Sustainable Systems Engineering (INATECH) University of Freiburg Freiburg Germany; ^5^ Department of Materials Science Engineering University of Ioannina University Campus‐Dourouti Ioannina Greece; ^6^ Institute of Functional Interfaces (IFG) Karlsruhe Institute of Technology (KIT) Eggenstein‐Leopoldshafen Germany; ^7^ Rheinland‐Pfälzische Technische Universität Kaiserslautern‐Landau (RPTU) Department of Chemistry Technical Polymer Chemistry Kaiserslautern Germany; ^8^ Leibniz‐Institut für Verbundwerkstoffe GmbH Kaiserslautern Germany; ^9^ Helmholtz Institute for Polymers in Energy Applications Jena (HIPOLE Jena) Helmholtz‐Zentrum Berlin für Materialien und Energie (HZB) Jena Germany; ^10^ Helmholtz‐Zentrum Berlin for Materials and Energy GmbH Berlin Germany; ^11^ Friedrich Schiller University Jena Institute of Organic Chemistry and Macromolecular Chemistry Jena Germany

**Keywords:** functional materials discovery, Li–S battery electrolyte additives, molecular design space, polybutadiene, polymer libraries, post‐polymerization modification, structure–property–function relationships

## Abstract

Multicomponent post‐polymerization modification (PPM) enables polymer diversification, yet its potential for exploring structure‐property‐function relationships remains underexplored. Herein, we report an electrophilic multicomponent molecular editing strategy that enables the construction of a polybutadiene‐derived molecular design space through systematic variation of cyclic ethers and *N*‐ or *O‐*nucleophiles. Combining three cyclic ethers with six nucleophiles generated an 18‐member library, varying spacer architecture, polarity, and intermolecular interactions (**P1** ‐ **P18**). Mapping thermal, selected morphological, and molecular mobility properties revealed substantial changes arising from subtle molecular variations. In particular, **P9** was distinguished by its nanoscale organization and proton relaxation behavior, providing insight into the influence of molecular structure on morphology and molecular dynamics. Mapping these complementary property landscapes guided the selection of **P11** for preliminary evaluation as an electrolyte additive for Li–S batteries, based on its balance of functionality and physicochemical properties. Electrochemical testing in a deliberately LiNO_3_‐free model electrolyte revealed that **P11** altered sulfur redox behavior and impedance response, increased Coulombic efficiency, and showed more gradual capacity decay relative to the additive‐free baseline, albeit with increased polarization and reduced initial capacity. These trade‐offs demonstrate library‐guided selection without establishing **P11** as an optimized additive. Descriptive sensitivity analysis identified nucleophile identity as the dominant input for functionalization degree (FD) and thermal stability, whereas glass‐transition temperature showed sensitivity to both molecular inputs.

## Graphical abstract

Multicomponent molecular editing transforms polybutadiene into an 18‐member polymer library with tunable thermal properties, nanoscale organization, and molecular dynamics. Mapping this multidimensional design space reveals distinct molecular input–property relationships and guides the selection of a candidate electrolyte additive for lithium–sulfur batteries, demonstrating how systematic polymer diversification can bridge molecular design and application‐oriented materials discovery.

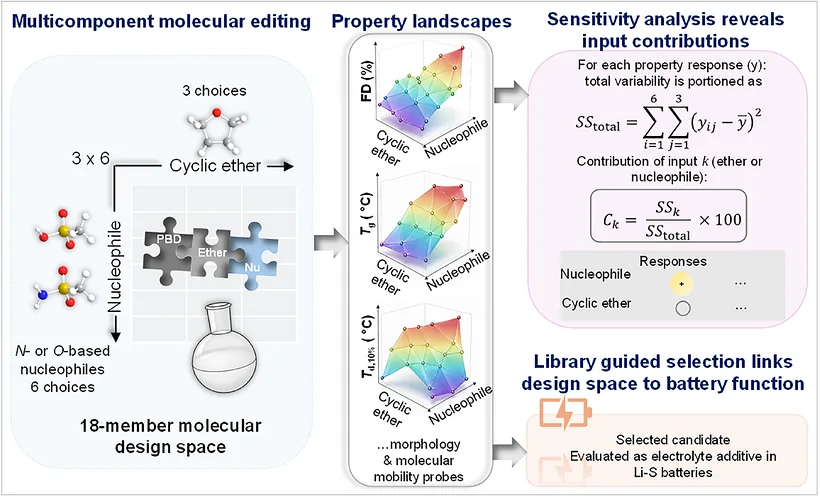

## 1 Introduction

Polybutadiene (**PBD**) is a widely produced synthetic polymer and constitutes a major component of commercial elastomers such as styrene‐butadiene rubber (SBR), which is extensively used in tire manufacturing [1, 2]. Given its large production volume and the growing demand for sustainable materials management, strategies to transform polybutadiene‐based materials into value‐added products have become increasingly important [3, 4]. In this context, post‐polymerization modification (PPM) offers an attractive approach as it enables the introduction of new functionalities into existing polymer backbones without requiring the synthesis of entirely new monomers or polymers [5]. Multicomponent post‐polymerization modification reactions have recently emerged as versatile tools for the rapid diversification of polymer structures. In contrast to conventional sequential functionalization approaches, multicomponent reactions enable the simultaneous incorporation of several structural elements in a single transformation, thereby reducing synthetic complexity [6]. Such approaches may also reduce *E*‐factors, improve atom economy, and minimize waste generation, particularly when reaction components such as solvents are directly incorporated into the final material [7].

For polybutadiene and its derivatives, numerous PPM strategies have been reported to introduce polar groups [8], reactive handles [9], supramolecular motifs [10], and electrochemically active functionalities [11]. While these studies have significantly expanded the range of accessible polybutadiene‐derived materials, they have predominantly focused on individual materials. Consequently, structure‐property relationships are often established on a case‐by‐case basis, limiting the identification of general molecular design principles. Although examples of multicomponent functionalization of polybutadiene have recently emerged [9, 12, 13, 14], the systematic exploration of multicomponent molecular design spaces and their influence on material properties remains largely underexplored.

Advances in materials discovery increasingly rely on establishing structure‐property‐function relationships across chemically diverse material libraries [15, 16]. However, the construction of sufficiently diverse yet structurally related libraries in polymer chemistry remains synthetically demanding. We hypothesized that electrophilic multicomponent molecular editing of polybutadiene could provide a modular approach to this challenge by enabling the independent variation of two orthogonal molecular editing elements: the nucleophile and the cyclic ether. Whereas the nucleophile determines the polarity, reactivity, and intermolecular interaction profile of the introduced functionality, the cyclic ether defines the spacer architecture connecting the functionality to the polymer backbone. Together, these variables generate a chemically diverse yet structurally related molecular landscape from a common polybutadiene precursor [17].

Herein, we report an electrophilic multicomponent molecular editing strategy that enables the rapid construction of a systematically tunable polybutadiene library. By combining six different N‐ and O‐nucleophiles with three cyclic ethers, an 18‐member library was constructed (**P1**‐**P18**) in which both the chemical functionality and the spacer architecture were systematically varied (Figure 1). The resulting design space was subsequently mapped across multiple property dimensions, including thermal behavior (Differential Scanning Calorimetry (DSC), Thermogravimetric Analysis (TGA)), nanoscale morphology (Atomic Force Microscopy (AFM), Transmission Electron Microscopy (TEM)), and molecular dynamics (low‐field proton nuclear magnetic resonance (^1^H NMR) relaxometry). Comparison across these complementary property landscapes revealed that relatively subtle changes in the molecular editing components can translate into pronounced differences in higher‐order material behavior. In particular, **P9** exhibited distinct nanoscale contrast and proton relaxation behavior compared with closely related library members, indicating how molecular editing can influence both nanoscale organization and molecular dynamics. To further explore whether this library‐based materials diversification could translate into application‐relevant function, **P11** was selected as a candidate electrolyte additive for Li–S batteries based on its balanced combination of chemical functionality and physicochemical properties. This selection was further motivated by the presence of sulfonate ester groups in **P11**, which provide polar sulfur‐ and oxygen‐containing motifs with potential relevance to electrolyte/electrode interfacial processes. Its subsequent electrochemical evaluation demonstrated how multicomponent molecular editing can connect systematic design‐space exploration with the identification of functional polymer candidates. Finally, descriptive sensitivity analysis was performed to distinguish the relative contributions of cyclic ether and nucleophile identity to the observed variation in functionalization degree (FD) and material properties across the multidimensional library [18, 19].

**FIGURE 1:**
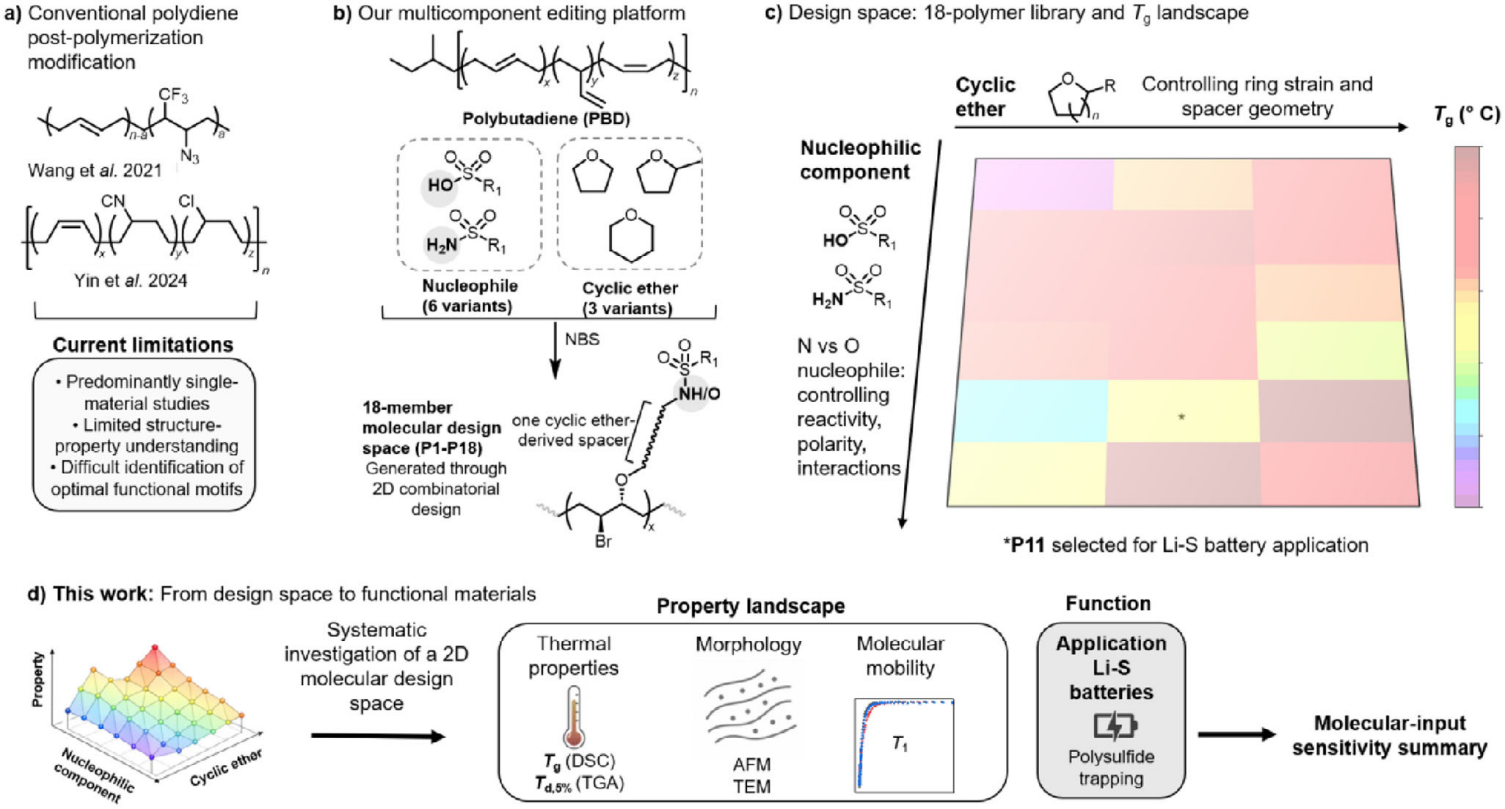
Construction of a multicomponent molecular editing design space for polybutadiene. (a) Current limitations of post‐polymerization modification studies [9, 14], which predominantly focus on individual materials and therefore provide only limited insight into broader structure‐property relationships. (b) Electrophilic multicomponent molecular editing of polybutadiene through the simultaneous incorporation of cyclic ethers and *N*‐ or *O*‐nucleophiles. The cyclic ether controls the spacer architecture between the polymer backbone and the introduced functionality, while the nucleophile modulates the polarity and intermolecular interaction profile of the resulting material. (c) Construction of an 18‐member molecular design space by systematically varying both molecular editing components. (d) Mapping of the resulting library across multiple property dimensions, including thermal behavior (DSC, TGA), nanoscale morphology (AFM, TEM), and molecular mobility as reflected in proton relaxation behavior (low‐field ^1^H NMR relaxometry), generates structure‐property landscapes that enable the identification of promising functional materials. Electrochemical evaluation of a selected library member as an electrolyte additive for Li─S batteries provides a proof‐of‐concept for connecting systematic design‐space exploration with functional materials discovery.

This approach transforms a single commodity polymer into a chemically addressable materials design space, connecting molecular inputs to distinct property responses and, ultimately, electrochemical function.

## 2 Results and Discussion

### 2.1 Construction and Structural Verification of the Molecular Library

To construct the molecular library, our previously reported electrophilic multicomponent molecular editing protocol [13] was employed (see Section SA.2 for experimental details). The pristine **PBD** precursor was microstructurally heterogeneous, comprising 16 mol% 1,2‐vinyl, 34 mol% *cis*‐1,4, and 50 mol% *trans*‐1,4 repeating units, as determined by quantitative ^1^H and quantitative ^13^C NMR spectroscopy (Section SC.10). Consequently, functionalization of these different unsaturated repeating‐unit environments can generate stereogenic centers; no stereochemical assignment of the resulting polymer‐bound functionalities is implied by the schematic structures shown herein. Three cyclic ethers (tetrahydrofuran (THF), 2‐methyltetrahydrofuran (2‐MeTHF), and tetrahydropyran (THP)) were combined with six *O*‐ and *N*‐nucleophiles, including four sulfonamides (methanesulfonamide (MeSO_2_NH_2_), *p*‐toluenesulfonamide (*p*‐TsNH_2_), 4‐nitrobenzenesulfonamide (*p*‐NsNH_2_), and 4‐(trifluoromethyl)benzenesulfonamide (*p*‐CF_3_BsNH_2_)) and two sulfonic acids (methanesulfonic acid (MSA) and *p*‐toluenesulfonic acid (*p*‐TsOH)), affording an 18‐member polymer library (**P1**–**P18**, Figure 2a). This modular design enables the independent variation of two orthogonal molecular design variables: (i) the spacer architecture, defined by the cyclic ether, and (ii) the polarity and intermolecular interaction profile, governed by the nucleophile.

**FIGURE 2:**
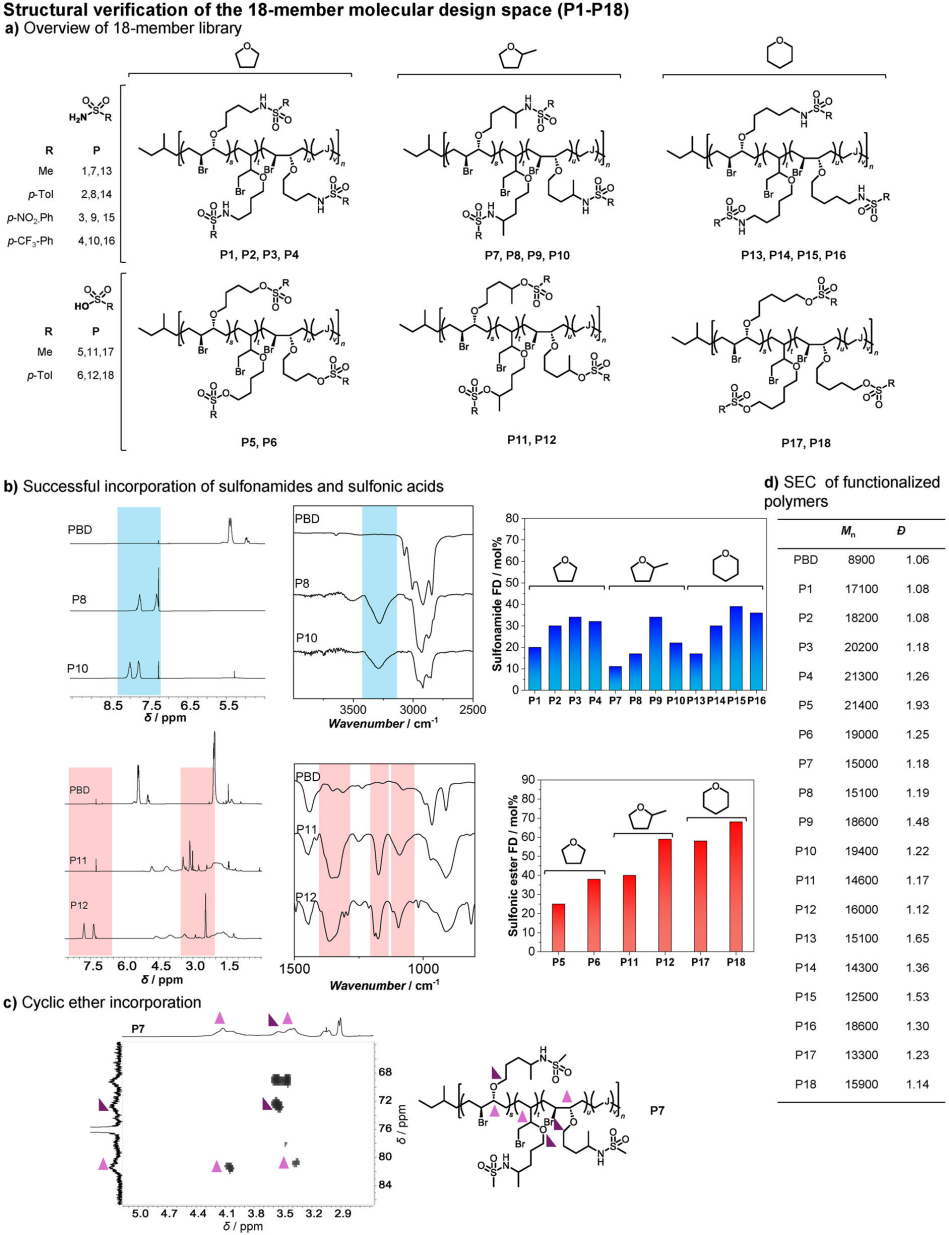
Construction and structural verification of the 18‐member molecular design space (**P1‐P18**). (a) Overview of the library generated by combining three cyclic ethers (**THF**, **2‐MeTHF**, and **THP**) with four sulfonamides (**MeSO_2_NH_2_**, ***p*‐TsNH_2_**, ***p*‐NsNH_2_**, and ***p*‐CF_3_BsNH_2_**) and two sulfonic acids (**MSA** and ***p‐*TsOH**). (b) Successful incorporation of sulfonamides and sulfonate esters is supported by representative ^1^H NMR and FTIR spectra, together with the average functionalization degree (FD), estimated by internal‐standard quantitative ^1^H NMR spectroscopy (the calculation is detailed in Section SA.4). (c) Verification of cyclic ether incorporation by HSQC NMR spectroscopy (exemplified for **P7**). (d) SEC analysis in THF summarizing the apparent number‐average molar masses (*M*_n_) and dispersity (*Đ*) values of the edited polymers relative to pristine polybutadiene. Polymer structures are shown schematically and do not imply a defined sequence or relative positioning of the introduced functionalities along individual polymer chains.

The successful incorporation of sulfonamide functionalities was confirmed by ^1^H NMR and FTIR spectroscopy. Representative ^1^H NMR spectra of **P8** and **P10** (Figure 2b) show the expected aromatic resonances arising from the introduced sulfonamide substituents, while FTIR spectra display the characteristic N–H stretching vibration at approximately 3300 cm^−1^ together with the expected S═O and C─O stretching vibrations (Figure 2b and Figures S72–S89). Structural assignments for all library members were further supported by ^13^C NMR spectroscopy (cf. Figures S19–S71 for all NMR spectra). Internal‐standard quantitative ^1^H NMR (qNMR) spectroscopy was used to estimate the functionalization degree (FD) using the calculation procedure detailed in Section SA.4. *Note to the reader*: The reported FD represents the ensemble‐average molar fraction of repeating units bearing the incorporated sulfonamide or sulfonate ester functionality; it does not resolve derivatization separately at 1,2‐ and 1,4‐derived **PBD** units or the sequence/relative positioning of the functional groups along individual polymer chains. The FD (mol%) depended on both the cyclic ether and the nucleophile, highlighting that both molecular editing components influence the final polymer composition. Across all three cyclic ether‐derived subsets, sulfonamide FDs ranged from 20 to 34 mol% for the THF series (**P1**–**P4**), 11 to 34 mol% for the 2‐MeTHF series (**P7**–**P10**), and 17 to 39 mol% for the THP series (**P13**–**P16**) (Table S17). Within each cyclic ether‐derived subset, the FD varied non‐monotonically with sulfonamide identity, highlighting the pronounced influence of the nucleophile on functionalization efficiency. The observed differences in FD values can be rationalized by the electrophilic multicomponent editing mechanism, described in the literature [17], which proceeds through initial bromonium ion formation at the **PBD** C═C bonds, followed by attack of the cyclic ether and nucleophilic ring opening of the resulting oxonium intermediate. Accordingly, one cyclic ether molecule is incorporated per functionalized C═C double bond. Although NBS can promote allylic bromination under radical conditions [20], the present reaction is conducted without intentional radical initiation and under conditions established for electrophilic alkene aminoalkoxylation [17]; minor competing bromination pathways cannot, however, be excluded. Furthermore, the resulting FD values are used as ensemble‐average composition descriptors of the individual library members; differences between individual FD values are therefore interpreted descriptively rather than as statistically significant pairwise differences. Notably, this variation in FD constitutes an additional compositional dimension of the resulting polymer library and is not treated as a constant background parameter. Accordingly, subsequent property comparisons consider FD as part of the experimentally generated material state and do not attribute the observed responses exclusively to cyclic ether or nucleophile identity. The cyclic ether may influence ring‐opening energetics and intermediate stability, whereas the nucleophile governs trapping of the oxonium intermediate through differences in its steric and electronic properties. The non‐monotonic incorporation trends observed for the sulfonamide series indicate that functionalization is controlled by the interplay of nucleophile reactivity, intermediate stability, steric accessibility, and polymer solubility, instead of a simple electronic effect.

The formation of the sulfonate ester derivatives was similarly confirmed by NMR and FTIR spectroscopy (with representative spectra of **P11** and **P12** shown in Figure 2b). As expected, the sulfonate ester derivatives lacked N‐H stretching vibrations characteristic of the sulfonamide derivatives, while retaining the characteristic S═O absorption bands. The corresponding average FDs, estimated by the same internal‐standard qNMR procedure used for the sulfonamide‐functionalized polymers, were consistently higher for the sulfonate ester derivatives. For all three cyclic ethers, the *p*‐toluenesulfonic acid‐derived polymer exhibited higher incorporation than its methanesulfonic acid‐derived counterpart, yielding 25 and 38 mol% for **P5** and **P6**, 40 and 59 mol% for **P11** and **P12**, and 58 and 68 mol% for **P17** and **P18**, respectively (Table S17). In addition, the sulfonate ester FD increased systematically across the cyclic ether series, following the trend THF < 2‐MeTHF < THP for both sulfonic acid nucleophiles. Within the proposed reaction pathway, this trend may reflect differences in oxonium‐ion stability and nucleophile accessibility, together with polymer‐reaction‐medium interactions associated with the respective cyclic ethers. Notably, the THP‐derived sulfonate ester polymers **P17** and **P18** exhibited the highest FD values within the entire library, with **P18** reaching 68 mol%. Consequently, these results show that the multicomponent editing strategy provides not only structural diversity but also a tunable incorporation landscape governed by both nucleophile class and cyclic ether identity.

Successful incorporation of the cyclic ether component was further verified by 2D NMR spectroscopy. The Heteronuclear Single Quantum Coherence (HSQC) spectrum of **P7** (Figure 2c) displayed the expected correlation between the characteristic carbon resonance at ∼80 ppm and adjacent proton resonances of the incorporated ether unit at 4.1 and 3.3 ppm. Furthermore, ^13^C NMR confirmed the incorporation of **THF**, **2‐MeTHF**, and **THP‐**derived spacer units throughout the library (Section SC.1).

Finally, Size‐Exclusion Chromatography (SEC) analysis in THF provided complementary evidence for changes in the hydrodynamic behavior following molecular editing relative to the polybutadiene precursor. Compared to pristine **PBD** (*M*_n_ = 8900 g mol^−^^1^, *Đ* = 1.06), all edited polymers exhibited increased apparent molar masses (Figure 2d and Table S17). The edited polymers exhibited apparent dispersities ranging from *Đ* = 1.08 to 1.93 (Figure 2d and Table S17). Several polymers retained comparatively narrow apparent distributions, including **P1** and **P2** (*Đ* = 1.08) and **P12** (*Đ* = 1.12), whereas broader distributions were observed for **P9** (*Đ* = 1.48), **P15** (*Đ* = 1.53), **P13** (*Đ* = 1.65), and **P5** (*Đ* = 1.93). These SEC‐derived values may reflect both differences in molar‐mass distribution and differences in hydrodynamic behavior and chromatographic interactions arising from the introduced functionalities. Accordingly, the reported *M*_n_ and *Đ* values are treated as apparent SEC‐derived characterization descriptors.

The condition dependence of the SEC‐derived distributions is further illustrated by **P3**, for which analysis under alternative SEC conditions in DMAc yielded a different apparent distribution compared with the corresponding THF measurement (Figure S136). Accordingly, differences in apparent molar‐mass characteristics are recognized as an additional component of the resulting material‐state description and are not assumed to be constant across the library.

Hence, the combined NMR, FTIR, HSQC, and SEC analyses confirm the successful construction of the 18‐member polymer library. The molecular editing strategy thus provides access to a chemically diverse yet structurally related polymer library for systematically investigating structure‐property‐function relationships.

### 2.2 Thermal Property Landscape of the Polymer Library

The influence of molecular editing on the thermal behavior of **PBD** was evaluated by differential scanning calorimetry (DSC) and thermogravimetric analysis (TGA). The glass transition temperature (*T*_g_) and thermal degradation temperature at 10% mass loss (*T*_d,10%_) values were determined for all 18 library members (cf. Figures S148–S150, S152–S166, respectively and Table S17; the thermograms of P1‐P3 were previously reported in Ref. [13]). The resulting thermal property landscapes are summarized in Figure 3. The color coding represents the magnitude of each measured property at the corresponding cyclic ether‐nucleophile combination and is intended to facilitate comparison of the response patterns across the common molecular‐input grid, and it does not imply a direct correlation between *T*_g_ and *T*_d,10%_.

**FIGURE 3:**
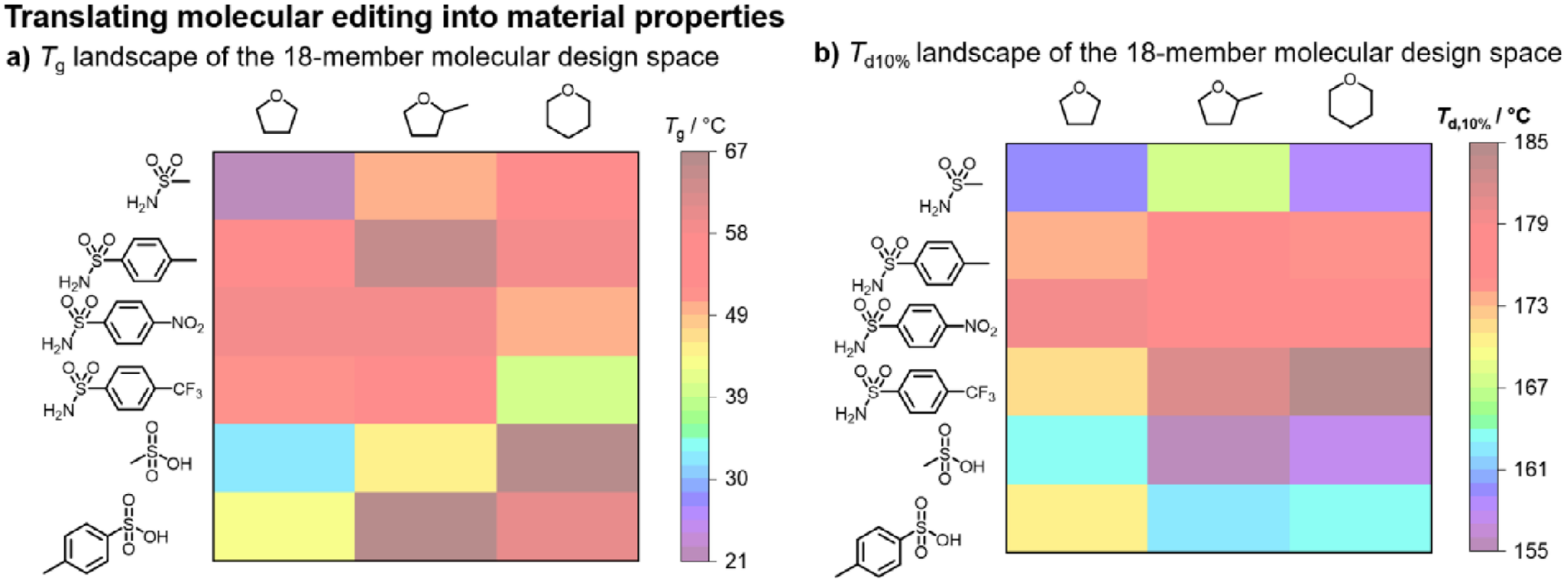
Thermal property landscapes of the 18‐member polymer library: (a) Glass transition temperature (*T*_g_) and (b) degradation temperature at 10% mass loss (*T*_d,10%_) values, respectively. Rows correspond to the incorporated nucleophiles (**MeSO_2_NH_2_**, ***p*‐TsNH_2_**, ***p‐*NsNH_2_**, ***p*‐CF_3_BsNH_2_**, **MSA**, and ***p‐*TsOH** from top to bottom), while columns represent the cyclic ether used during molecular editing. The left, middle, and right columns correspond to the **THF**‐derived polymers (**P1**‐**P6**), **2‐MeTHF**‐derived polymers (**P7**‐**P12**), and **THP**‐derived polymers (**P13**‐**P18**). *T*_g_ values range from 21°C to 67°C and *T*_d,10%_ values range from 155°C to 185°C, respectively. The color maps visualize the distribution of each measured thermal property across the common 3 × 6 molecular‐input space; similarity or dissimilarity between the maps therefore reflects whether the two properties respond similarly or differently to the same molecular inputs and does not represent a direct *T*_g_‐*T*_d,10%_ correlation.

Molecular editing produced a broad thermal‐property landscape across the polymer library, with *T*_g_ values ranging from 21°C (**P1**) to 67°C (**P12** and **P17**), while *T*_d,10%_ values varied between 155°C (**P11**) and 185°C (**P16**). These results demonstrate that relatively small variations in the molecular editing components (i.e., the cyclic ether and nucleophile) can substantially influence the thermal behavior of the edited polymers. The *T*_g_ landscape revealed a clear dependence on the cyclic ether‐derived spacer, although the magnitude and direction of this effect depended on the nucleophile identity. For several nucleophile families, the transition from THF‐ to 2‐MeTHF‐ and THP‐derived structures resulted in higher glass transition temperatures. In the methanesulfonamide series, *T*_g_ increased from 21°C for **P1** to 50°C for **P7** and 53°C for **P13**, respectively. Similarly, the MSA‐derived sulfonate ester series showed *T*_g_ values of 32°C, 45°C, and 67°C for **P5**, **P11**, and **P17**, respectively. The *p*‐toluenesulfonate series exhibited comparable behavior, with *T*_g_ values of 43°C, 67°C, and 60°C for **P6**, **P12**, and **P18**, respectively. Although the increase was not strictly monotonic in the latter series, both the 2‐MeTHF‐ and THP‐derived polymers exhibited higher *T*_g_ values than the corresponding THF‐derived polymer. These variations likely reflect differences in side‐chain architecture, steric environment, segmental mobility, and intermolecular interactions introduced by the cyclic ether‐derived spacers. However, the influence of spacer structure was not universal across all nucleophile families. For example, the *p*‐nitrobenzenesulfonamide derivatives **P3**, **P9**, and **P15** displayed *T*_g_ values of 58°C, 58°C, and 50°C, respectively, while the *p*‐CF_3_‐substituted sulfonamide derivatives **P4**, **P10**, and **P16** exhibited values of 51°C, 53°C, and 40°C, respectively. Since each cyclic ether‐nucleophile combination generates a distinct material state that also differs in average functionalization degree and apparent molar‐mass characteristics, the thermal response is not a simple one‐parameter correlation with either molecular input. Instead, the complete molecular design space is used below to compare descriptively the relative contributions associated with cyclic ether identity, nucleophile identity, and residual and non‐additive variation. Thus, these results demonstrate that variation of both molecular editing components provides access to a broad range of thermal properties from the same parent polymer scaffold.

The thermal stability landscape showed a different dependence on molecular structure. All library members displayed degradation temperatures at or above 155°C. The highest thermal stabilities were observed for several aromatic sulfonamide‐containing polymers, including **P3**, **P9**, **P10**, and **P16**, with *T*_d,10%_ values of 179°C, 178°C, 181°C, and 185°C, respectively. The enhanced thermal stability of these materials may be associated with their aromatic substituents and differences in intermolecular interactions. In contrast, the sulfonate ester‐containing polymers generally displayed lower thermal stabilities. The MSA‐derived sulfonate ester polymers **P5**, **P11**, and **P17** exhibited *T*_d,10%_ values of 163°C, 155°C, and 157°C, respectively, while **P6**, **P12**, and **P18** showed values of 170°C, 162°C, and 163°C, respectively. Due to the presence of bromine moieties, the polymer‐bound Br content was additionally estimated using the average‐composition model employed for the qNMR analysis (Section SA.4 and Table S17). The resulting model‐derived Br contents span 26‐52 wt.% across the library. However, Br content is not independently varied from the other compositional characteristics of the polymers, and its specific contribution to thermal stability therefore cannot be isolated. For example, the THP‐derived polymers **P15**, **P16**, and **P17** each contain approximately 33 wt.% model‐derived Br but exhibit *T*_d,10%_ values of 175°C, 185°C, and 157°C, respectively. Thus, bromine loading alone does not account for the observed thermal‐stability landscape, and a contribution from dehydrohalogenation/HBr elimination cannot be excluded. Accordingly, the *T*_g_ and *T*_d,10%_ landscapes are non‐congruent, indicating that the two thermal characteristics respond differently to the molecular editing variables. For example, **P12** and **P17**, both with *T*_g_ values of 67°C, exhibited only moderate thermal stability, with *T*_d,10%_ values of 162°C and 157°C, respectively. Conversely, **P16** displayed the highest thermal stability of the library (*T*_d,10%_ = 185°C) despite a comparatively low *T*_g_ of 40°C. These non‐congruent response patterns indicate that glass‐transition behavior and thermal degradation are governed by different combinations of material‐state characteristics and illustrate the multidimensional nature of the molecular design space.

To evaluate whether the observed thermal behavior was primarily governed by the extent of functionalization, *T*_g_ was plotted against FD determined by qNMR/internal‐standard analysis and mass‐balance calculations (Figure S151). No strong linear correlation between FD and *T*_g_ was observed (*R* ^2^ = 0.17). For example, polymers with similar functionalization levels (such as **P1** and **P10**) displayed substantially different *T*_g_ values, whereas **P18,** which exhibited the highest estimated incorporation, did not show the highest *T*_g_.

The thermal property landscape revealed that systematic variation of both cyclic ether and nucleophile identity enables substantial tuning of the thermal properties of the molecularly edited polymers. More importantly, the absence of a simple one‐parameter correlation emphasizes that these properties emerge from the combined influence of multiple molecular design variables, including spacer architecture, nucleophile identity, functionalization level, and intermolecular interactions. Accordingly, differences in thermal behavior cannot be assigned independently to cyclic ether or nucleophile identity alone, because the molecular editing variables also generate differences in functionalization level. The resulting properties are therefore interpreted as responses of the complete material state, arising from the combined influence of spacer architecture, nucleophile‐derived functionality, functionalization level, and intermolecular interactions.

### 2.3 The Influence of Molecular Editing on Nanoscale Organization

To investigate whether molecular editing influences nanoscale organization and molecular mobility, selected library members were characterized by transmission electron microscopy (TEM), atomic force microscopy (AFM), and low‐field ^1^H NMR relaxometry. **P9** was initially selected because of the high polarity of the *p*‐nitrobenzenesulfonamide functionality, the anticipated electron contrast in TEM, and its relatively high FD of 34 mol% (Table S17, entry 9). **P8** and **P9** were then selected for comparison because both were generated using the same **2‐MeTHF**‐derived spacer but different sulfonamide nucleophiles. TEM analysis of bulk samples prepared by microtomy revealed markedly different nanoscale contrasts for the two polymers (Figure 4a,b).

**FIGURE 4:**
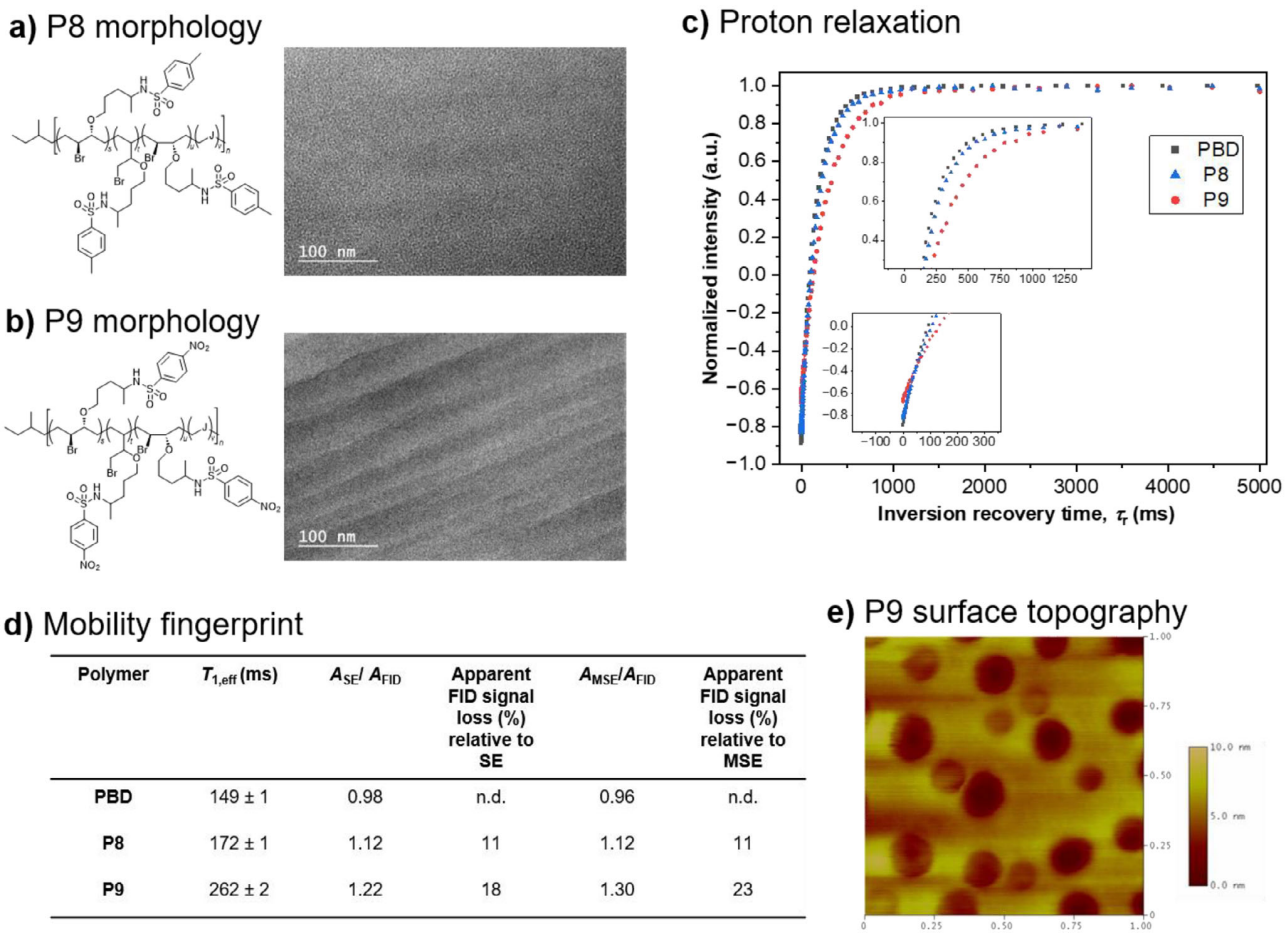
Morphology and molecular mobility of selected molecularly edited PBD derivatives. (a,b) Chemical structures and representative TEM images of **P8** and **P9**, respectively. (c) Normalized ^1^H inversion–recovery profiles of pristine **PBD**, **P8**, and **P9** measured by low‐field NMR relaxometry. Insets highlight the early‐ and intermediate‐time regions of the recovery curves. (d) Comparative mobility fingerprint summarizing *T*_1,eff_, solid‐echo (SE)‐to‐FID and magic‐sandwich echo (MSE)‐to‐FID amplitude ratios, and the corresponding apparent FID signal losses. Apparent signal‐loss values are used as comparative pulse‐sequence‐dependent descriptors (see Section SC.8 for details regarding the methodology). (e) Representative AFM height image of **P9**, revealing heterogeneous surface topography and nanoscale domains; Feret‐diameter analysis gave an average apparent domain diameter of 207 nm.

Whereas **P8** displayed comparatively weak contrast, **P9** exhibited pronounced, regularly spaced contrast features with a characteristic separation of approximately 30 nm (Figure S167). Although the precise origin of this contrast cannot be assigned unambiguously from TEM alone, the comparison indicates that variation of the incorporated sulfonamide functionality may substantially influence nanoscale organization. Importantly, the same qualitative trend was observed for the **THF**‐derived analogues **P2** and **P3** (Figures S168 and S169). As observed for **P8** and **P9**, the *p*‐nitrobenzenesulfonamide‐containing polymer **P3** exhibited more pronounced nanoscale contrast than its *p*‐toluenesulfonamide counterpart **P2**, although the contrast in **P3** was slightly weaker than that observed for the corresponding **2‐MeTHF**‐derived polymer **P9**. The recurrence of this qualitative trend across both cyclic‐ether‐derived spacer architectures suggests that the identity of the sulfonamide substituent contributes to the observed nanoscale organization, while the spacer structure may modulate its extent. The stronger contrast associated with the nitro‐substituted polymers may partly reflect their higher heteroatom content and associated electron contrast, but differences in intermolecular interactions and organization may also contribute; these effects cannot be distinguished from the present TEM data alone. Because nucleophile identity also influences FD, model‐derived Br content, and other characteristics of the resulting material state, this comparison does not permit the independent contribution of brominated motifs to the observed nanoscale organization to be isolated. Motivated by the particularly distinct TEM contrast of **P9**, its surface topography was further examined by AFM (Figure 4e).

The height image revealed a heterogeneous thin‐film surface containing discrete nanoscale domains, with Feret‐diameter analysis yielding an average apparent domain diameter of approximately 207 nm. This length scale is substantially larger than the approximately 30 nm characteristic contrast spacing observed by TEM and is therefore not interpreted as representing the same structural feature. Rather, the two techniques provide complementary views of differently prepared material states: TEM was performed on sections representative of the bulk polymer, whereas AFM probes the surface morphology of a deposited thin film. The difference between the bulk and thin‐film morphologies may arise from confinement and processing effects during film formation. Accordingly, the characteristic length scales obtained by TEM and AFM can not be directly correlated. Together, however, the measurements have indicated that **P9** exhibits structural heterogeneity across different nanoscale length scales and material geometries.

To assess whether the observed morphological differences are accompanied by changes in proton dynamics, **P8** and **P9** were further compared with pristine **PBD** by low‐field ^1^H NMR relaxometry (Figure 4c,d and Figure S171). Monoexponential analysis of the inversion–recovery profiles yielded effective longitudinal relaxation times, *T*_1,eff_ of 149 ± 1, 172 ± 1, and 262 ± 2 ms for pristine **PBD**, **P8**, and **P9**, respectively (the fitting method and all calculation details are provided in Section SC.8). The substantially longer *T*_1,eff_ observed for **P9** has indicated a distinctly different proton relaxation environment compared with both pristine **PBD** and **P8**. Because *T*_1_ depends on molecular motions occurring on the relevant relaxation timescale, these values are treated as comparative effective relaxation parameters rather than as direct measures of polymer rigidity. Moreover, comparison of direct free‐induction decay (FID), solid‐echo (SE), and magic‐sandwich echo (MSE) signals provided a complementary probe of rapidly dephasing proton contributions. Relative to the direct FID, **P8** exhibited an apparent signal loss of approximately 11% when referenced to either SE or MSE, whereas the corresponding losses for **P9** were 18% and 23%, respectively (Figure 4d and Section SC.8 for calculation details). No positive apparent signal loss was observed for pristine **PBD**. Because these values depend on the pulse sequence and experimental time window, they are not interpreted as absolute rigid‐ or mobile‐proton fractions. Instead, the combined *T*_1,eff_ and FID/SE/MSE measurements provide a comparative mobility fingerprint, with **P9** displaying both the most distinct longitudinal relaxation behavior and the largest apparent signal losses associated with rapidly dephasing proton contributions. Thus, the TEM, AFM, and low‐field ^1^H NMR results indicate that molecular editing extends beyond chemical diversification to influence both nanoscale organization and proton dynamics. The recurrence of related TEM trends across the **THF**‐ and **2‐MeTHF**‐derived polymer pairs further indicates that these effects are not restricted to a single spacer architecture, revealing a multidimensional molecular design space in which the identities of both the sulfonamide substituent and cyclic‐ether‐derived spacer can influence higher‐order material properties. These structure‐dependent differences further motivate investigating a selected library member as a functional material for a technologically relevant application, linking molecular editing to performance in a real‐world materials context.

### 2.4 Application of **P11** as a Functional Electrolyte Additive for Li–S Batteries

**P11** was selected as a representative candidate based on a combination of chemical functionality and material properties. In particular, **P11** contains aliphatic sulfonate ester functionalities, providing sulfur‐ and oxygen‐rich polar motifs with potential relevance to electrolyte/electrode interfaces. Sulfur‐containing functionalities are well‐established electrolyte‐active motifs in lithium batteries, where they can participate in interphase formation, and related sulfonate‐containing additives have also been shown to influence polysulfide speciation and interfacial processes in Li–S systems [21]. The sulfonate ester functionality presents an electrophilic carbon site (C─O) that is, in principle, susceptible to nucleophilic substitution by polysulfide anions (S_n_^2^^−^). Precedent for such reactivity exists: Ling et al. [22] demonstrated that sulfate leaving groups (R–OSO_3_^−^) on polymer binders undergo nucleophilic attack by lithium polysulfides, forming covalent C–S bonds that anchor polysulfide intermediates. The sulfonate ester (mesylate) functionality present in **P11** is a closely related and comparably effective leaving group in nucleophilic substitution reactions. This literature precedent provides a plausible, albeit unconfirmed, chemical basis for interaction with polysulfide species. At the same time, **P11** occupies an intermediate position within the molecular library: its FD of 40 mol% lies between, for example, those of the other aliphatic sulfonate ester functionalized derivatives **P5** (25 mol%) and **P17** (58 mol%), and its *T*_g_ of 45°C similarly falls within the intermediate range of the investigated materials. **P11** additionally exhibits a relatively narrow molar‐mass distribution (*Đ* = 1.17) and a *T*_d,10%_ of 155°C, well above the typical operating temperature range of Li–S cells. These characteristics made **P11** a suitable candidate for assessing whether the molecularly edited PBD platform could translate its tunable structural and physical properties into electrochemical performance in a technologically relevant Li–S battery environment.

The need to investigate alternative electrolyte additives arises from the limitations of lithium nitrate (LiNO_3_), a widely used additive in Li–S batteries. LiNO_3_ contributes to the formation of a protective interphase on the lithium‐metal anode, thereby mitigating parasitic reactions between soluble polysulfides and lithium and improving Coulombic efficiency. However, this passivation strategy addresses the consequences of polysulfide migration at the anode rather than the underlying dissolution and diffusion of polysulfides themselves. Furthermore, LiNO_3_ is progressively consumed during cycling, and its decomposition may contribute to gas‐generating side reactions [23]. Therefore, there is interest in exploring alternative electrolyte additives that may mitigate polysulfide‐related parasitic reactions without relying on LiNO_3_. In this study, **P11** was evaluated as a potential additive in a LiNO_3_‐free ether‐based electrolyte. As mentioned above, **P11** contains sulfonate ester groups (Figure 5a), which could potentially interact with polysulfide species. Such interactions could affect polysulfide solubility, transport, or conversion in Li─S cells. However, the nature and strength of these interactions were not directly investigated in the present study. Accordingly, **P11** was evaluated experimentally as a potential electrolyte additive.

**FIGURE 5:**
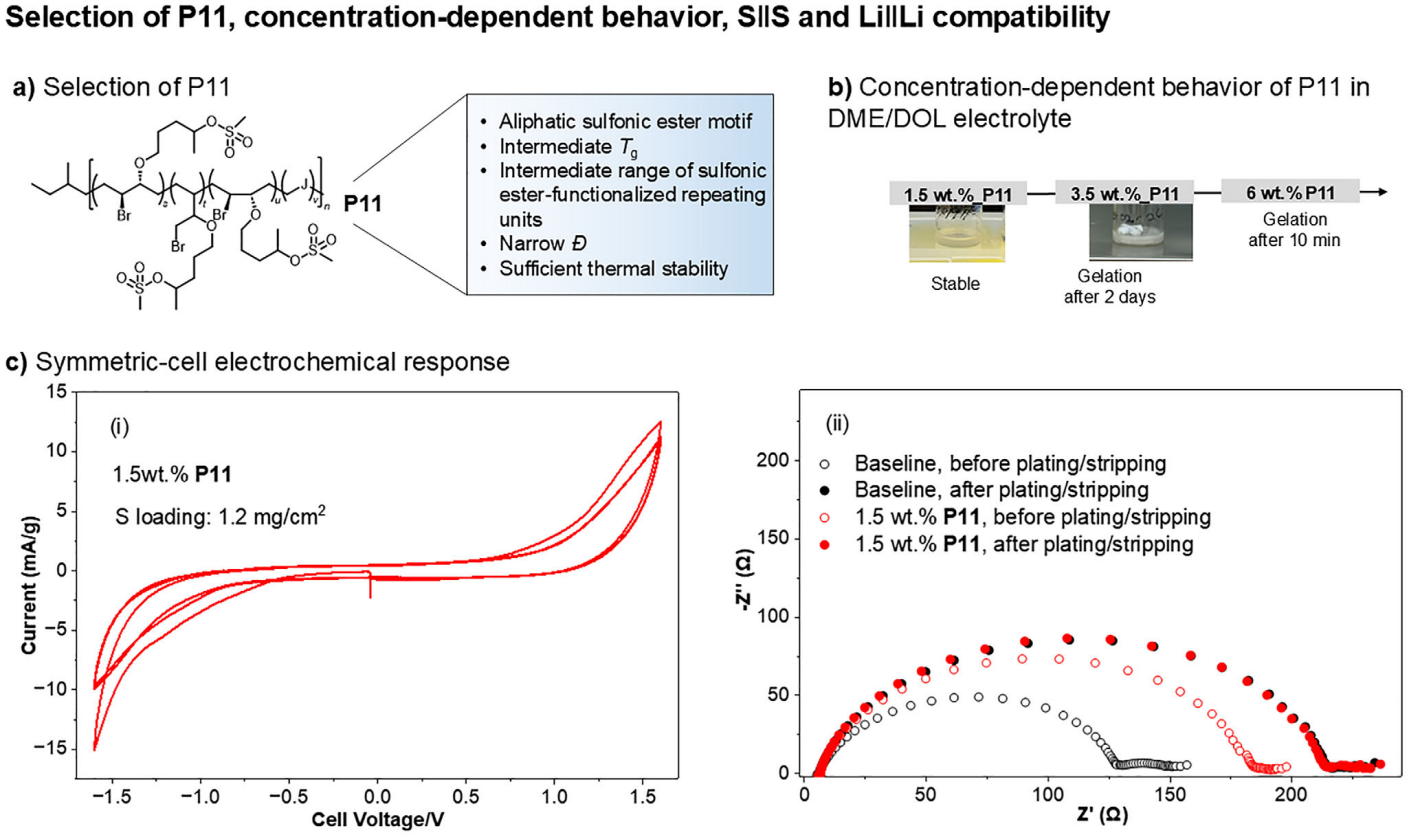
Selection of **P11**, concentration‐dependent electrolyte behavior, electrochemical response in S‖S and Li‖Li symmetric cells. (a) Selection of **P11** based on its combination of sulfonate ester functionality, narrow molar mass dispersity, intermediate *T*_g_, and sufficient thermal stability. (b) Concentration‐dependent behavior of **P11** in 1 M LiTFSI DME/DOL electrolyte, showing a homogeneous electrolyte at 1.5 wt.% and gel formation at higher concentrations. (c) Electrochemical response in symmetric cells: (i) cyclic voltammetric response of **P11**‐containing electrolyte in a symmetric S‖S cell and (ii) impedance spectra of Li||Li cells before and after three full cycles of plating/stripping.

As described in Section SB.9, **P11** is fully soluble in the baseline electrolyte (1 m LiTFSI in DME/DOL, 1:1 v/v) and exhibits pronounced concentration‐dependent behavior (Figure 5b). At a concentration of 1.5 wt.%, homogeneous solutions were obtained, whereas increasing the concentration to 3.5 wt.% resulted in gelation after approximately two days. At 6 wt.%, gelation occurred within minutes after preparation. This concentration screening served exclusively to establish the solubility and physical compatibility of **P11** with the electrolyte; the 3.5 and 6 wt.% formulations gelled and were therefore not subjected to any electrochemical testing. All electrochemical measurements reported in this work, i.e., cyclic voltammetry, electrochemical impedance spectroscopy, and galvanostatic cycling, were performed using a single **P11** concentration of 1.5 wt.%. The spontaneous, concentration‐dependent gelation observed at 3.5 wt.% and 6.0 wt.% **P11** in the absence of any applied electrochemical potential suggests that **P11** actively alters the physical state of the electrolyte at elevated concentrations. The molecular origin of this gelation, however, cannot be established from this observation alone. One plausible explanation is **P11‐** induced cationic ring‐opening polymerization of DOL, considering the precedent of Lewis acid‐induced polymerization reported for Al(OTf)_3_, LiDFOB, and BF_3_ in ether‐based electrolytes [18, 24]. Alternatively, gelation could involve direct interactions or reactions involving the functionalities of **P11,** or a combination of processes. Further studies, including solvent‐specific controls (DME‐only *vs*. DOL‐only) and FTIR analysis, are required to distinguish between **P11**‐induced DOL polymerization, direct nucleophilic substitution involving the functional groups of **P11**, or the coexistence of both mechanisms.

To explore the electrochemical response of the **P11**‐containing electrolyte at the sulfur‐electrode environment while excluding contributions from lithium metal, cyclic voltammetry was performed using a symmetric S||S cell (Figure 5ci). A relatively low current response was observed from approximately ‐0.7 V through +0.7 V, followed by a pronounced increase in current outside this voltage range. Because the measurement was conducted in a two‐electrode configuration without a reference electrode, the contributions of the individual electrodes and the origin of the observed currents cannot be unambiguously resolved. Accordingly, this experiment provides an exploratory assessment of the overall electrochemical response of the **P11**‐containing electrolyte in the sulfur electrode environment, rather than a mechanistic evaluation of sulfur conversion or **P11** stability. To investigate the influence of **P11** on the lithium‐metal/electrolyte interface, EIS measurements were performed using Li||Li symmetric cells containing either the baseline electrolyte or the baseline electrolyte with 1.5 wt.% **P11** (Figure 5cii). Before lithium plating/stripping, the cells exhibited different impedance responses, with apparent arc diameters of approximately 130 Ω for the baseline electrolyte and 180 Ω for the **P11**‐containing electrolyte, as estimated from the Nyquist plots. After plating/stripping, both spectra showed larger and similar apparent arc diameters of approximately 215 Ω. Thus, under the investigated conditions, **P11** initially altered the impedance response of the Li||Li cell, whereas no clear additional impedance contribution associated with **P11** was observed after plating/stripping. However, EIS alone cannot determine whether **P11** decomposes or participates in the formation of the solid electrolyte interphase (SEI) at the lithium electrode.

To evaluate whether **P11** fulfills its proposed role as an electrolyte additive, its impact on Li‐S cell behavior was investigated using complementary electrochemical techniques. Based on the functional groups present in **P11**, the additive was hypothesized to interact with soluble polysulfide species and thereby affect sulfur conversion and polysulfide migration. However, the present electrochemical measurements do not directly demonstrate these molecular interactions. Cyclic voltammetry of the full Li─S cells shown in Figure 6ai reveals that the addition of 1.5 wt.% **P11** substantially alters the sulfur redox response compared with the baseline cell. Specifically, the redox peaks become broader, and the separation between anodic and cathodic peaks increases, indicating a more polarized electrochemical response in the presence of **P11**. The Nyquist plots shown in Figure 6aii additionally reveal pronounced differences in the impedance response. Both cells exhibit a comparable high‐frequency intercept of approximately 6 Ω, indicating comparable overall ohmic resistance under the investigated conditions. In contrast, the apparent arc diameter, estimated visually from the Nyquist plots, increases from approximately 130 Ω for the baseline cell to approximately 315 Ω for the **P11**‐containing cell prior to cycling, indicating that **P11** modifies the overall impedance response. Nonetheless, the molecular origin of this increased impedance cannot be resolved by EIS alone. After five CV cycles, the apparent arc diameter for the **P11**‐containing cell decreases to approximately 140 Ω, indicating a substantial change of the impedance response during the initial cycling. This change could reflect improved electrode wetting, activation of the sulfur cathode, and/or restructuring of an additive‐derived surface layer, although EIS alone cannot distinguish between these possibilities.

**FIGURE 6:**
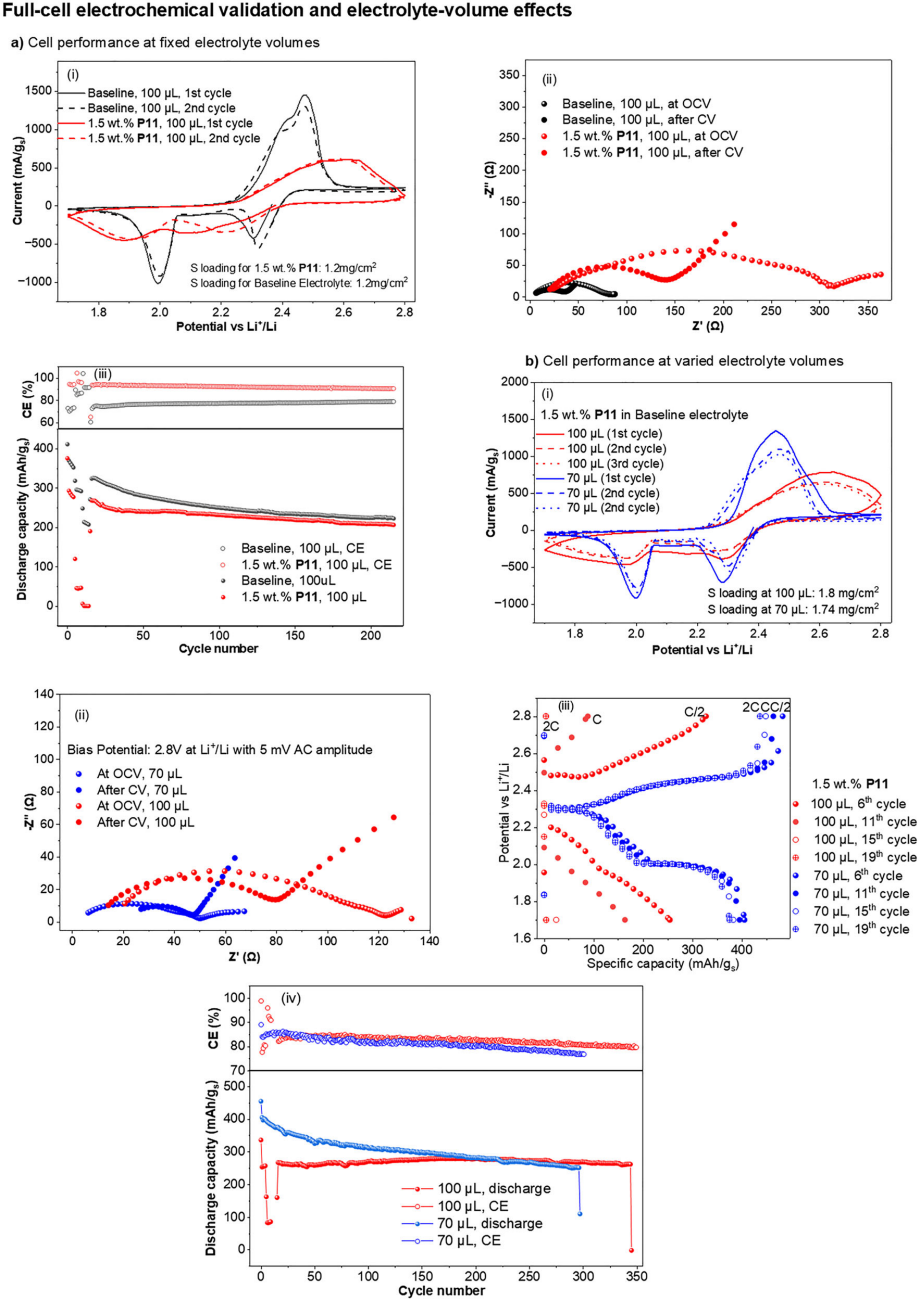
Full‐cell electrochemical performance of **P11**‐containing Li‐S cells and electrolyte‐volume effects. (a) Comparison of the baseline electrolyte and the electrolyte containing 1.5 wt.% **P11**: (i) cyclic voltammetric response, (ii) Nyquist plots before and after CV cycling, and (iii) long‐term cycling performance. (b) Effect of electrolyte volume on cells containing 1.5 wt.% **P11**: (i) cyclic voltammetric response, (ii) Nyquist plots before and after CV cycling, (iii) rate performance and corresponding voltage profiles, and (iv) long‐term cycling performance.

The influence of the altered polarization and impedance response is also reflected in the cycling performance shown in Figure 6aiii. The **P11**‐containing cell exhibits a lower initial discharge capacity of approximately $275\;{\mathrm{mAhg}}_{s^{-1}}$ than the baseline cell. However, the long‐term electrochemical behavior reveals a different trend. The cell containing the baseline electrolyte undergoes continuous capacity decay accompanied by a relatively low Coulombic efficiency (CE) of approximately 78%, a pattern typically associated with persistent polysulfide shuttling and irreversible loss of active material in Li–S cells. In contrast, the **P11**‐containing cell exhibits a considerably slower capacity‐fading rate together with a higher CE of ∼93%, although a gradual decline is observed during extended cycling. The improved CE and enhanced capacity retention are consistent with a reduction in parasitic processes associated with soluble polysulfide species over extended cycling, although the present data do not directly establish suppression of polysulfide migration or stabilization of soluble sulfur species as the underlying cause. Altogether, these observations indicate an electrochemical trade‐off: while the additive initially increases polarization and the apparent impedance response, it is associated with higher Coulombic efficiency and more gradual capacity decay over extended cycling. Meanwhile, it is important to highlight that the coulombic efficiencies should be interpreted within the context of the deliberately LiNO_3_‐free and electrolyte‐rich model system employed here. Low Coulombic efficiencies are well documented for Li–S cells using 1 M LiTFSI in DOL/DME without LiNO_3_; Ding et al. [25] reported an initial CE of only 66.4% at an electrolyte volume of 80 µL in this formulation, with a high CE of 99.1% achieved only at a substantially reduced volume of 10 µL. Furthermore, cycling efficiency is known to decline with increasing electrolyte‐to‐sulfur ratio, even in LiNO_3_‐containing cells [26]. The E/S ratios employed in this work (∼49 and ∼36 µL mg^−1^) place the cells firmly in the electrolyte‐rich regime, where polysulfide shuttle losses are expected to be pronounced. The comparison therefore evaluates the relative influence of **P11** against the additive‐free baseline under identical conditions and does not establish equivalence or superiority to conventional LiNO_3_‐containing Li–S electrolytes. Although **P11** increases CE relative to the baseline, it does not suppress parasitic shuttle processes to an extent that would justify its use as a stand‐alone shuttle‐inhibiting additive.

In line with the aforementioned evidence from the literature, the electrochemical response of Li–S cells containing 1.5 wt.% **P11** also depends on electrolyte volume, as shown by comparing cells with electrolyte volumes of 100 µL [electrolyte‐to‐sulfur (E/S) ratio ≈ 49 µL mg^−1^] and 70 µL (E/S ≈ 36 µL mg^−1^). The cyclic voltammograms (Figure 6bi) show broad, low‐magnitude Faradaic features for the cell containing 100 µL, whereas the cell containing 70 µL exhibits sharper, higher‐current peaks at ∼2.3 and ∼2.0 V vs. Li^+^/Li. Because the GFA separator and electrode architecture were identical under both conditions, and both electrolyte volumes are expected to provide adequate cell wetting, differences in electrode/electrolyte contact are unlikely to account for the sharper response at the lower electrolyte volume. Instead, the different responses may reflect changes in the concentration and transport of soluble polysulfide species within the available electrolyte volume. At 100 µL, the larger electrolyte reservoir could favor greater spatial distribution and dilution of soluble intermediates, whereas at 70 µL, these species may remain more concentrated within the smaller volume. Such differences in polysulfide distribution and transport could contribute to the broader CV response at 100 µL and the sharper features at 70 µL. On this basis, the CV differences are consistent with electrolyte‐volume‐dependent effects on polysulfide concentration and transport and should not, by themselves, be interpreted as evidence for faster intrinsic sulfur redox kinetics at the lower electrolyte volume. This interpretation is further supported by the Nyquist plot data (Figure 6bii). For cells containing 100 and 70 µL of electrolyte, corresponding to E/S ratios of approximately 49 and 36 µL mg^−1^, respectively, the high‐frequency intercept is similar (∼6 Ω), indicating comparable ohmic resistance. However, the overall apparent impedance diameter is smaller for the cell containing 70 µL (∼50 Ω at OCV, ∼25 Ω after CV) than for the cell containing 100 µL (∼125 Ω at OCV, ∼80 Ω after CV). These values were estimated directly from the Nyquist plots and represent the total impedance response of the cell, comprising the high‐frequency intercept together with the overlapping features at lower frequencies; no equivalent‐circuit fitting was performed, and the values are therefore not assigned to any individual process such as charge transfer or interfacial film formation. Consistent with the lower apparent impedance, the 70 µL cell also exhibits a higher rate capability, delivering ∼375 mAh g s^−1^ at 2 C (Figure 6b iii). Importantly, this improved rate response does not by itself establish faster intrinsic sulfur redox kinetics and may instead reflect electrolyte‐volume‐dependent differences in interfacial transport and soluble‐species distribution. However, the advantage observed at lower electrolyte volume is not sustained during prolonged cycling. Long‐term cycling (Figure 6biv) shows that the cell containing 70 µL, despite its favorable early‐stage rate performance, undergoes a progressive decline in CE toward ∼78%, whereas the 100 µL cell sustains a higher CE (∼83%) beyond 345 cycles.

A single abrupt drop in capacity is observed near cycle 300 for the 70 µL cell; as no corresponding change is evident in the surrounding cycles or in the voltage response, this isolated point is attributed to a measurement artifact rather than to a genuine loss of cell function, and cycling of this cell was not continued beyond this point. This decline in CE at the lower E/S ratio is consistent with increased susceptibility to parasitic reactions during extended cycling at reduced electrolyte volume. Thus, these results suggest an electrolyte‐volume‐dependent trade‐off: reducing the electrolyte volume is associated with a lower initial impedance response and higher rate capability but with earlier capacity loss during prolonged cycling under the investigated conditions.

Finally, to integrate the multidimensional library and summarize the relationships between molecular inputs, resulting material states, and material responses, these datasets were combined into a unified framework (Figure 7). Variation of cyclic ether and nucleophile identity defines the molecular input space (Figure 7, panel 1), generating distinct material states characterized by functionalization degree (FD) and, for selected representatives, molecular mobility and nanoscale morphology (Figure 7, panel 2). These material states translate into distinct thermal‐property landscapes, while the evaluation of **P11** as an electrolyte additive provides a selected proof of application‐relevant function (Figure 7, panel 3). Importantly, the FD, *T*_g_, and *T*_d,10%_ landscapes are non‐congruent (Figure 7, panels 2A and 3), demonstrating that distinct material characteristics respond differently to the two molecular editing variables. FD varies predominantly with nucleophile identity, whereas *T*_g_ exhibits a more distributed dependence on both molecular inputs, and *T*_d,10%_ is strongly nucleophile‐dependent but essentially insensitive to cyclic ether identity. To summarize these non‐equivalent response patterns, a descriptive sums‐of‐squares analysis was applied to the complete 3 × 6 dataset for FD, *T*_g_, and *T*_d,10%_, partitioning the observed variation into contributions associated with cyclic ether identity, nucleophile identity, and remaining/non‐additive variation (Figure 7, panel 4; Section SC.9): nucleophile identity accounted for 67.6% and 82.6% of the observed variation in FD and *T*_d,10%_, respectively, whereas cyclic ether identity contributed only 12.5% and 0.5% (Table S18). In contrast, *T*_g_ showed more comparable contributions from cyclic ether (23.9%) and nucleophile identity (29.1%), together with substantial remaining or non‐additive variation (Table S18). The selected mobility and morphology measurements further illustrate how this multidimensional response extends beyond the library‐wide thermal properties. Under constant 2‐MeTHF conditions, changing the nucleophile from *p*‐TsNH_2_ in **P8** to *p*‐NsNH_2_ in **P9** increased *T*_1,eff_ from 172 to 262 ms and increased the apparent loss of rapidly relaxing proton signal. Similarly, the selected TEM comparisons revealed nucleophile‐dependent differences in nanoscale organization that recurred across both the THF‐ and 2‐MeTHF‐derived polymer pairs, while the presented data indicated a comparatively weaker effect of cyclic ether identity (Figure 7, panels 2B, 2C, and 4; Section SC.9). Accordingly, these non‐equivalent sensitivity profiles demonstrate that polymer composition, thermal behavior, molecular dynamics, and nanoscale organization cannot be predicted from a single molecular descriptor. The systematic molecular‐editing approach thus enables identification of which molecular inputs are most strongly associated with variation in specific material characteristics within the investigated design space. The resulting input–response relationships organize the library into a multidimensional structure‐property‐function map that can support the selection of materials with different property combinations.

**FIGURE 7:**
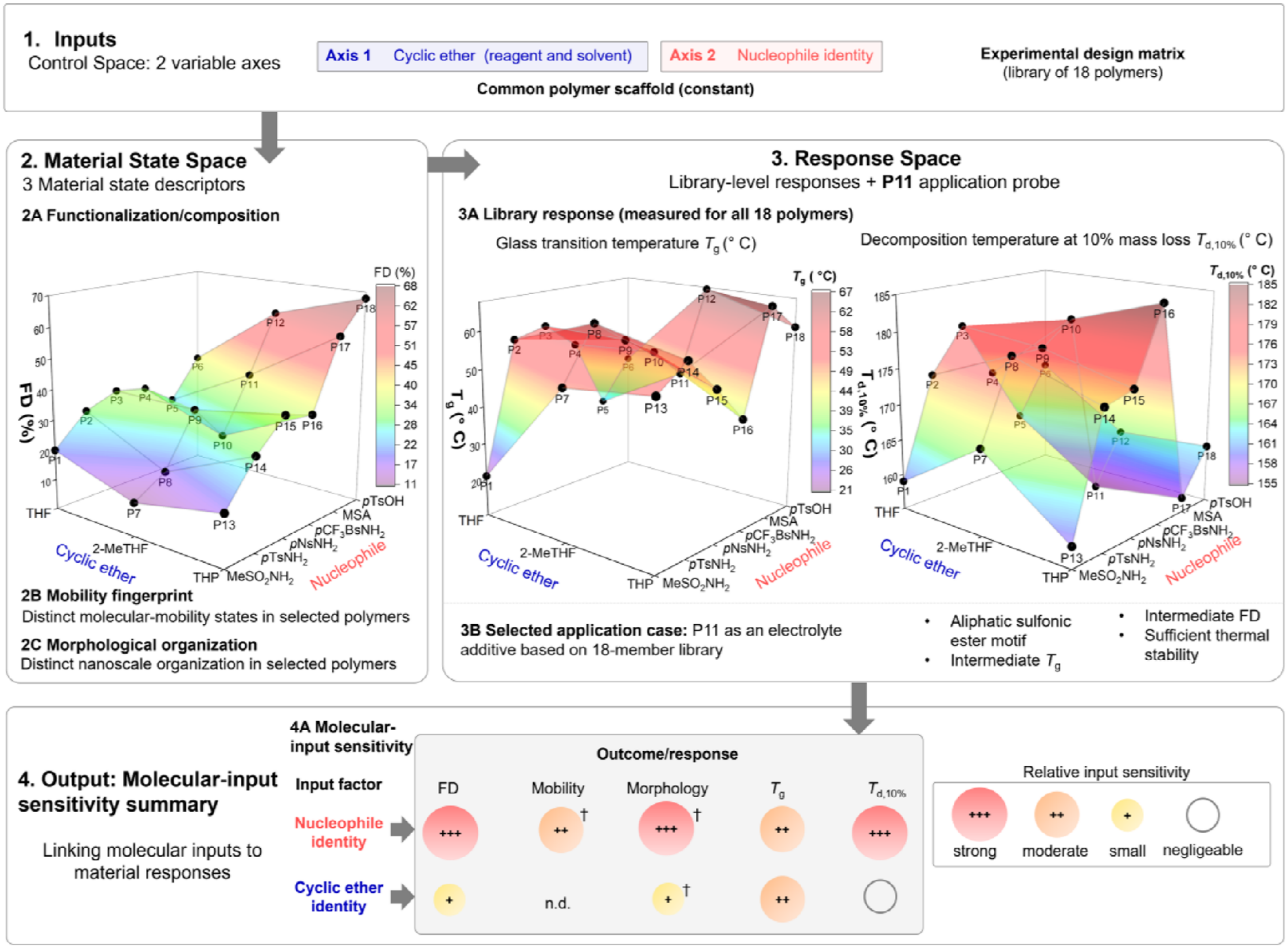
Molecular design space, material‐state descriptors, response landscapes, and molecular‐input sensitivity summary across the 18‐member polymer library. (1) Inputs: the control space is defined by two molecular editing variables, cyclic ether and nucleophile identity, while maintaining a common polymer scaffold. (2) Material State Space: molecular editing generates distinct material states characterized by functionalization degree (FD), molecular mobility, and nanoscale morphology; mobility and morphology were evaluated for selected representative polymers. (3) Response Space: library‐wide *T*_g_ and *T*_d,10%_ surfaces reveal distinct thermal‐property landscapes, while **P11** serves as a selected application probe for evaluating the library as a source of functional electrolyte additives. (4) Molecular‐input sensitivity summary: relative sensitivities of the measured material characteristics toward nucleophile and cyclic ether identity are summarized across the design space. For FD, *T*_g_, and *T*_d,10%_, classifications are derived from the descriptive sums‐of‐squares analysis of the complete 3 × 6 library; mobility and morphology were assessed qualitatively from selected representative polymers and are marked with †. The summary does not represent a pairwise statistical correlation matrix. O, negligible; +, weak; ++, moderate; +++, strong relative sensitivity; n.d., not determined. Calculation and classification are detailed in Section SC.9.

## 3 Conclusions

In summary, we have demonstrated that electrophilic multicomponent molecular editing provides a modular strategy for constructing chemically diverse polymer libraries from a common polybutadiene precursor. By systematically varying both cyclic ether and nucleophile identity, an 18‐member library with distinct compositional, thermal, morphological, and molecular‐mobility characteristics was generated. Mapping the resulting response landscapes revealed that relatively small molecular variations can induce substantial changes in material behavior and, importantly, that the different responses are not governed uniformly by the molecular input variables. Descriptive sensitivity analysis summarized the relative associations of the two molecular inputs with the measured response landscapes, revealing that nucleophile identity is associated with most of the observed variation in FD and *T*_d,10%_, whereas *T*_g_ displays a more distributed and non‐additive dependence on both cyclic ether and nucleophile identity. Selected mobility and morphology measurements further indicated substantial nucleophile‐dependent changes in molecular dynamics and nanoscale organization. Consequently, these non‐equivalent sensitivity profiles demonstrate that no single molecular descriptor, including FD, is sufficient to predict the full material‐property landscape. This library‐based analysis further guided the selection of **P11** as a candidate electrolyte additive with a balanced combination of chemical functionality and physicochemical properties. Subsequent preliminary evaluation in Li‐S cells employing a deliberately LiNO_3_‐free model electrolyte showed that **P11** alters the sulfur redox behavior and overall impedance response and is associated with higher coulombic efficiency and more gradual capacity decay relative to the additive‐free baseline. These effects are accompanied by increased polarization and reduced initial capacity accessibility, revealing an electrochemical trade‐off under the investigated conditions. These results provide a proof of concept that systematic exploration of a molecularly edited polymer library can be connected to the identification and evaluation of application‐relevant functional candidates, without establishing **P11** as an optimized or sufficient Li–S electrolyte additive.

Thus, at a broader level, this work establishes multicomponent molecular editing not only as a versatile synthetic methodology but also as a strategy for navigating multidimensional polymer design spaces and resolving molecular input–property‐function relationships. By systematically varying multiple molecular parameters within a common scaffold, this approach transforms polymer diversification from the generation of individual derivatives into the systematic mapping of design spaces that support comparative material selection and the identification of functional candidates. Current efforts are focused on elucidating the molecular origin of the concentration‐dependent gelation behavior of **P11** in DME/DOL‐based electrolytes and its influence on electrolyte organization. Beyond the present system, this modular molecular editing strategy offers opportunities for extension to commodity and waste‐derived polymers (such as vulcanized rubber), enabling the generation of functional polymer libraries and potentially accelerating the discovery of advanced materials for energy storage, separations, and circular polymer technologies.

## 4 Experimental Procedures

Details regarding the experimental procedures can be found in the supplemental experimental procedures.

## Author Contributions

J.J.: conceptualization, methodology, investigation, formal analysis, data curation, visualization, writing – original draft of the whole text, and writing – review and editing; polymer synthesis, purification, and isolation of **P1**‐**P18**; NMR spectroscopy; SEC (THF); FTIR spectroscopy; TGA; DSC; TEM sample preparation, qNMR analysis for functionalization degree, low‐field ^1^H NMR data treatment, quantitative sensitivity analysis. O.E.E.: investigation, formal analysis, visualization, and writing – original draft (Li–S electrochemistry section); writing – review and editing; design and execution of the electrochemical evaluation of **P11** as an electrolyte additive for Li–S batteries, including cyclic voltammetry, electrochemical impedance spectroscopy, galvanostatic cycling, symmetric‐cell (S‖S, Li‖Li) measurements, and electrolyte‐volume/concentration screening. I.M.: AFM measurements. L.V.: TEM measurements. C.V.: low‐field ^1^H NMR T1 measurements. A.K.: SEC measurements in DMAc. A.S.: supervision (Li–S electrochemistry), formal analysis, and writing – review and editing; contribution to data interpretation and discussion of trends in the Li–S battery study. S.D.: supervision of O.E.E., resources, and writing – review and editing; contribution to the interpretation and discussion of the Li‐S electrochemical data. H.M.: conceptualization, methodology, supervision, project administration, funding acquisition, and writing – review and editing.

## Lead Contact

Further information and requests for resources should be directed to and will be fulfilled by the lead contact, Hatice Mutlu (hatice.mutlu@uni-jena.de).

## Materials Availability

All reagents in this study are commercially available or can easily be synthesized. Full experimental procedures as well as spectra are provided in the supplemental information.

## Conflicts of Interest

The authors declare no conflicts of interest.

## Supporting information




**Supporting File**: advs78468‐sup‐0001‐SuppMat.pdf.

## Data Availability

The data supporting the findings of this study are provided in the manuscript and its Supporting Information. Additional data and code used for the TEM image analysis are available from the corresponding author upon reasonable request.
