# Supplementary material for: Multicomponent Molecular Editing of Polybutadiene: From Design Space to Battery Function
Source: Adv Sci (Weinh). 2026 Nov 3:e78468. Online ahead of print. doi: 10.1002/advs.78468 (PMC13731085; doi:10.1002/advs.78468)
Supplement: Supplementary file 1 [file ADVS-9999-e78468-s001.pdf]

Supporting Information

Multicomponent Molecular Editing of Polybutadiene: From Design Space to Battery Function

Janna Jeschke,[a] Oghenegare Emmanuel Eyankware,[b],[c],[d] Ioannis Moutsios,[e] Loïc Vidal,[a] Cyril Vaulot,[a] Azra Kocaarslan,[f] Angelina Sarapulova,[b],[c],[i],[j] Sonia Dsoke,[b],[c],[d] * Hatice Mutlu*[a],[g],[h],[i],[j],[k]

[a] Institut de Science des Matériaux de Mulhouse (IS2M), UMR 7361 CNRS/Université de Haute Alsace (UHA), 15 rue Jean Starcky, Mulhouse Cedex, 68057 France
Email: hatice.mutlu@uha.fr

[b] Fraunhofer Institute for Solar Energy Systems, Heidenhofstr. 2, 79110 Freiburg, Germany

[c] FMF-Freiburg Materials Research Center, University of Freiburg, Stefan-Meier Str. 21, 79104 Freiburg, Germany

[d] Institute for Sustainable Systems Engineering (INATECH), University of Freiburg, Emmy-Noether-Straße 2, 79110, Freiburg, Germany

[e] Department of Materials Science Engineering, University of Ioannina University Campus-Dourouti, 45110, Ioannina, Greece

[f] Institute of Functional Interfaces (IFG), Karlsruhe Institute of Technology (KIT), Hermann-von-Helmholtz-Platz 1, 76344 Eggenstein-Leopoldshafen, Germany

[g] Prof. Dr. rer.nat. H. Mutlu
Rheinland-Pfälzische Technische Universität Kaiserslautern-Landau (RPTU), Department of Chemistry, Technical Polymer Chemistry, Erwin-Schrödinger-Straße, Gebäude 52–54, 67663 Kaiserslautern, Germany

[h] Prof. Dr. rer.nat. H. Mutlu
Leibniz-Institut für Verbundwerkstoffe GmbH, Erwin-Schrödinger-Straße 58, 67663 Kaiserslautern, Germany

[i] Helmholtz Institute for Polymers in Energy Applications Jena (HIPOLE Jena), Helmholtz-Zentrum Berlin für Materialien und Energie (HZB), Lessingstrasse 12-14, 07743 Jena, Germany

[j] Helmholtz-Zentrum Berlin for Materials and Energy GmbH, Hahn-Meitner-Platz 1, 14109 Berlin, Germany

[k] Friedrich Schiller University Jena, Institute of Organic Chemistry and Macromolecular Chemistry, Jena, Germany
Email: hatice.mutlu@uni-jena.de

Content

A. Experimental procedures
1. Materials
2. General Protocol
3. Calculation of Isolated Yield
4. Calculation of Functionalization Degree (FD)

B. Measurements and analytical methods
1. Nuclear magnetic resonance (NMR) spectroscopy
2. Size exclusion chromatography (SEC)
3. Transmission Fourier-Transform Infrared Spectroscopy (FTIR)
4. Differential Scanning Calorimetry (DSC)
5. Thermogravimetric Analysis (TGA)
6. Atomic force microscopy (AFM)
7. Transmission Electron Microscopy (TEM)
8. Spin–Lattice Relaxation Time (T1) Analysis
9. Preparation of P11-Containing Electrolytes, Cell Assembly, and Electrochemical Testing

C. Additional data, figures and discussions
1. NMR spectra
2. FTIR spectra
3. SEC traces
4. DSC analysis
5. TGA analysis
6. TEM images
7. AFM image
8. T1 analysis
9. Assessment of molecular-input sensitivity
10. PBD Microstructure

D. References

A. **Experimental Procedures**

A.1 **Materials**

All reagents were commercially available and used without further purification unless indicated otherwise. For all spectroscopic studies, spectroscopic grade solvents were used as purchased unless otherwise noted.

The polybutadiene (PBD, LBR-307-B, $M_{n,\,SEC}$ = 8900 g mol^{-1}, $Đ$ = 1.06) employed in this work was kindly supplied by Kuraray Kashima Plant and used without further purification. Tetrahydrofuran (THF, Acros Organics, 99.5%, extra dry, over molecular sieves), 2-methyltetrahydrofuran (2-MeTHF, anhydrous, ≥99%, Inhibitor-free), tetrahydropyran (THP, anhydrous, 99%), methanol (MeOH, VWR, normapur), *N*-bromosuccinimide (NBS, Merck, ≥ 99.0 %), 4-Methylbenzene-1-sulfonamide (*p*$TsNH_2$, *para*-toluenesulfonamide, Merck, ≥99%), methanesulfonamide ($MeSO_2NH_2$, Merck, ≥99%), 4-nitrobenzenesulfonamide (*p*$NsNH_2$, Merck, 97%), 4-(trifluoromethyl)benzenesulfonamide (*p*-CF_3BsNH_2, TCI, >98.0%), methanesulfonic acid (MSA, Merck, ≥99.0%), *para*-toluenesulfonic acid (*p*-TsOH, Sigma Aldrich, 97%), chloroform ($CHCl_3$, ≥99.8%, contains 0.5-1.0% ethanol as stabilizer), dichloromethane (DCM, VWR, 99.8 %, stabilized with 0.2 % of ethanol), 1,4-dimethoxybenzene (TCI, >99.0%), chloroform-d1 ($CDCl_3$, Sigma Aldrich, 99.8% D), tetrahydrofuran (THF-d8, eurisotop, 99.8% D) were used as received.

A.2 **General Multicomponent Post-Polymerization Modification Protocol and Polymer Structures**

P1-**P3** were synthesized according to our previously reported procedure.[1] The same procedure was applied to the synthesis of **P4**-**P18**. Accordingly, **PBD** ($M_{n,SEC}$ = 8,900 g mol^{-1}, $Đ$ = 1.06; 400 mg, 7.40 mmol repeating units, 1.00 equiv.) and the corresponding sulfonamide or sulfonic acid nucleophile (7.40 mmol, 1.00 equiv.) were dissolved in the respective dry cyclic ether, namely THF, 2-MeTHF, or THP (494 mmol, 67.0 equiv.), and the reaction mixture was cooled to 0 °C using an ice bath. The reaction conditions for **P1**-**P6**, **P7**-**P12**, and **P13**-**P18** are summarized in **Schemes S1**, **S2**, and **S3**, respectively.

Subsequently, NBS (5.27 g, 29.6 mmol, 4.00 equiv.) was added, and the reaction mixture was stirred for 4 h while gradually warming to ambient temperature. The resulting polymer was precipitated into ice-cold methanol and purified by two additional precipitations cycles from THF into methanol. The proposed structures and isolated yields of the THF-derived polymers **P4**-**P6** are shown in **Scheme S4**, those of the 2-MeTHF-derived polymers **P7**-**P12** in **Scheme S5**, and those of the THP-derived polymers **P13**-**P18** in **Scheme S6,** respectively.

The calculation of the isolated yields is detailed in Section **A.3**.

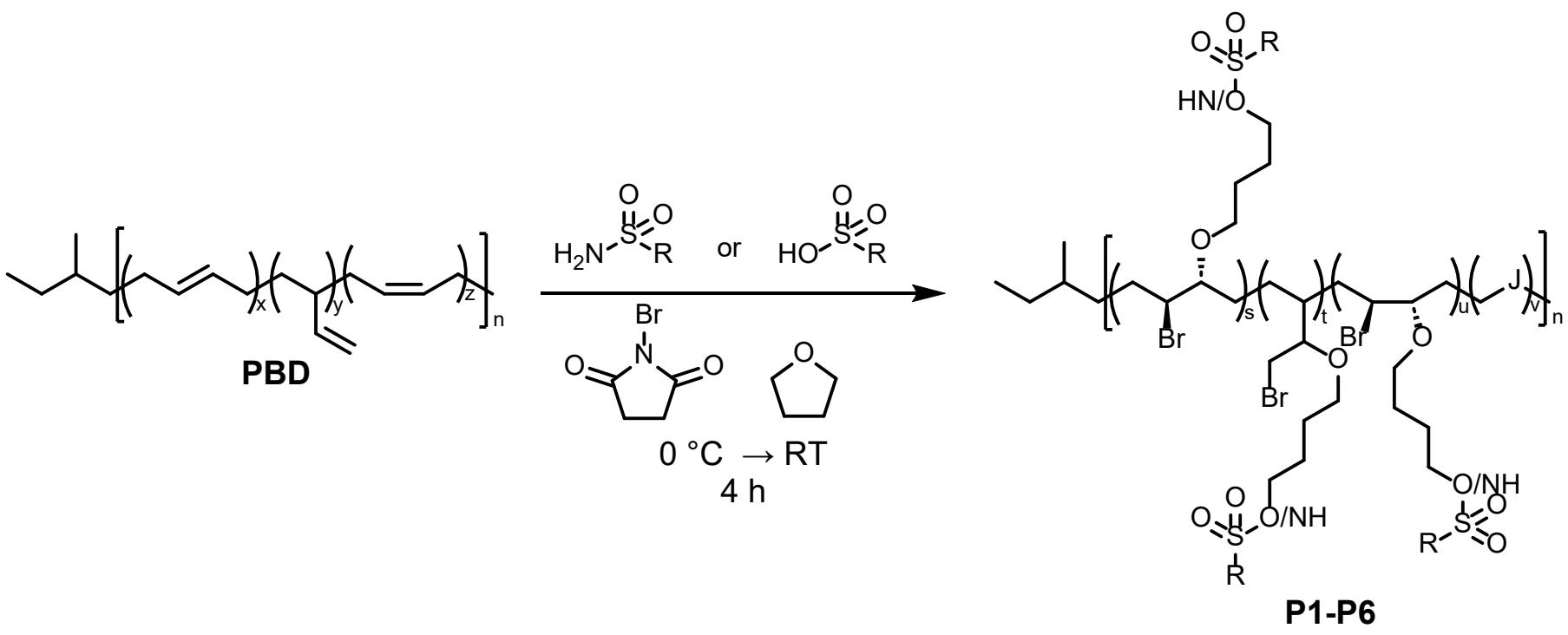

Scheme S1. Synthesis of THF-derived polymers **P1**-**P6** via NBS-mediated multicomponent post-polymerization modification of **PBD** in the presence of the corresponding sulfonamide or sulfonic acid nucleophile.

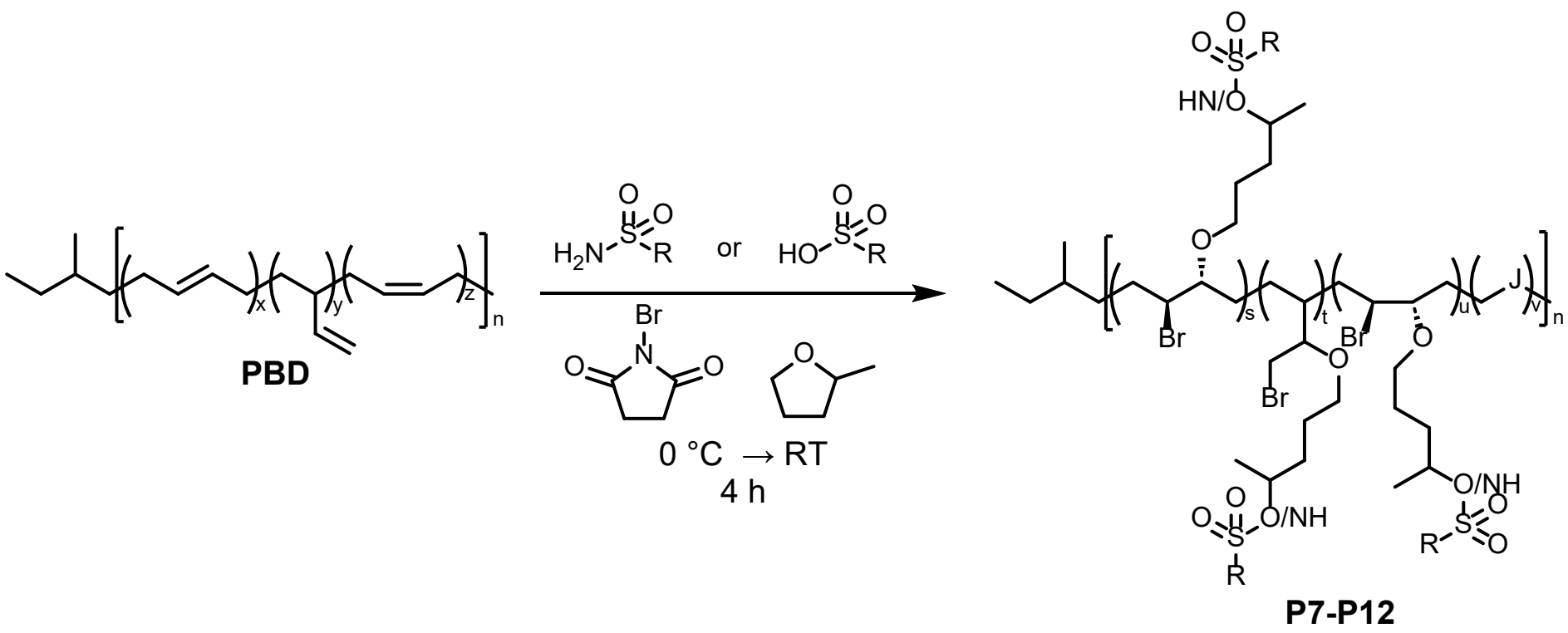

Scheme S2. Synthesis of 2-MeTHF-derived polymers **P7**-**P12** via NBS-mediated multicomponent post-polymerization modification of **PBD** in the presence of the corresponding sulfonamide or sulfonic acid nucleophile.

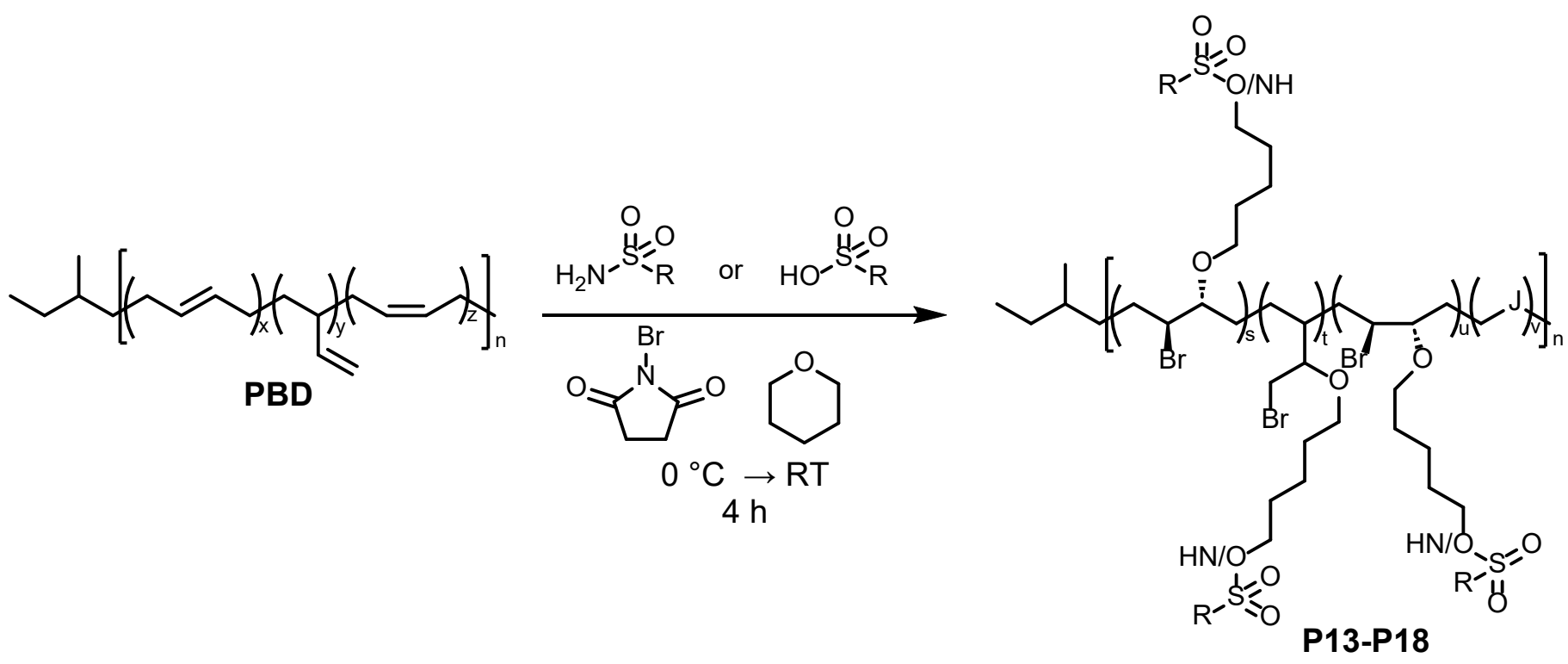

Scheme S3. Synthesis of THP-derived polymers **P13**-**P18** via NBS-mediated multicomponent post-polymerization modification of **PBD** in the presence of the corresponding sulfonamide or sulfonic acid nucleophile.

The polymer structures depicted in Schemes **S4-S6** are schematic representations of the experimentally supported average compositions. They illustrate representative repeating-unit motifs and do not imply selective derivatization of 1,2- versus 1,4-derived PBD units, a defined sequence, spacing, or relative positioning of the incorporated functionalities, or a defined stereochemical configuration at stereogenic centers generated during functionalization.

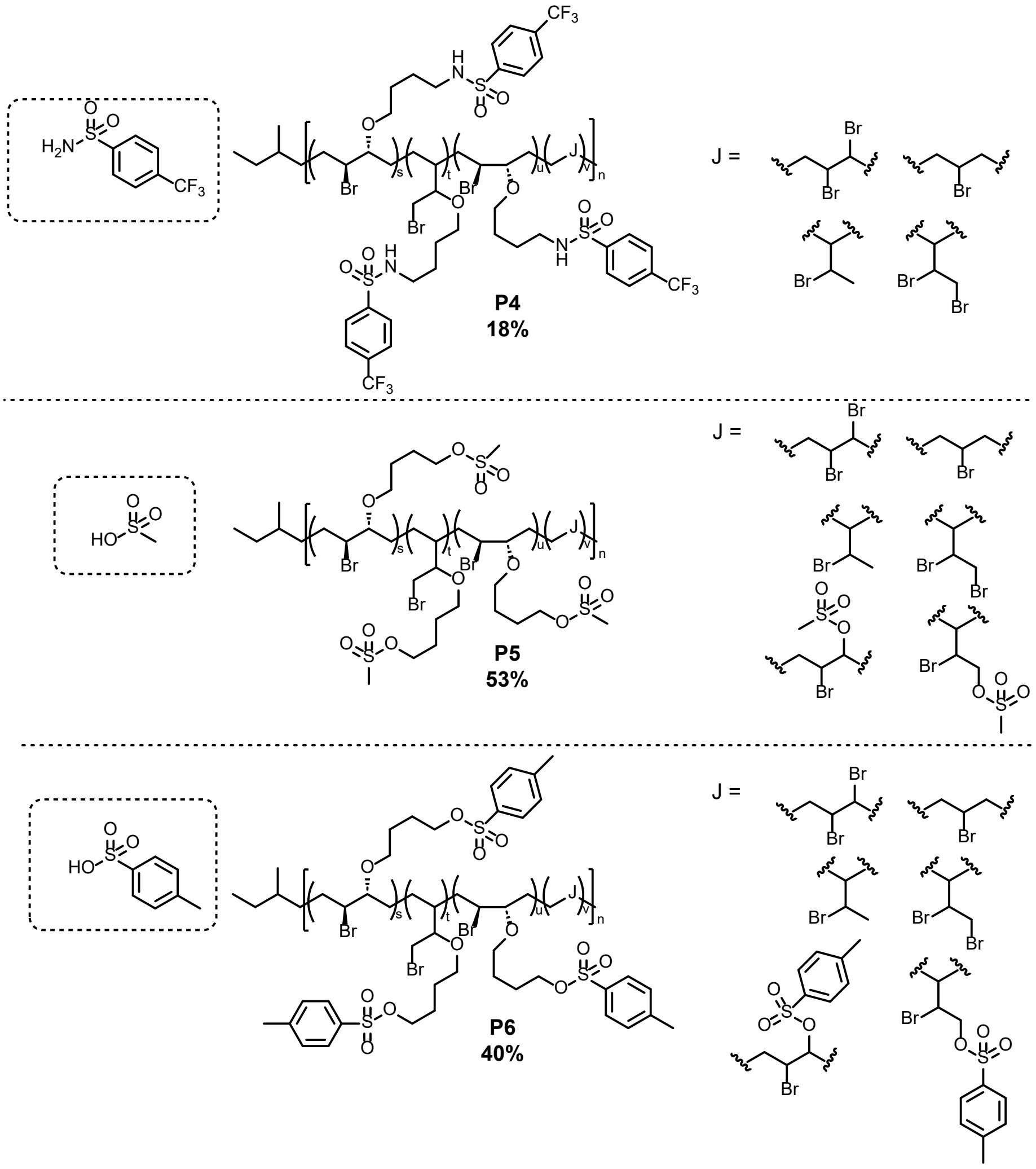

Scheme S4. Proposed structures and isolated yields of the THF-derived polymers **P4**-**P6** obtained by NBS-mediated multicomponent post-polymerization modification of **PBD**. The corresponding nucleophiles were 4-(trifluoromethyl)benzenesulfonamide for **P4**, methanesulfonic acid for **P5**, and *p*-toluenesulfonic acid for **P6**, affording the respective polymers in isolated yields of 18%, 53%, and 40%. The repeating-unit motifs labeled J denotes representative structural repeating-unit motifs resulting from residual bromination and/or nucleophile incorporation. Their formation is consistent with the quantitative double bond conversion of **PBD**, the use of excess NBS, and, for sulfonic acid nucleophiles. For sulfonic acid nucleophiles, both cyclic ether-assisted and direct bromonium-ion opening pathways may contribute to the observed product distribution.

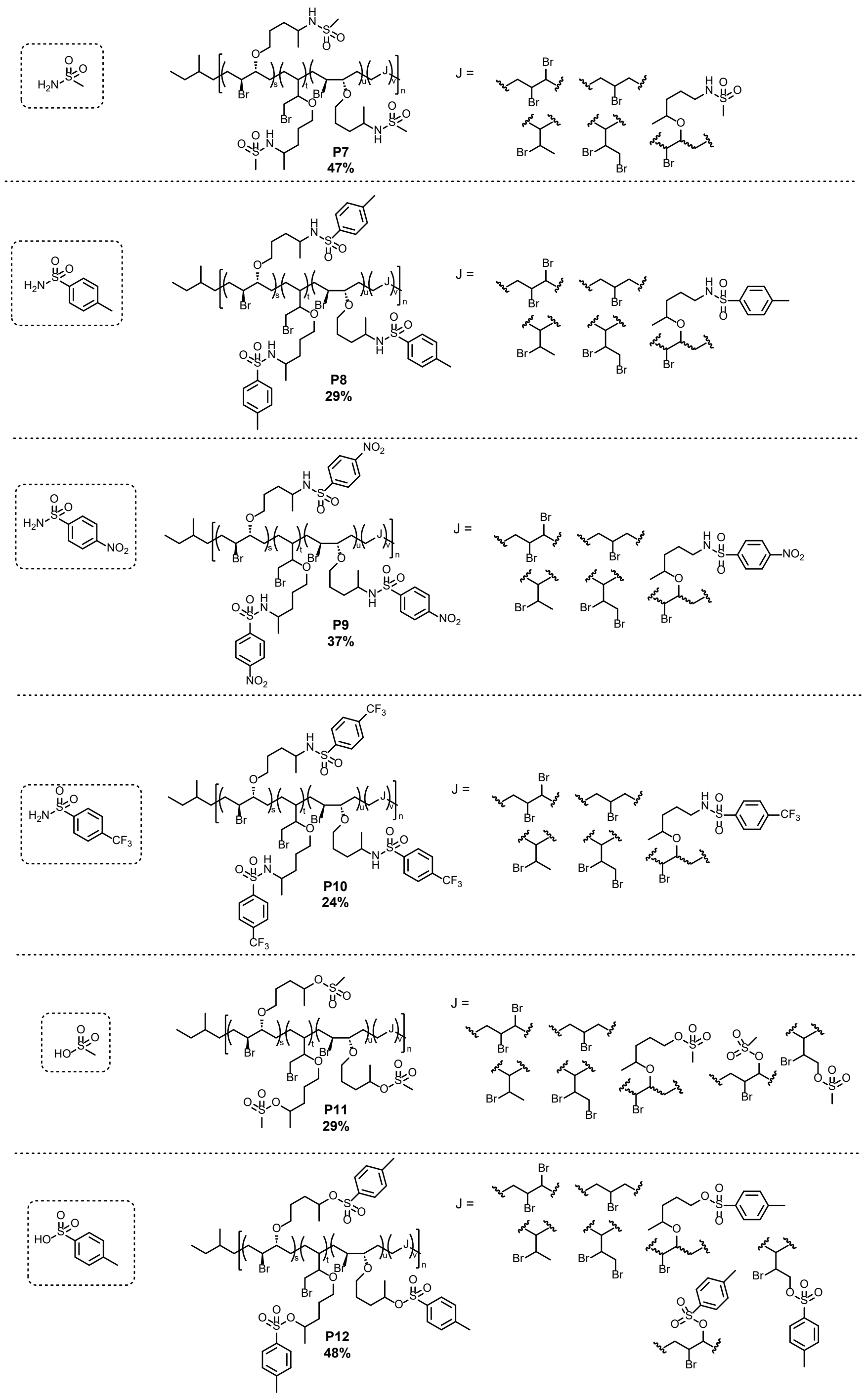

Scheme S5. Proposed structures and isolated yields of the 2-MeTHF-derived polymers **P7**-**P12** obtained by NBS-mediated multicomponent post-polymerization modification of **PBD**. The corresponding nucleophiles were methanesulfonamide for **P7**, *p*-toluenesulfonamide for **P8**, 4-nitrobenzenesulfonamide for **P9**, 4-(trifluoromethyl)benzenesulfonamide for **P10**, methanesulfonic acid for **P11**, and *p*-toluenesulfonic acid for **P12**, affording the respective polymers in isolated yields of 47%, 28%, 37%, 24%, 29%, and 48%. The repeating-unit motifs labeled J denotes representative alternative repeating-unit motifs resulting from residual bromination and/or nucleophile incorporation. Their formation is consistent with the quantitative double bond conversion of **PBD**, the use of excess NBS, and, for sulfonic acid nucleophiles. For the sulfonic acid-derived polymers, both 2-MeTHF-assisted and direct bromonium-ion opening pathways may contribute to the proposed product distribution.

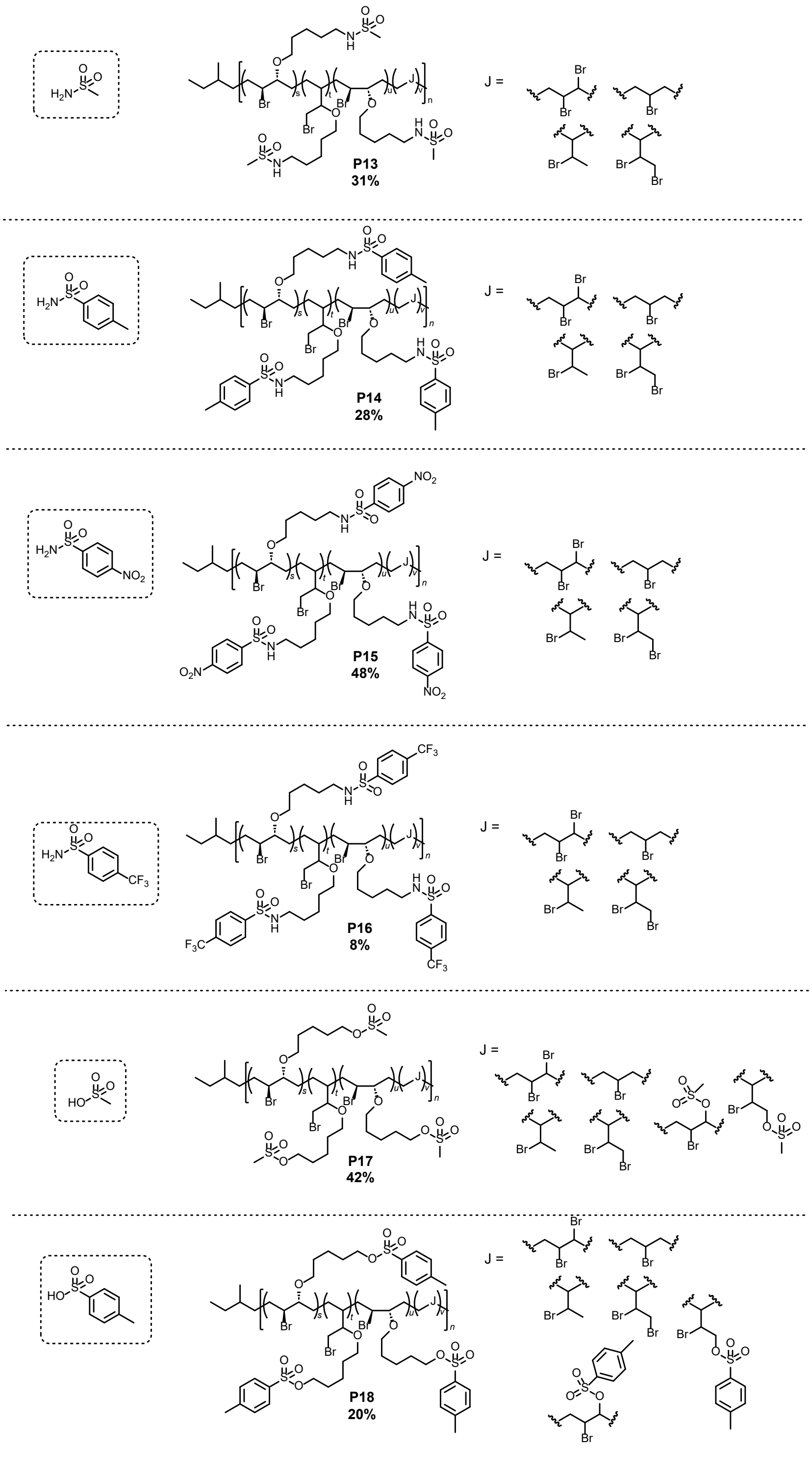

Scheme S6. Proposed structures and isolated yields of the THP-derived polymers **P13**-**P18** obtained by NBS-mediated multicomponent post-polymerization modification of **PBD**. The corresponding nucleophiles were methanesulfonamide for **P13**, *p*-toluenesulfonamide for **P14**, 4-nitrobenzenesulfonamide for **P15**, 4-(trifluoromethyl)benzenesulfonamide for **P16**, methanesulfonic acid for **P17**, and *p*-toluenesulfonic acid for **P18**, affording the respective polymers in isolated yields of 31%, 28%, 48%, 8%, 42%, and 20%. The repeating-unit motifs labeled J denotes representative alternative repeating-unit structures arising from residual bromination and/or nucleophile incorporation. Their formation is consistent with the quantitative double bond conversion of **PBD**, the use of excess NBS, and, for sulfonic acid nucleophiles. For the sulfonic acid-derived polymers, both THP-assisted and direct bromonium-ion opening pathways may contribute to the proposed product distribution.

A.3 **Calculation of Isolated Yield**

Isolated yields were calculated based on the theoretical mass of the idealized, fully edited (i.e., functionalized) polymer product, assuming incorporation of one bromine atom, one cyclic ether unit, and one sulfonamide or sulfonate ester moiety into every PBD repeat unit according to the idealized reaction stoichiometry. Accordingly, the theoretical product mass ($m_{theor.}$) was determined as the sum of (i) the initial mass of **PBD** (m_{PBD}), (ii) the theoretical mass contribution of the incorporated sulfonamide or sulfonate ester moiety, accounting for the loss of one hydrogen atom ($m_{0,sulfonamide/sulfonic\ ester}$), (iii) the theoretical mass contribution of one bromine atom incorporated per **PBD** repeating unit ($m_{0,Br}$), and (iv) the theoretical mass contribution of one tetrahydrofuran (THF), 2-methyl tetrahydrofuran (2-MeTHF) or tetrahydropyran (THP) unit incorporated per **PBD** repeating unit ($m_{0,THF}$):

$$m_{theor.} = m_{oPBD} + m_{0,sulfonamide/sulfonic\ ester} + m_{0,Br} + m_{0,ether}$$

The number of **PBD** repeating units $n_{0,PBD}$ were calculated from the initial mass of PBD (0.400 g) using a repeat-unit molecular weight of 54 g mol^{-1}:

$$n_{0,PBD} = \frac{0{,}400}{54} = 7.407 \times 10^{-3}\ moles$$

Isolated yields were then calculated according to:

$$Isolated\ Yield\ (\%) = \left(\frac{m_{isolated}}{m_{theor.}}\right) \times 100$$

Because the theoretical product mass is based on the idealized fully edited polymer, the isolated yield should be regarded as a measure of polymer recovery after purification rather than the functionalization efficiency of the multicomponent editing reaction. The extent of functionalization was determined independently by quantitative ^{1}H NMR spectroscopy and is discussed in the main text.

For example, for P9:

The theoretical product mass ($m_{theor.}$) was calculated according to:

$$m_{theor.\ P9} = 0.400 + \left(\left(\frac{0.400}{54}\right) \times 201.184\right) + \left(\left(\frac{0.400}{54}\right) \times 79.904\right) + \left(\left(\frac{0.400}{54}\right) \times 86.134\right) = 3.120\ \text{g}$$

Using an isolated mass ($m_{isolated}$) of 1.1535 g, an isolated yield of 37% was obtained:

$$\boldsymbol{Isolated\ Yield\ P9}\ (\%) = \left(\frac{1.1535}{3.120}\right) \times 100 \approx \mathbf{37}\%$$

A.4 **Estimation of the Average Functionalization Degree (FD)**

Quantitative ^1H NMR measurements were performed in $CDCl_3$ or DMSO-d_6 at 25 °C on a 300 MHz spectrometer using 1,4-dimethoxybenzene as an internal standard (ISTD). Spectra were acquired with a relaxation delay of 25 s, a 45° excitation pulse (6.4 μs; calibrated 90° pulse = 12.8 μs), and 32 scans. These conditions were selected to minimize differential saturation and enable reliable quantitative integration of the analyte and internal-standard signals. The reported FD represents an ensemble-average incorporation level across the analyzed polymer sample. The measurement does not resolve functionalization separately at 1,2- and 1,4-derived PBD units, nor does it provide the distribution, sequence, or relative spacing of functional groups along individual polymer chains.

Exemplary calculation for P4: For quantitative evaluation, an NMR response factor, (k_{NMR}), was calculated from the known amount and integral of the internal standard:

$$k_{\mathrm{NMR}} = \frac{I_{\mathrm{ISTD}}}{n_{\mathrm{ISTD}} \times N_{\mathrm{ISTD}}}$$

where I_{ISTD} corresponds to the integral of ISTD signal, n_{ISTD} the moles of internal standard, and N_{ISTD} the number of protons corresponding to the selected resonance.

For P4:

$$k_{\mathrm{NMR}} = \frac{4}{1.6140 \times 10^{-5} \times 4}$$

The moles of incorporated sulfonamide were then calculated according to:

$$n_{\mathrm{sulfonamide}} = \frac{I_{\mathrm{sulfonamide}}}{k_{\mathrm{NMR}} \times N_{\mathrm{sulfonamide}}}$$

Thus, for P4, $n_{sulfonamide}$ was calculated as follows:

$$n_{sulfonamide} = \frac{2.26}{61959.64 \times 2} = 1.82377 \times 10^{-5}\, mol$$

The analyzed total qNMR polymer sample had a mass (m_{sample}) of:

$$m_{sample} = 0.01301\, g$$

The molecular weight of one sulfonamide-functionalized repeating unit ($M_{sulf\text{-}RU}$) was calculated according to:

$$M_{sulf-RU} = M_0 + (M_{sulfonamide} - 1) + M_{Br} + M_{THF}$$

Where M_0 is the molecular weight of one **PBD** repeating unit, $M_{sulfonamide}$ is the molecular weight of 4-trifluoromethylbenzenesulfonamide, M_{Br} is the molecular weight of one bromine atom, and M_{THF} is the molecular weight of the cyclic ether spacer (THF in the case of P4):

$$M_{sulf-RU} = 54 + (225{,}19 - 1) + 79.90 + 72.11 = 430.2\, g\, mol^{-1}$$

The mass corresponding to the sulfonamide-functionalized repeat units was calculated as:

$$m_{sulf-RU} = n_{sulfonamide} \times M_{sulf-RU}$$

$$m_{sulf-RU} = 1.82377 \times 10^{-5} \times 430.2 = 0.0078\ g$$

The remaining polymer mass was therefore:

$$m_{remaining} = m_{sample} - m_{sulf-RU} = 0.01301 - 0.0078 = 0.0052\ g$$

Consistent with the quantitative consumption of the PBD double bonds observed by NMR spectroscopy and the use of excess NBS during multicomponent editing reaction, the remaining repeating units were assumed to be monobrominate, thus their molecular weight was calculated as:

$$M_{monoBr-RU} = M_0 + M_{Br} = 54 + 79{,}90 = 133.905\ g\ mol^{-1}$$

Hence, the moles of monobrominated repeating units were calculated accordingly:

$$n_{monoBr-RU} = \frac{m_{remaining}}{M_{monoBr-RU}}$$

$$n_{monoBr-RU} = \frac{0.0052}{133.905} = 3.86 \times 10^{-5} mol$$

The total amount of repeating units in the analyzed sample was then obtained as:

$$n_{total\ RU} = n_{sulfonamide} + n_{monoBr-RU}$$

$$n_{total\ RU} = 1.82377 \times 10^{-5} + 3.86 \times 10^{-5} = 5.6803\ 10^{-5}\ mol$$

Finally, the sulfonamide FD (mol%) was calculated according to:

$$FD, P4\ (mol\%) = \frac{n_{sulfonamide}}{n_{total\ RU}} \times 100 = \frac{1.82377 \times 10^{-5}}{5.6803\ 10^{-5}} \times 100 = \mathbf{32\ mol}\%$$

Accordingly, the resulting FD values are interpreted as ensemble-average estimates of the incorporated sulfonamide or sulfonate ester functionality rather than exact, site-resolved compositions of the polymer chains.

Estimation of model-derived polymer-bound bromine content

The polymer-bound bromine content of **P1-P18** was estimated using the same average-composition model employed for the qNMR-derived functionalization degree. Within this model, one polymer-bound Br atom is assigned per original PBD repeating unit. The qNMR-derived average FD, f_{FD}, defines the fraction of repeating units additionally bearing the corresponding cyclic-ether/nucleophile-derived functionality, whereas the remaining repeating units are treated as monobrominated, as described above. The average model-derived repeating-unit molar mass ($\overline{M}_{RU}$) was therefore calculated according to

$$\overline{M}_{RU} = M_{0,RU\ PBD} + M_{Br} + (f_{FD} \times (M_{cyclic\ ether} + (M_{nucleophile} - (M_H)))$$

The model-derived bromine content was subsequently calculated as

$$w_{Br} = \frac{M_{Br}}{\overline{M}_{RU}} \times 100$$

Thus, for **P4**:

$$\overline{M}_{RU,P4} = 54 + 79.904 + \left(0.3211 \times \left(72.11 + (225.19 - 1.008)\right)\right) \approx 229.0\ g\ mol^{-1}$$

$$w_{Br,P4} = \frac{79.904}{229.0} \times 100 \approx 35\%$$

The resulting values represent model-derived average polymer-bound bromine contents and should not be interpreted as independent elemental determinations of bromine.

Table S1. Quantitative ^{1}H NMR input values used to estimate the average functionalization degree (FD) of P1-P18 using 1,4-dimethoxybenzene as internal standard (ISTD).

Entry	Sample	$N_{\text{sulfonamide/sulfonate ester}}$	$I_{\text{sulfonamide/sulfonate ester}}$	n_{ISTD} / µmol	k_{NMR}	FD /mol%
1	P1	3	1.37	24.68	40519.06	20
2	P2	2	1.81	18.53	53972.66	30
3	P3	2	1.14	43.06	23221.85	34
4	P4	2	2.26	16.14	61959.64	32
5	P5	3	2.06	21.35	46837.29	25
6	P6	2	2.24	16.07	62238.74	38
7	P7	3	1.21	20.48	48823.32	11
8	P8	2	0.96	21.00	47644.83	17
9	P9	2	1.41	35.32	28313.52	34
10	P10	2	0.81	16.43	60867.84	22
11	P11	3	3.8	15.13	66110.05	40
12	P12	2	1.85	37.13	26933.72	59
13	P13	3	2.06	19.54	51174.07	17
14	P14	2	0.93	22.07	45301.64	30
15	P15	2	1.64	18.17	55047.81	39
16	P16	2	0.75	23.30	42909.94	36
17	P17	3	4.49	25.84	38703.08	58
18	P18	2	3.29	15.13	66110.05	68

$N_{\text{sulfonamide/sulfonate ester}}$ and $I_{\text{sulfonamide/sulfonate ester}}$ denote the number of protons and integral, respectively, of the diagnostic resonance of the incorporated sulfonamide or sulfonate ester. The integral of the 1,4-dimethoxybenzene ISTD resonance at 6.83 ppm was normalized to 4.00, corresponding to 4H. FD values represent ensemble-average incorporation estimates.

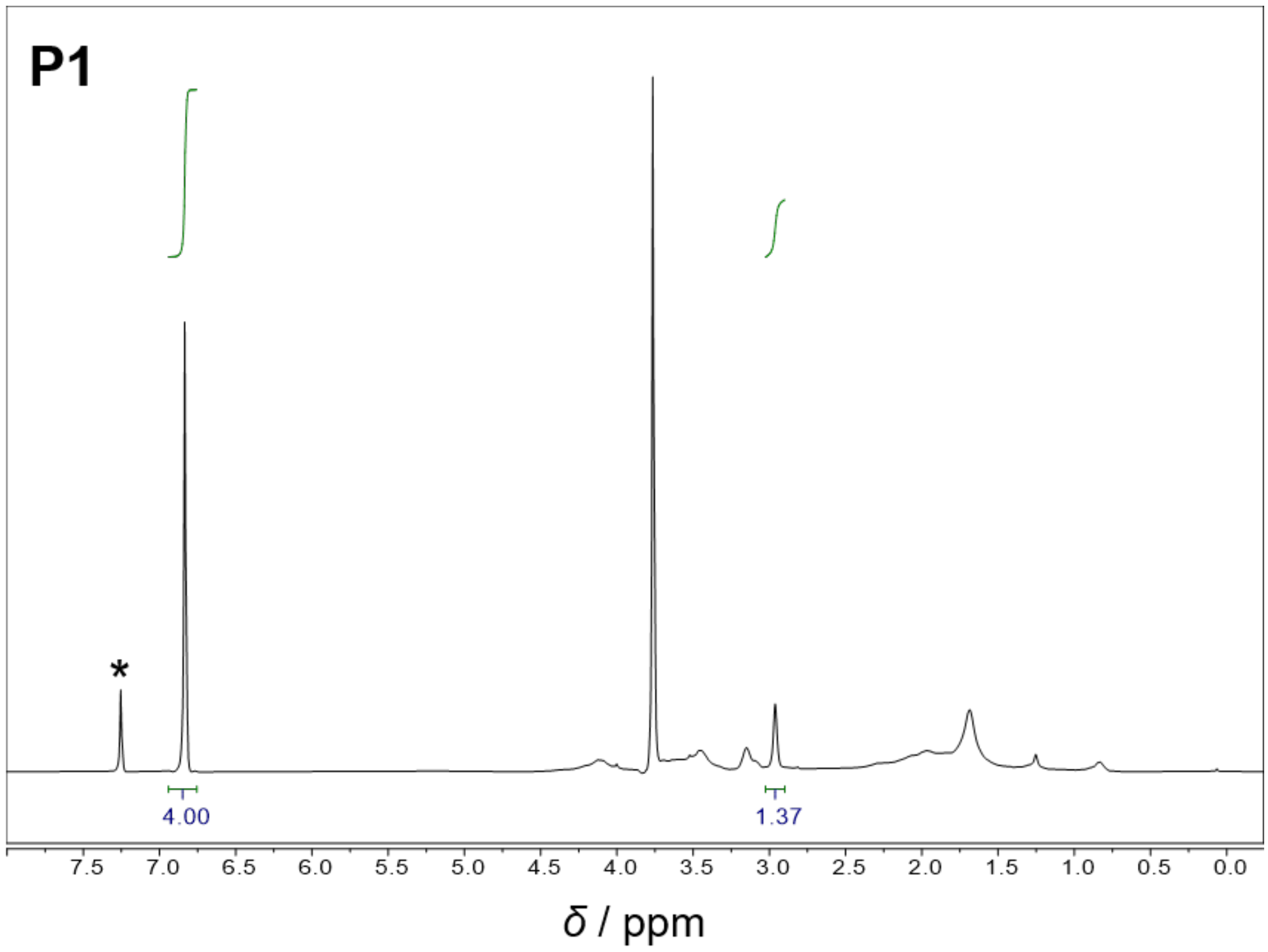

Figure S1. Quantitative ^{1}H NMR spectrum of **P1** (300 MHz, $CDCl_3$, 25 °C) using 1,4-dimethoxybenzene as an internal standard (ISTD). *residual $CHCl_3$.

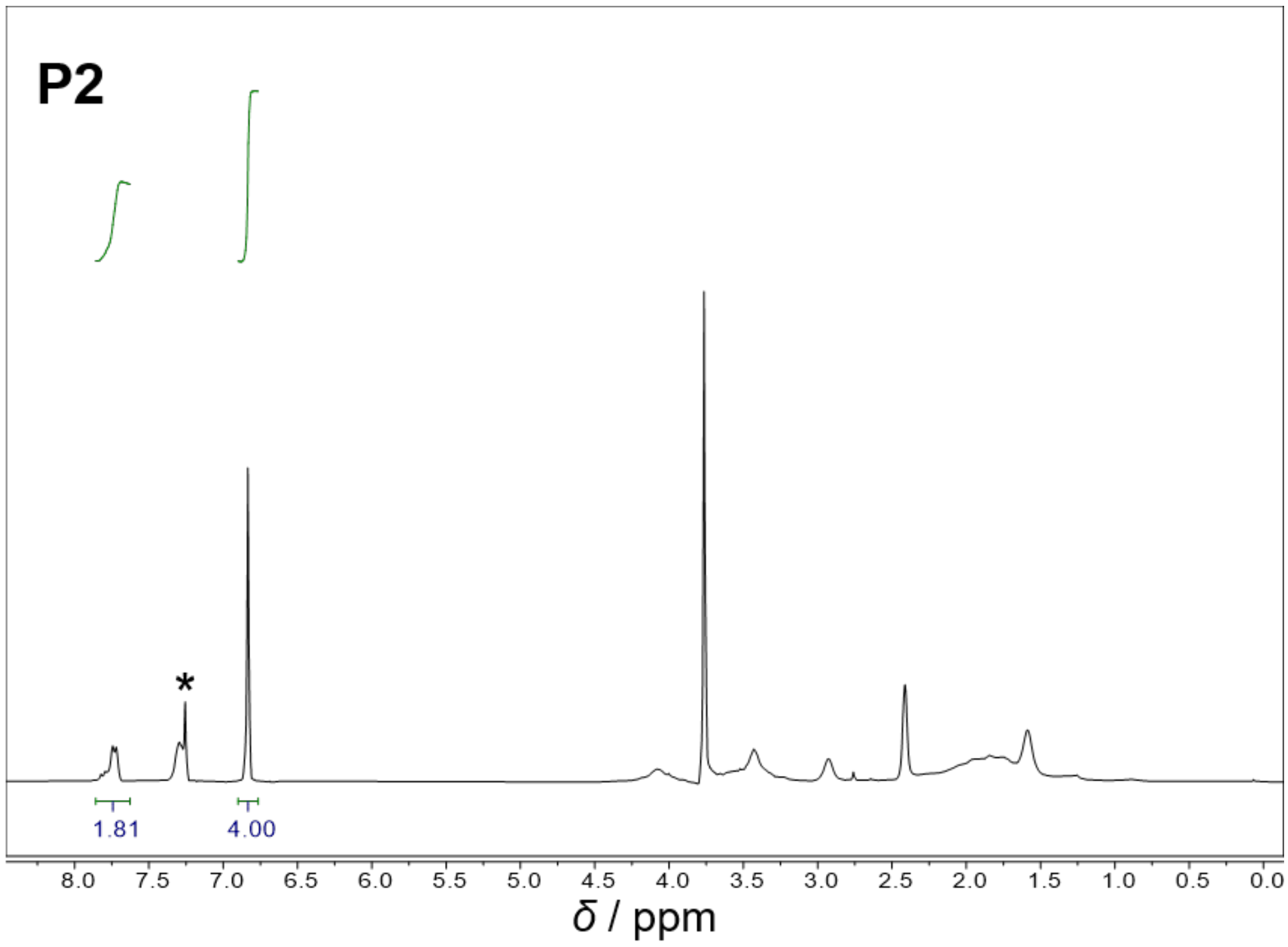

Figure S2. Quantitative ^{1}H NMR spectrum of **P2** (300 MHz, $CDCl_3$, 25 °C) using 1,4-dimethoxybenzene as an internal standard (ISTD). *residual $CHCl_3$.

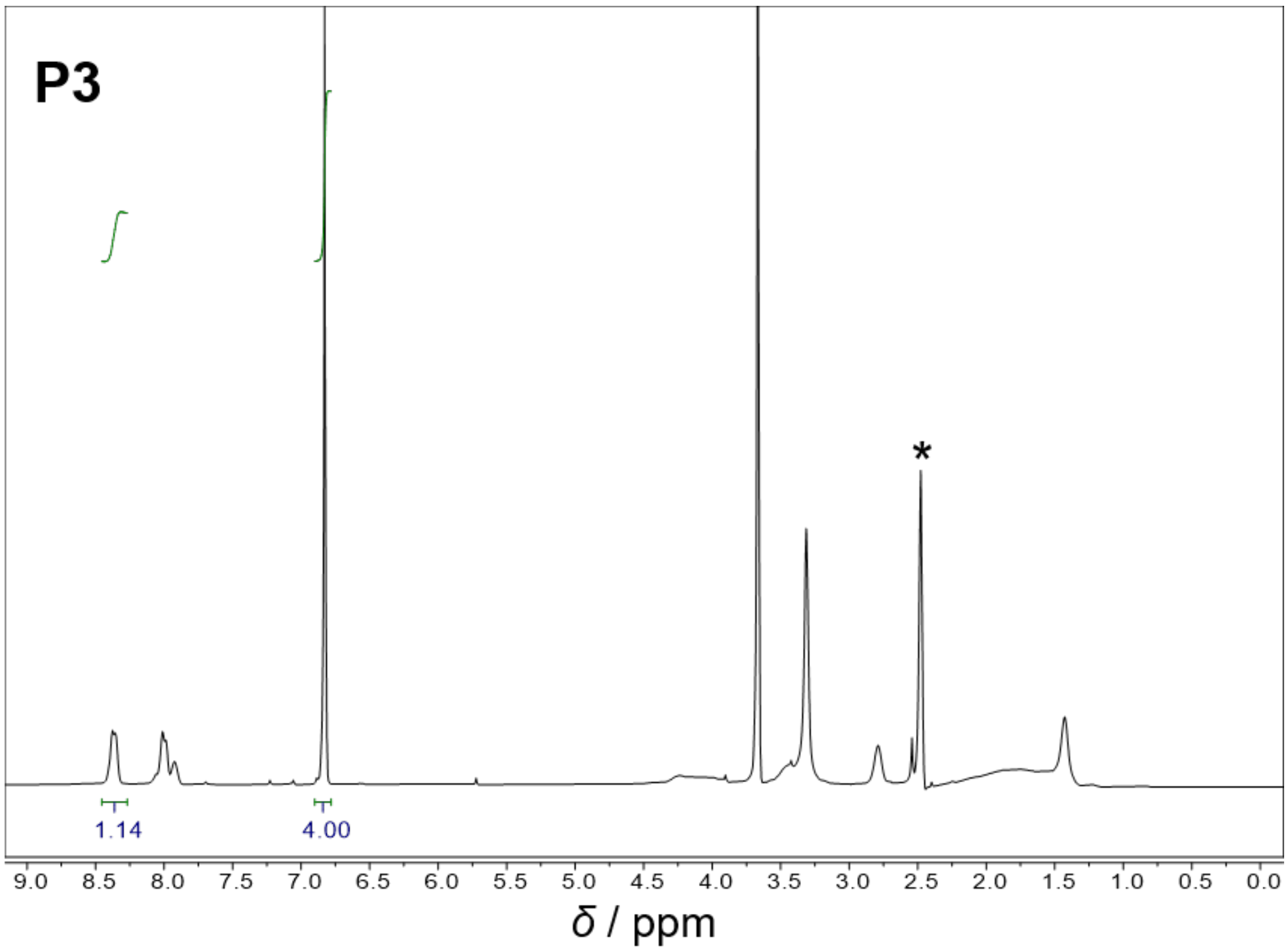

Figure S3. Quantitative ^{1}H NMR spectrum of **P3** (300 MHz, DMSO-d_6, 25 °C) using 1,4-dimethoxybenzene as an internal standard (ISTD). *residual DMSO.

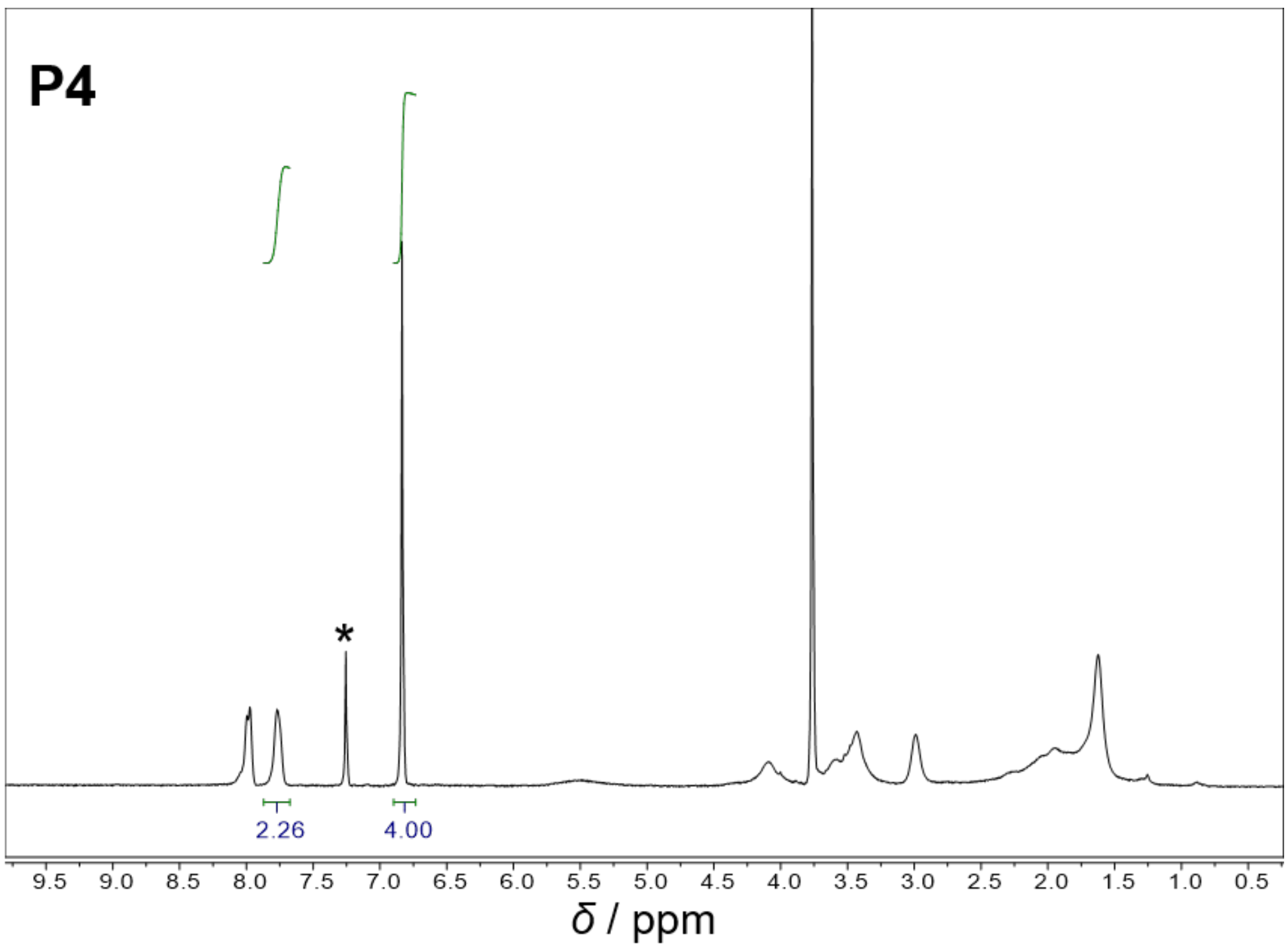

Figure S4. Quantitative ^{1}H NMR spectrum of **P4** (300 MHz, $CDCl_3$, 25 °C) using 1,4-dimethoxybenzene as an internal standard (ISTD). *residual $CHCl_3$.

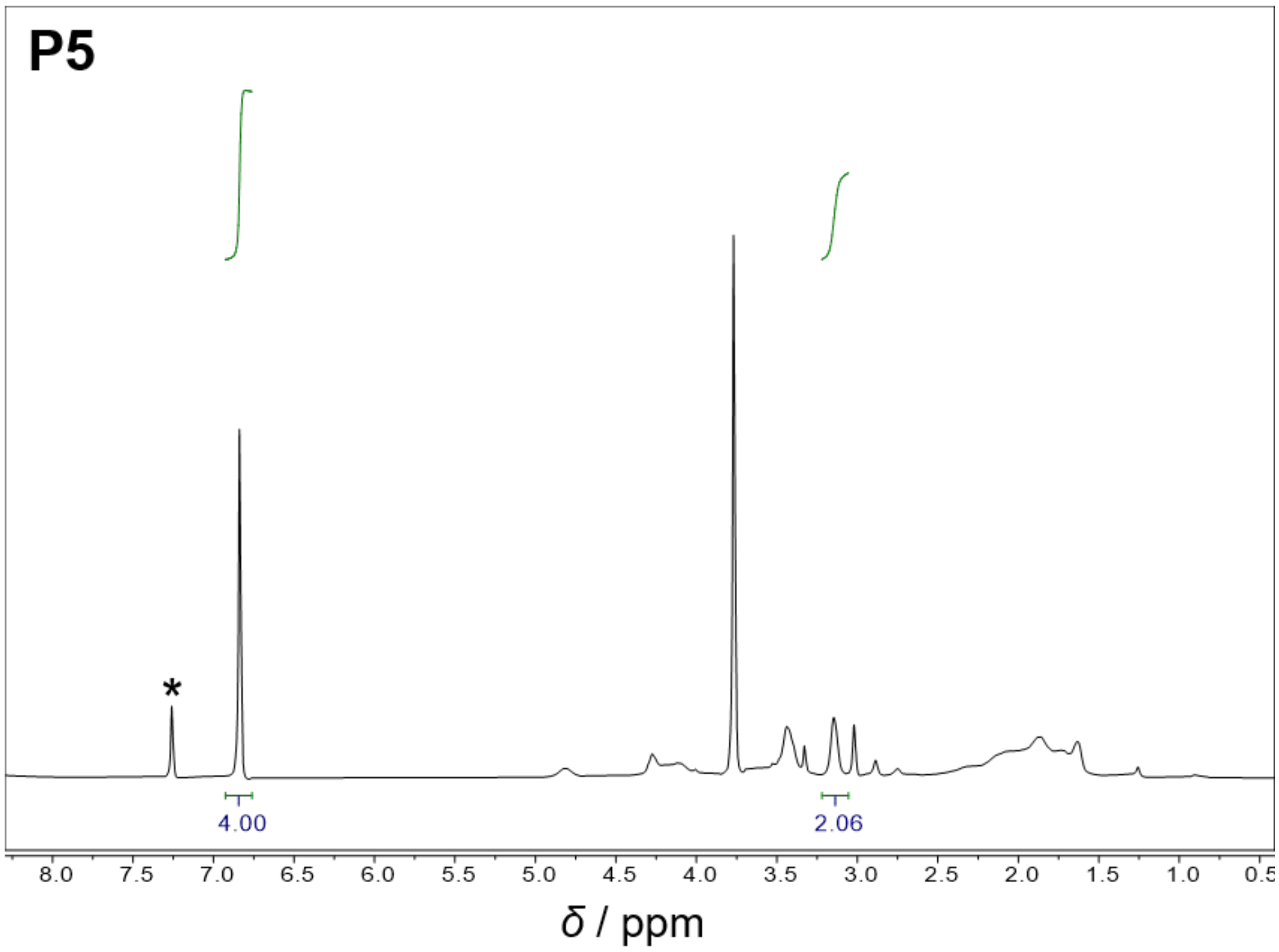

Figure S5. Quantitative ^{1}H NMR spectrum of **P5** (300 MHz, $CDCl_3$, 25 °C) using 1,4-dimethoxybenzene as an internal standard (ISTD). *residual $CHCl_3$.

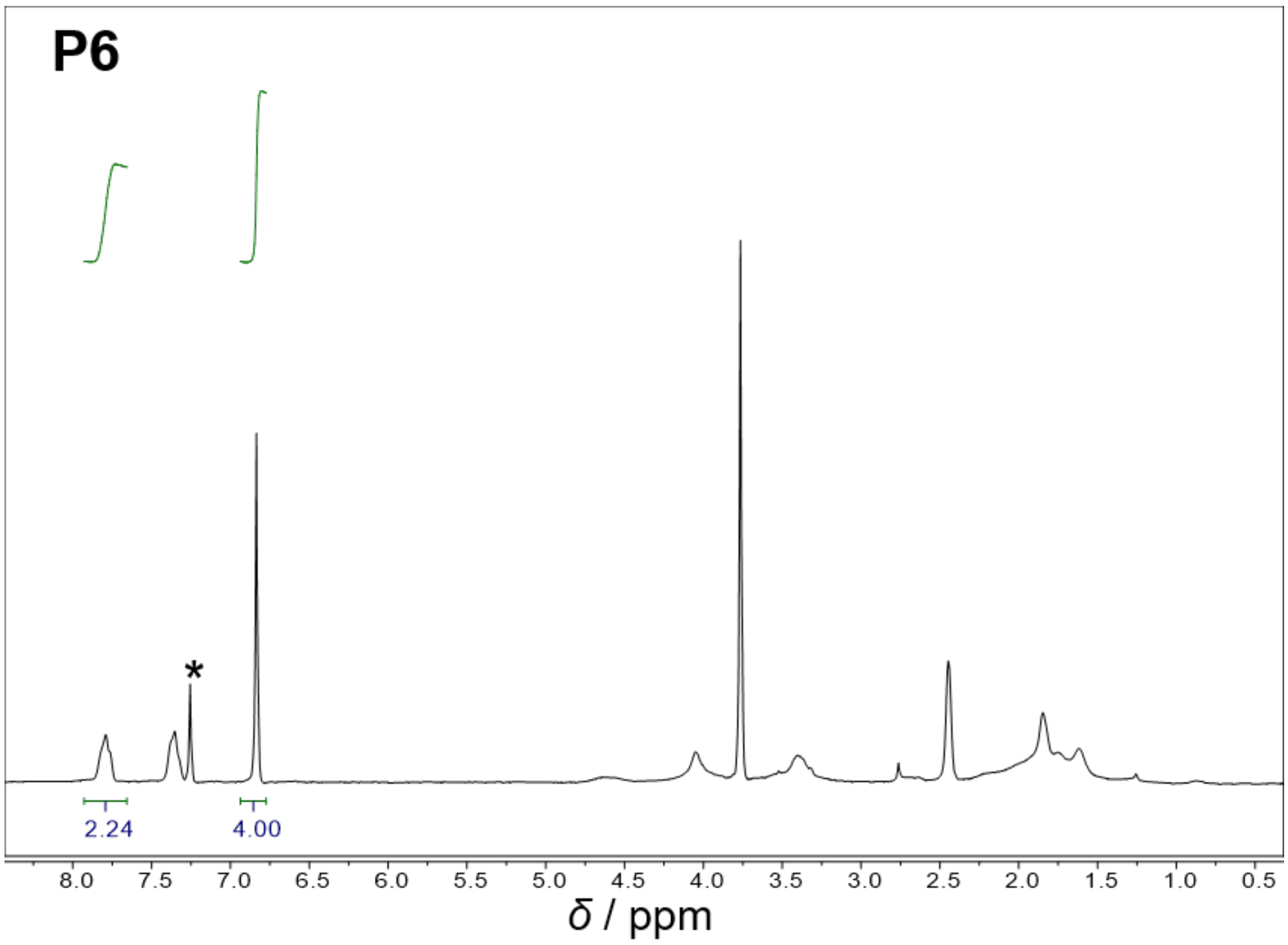

Figure S6. Quantitative ^{1}H NMR spectrum of **P6** (300 MHz, $CDCl_3$, 25 °C) using 1,4-dimethoxybenzene as an internal standard (ISTD). *residual $CHCl_3$.

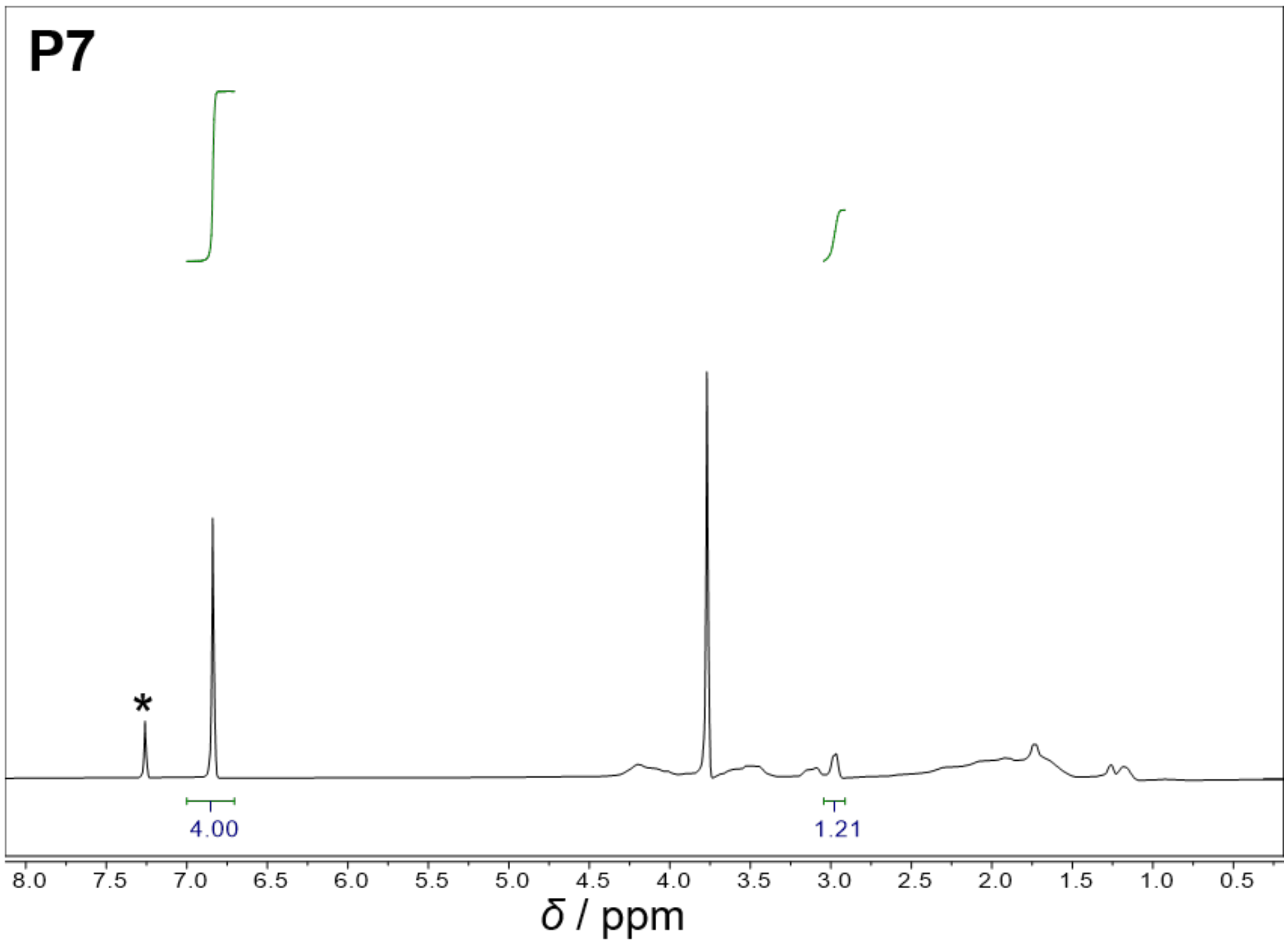

Figure S7. Quantitative ¹H NMR spectrum of **P7** (300 MHz, $CDCl_3$, 25 °C) using 1,4-dimethoxybenzene as an internal standard (ISTD). *residual $CHCl_3$.

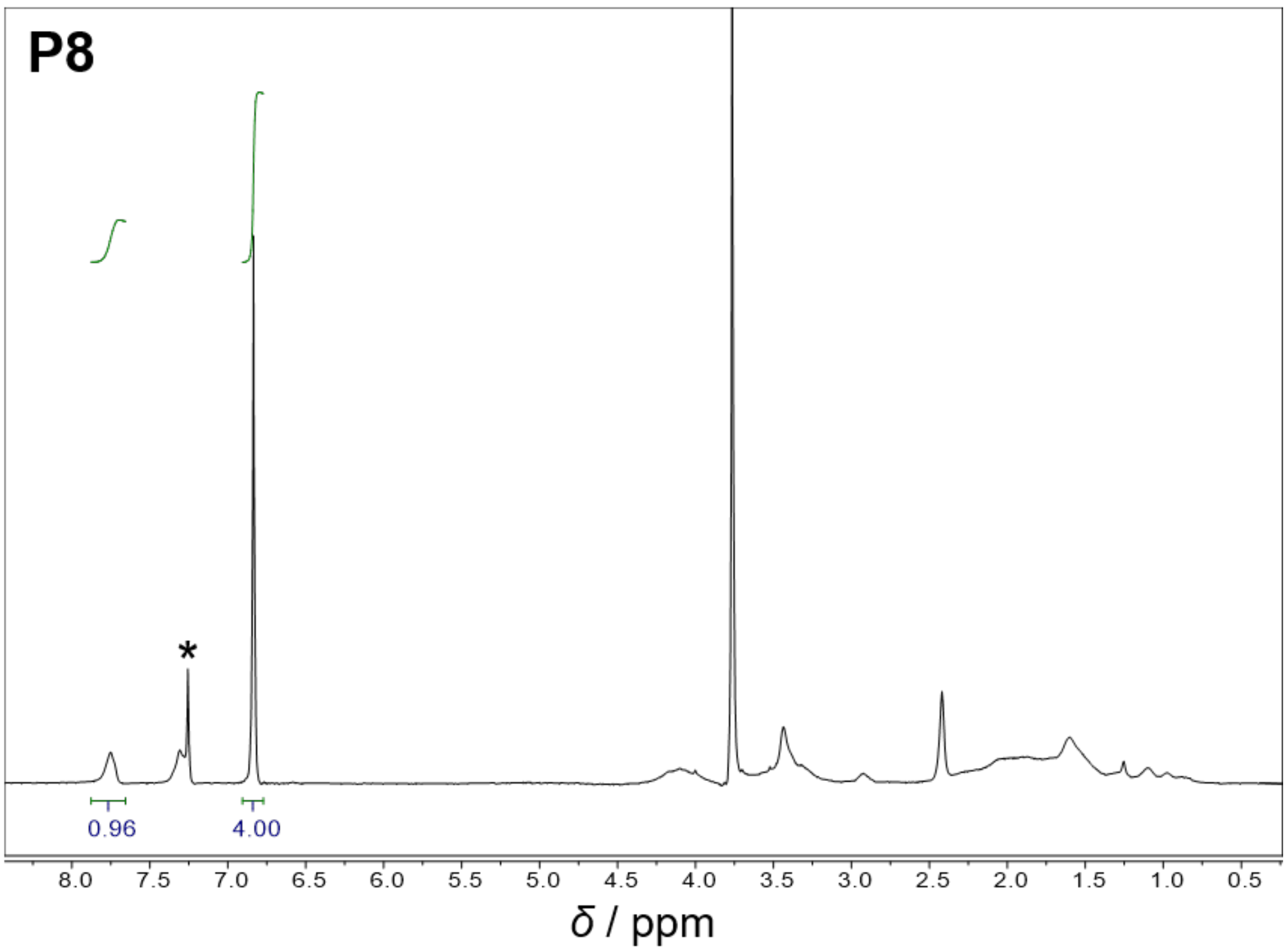

Figure S8. Quantitative ¹H NMR spectrum of **P8** (300 MHz, $CDCl_3$, 25 °C) using 1,4-dimethoxybenzene as an internal standard (ISTD). *residual $CHCl_3$.

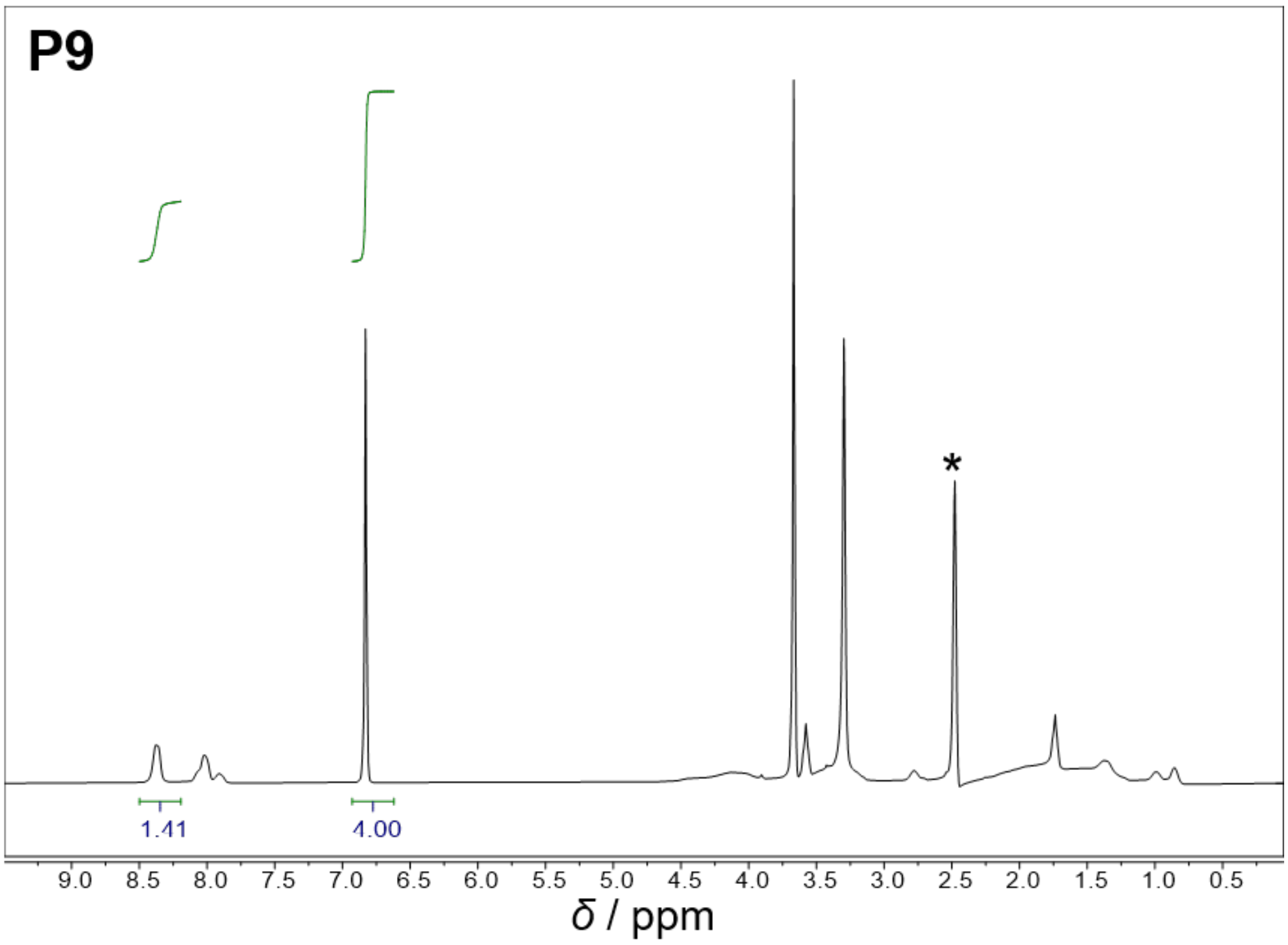

Figure S9. Quantitative ^{1}H NMR spectrum of **P9** (300 MHz, DMSO-d_6, 25 °C) using 1,4-dimethoxybenzene as an internal standard (ISTD). *residual DMSO.

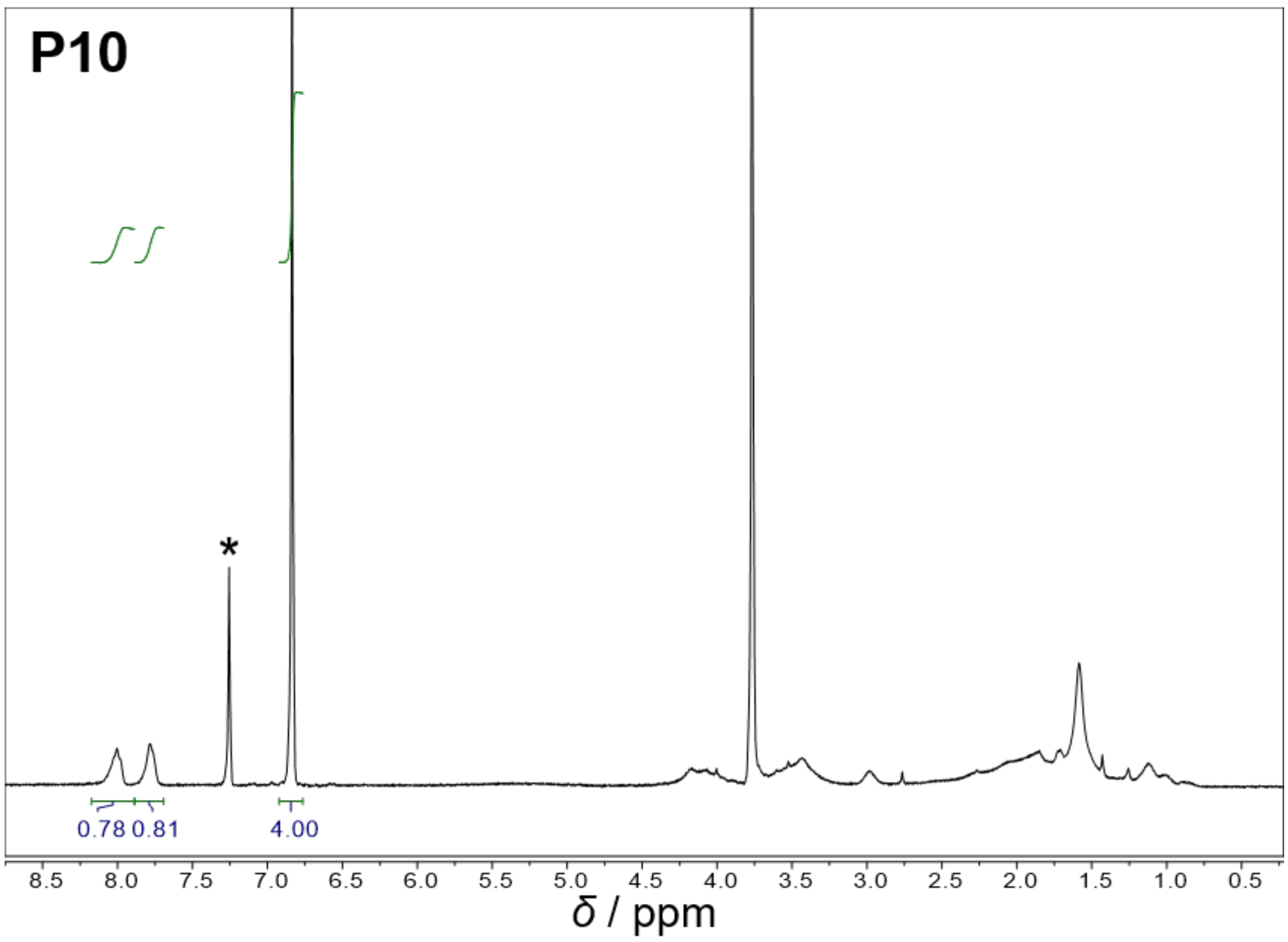

Figure S10. Quantitative ^{1}H NMR spectrum of **P10** (300 MHz, $CDCl_3$, 25 °C) using 1,4-dimethoxybenzene as an internal standard (ISTD). *residual $CHCl_3$.

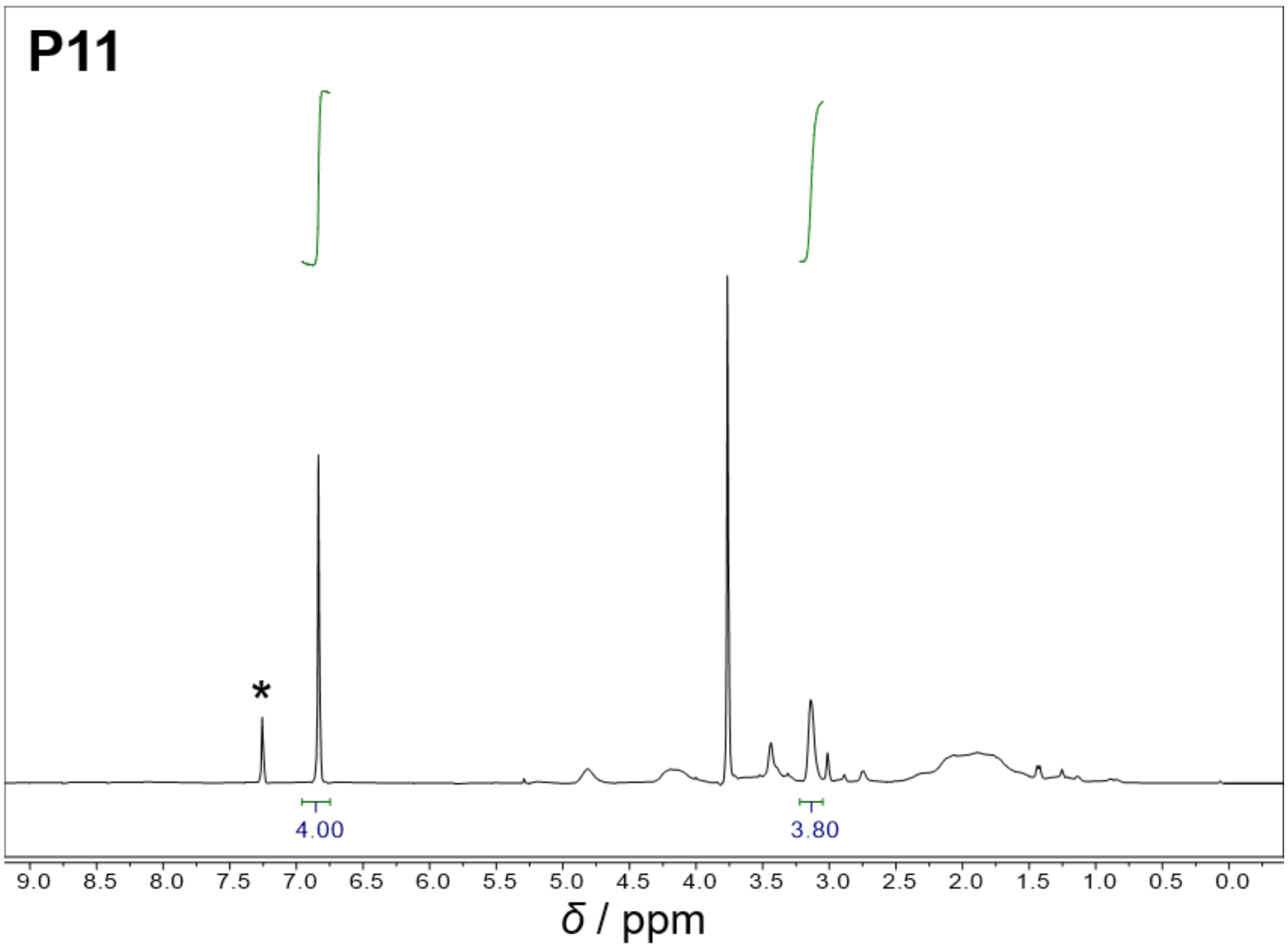

Figure S11. Quantitative ^{1}H NMR spectrum of **P11** (300 MHz, $CDCl_3$, 25 °C) using 1,4-dimethoxybenzene as an internal standard (ISTD). *residual $CHCl_3$.

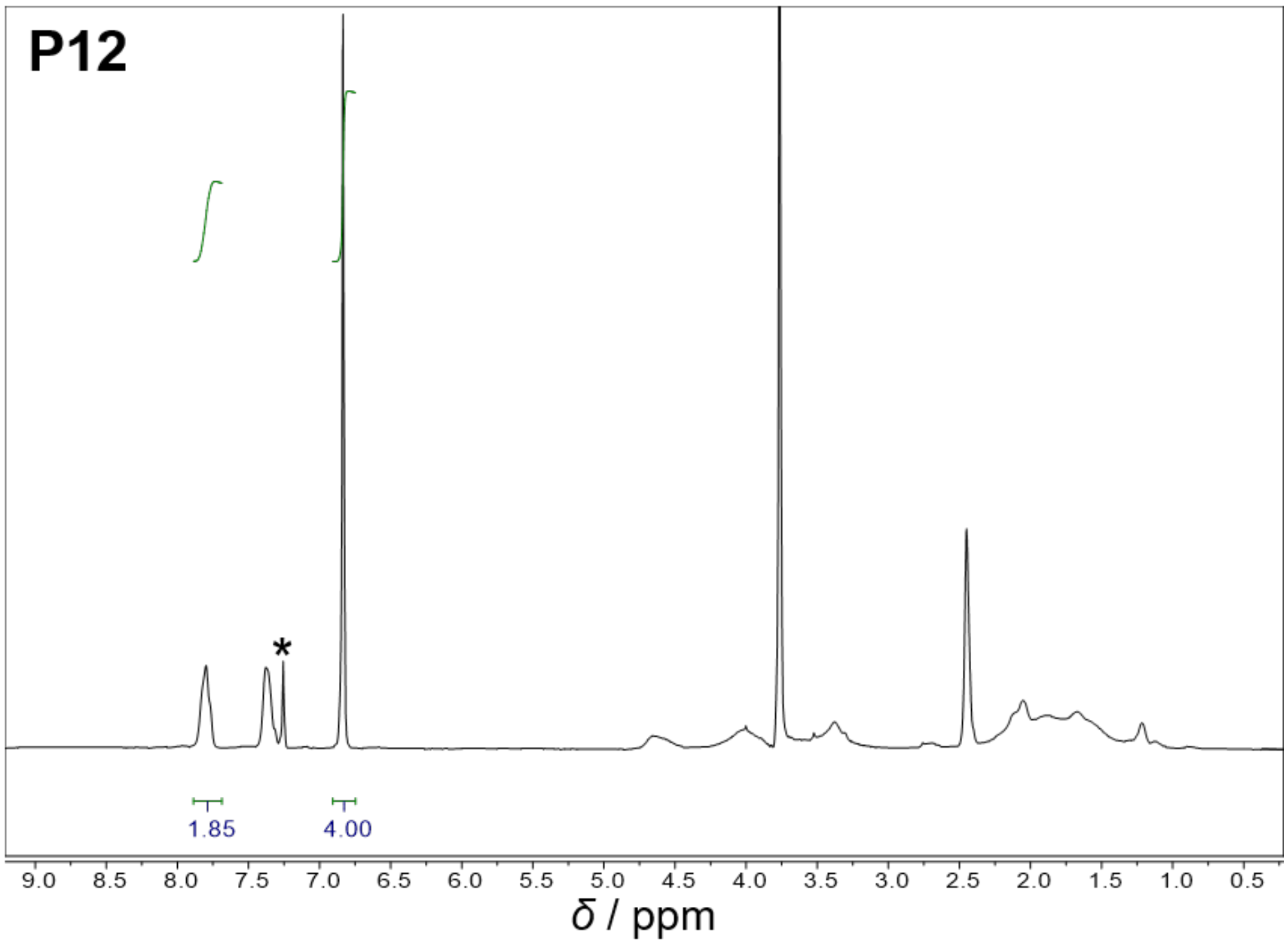

Figure S12. Quantitative ^{1}H NMR spectrum of **P12** (300 MHz, $CDCl_3$, 25 °C) using 1,4-dimethoxybenzene as an internal standard (ISTD). *residual $CHCl_3$.

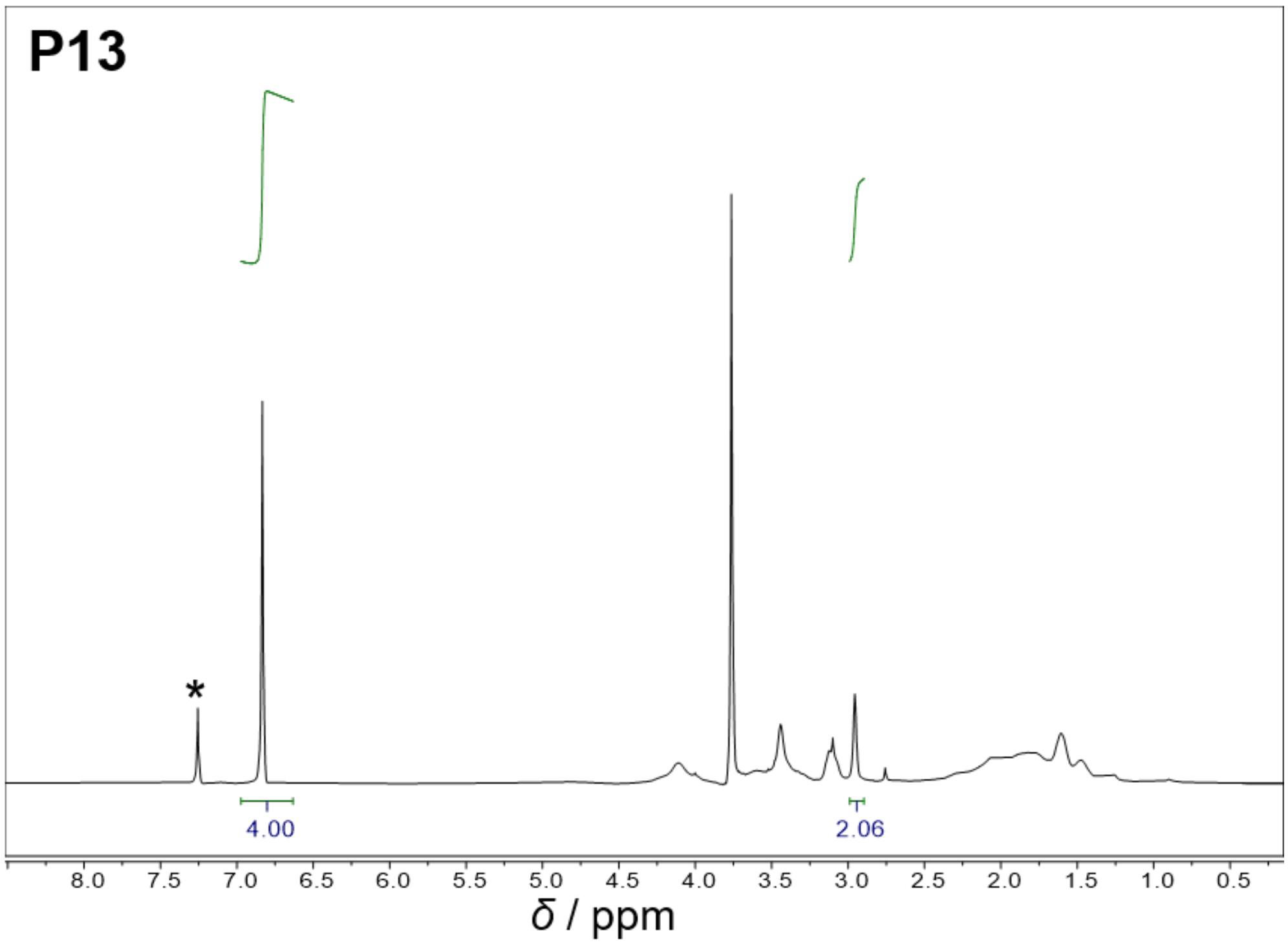

Figure S13. Quantitative ^{1}H NMR spectrum of **P13** (300 MHz, $CDCl_3$, 25 °C) using 1,4-dimethoxybenzene as an internal standard (ISTD). *residual $CHCl_3$.

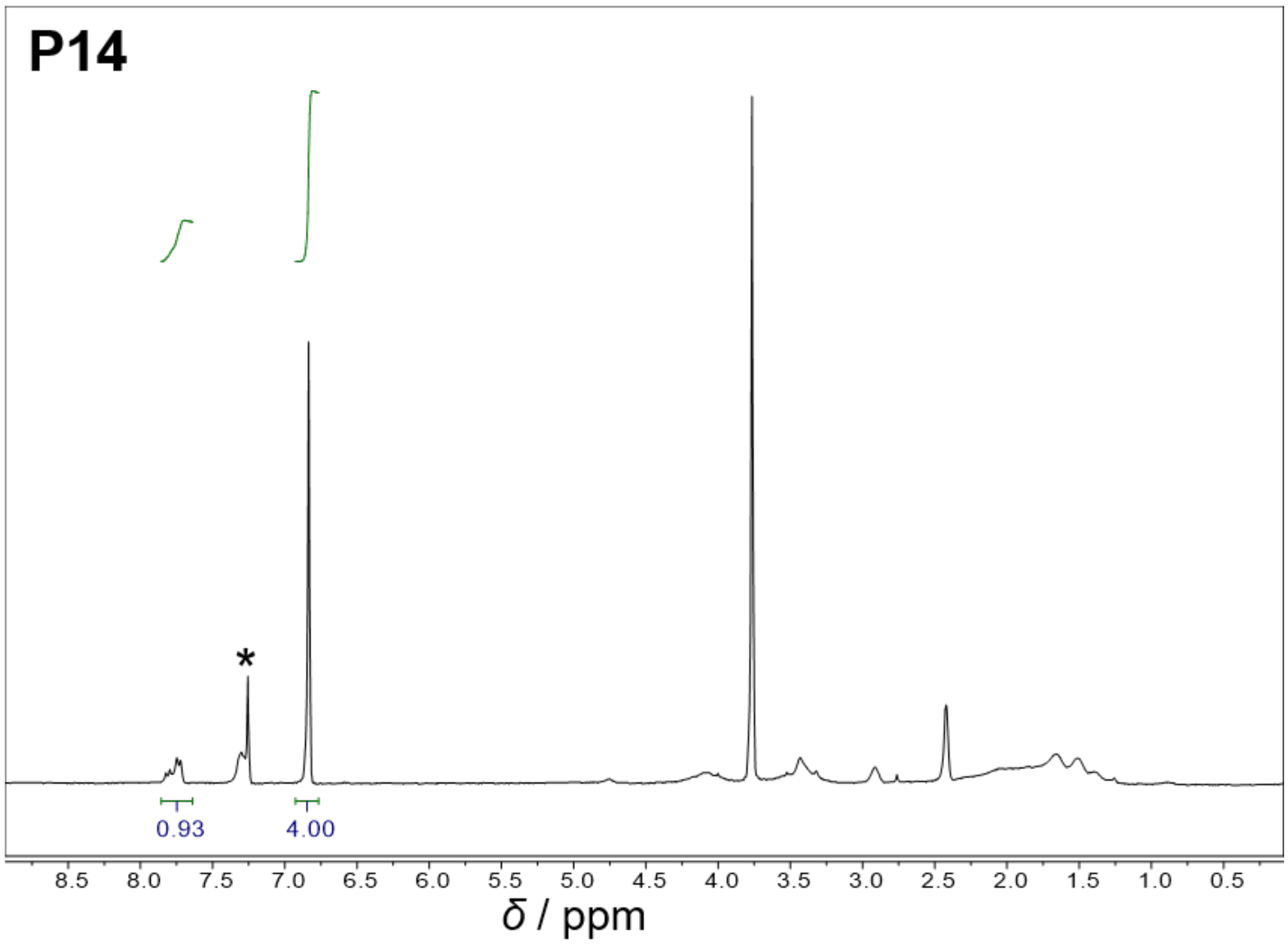

Figure S14. Quantitative ^{1}H NMR spectrum of **P14** (300 MHz, $CDCl_3$, 25 °C) using 1,4-dimethoxybenzene as an internal standard (ISTD). *residual $CHCl_3$.

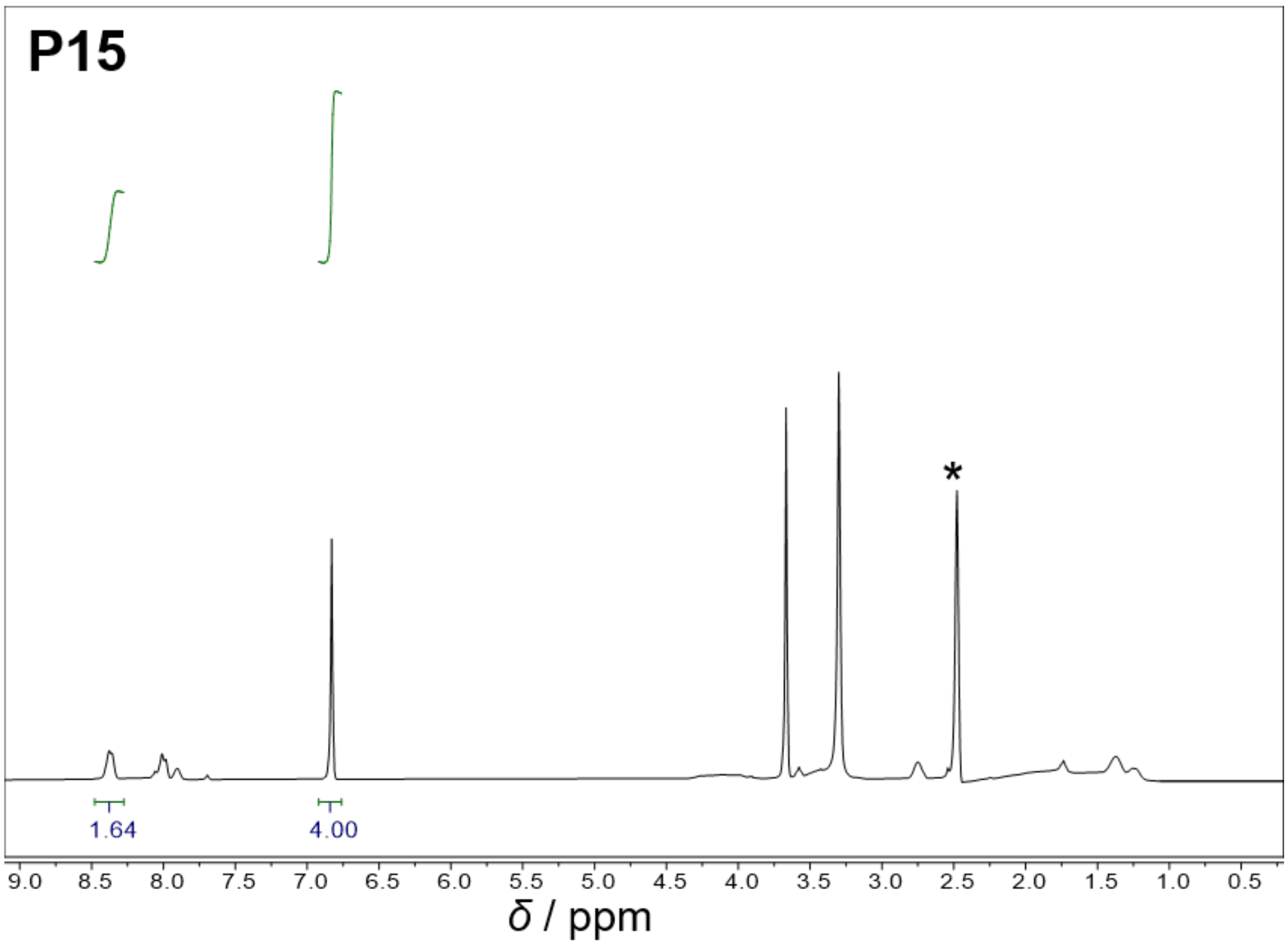

Figure S15. Quantitative ^{1}H NMR spectrum of **P15** (300 MHz, DMSO-d_6, 25 °C) using 1,4-dimethoxybenzene as an internal standard (ISTD). *residual DMSO.

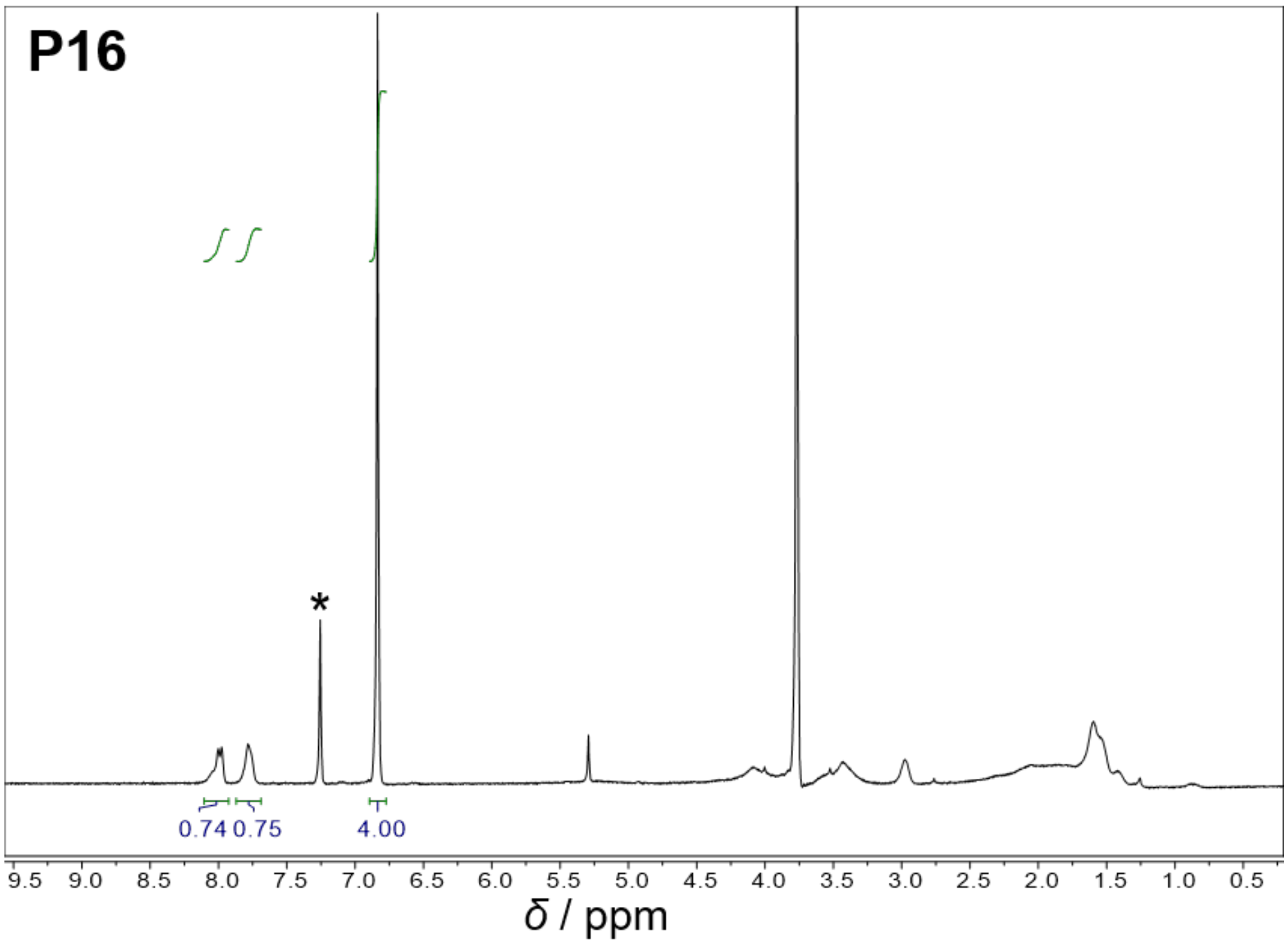

Figure S16. Quantitative ^{1}H NMR spectrum of **P16** (300 MHz, $CDCl_3$, 25 °C) using 1,4-dimethoxybenzene as an internal standard (ISTD). *residual $CHCl_3$.

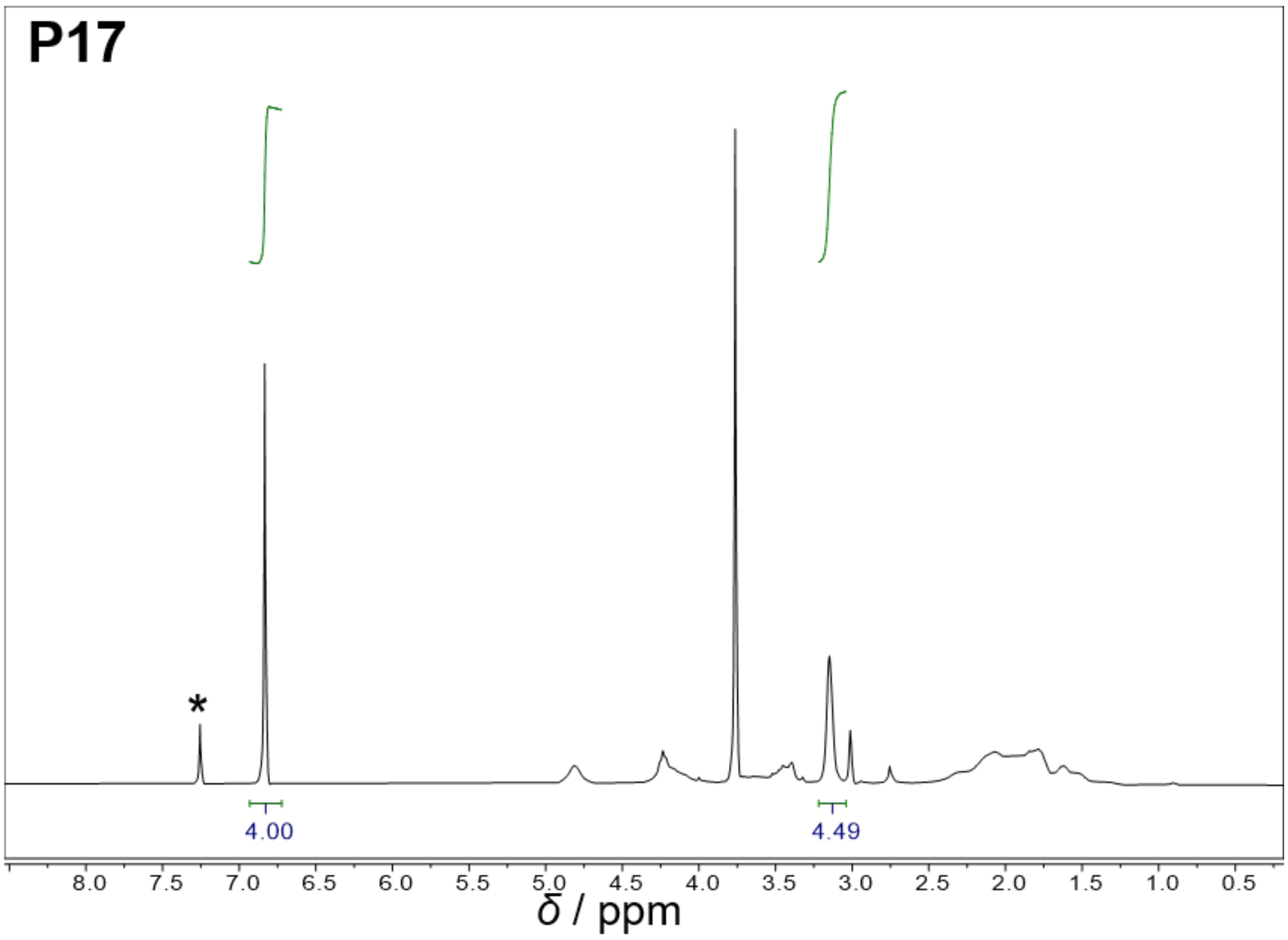

Figure S17. Quantitative ^{1}H NMR spectrum of **P17** (300 MHz, $CDCl_3$, 25 °C) using 1,4-dimethoxybenzene as an internal standard (ISTD). *residual $CHCl_3$.

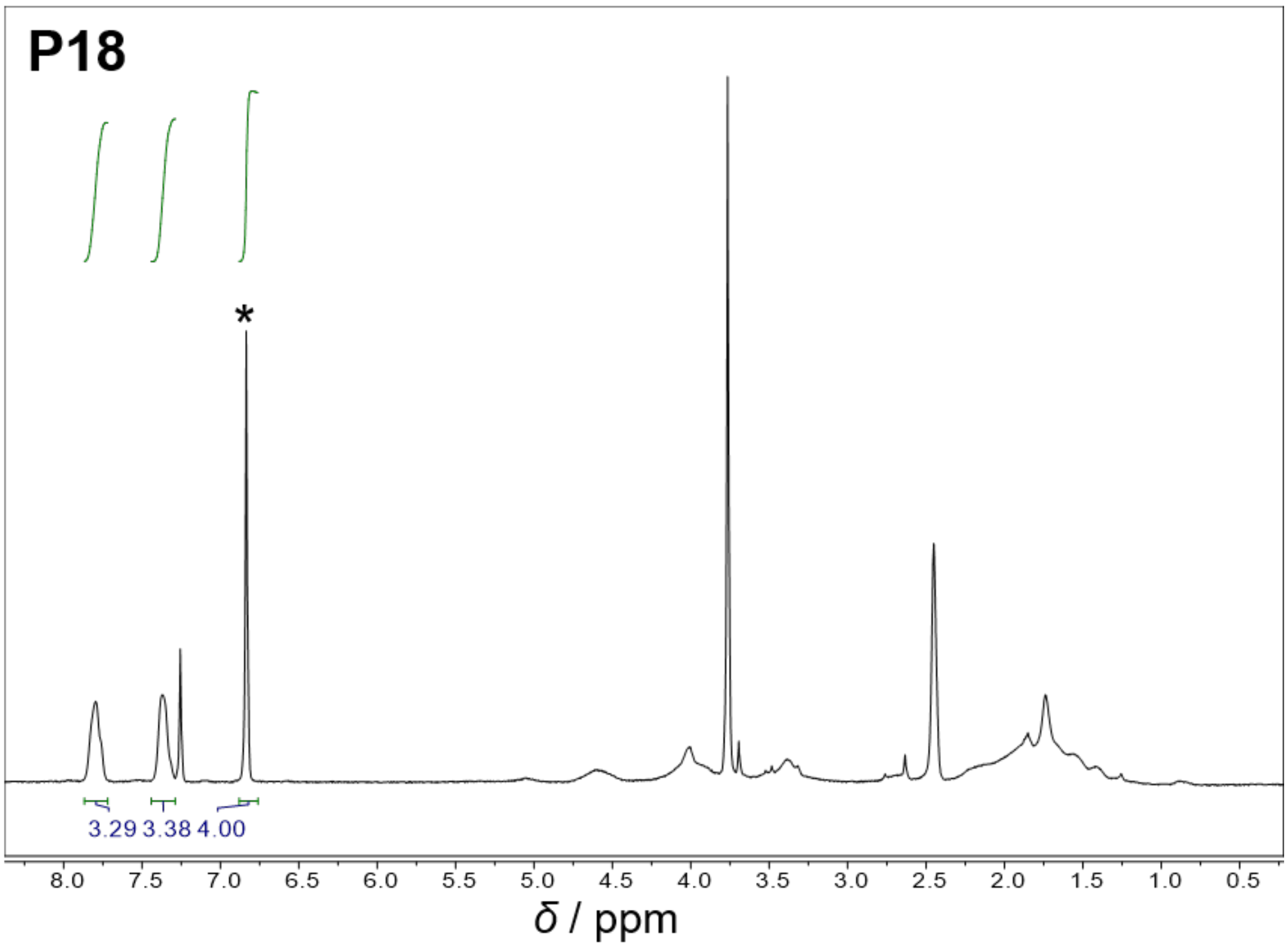

Figure S18. Quantitative ^{1}H NMR spectrum of **P18** (300 MHz, $CDCl_3$, 25 °C) using 1,4-dimethoxybenzene as an internal standard (ISTD). *residual $CHCl_3$.

B. Measurements and analytical methods

B.1 Nuclear magnetic resonance (NMR) spectroscopy

All 1D and 2D NMR spectra were recorded in $CDCl_3$,THF-d_8 or DMSO-d_6 as solvents using a Bruker Avance spectrometer equipped with Ultrashield magnets and operating at 300 MHz for ^{1}H NMR and 75 MHz for ^{13}C NMR. Chemical shifts are reported in parts per million (ppm). The ^{1}H NMR spectra were referenced to the residual proton impurities of the solvents at δ H 3.76, 1.85 ppm (THF-d_8) and 7.26 ($CDCl_3$), respectively. The ^{13}C-NMR spectra were referenced at δ C 25.31, 67.21 and 77.00 ppm to ^{13}THF-d8 and $^{13}CDCl_3$, respectively. Signal multiplicities are abbreviated as s (singlet), d (doublet), t (triplet), q (quartet), and m (multiplet). Coupling constants (*J*) are reported in hertz (Hz).

B.2 Size exclusion chromatography (SEC)

THF: The apparent number average molar mass (M_n) and the molar mass distribution [*Đ* (dispersity index) = M_w/M_n] values of the polymers were determined by using size exclusion chromatography (SEC) measurements on an Agilent 1260 Infinity. The instrument is comprised of an autosampler, a set of columns composed of a guard column (50 × 7.5 mm) and two analytical columns (Polymer Laboratories ResiPore, 300 × 7.5 mm nominal particle size: 3 µm; porosity: 2 µm), G1314B variable wavelength detector operating at 280 nm, a G7800A multidetector suite consisting of a refractive index and a viscosimeter detector. The measurements were conducted with a flow rate of 1 mL min^{-1} at 35 °C using tetrahydrofuran as the eluent. The calibration was carried out by employing different linear polystyrene (PS) standards (EasiVial polystyrene standards from Agilent) ranging from 162 to 3.64 ×10^5 kDa. The polymer samples were dissolved at a concentration of 2 mg mL^{-1} in aforementioned eluent and filtered over a 0.2 *µ*m filter prior to the measurement. Agilent SEC software and multi-detector were used to obtain the molecular weight data (particularly, M_n and *Đ* values). Consequently, the reported M_n and *Đ* values represent apparent SEC-derived values relative to polystyrene calibration. Differences in polymer hydrodynamic behavior and chromatographic interactions with the eluent and stationary phase may additionally influence the apparent molar-mass distributions.

DMAc: The number-average molar mass M_n and molar-mass dispersity (*Đ*= M_w/M_n) of the polymers were determined by size-exclusion chromatography (SEC) using an Agilent Technologies 1260 Infinity II system. The instrument was equipped with an autosampler, a differential refractive-index detector (RID), and a variable-wavelength UV detector (VWD). Separation was performed using a series of three PSS GRAM Lux columns: two PSS GRAM Lux 1000 Å columns and one PSS GRAM Lux 30 Å column, each with a particle size of 5 µm and dimensions of 8 x 300 mm. The combination of columns with different pore sizes provided an extended separation range for polymers with different hydrodynamic volumes. The SEC system was calibrated using PSS ReadyCal Poly(methyl methacrylate) (PMMA) standards covering a nominal molar-mass range from 370 to 2.52 x 10^6 g·mol^{-1}. Consequently, the reported M_n, M_w and *Đ* values represent apparent molar masses relative to PMMA calibration and should not be interpreted as absolute molar masses. Differences between the hydrodynamic behavior of the measured polymers and that of the PMMA standards may therefore cause systematic deviations in the reported values. SEC samples were prepared at a concentration of 2 mg·mL^{-1}using the SEC eluent as the solvent and filtered over a 0.2 *µ*m filter prior to the measurement. HPLC-grade DMAc containing lithium bromide (LiBr; 0.79 g per 2.5 L of DMAc, corresponding to 0.316 g·L^{-1} or approximately 3.6 mmol·L^{-1}) was used as the mobile phase. LiBr was added to reduce polymer aggregation and minimize specific

interactions between polar polymer groups and the stationary phase. Measurements were conducted at 50ºC using a constant mobile-phase flow rate of 1.0 mL·min^{-1}.

B.3 Transmission Fourier-transform infrared spectroscopy (FTIR)

FTIR spectra were recorded at room temperature in transmission mode on a Bruker FTIR (IFS66/s) spectrometer in the 400-4000 cm^{-1} region with a DTGS detector spectrometer. Thin polymer films were prepared by drop-casting polymer solutions ($CHCl_3$) onto KBr plates, followed by complete evaporation of the solvent. The resulting films were measured directly in transmission mode using a clean KBr plate as the background.

B.4 Thermogravimetric analysis (TGA)

TGA data were recorded on a Thermogravimetric METTLER - TOLEDO TGA / DSC 3+ instrument under a nitrogen atmosphere. Samples were heated from ambient temperature to 600 ºC at 30 ºC min^{-1}.

B.5 Differential scanning calorimetry (DSC)

A Mettler Toledo DSC1 was used for thermal analysis. Samples (5 mg) were analyzed between −80 and 100 °C using a heating–cooling–heating cycle at a rate of 10 °C min^{-1} under a nitrogen atmosphere.; the glass transition temperature (T_g) values for each sample are reported form the second heating cycle of the respective DSC measurements and defined as constructed through the inflection point of the step (the point of steepest slope).

B.6 Atomic Force Microscopy (AFM)

The polymer thin film was deposited onto silicon wafers (1 x 1 cm) through dynamic spin-coating and measured utilizing a Bruker (Veeco) Multimode/Nanoscope 3D atomic force microscope. The polymer (50 mg) was dissolved in $CHCl_3$ (1 mL) and the solution was spin-coated at room temperature and 2000 rpm for 1 minute yielding thin films of approximately 200 nm. AFM measurements were performed in tapping mode under ambient conditions using a scan rate of 1 Hz, and all images were acquired at a resolution of 512 scan lines. The apparent domain diameter was quantified from AFM images using ImageJ/Fiji. The images were first calibrated using the corresponding scale bars, converted to 8-bit grayscale, and thresholded to generate binary masks of the observed domains. Domain sizes were determined using the Analyze Particles function, with the Feret diameter of each detected object taken as the apparent lateral domain size. Objects intersecting the image border and obvious artifacts were excluded from the analysis.

B.7 Transmission Electron Microscopy (TEM)

The samples were characterized by transmission electron microscopy (TEM) using a JEOL ARM-200F microscope operating at an accelerating voltage of 200 kV. Polymer samples were prepared for TEM by solvent casting followed by cryo-ultramicrotomy. The polymer sample (250 mg) was dissolved in $CHCl_3$ (2.5 mL), and the resulting solution was transferred into a 3 mL ceramic crucible. The solvent was slowly evaporated over 5 days under a saturated $CHCl_3$ atmosphere to obtain a homogeneous polymer film. The crucibles were subsequently immersed in liquid nitrogen, and the polymer specimens were carefully removed from the crucibles. Small triangular pieces were cut from the polymer film using a razor blade, mounted

onto specimen pins and sectioned by cryo-ultramicrotomy at -140 °C. The resulting ultrathin sections were collected on TEM grids for analysis.

B.8 **Spin–Lattice Relaxation Time (T1) Analysis**

Low-field ^{1}H NMR relaxation measurements were performed in 10 mm NMR tubes using a Bruker Minispec MQ-20 spectrometer operating at a proton resonance frequency of 20 MHz. Measurements were carried out under static conditions at an instrument temperature of 45 °C. Prior to the relaxation experiments, the proton spin–lattice relaxation time (T1) was determined using an inversion–recovery pulse sequence (180°–τ–90°–acquisition). The measured T1 values were subsequently used to optimize the recycle delay employed in the relaxation measurements.

B.9 **Preparation of P11-Containing Electrolytes, Cell Assembly, and Electrochemical Testing**

An ether-based electrolyte [1 M lithium bis(trifluoromethanesulfonyl)imide (LiTFSI) dissolved in a 1:1 (v/v) mixture of 1,2-dimethoxyethane (DME) and 1,3-dioxolane (DOL), (1M LiTFSI in DME/DOL, 1:1 v/v)] was prepared as a baseline electrolyte while another electrolyte was prepared using 1M LiTFSI in DME/DOL, 1:1 (baseline electrolyte) with P11 as an additive in varying mass concentrations. Varying concentrations of P11 in baseline concentration were prepared at 1.5 wt%, 3.5wt% and 6.0 wt% by mass; these were named as 1M LiTFSI + 1.5 wt%P_11 in DME/DOL (1:1), 1M LiTFSI + 3.5 wt%_P11 in DME/DOL (1:1) and 1M LiTFSI + 6.0 wt%_P11 in DME/DOL (1:1). Each electrolyte was stirred using a magnetic stirrer and the vortex mixer till complete dissolution of P11 was observed. All electrolytes were prepared in an argon-filled glovebox (O_2 <0.9ppm, H_2O <0.1ppm).

Further, 1M LiTFSI + 1.5wt%_P11 in DME/DOL (1:1) was used to assemble half- and full-cell two-electrode cell configuration. The electrode used was prepared by planetary wet ball milling sulfur (sourced from Sigma Aldrich, 99.98%, trace metal basis) (60%), conductive carbon, Super C65 (sourced from MSE Supplies, Tucson, AZ, USA; originally manufactured by Imerys/Timca) (20%), polyethylene oxide [sourced from Sigma-Aldrich, average M_v ~200,000, powder] (20%) (as a binder) and acetonitrile as the solvent. Planetary wet ball milling was performed at 250 rpm for a total net milling time of 3 hours. To manage temperature, the process was split into 18 cycles, with each cycle consisting of 10 minutes of rotation followed by a 10-minute pause. After that, coating was done on a 15μm thick Aluminum current collector, purchased from MSE supplies at a wet thickness of 120 μm using the doctor blade method. Electrode drying was done at 60 °C for 6h and punched at 12 mm diameter. A further drying under vacuum at room temperature for 3h was done and then transferred to the glovebox. It is important to note that sulfur electrodes were prepared in two separate batches. The first batch, with a sulfur loading of approximately 1.2 mg/cm^2, was used for the symmetric S//S cell measurements. A second batch, prepared separately, was used for the Li//S full-cell measurements and gave sulfur loadings of 1.8 mg/cm^2 and 1.74 mg/cm^2 for the cells tested with 100 μL and 70 μL of electrolyte, respectively.

For the symmetric S//S cells, two sulfur electrodes from the first batch with comparable loadings were assembled in a two-electrode Swagelok-type cell, separated by two layers of glass fibre separator (GFD, 12 mm diameter), with 300 μL of 1 M LiTFSI in DME/DOL (1:1 v/v) containing 1.5 wt%_PM11. For the Li//Li symmetric cells, two-electrode Swagelok symmetric cells were also assembled, using similar parameters. For the Li//S full cells, coin cells were assembled using a glass fibre separator (GF/A, 16 mm) and a Li metal ring (16 mm), with 1 M

LiTFSI in DME/DOL containing 1.5 wt%_PM11 as the electrolyte at volumes of 100 μL and 70 μL. Based on the electrode geometric area of 1.131 cm^2 (12 mm diameter), the corresponding sulfur masses were 2.04 mg and 1.97 mg, giving electrolyte-to-sulfur (E/S) ratios of approximately 49 and 36 μL/mg, respectively.

Li//Li symmetric cell measurements

Specifically, Li plating/stripping in Li//Li two-electrode symmetric Swagelok cells (12 mm diameter Li electrodes, GFD separator) was performed galvanostatically at 0.0097 mA/cm^2 (0.011 mA), with each half-cycle limited to 10 h or a 1.000 V cutoff (≈0.097 mAh/cm^2 per half-cycle; no capacity limit applied), for 3 full plate/strip cycles (6 half-cycles) with OCV rest between steps. EIS was recorded in a two-electrode configuration before cycling and after the 6th half-cycle (1 MHz–10 mHz, 10 points/decade, 5.0 mV amplitude, 3 averages per point).

S//S symmetric cell measurements

Symmetric S‖S cells were assembled in a two-electrode Swagelok-type cell configuration. Two sulfur-based cathodes with comparable sulfur loadings (~1.2 mg/cm^2 each) were used as working and counter electrodes, separated by two layers of glass fiber separator (GFD, 12 mm diameter). The electrolyte used was 1M LiTFSI + 1.5 wt%_P11 in DME/DOL (1:1), with a total electrolyte volume of 300 μL. Cyclic voltammetry was performed at a scan rate of 0.5 mV/s over a cell-voltage range of −1.6 V to +1.6 V for 2 cycles. The specific current (mA/g) was normalized to the sulfur mass of a single electrode.

Li//S cell measurements

For cyclic voltammetry, coin cells were cycled between 2.8V and 1.7V, at a scan rate of 0.1 mV/s for five consecutive cycles. Electrochemical impedance spectroscopy (EIS) was performed at three stages of the protocol: (i) prior to cycling, at open-circuit voltage (OCV) with no applied DC bias; (ii) after five CV cycles, at a fixed DC potential of 2.8 V vs. Li/Li^+ (fully charged state); and (iii) after galvanostatic cycling, again at 2.8 V vs. Li/Li^+. Spectra were recorded over a frequency range of 10 kHz to 10 mHz, with an AC amplitude of 5 mV for the pre- and post-CV spectra and post-cycling spectrum. For the charge and discharge profile, C-rates of 2C, C and C/2 were used. C-rates were calculated based on the theoretical specific capacity of sulfur (1675 mAh/g), such that 1C corresponds to full theoretical discharge/charge in 1 h. Measurements were performed on two independently assembled cells per condition; curves shown are representative of this dataset. Finally, all electrochemical measurements were performed at ambient laboratory temperature (~23°C), without active temperature control.

C. **Additional Data and Figures**

C.1 **NMR Spectra**

The following conventional ^{1}H NMR spectra are provided for structural characterization. Owing to the broad and overlapping polymer resonances, relative peak heights and integrals should not be interpreted as quantitative proton ratios. Functionalization degrees were determined independently using the internal-standard qNMR procedure described in **Section A.4**.

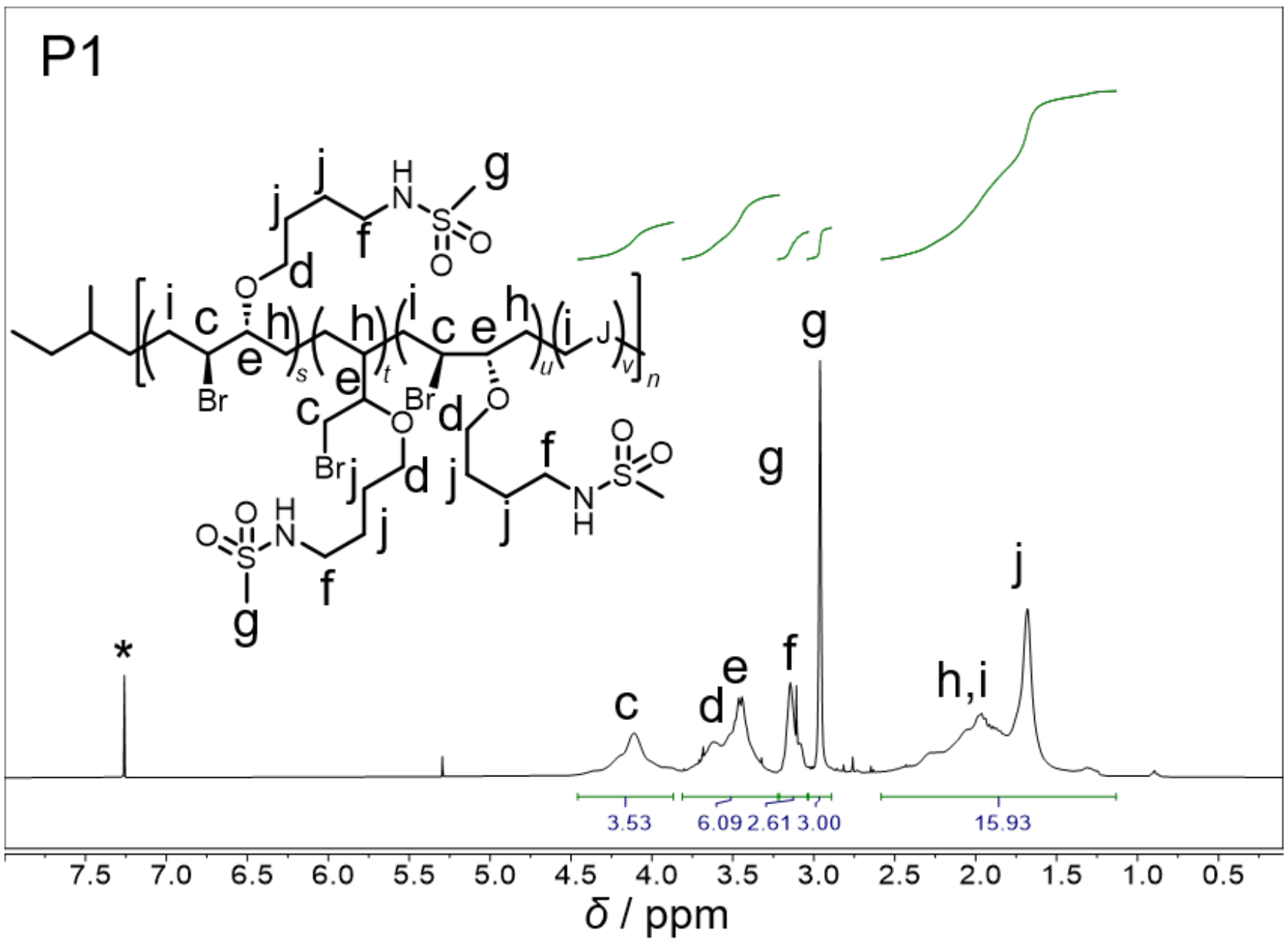

Figure S19. ^{1}H NMR spectrum of **P1** recorded in $CDCl_3$ at 300 MHz. The residual solvent signal is marked with an asterisk.

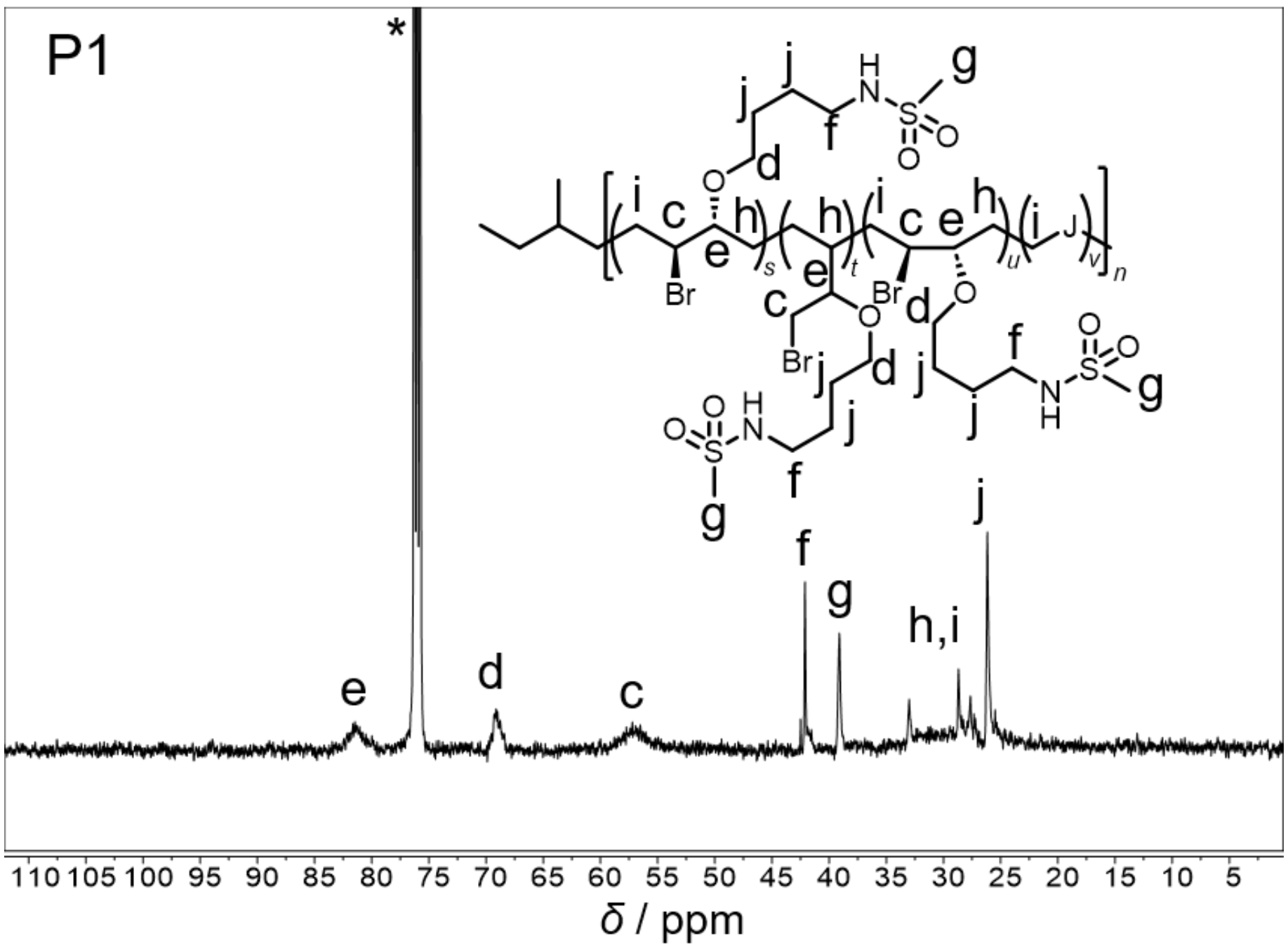

Figure S20. ^{13}C NMR spectrum of **P1** recorded in $CDCl_3$ at 75 MHz. The residual solvent signal is marked with an asterisk.

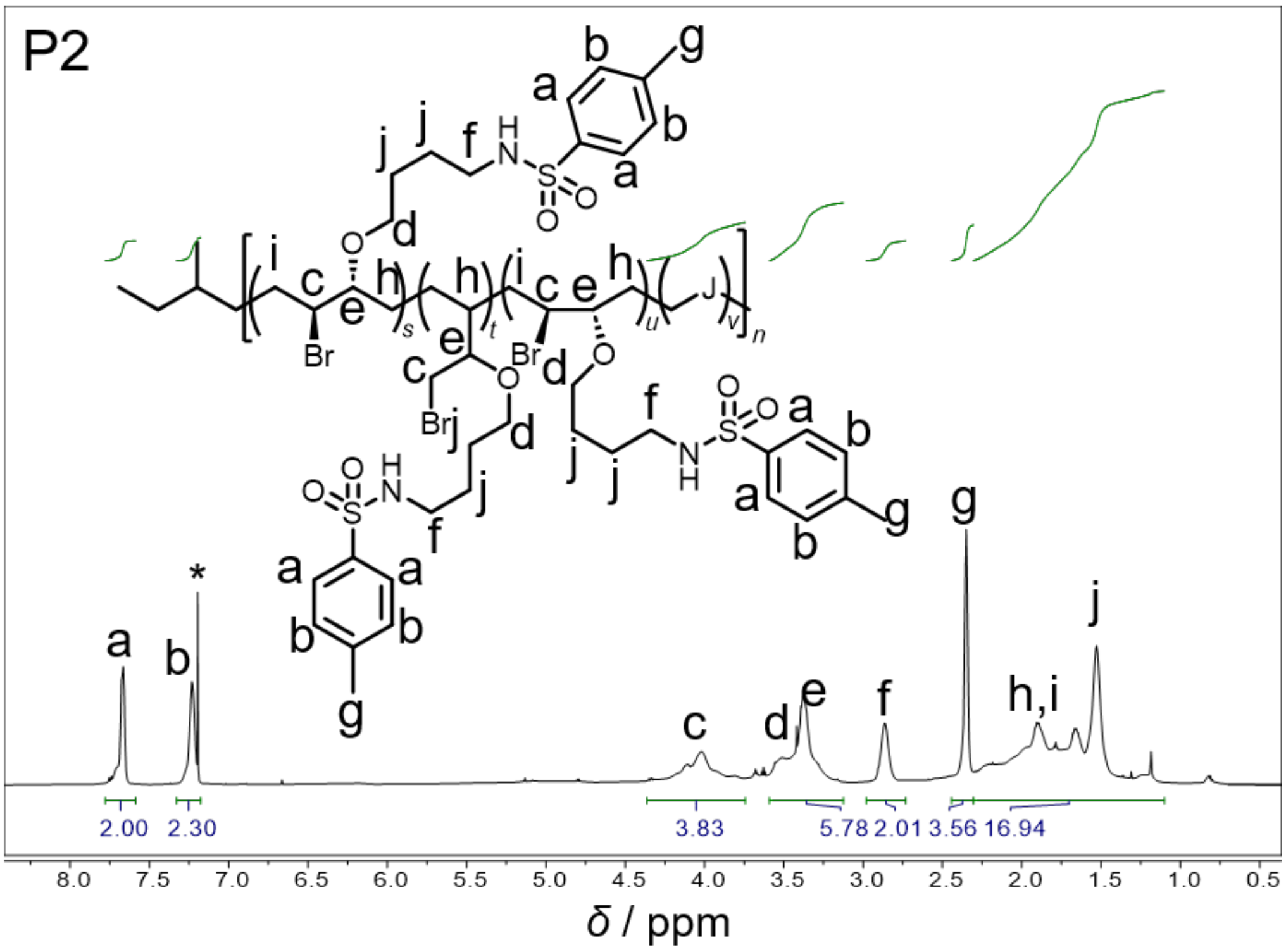

Figure S21. ^{1}H NMR spectrum of **P2** recorded in $CDCl_3$ at 300 MHz. The residual solvent signal is marked with an asterisk.

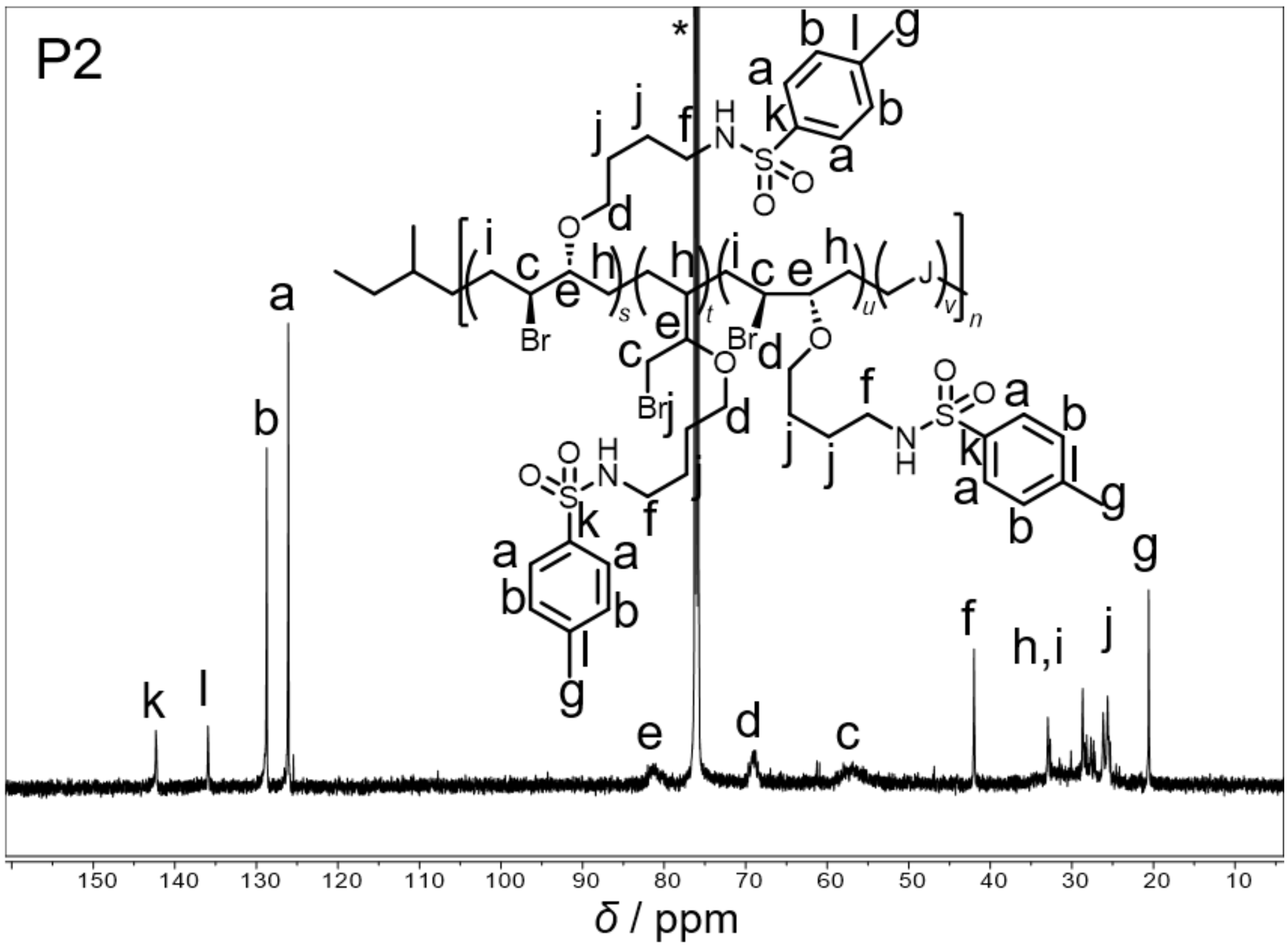

Figure S22. ^{13}C NMR spectrum of **P2** recorded in $CDCl_3$ at 75 MHz. The residual solvent signal is marked with an asterisk.

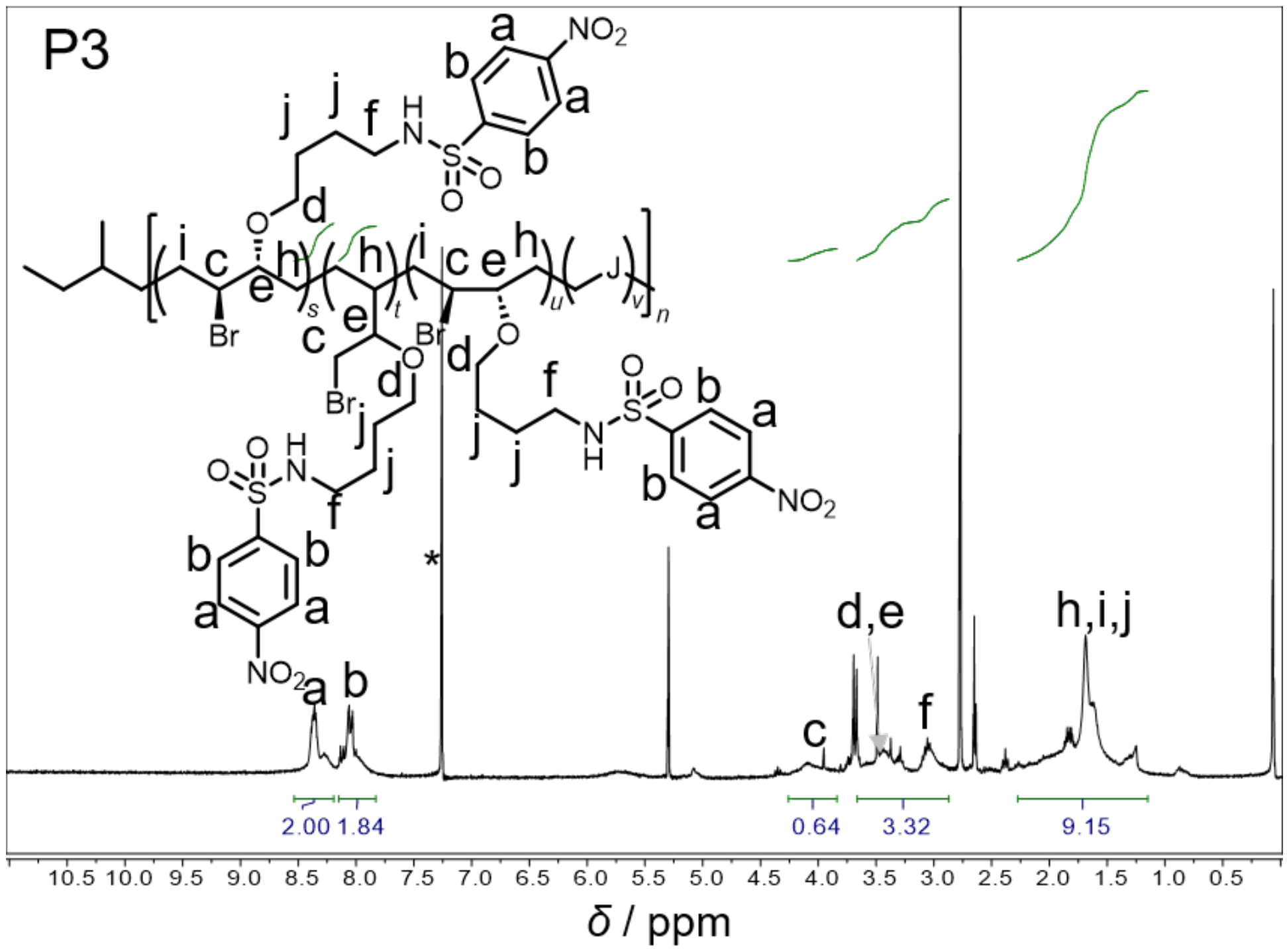

Figure S23. ^{1}H NMR spectrum of **P3** recorded in $CDCl_3$ at 300 MHz. The residual solvent signal is marked with an asterisk.

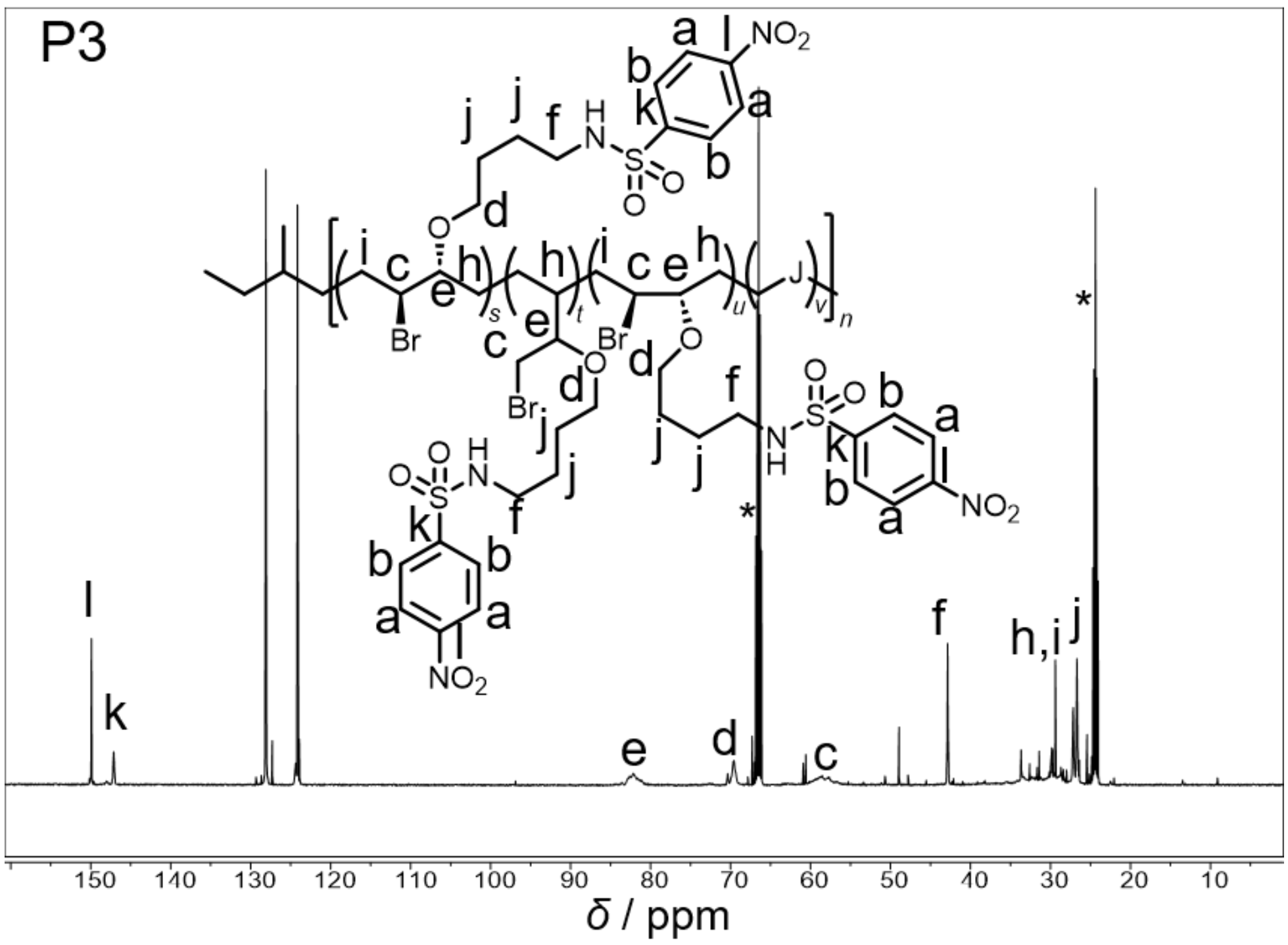

Figure S24. ^{13}C NMR spectrum of **P3** recorded in THF-d8 at 75 MHz. The residual solvent signal is marked with an asterisk.

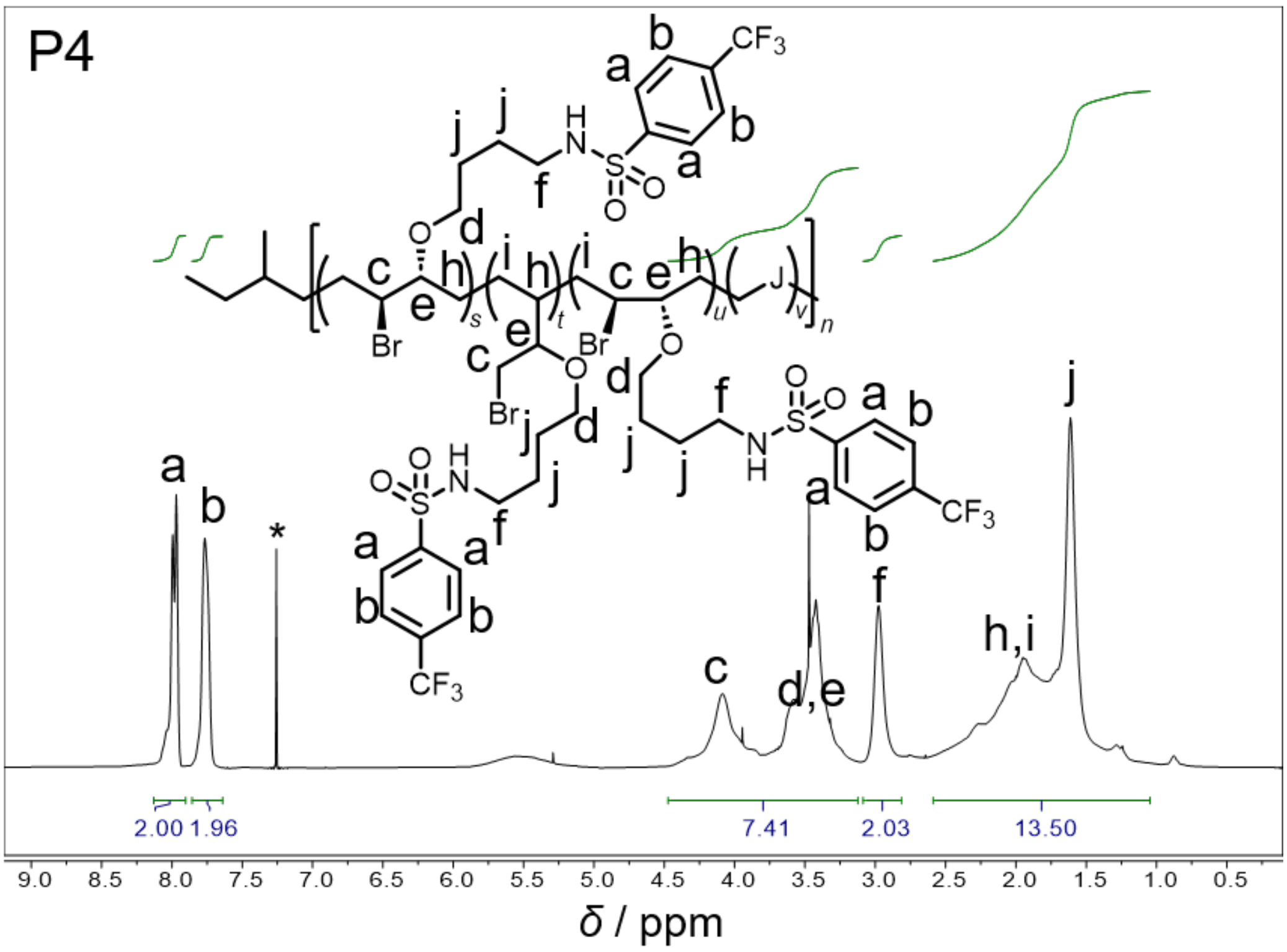

Figure S25. ^{1}H NMR spectrum of **P4** recorded in $CDCl_3$ at 300 MHz. The residual solvent signal is marked with an asterisk.

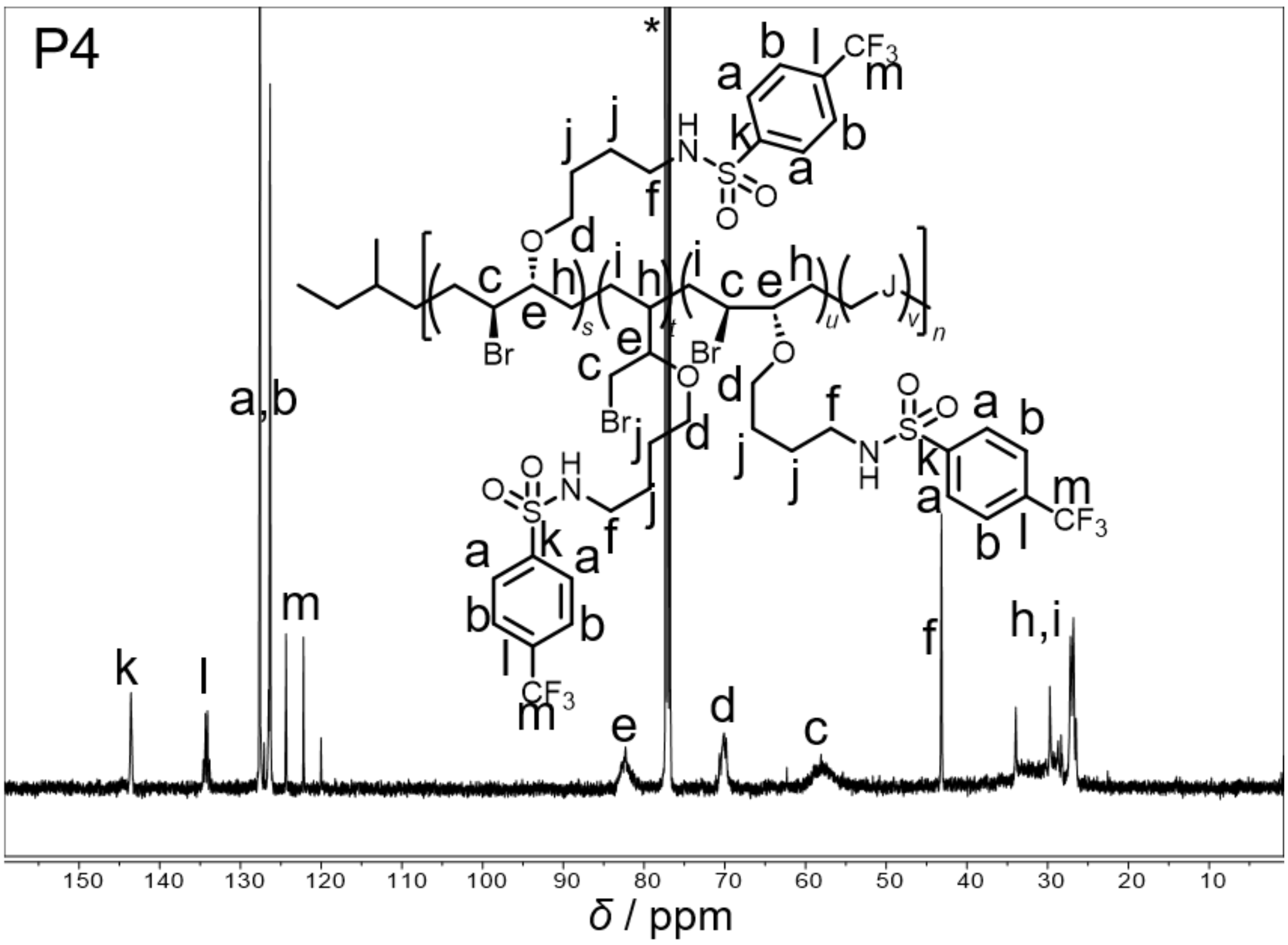

Figure S26. ^{13}C NMR spectrum of **P4** recorded in $CDCl_3$ at 75 MHz. The residual solvent signal is marked with an asterisk.

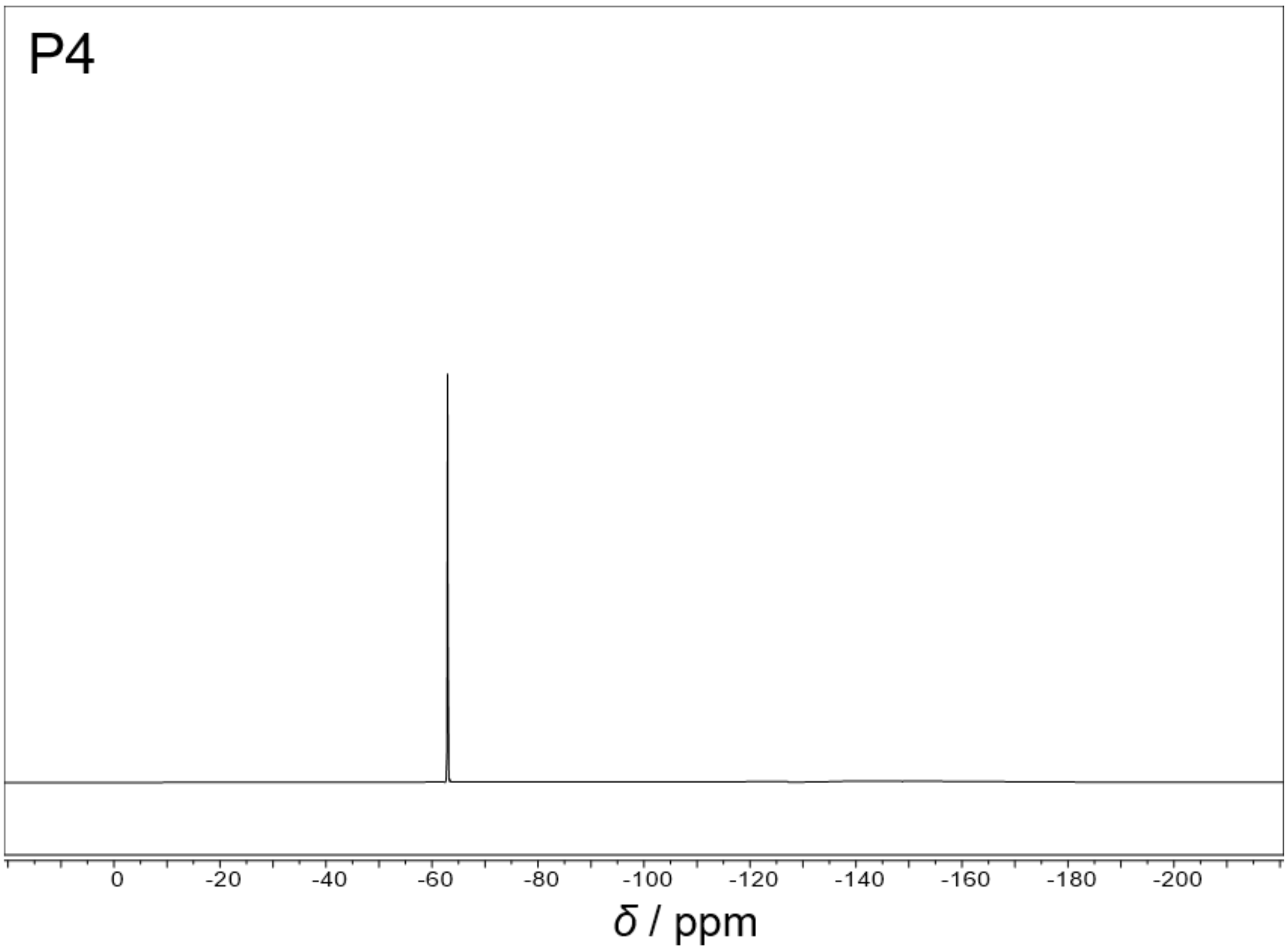

Figure S27. ^{19}F NMR spectrum of **P4** recorded in $CDCl_3$ at 282 MHz.

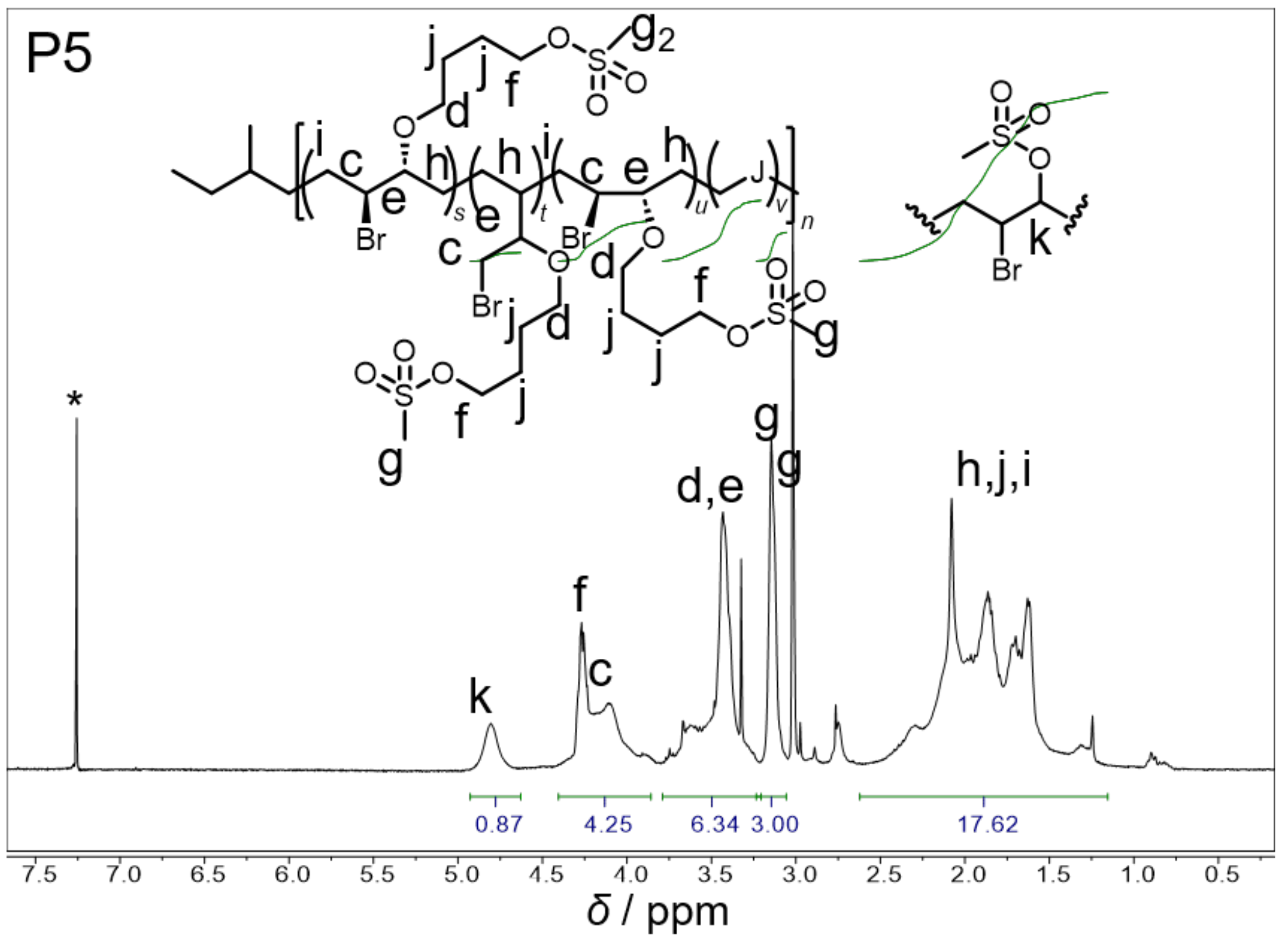

Figure S28. ^{1}H NMR spectrum of **P5** recorded in $CDCl_3$ at 300 MHz. The residual solvent signal is marked with an asterisk.

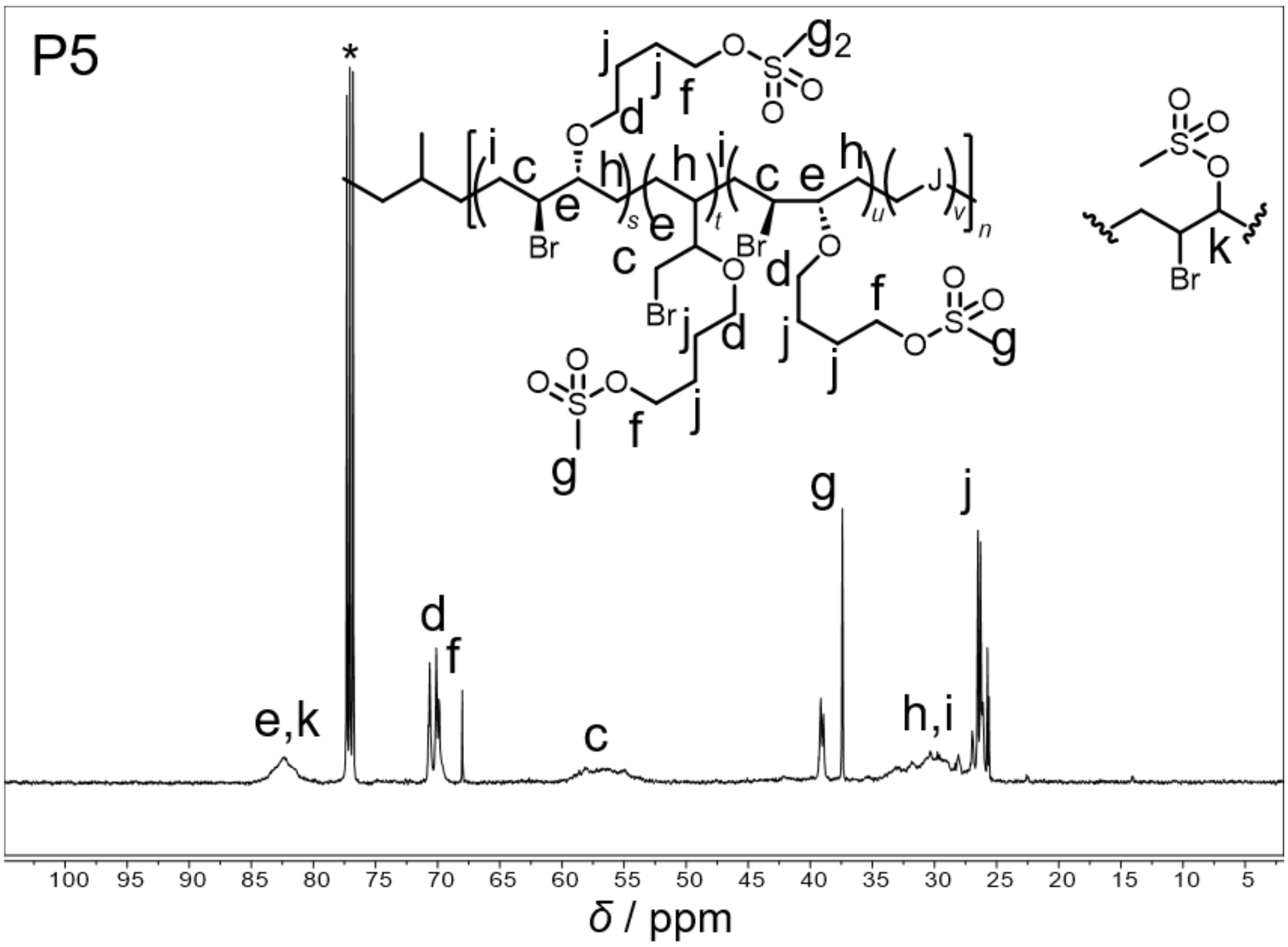

Figure S29. ^{13}C NMR spectrum of **P5** recorded in $CDCl_3$ at 75 MHz. The residual solvent signal is marked with an asterisk.

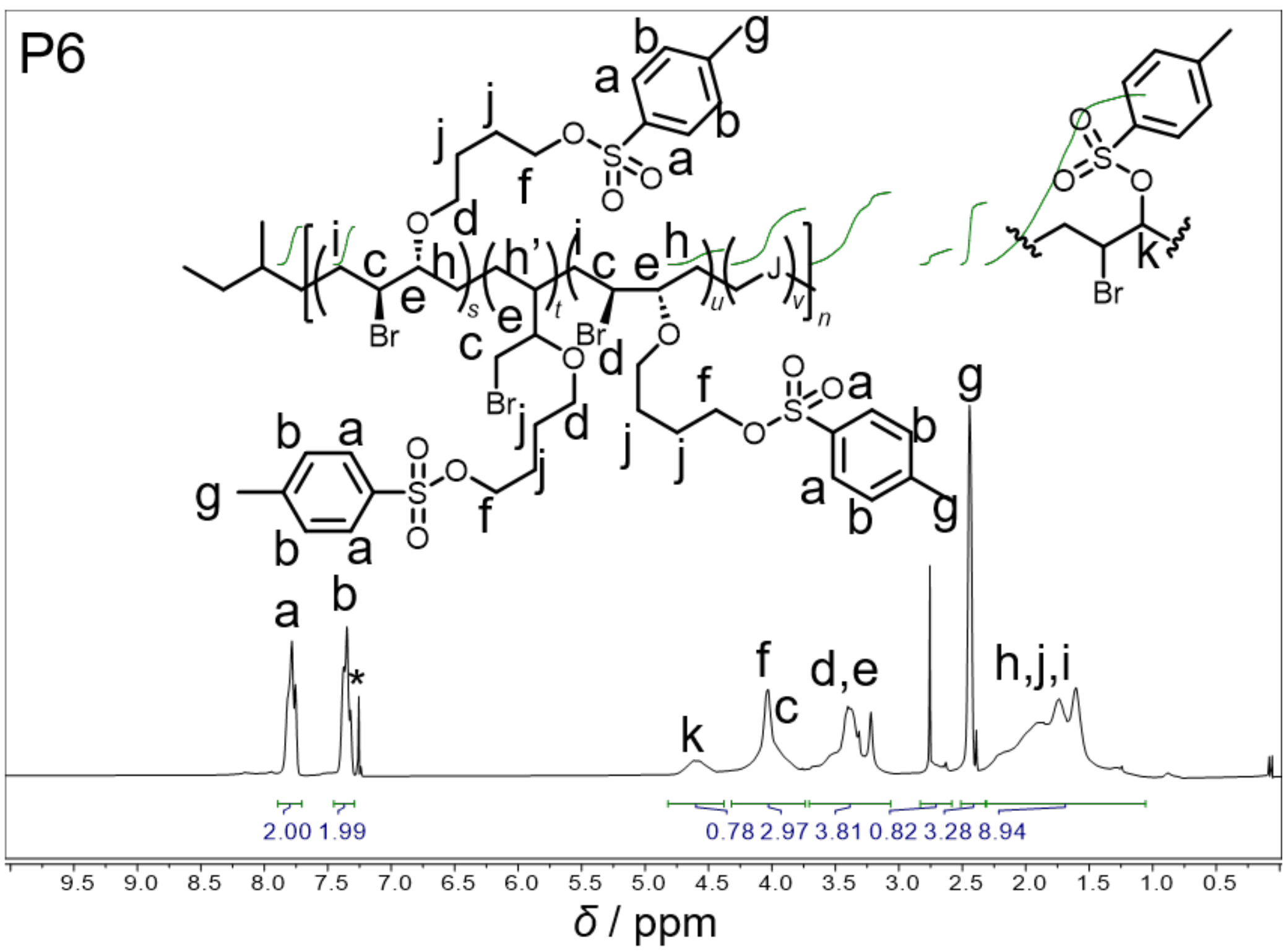

Figure S30. ^{1}H NMR spectrum of **P6** recorded in $CDCl_3$ at 300 MHz. The residual solvent signal is marked with an asterisk.

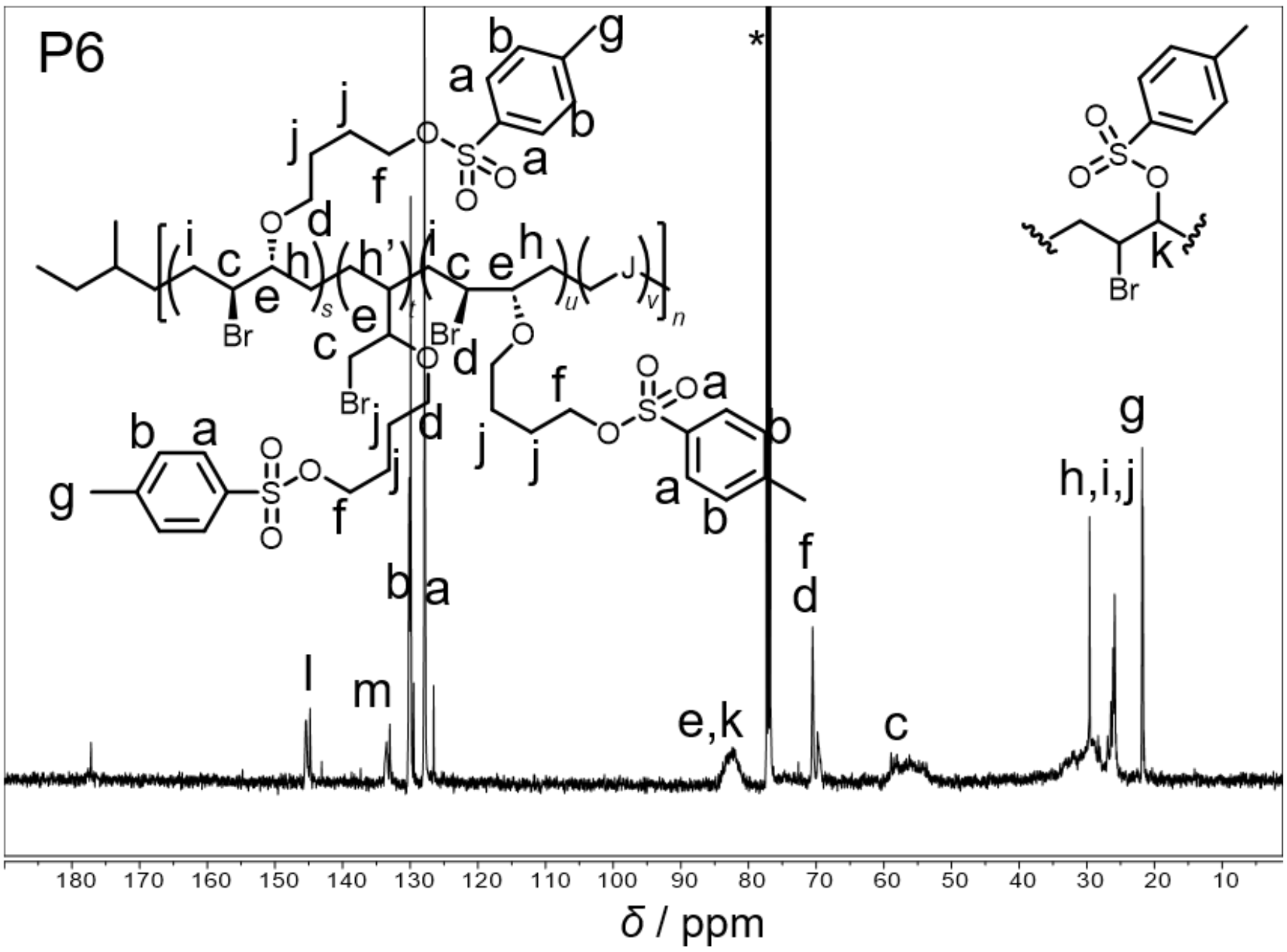

Figure S31. ^{13}C NMR spectrum of **P6** recorded in $CDCl_3$ at 75 MHz. The residual solvent signal is marked with an asterisk.

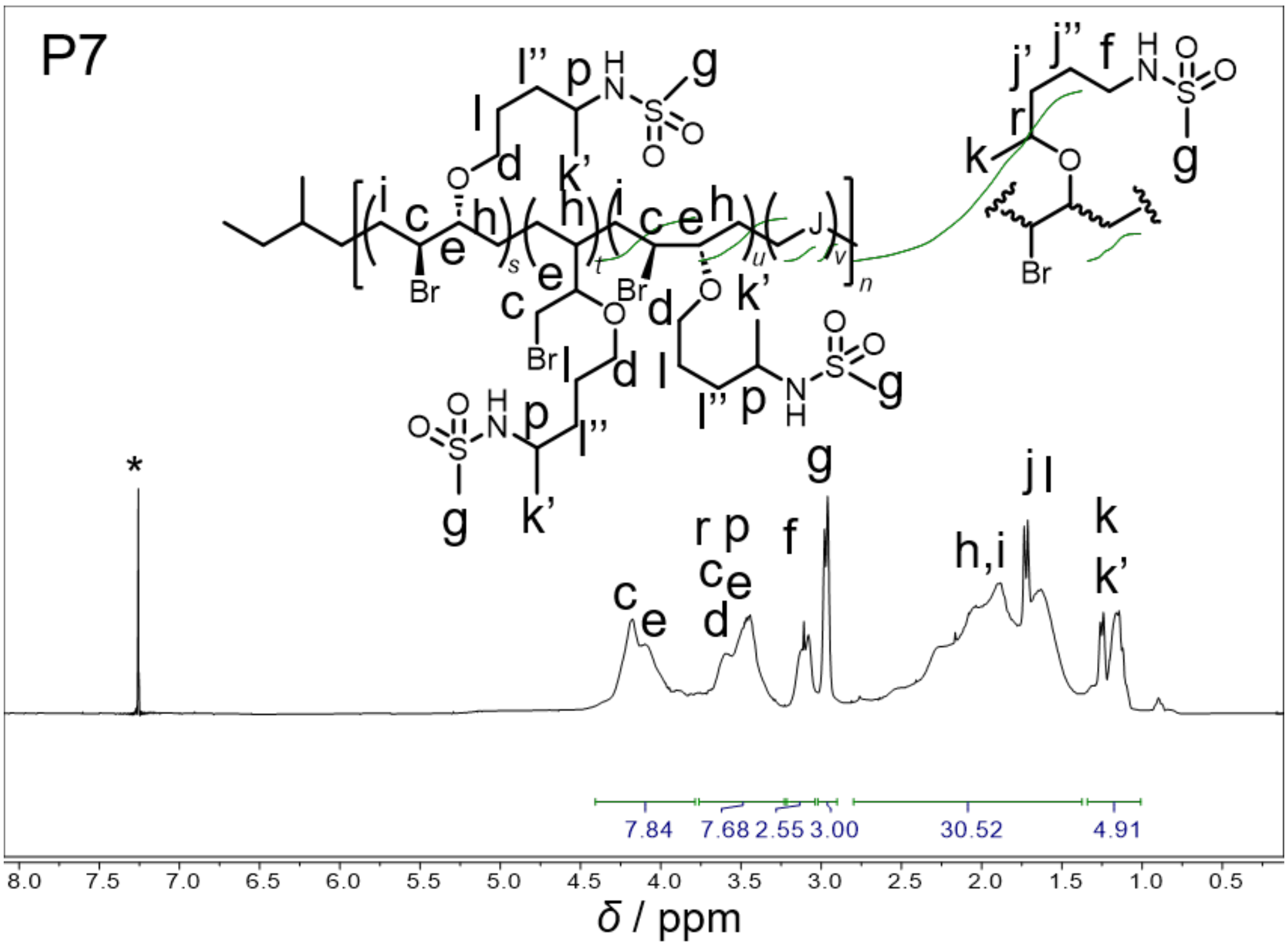

Figure S32. ^{1}H NMR spectrum of **P7** recorded in $CDCl_3$ at 300 MHz. The residual solvent signal is marked with an asterisk.

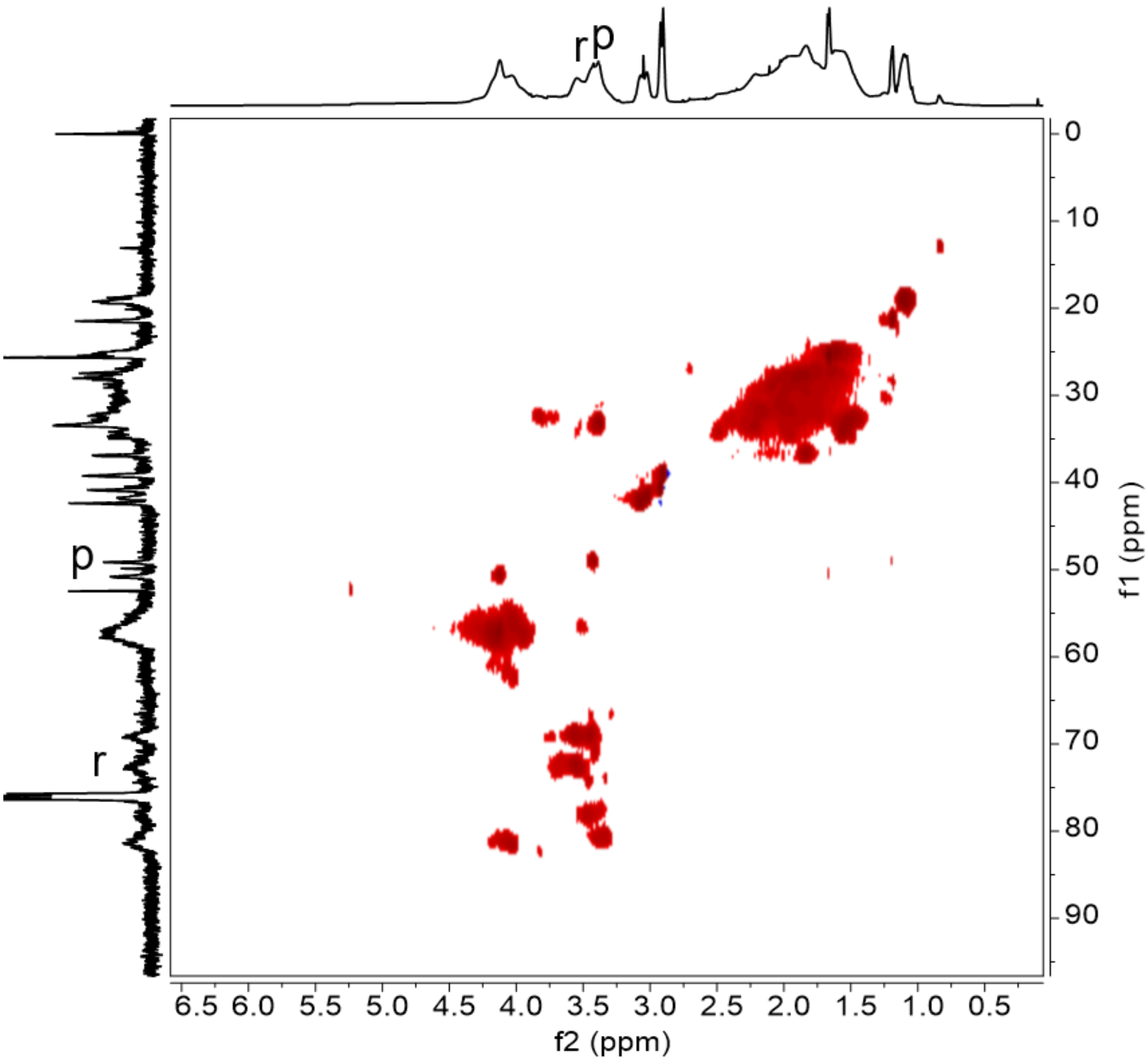

Figure S33. ^{1}H-^{13}C HSQC NMR spectrum of **P7** recorded in $CDCl_3$.

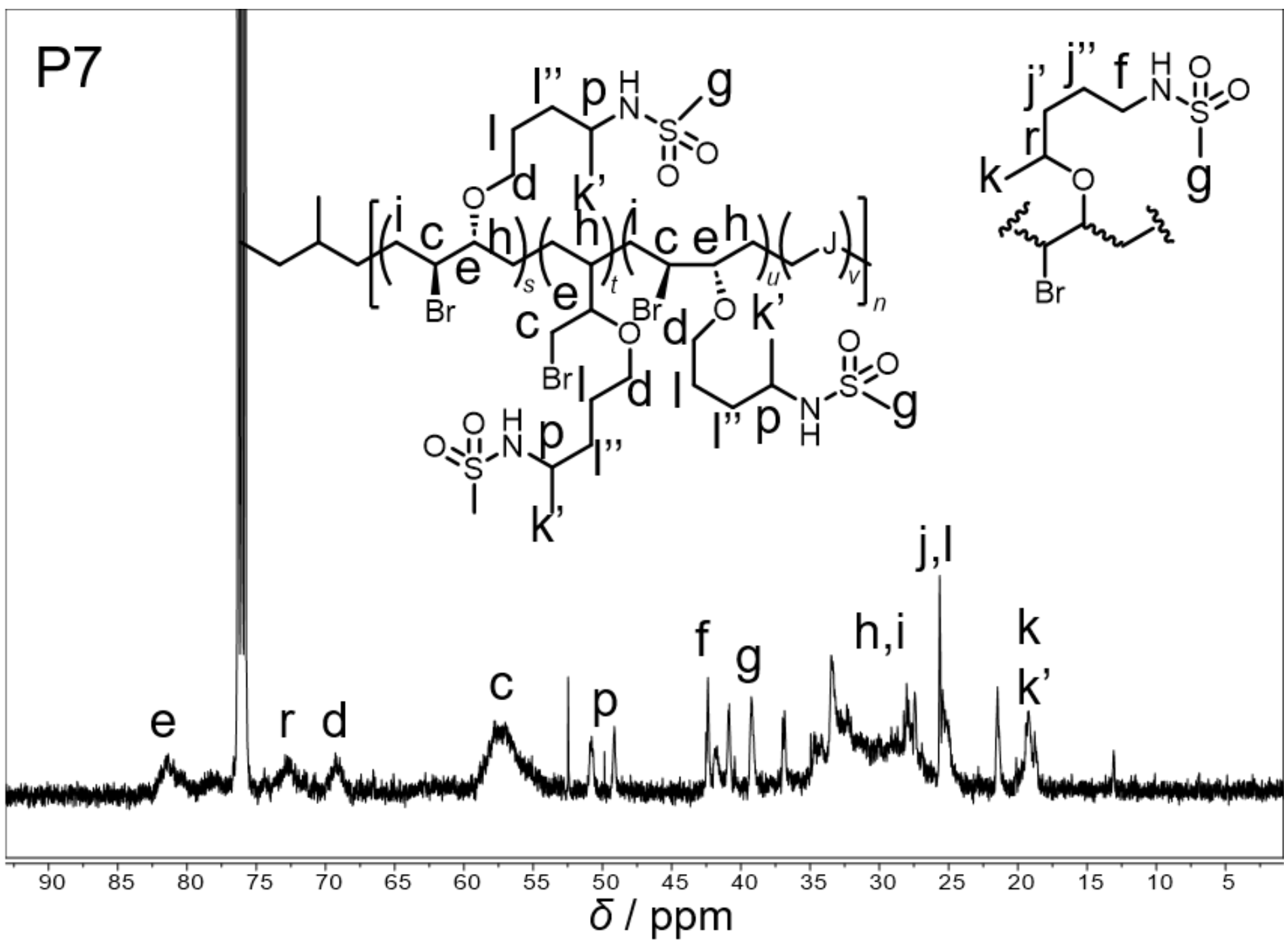

Figure S34. ^{13}C NMR spectrum of **P7** recorded in $CDCl_3$ at 75 MHz. The residual solvent signal is marked with an asterisk.

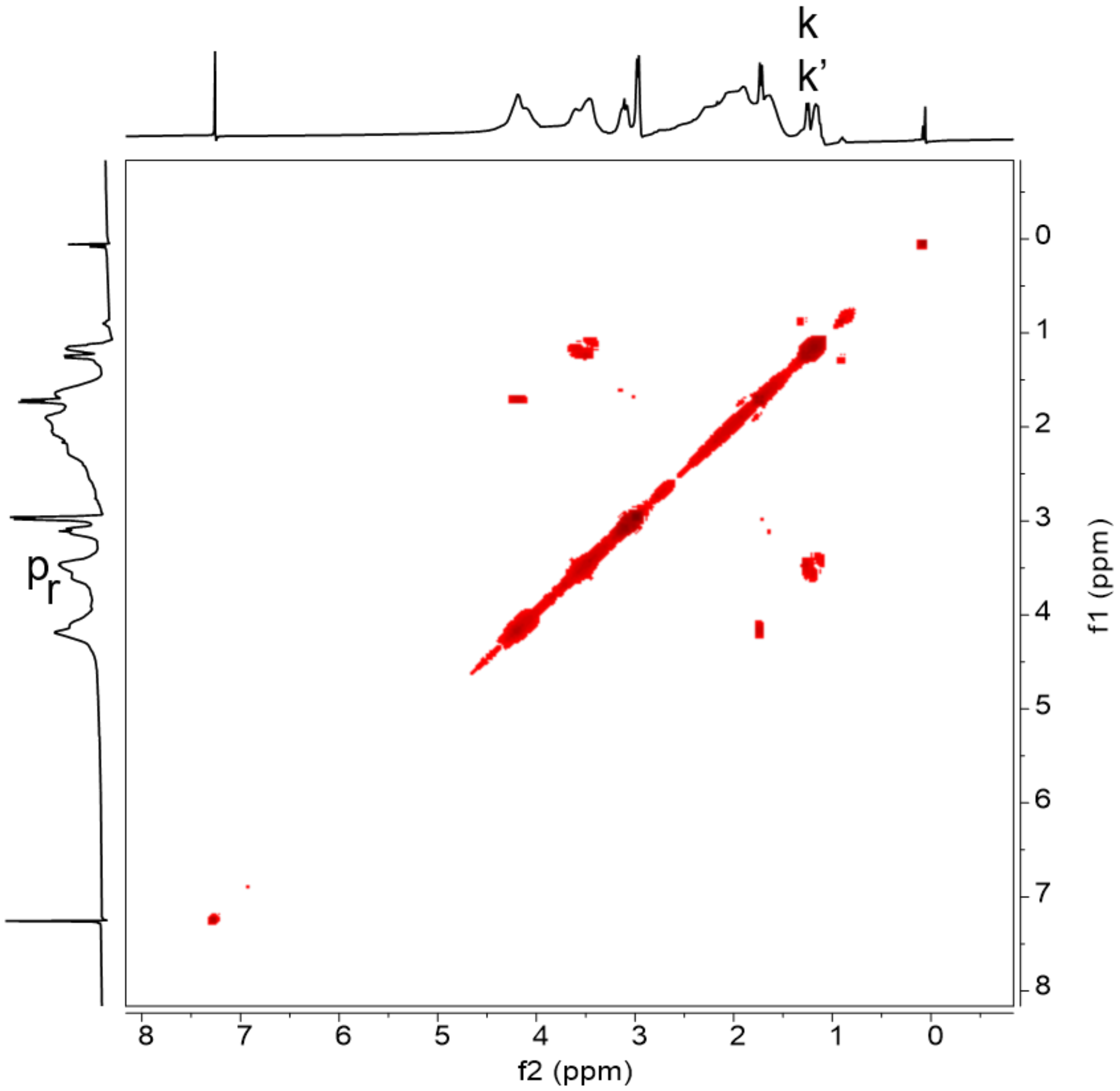

Figure S35. ^{1}H-^{1}H COSY NMR spectrum of **P7** recorded in $CDCl_3$.

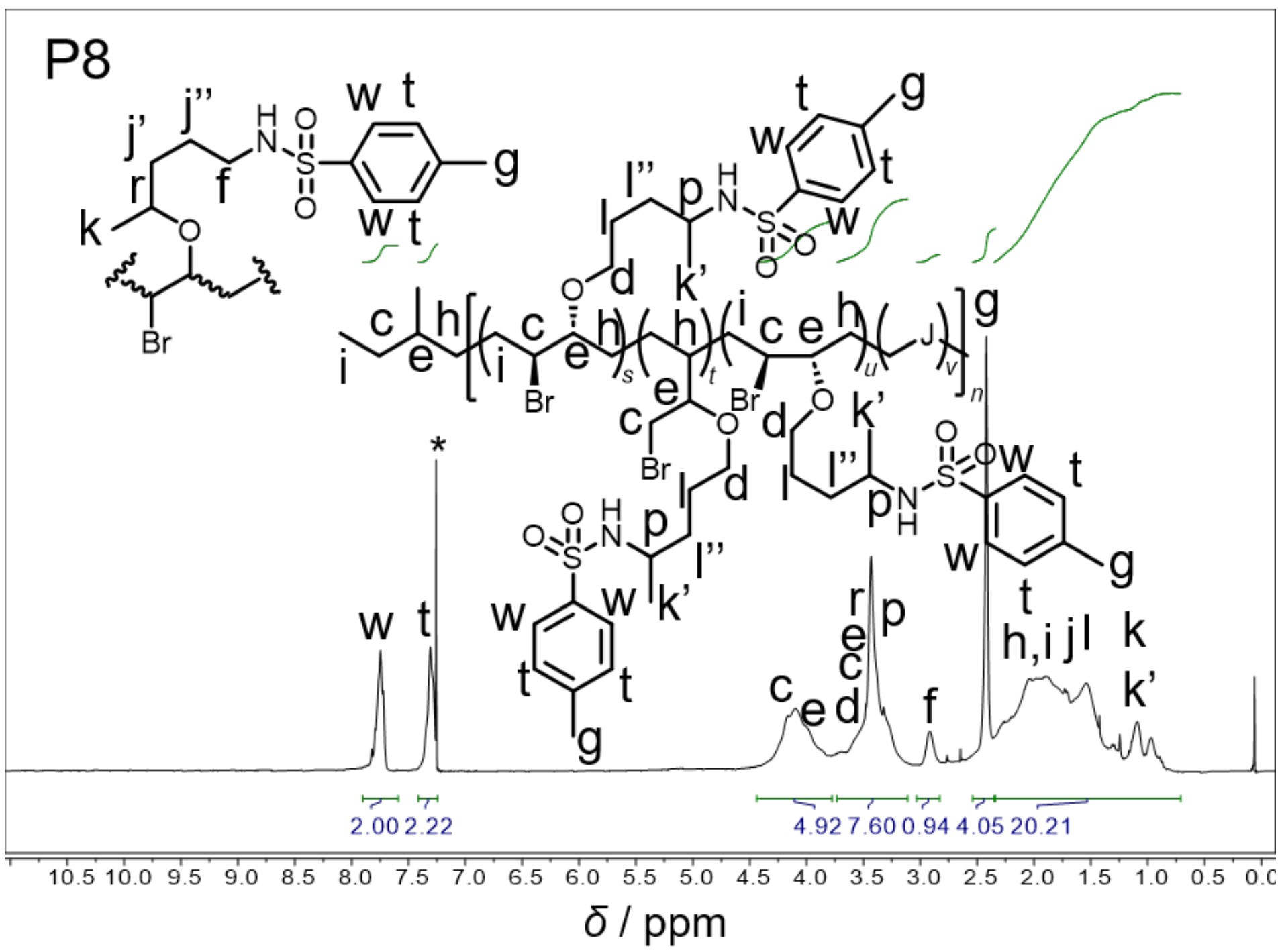

Figure S36. ^{1}H NMR spectrum of **P8** recorded in $CDCl_3$ at 300 MHz. The residual solvent signal is marked with an asterisk.

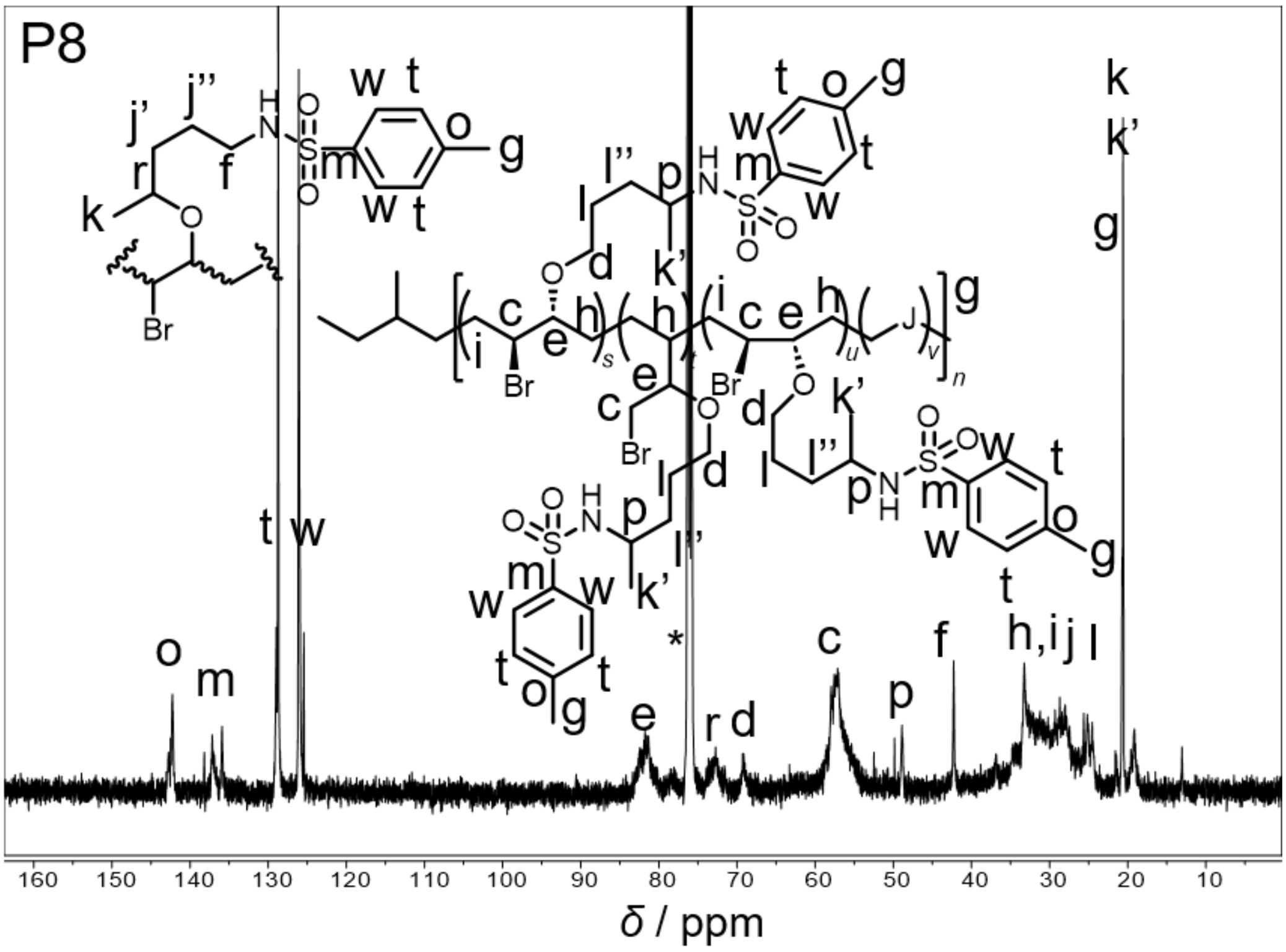

Figure S37. ^{13}C NMR spectrum of **P8** recorded in $CDCl_3$ at 75 MHz. The residual solvent signal is marked with an asterisk.

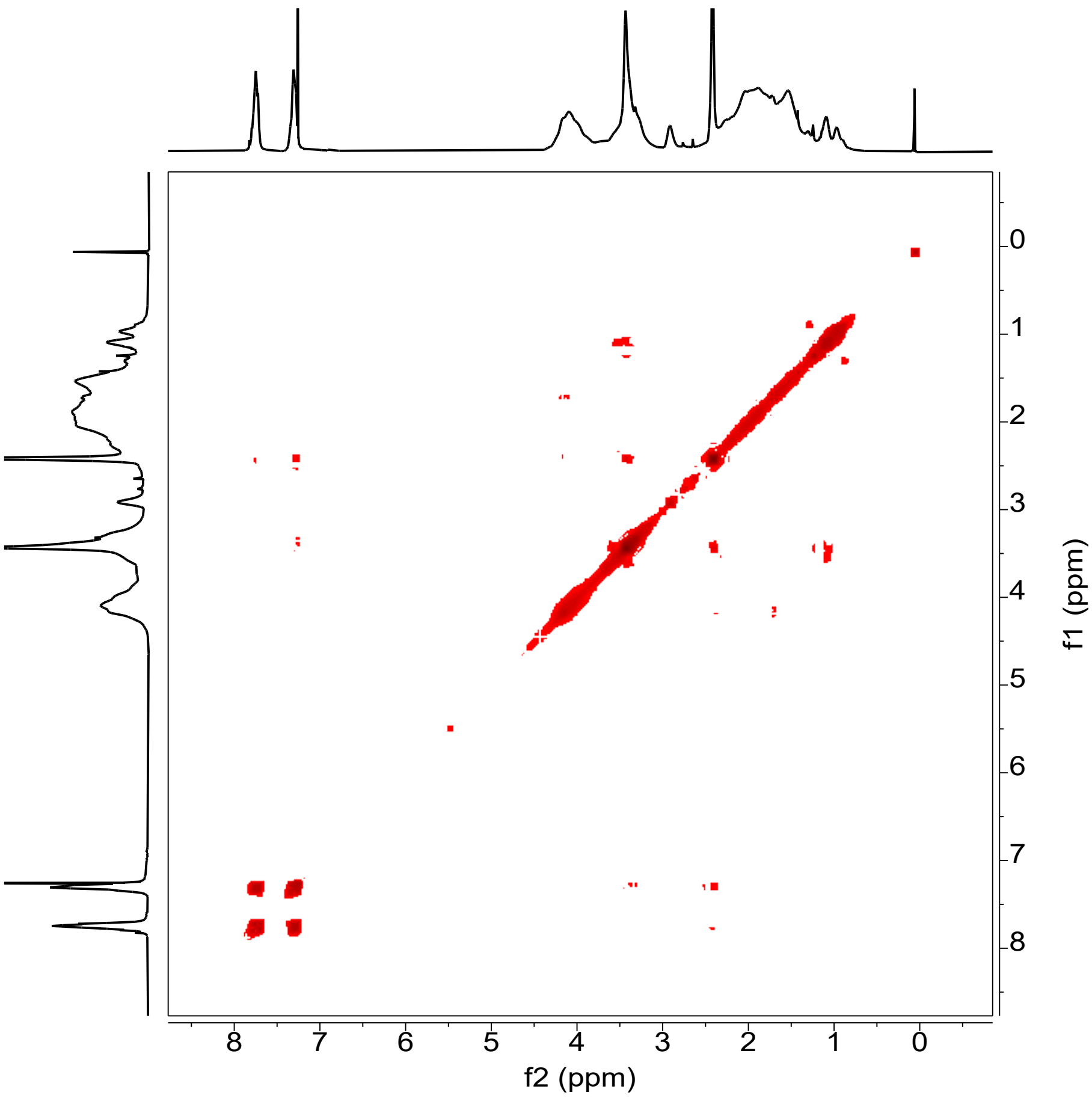

Figure S38. ^{1}H-^{1}H COSY NMR spectrum of **P8** recorded in $CDCl_3$.

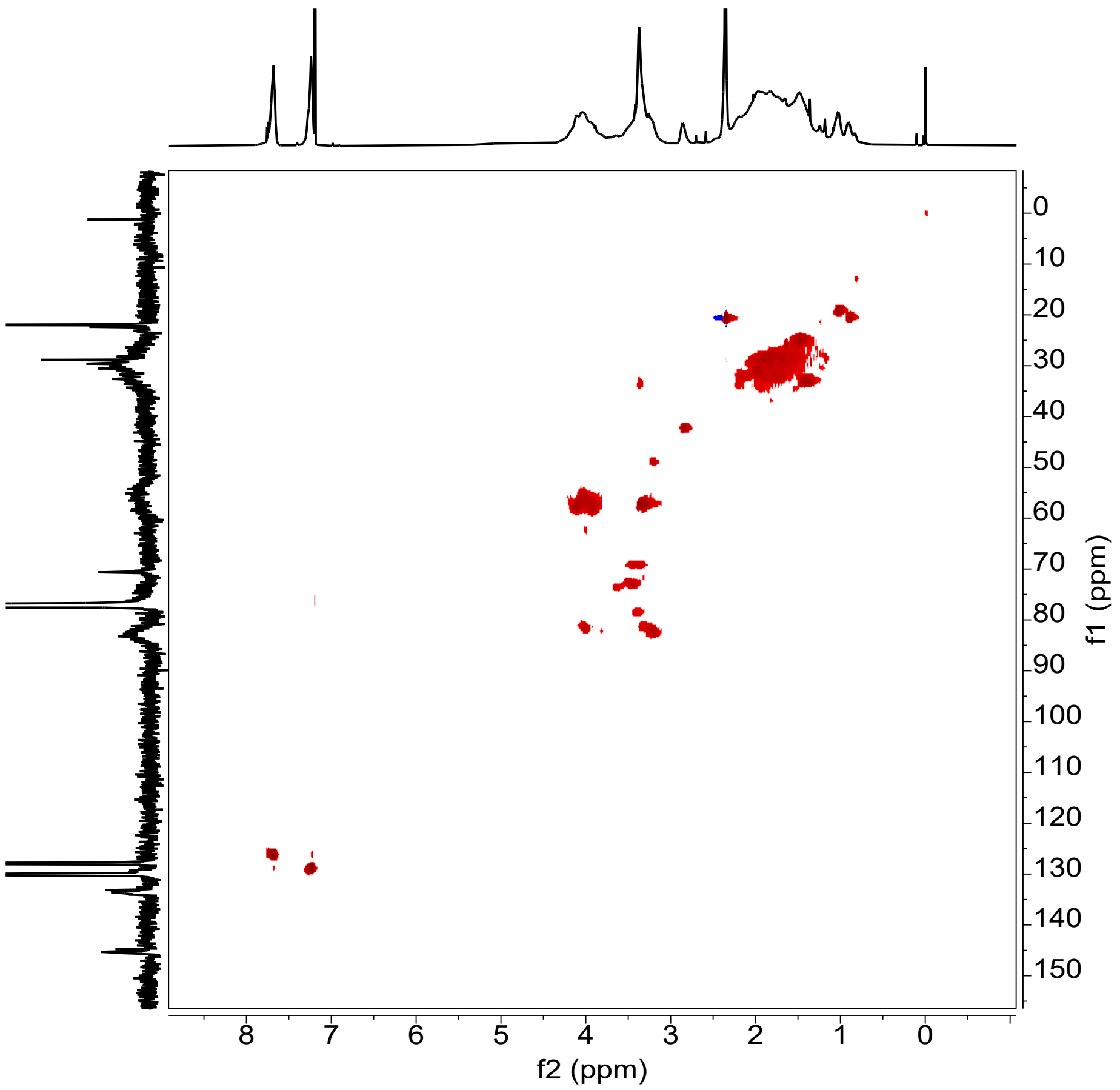

Figure S39. ^{1}H-^{13}C HSQC NMR spectrum of **P8** recorded in $CDCl_3$.

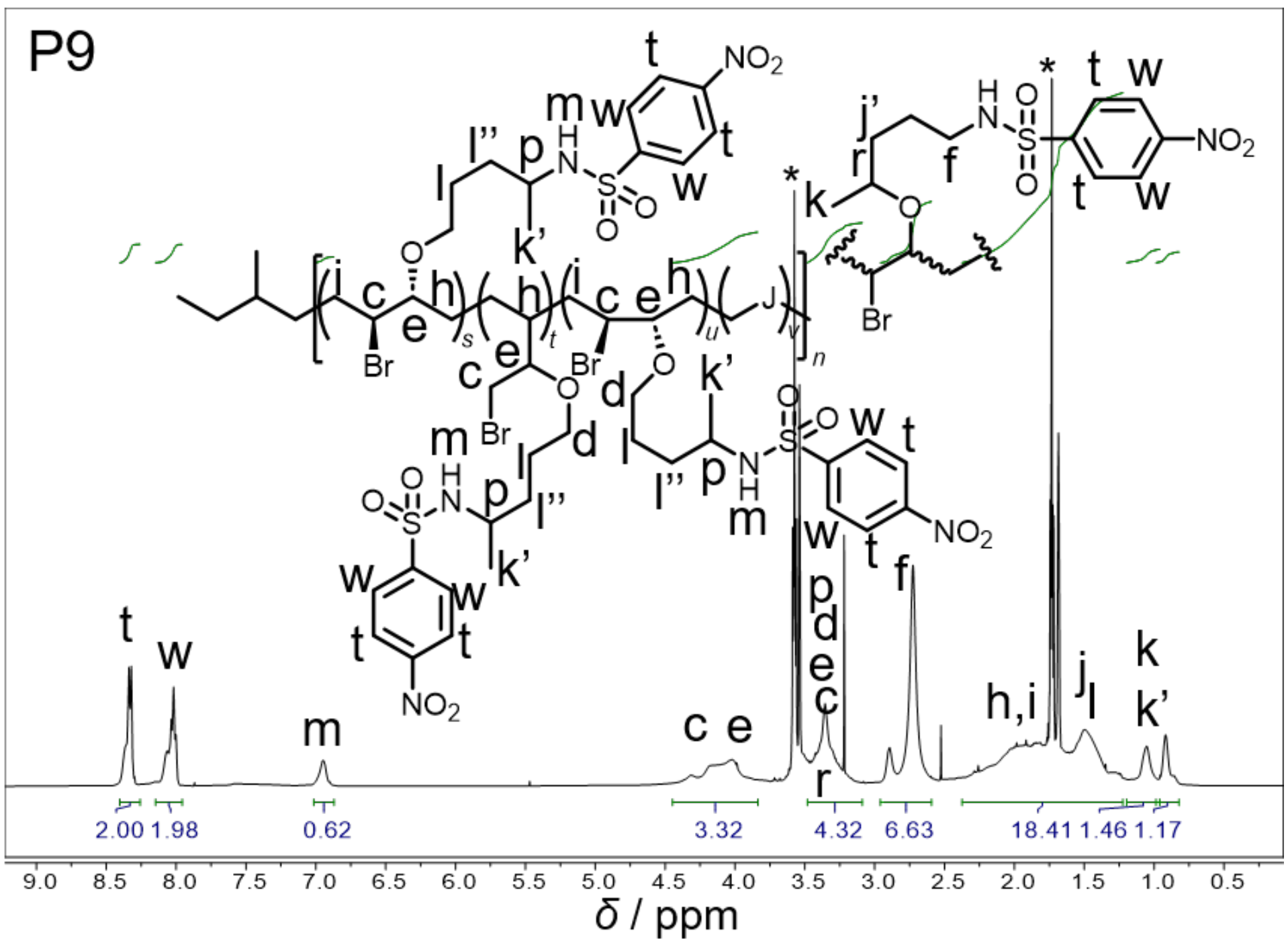

Figure S40. ^{1}H NMR spectrum of **P9** recorded in THF-*d8* at 300 MHz. The residual solvent signal is marked with an asterisk.

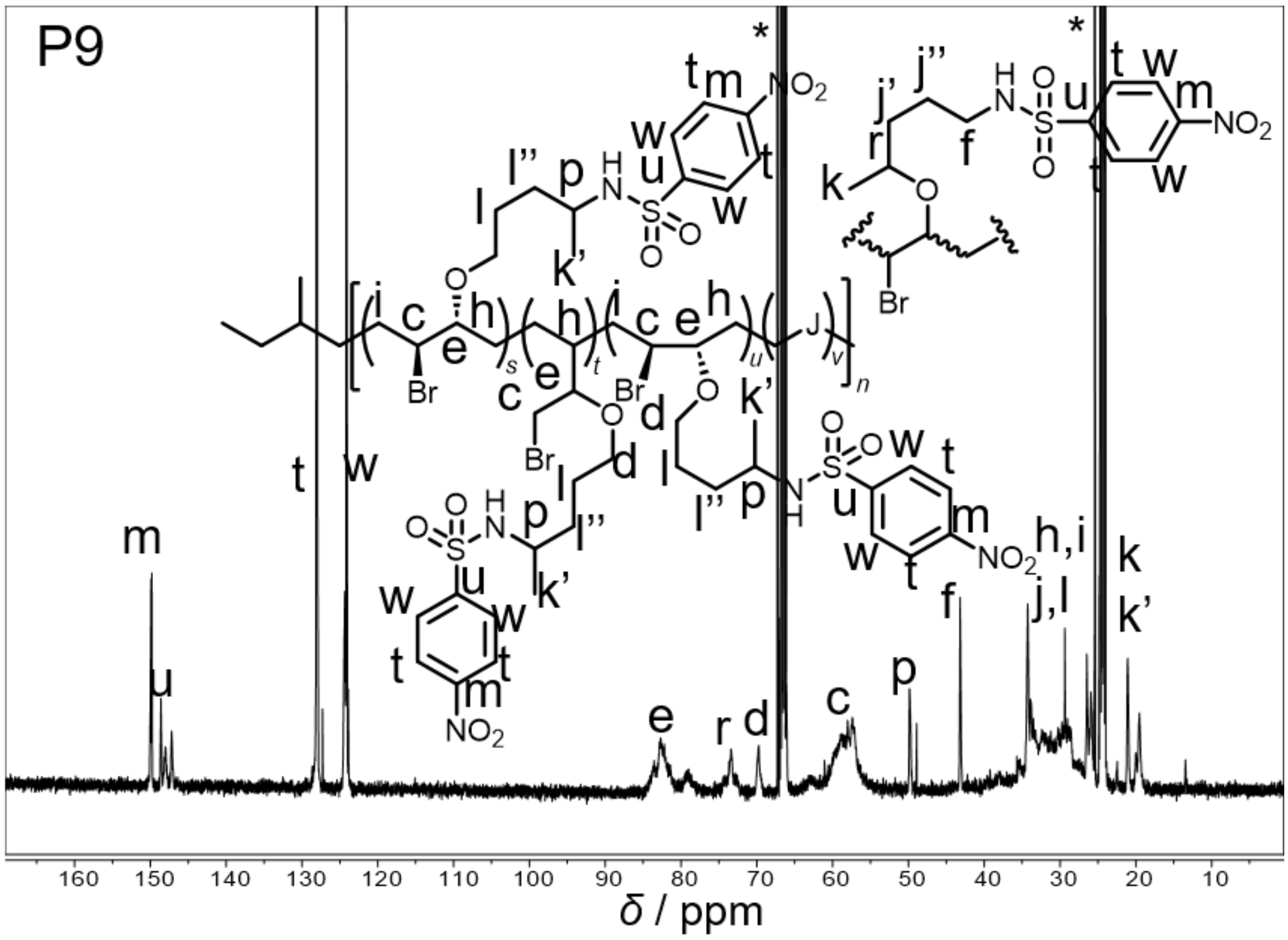

Figure S41. ^{13}C NMR spectrum of **P9** recorded in THF-*d8* at 75 MHz. The residual solvent signal is marked with an asterisk.

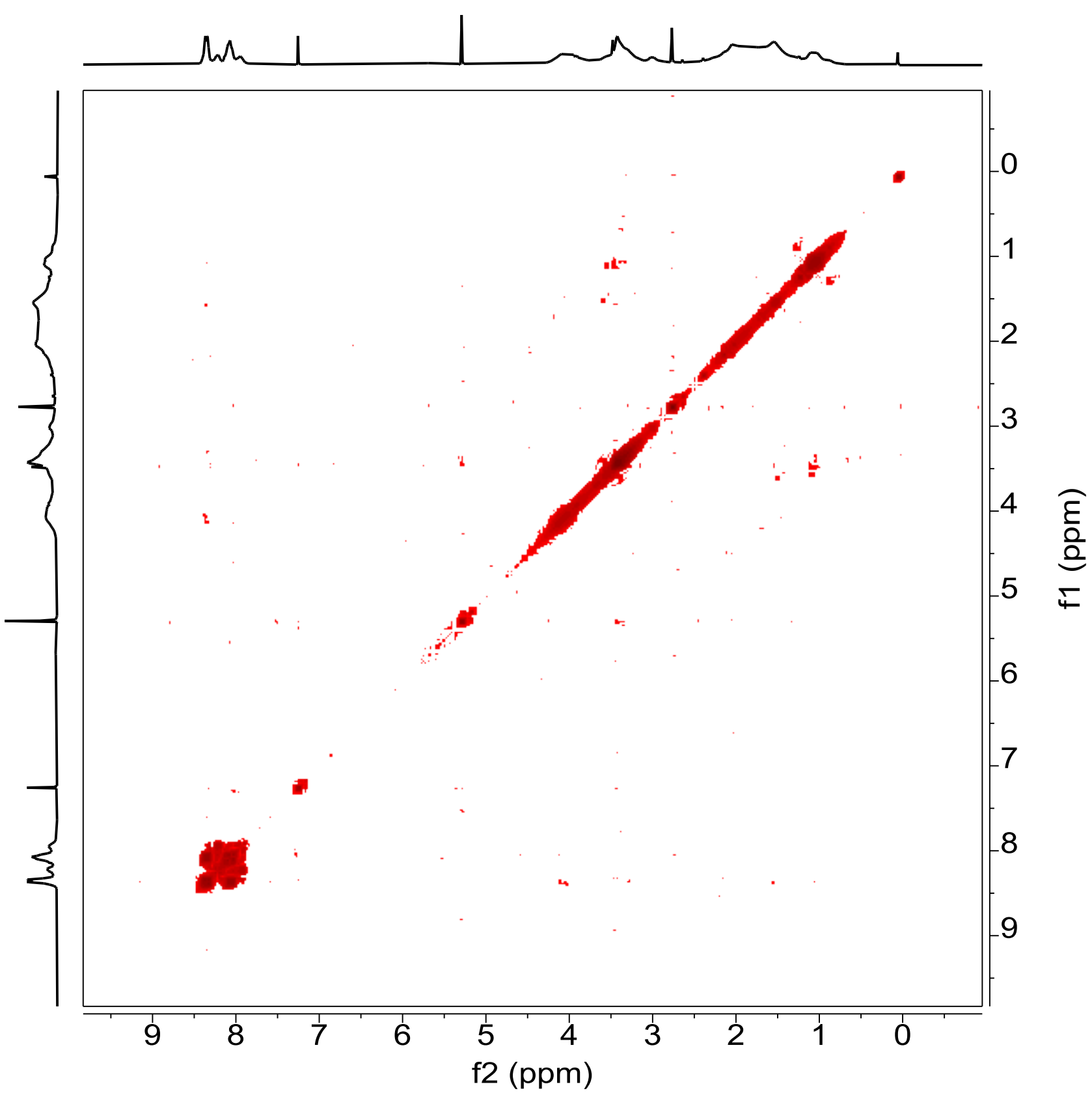

Figure S42. ^{1}H-^{1}H COSY NMR spectrum of **P9** recorded in THF-*d8*.

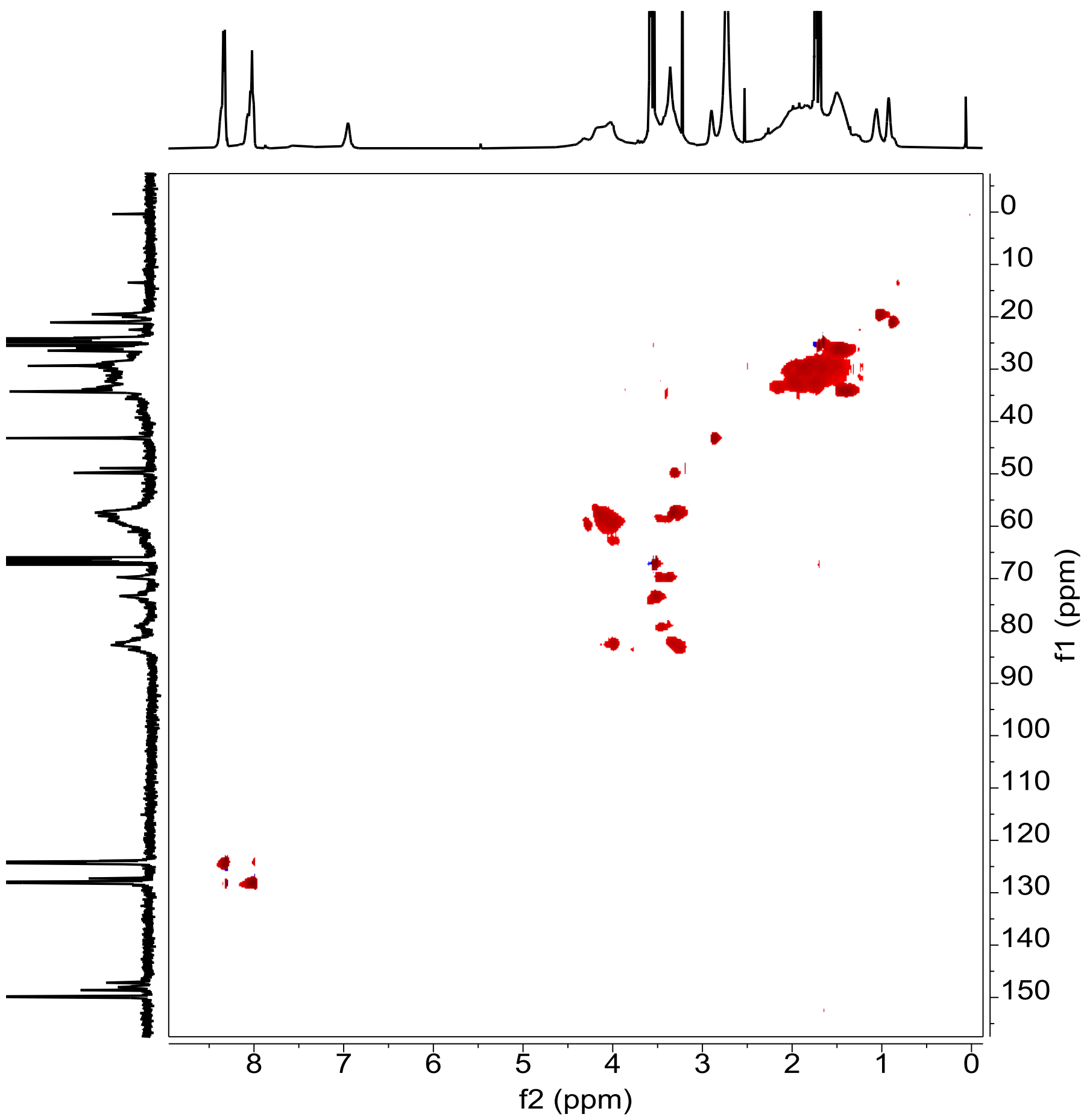

Figure S43. ^{1}H-^{13}C HSQC NMR spectrum of **P9** recorded in THF-d8.

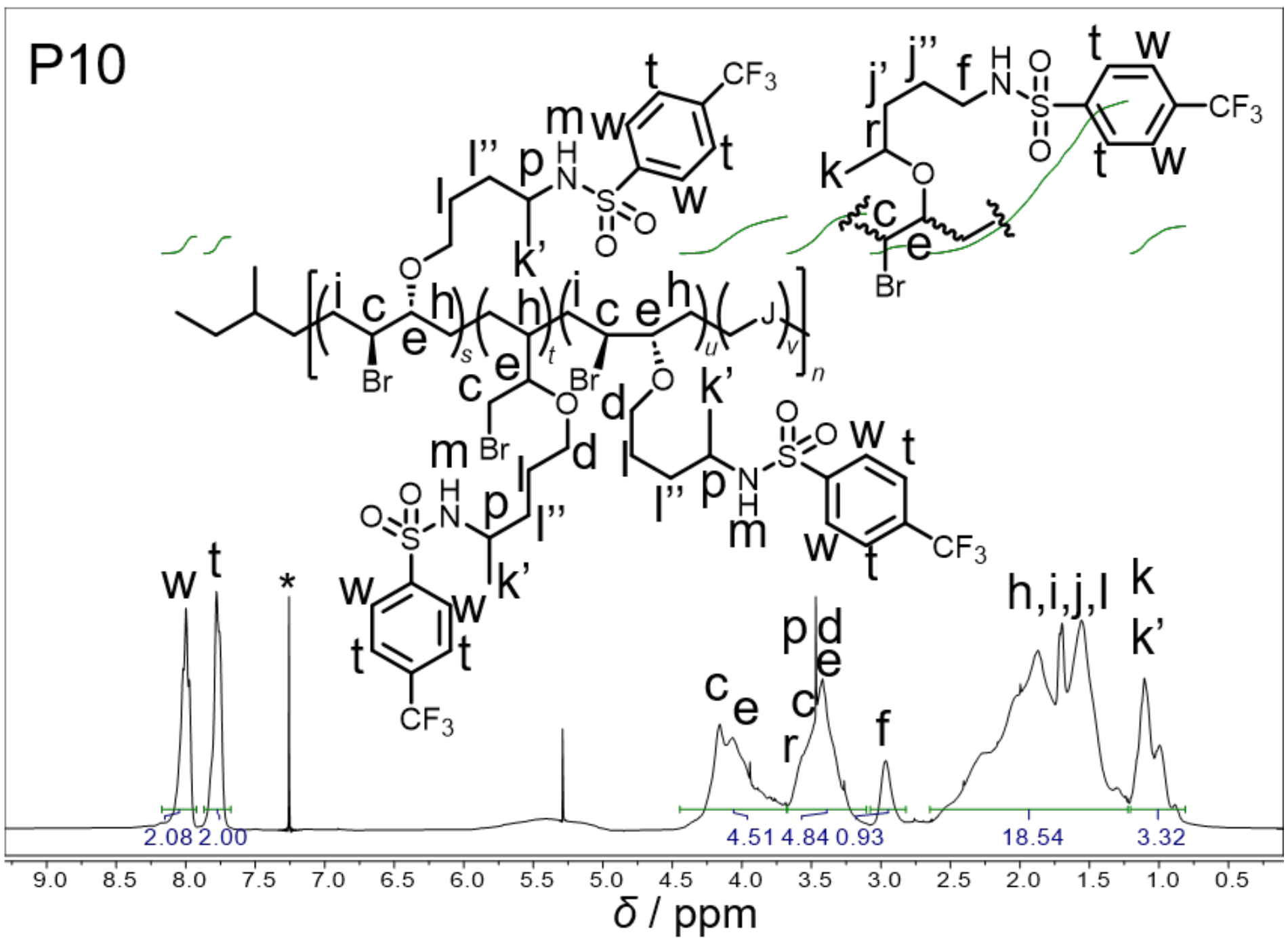

Figure S44. ^{1}H NMR spectrum of **P10** recorded in $CDCl_3$ at 300 MHz. The residual solvent signal is marked with an asterisk.

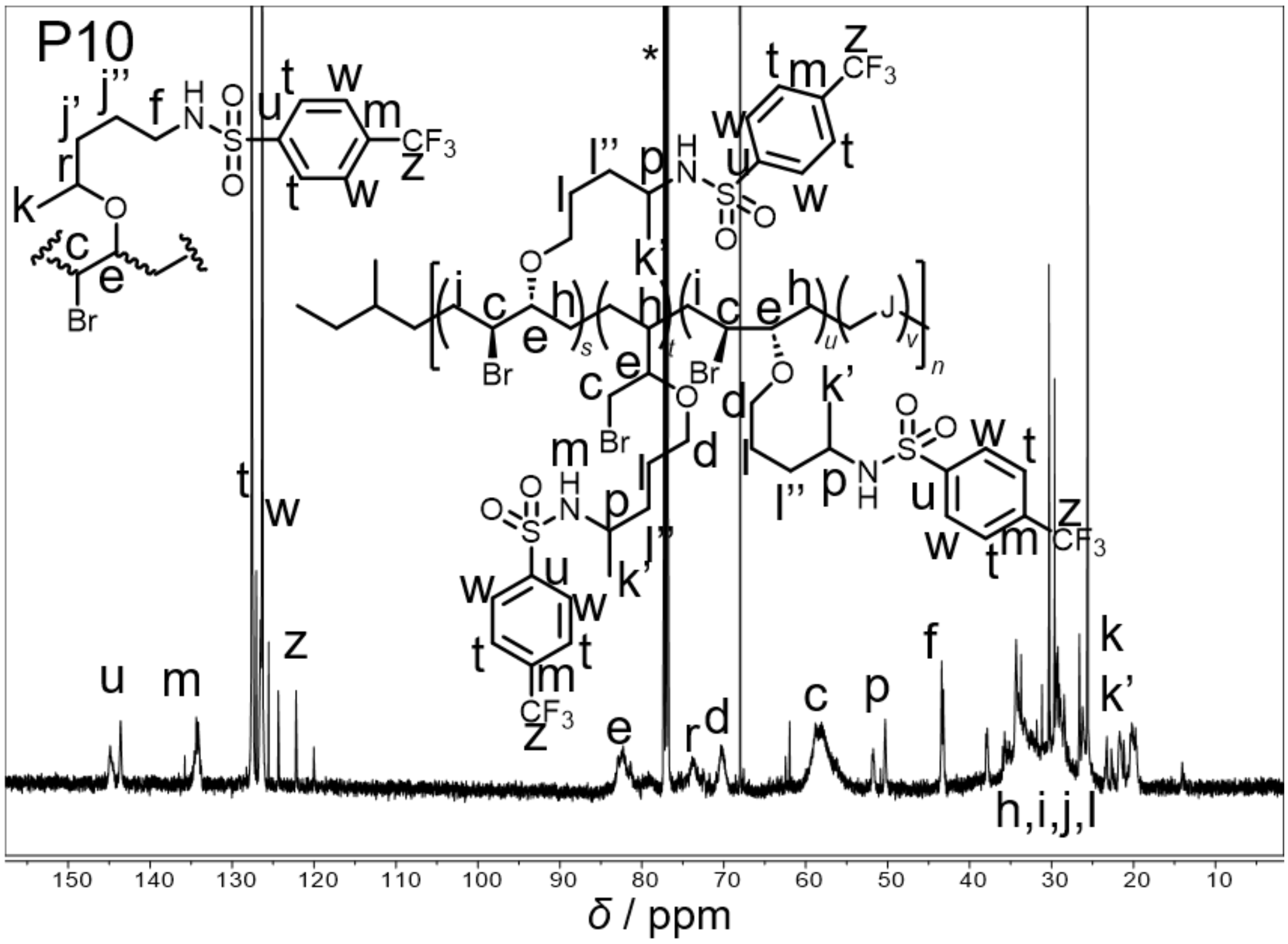

Figure S45. ^{13}C NMR spectrum of **P10** recorded in $CDCl_3$ at 75 MHz. The residual solvent signal is marked with an asterisk.

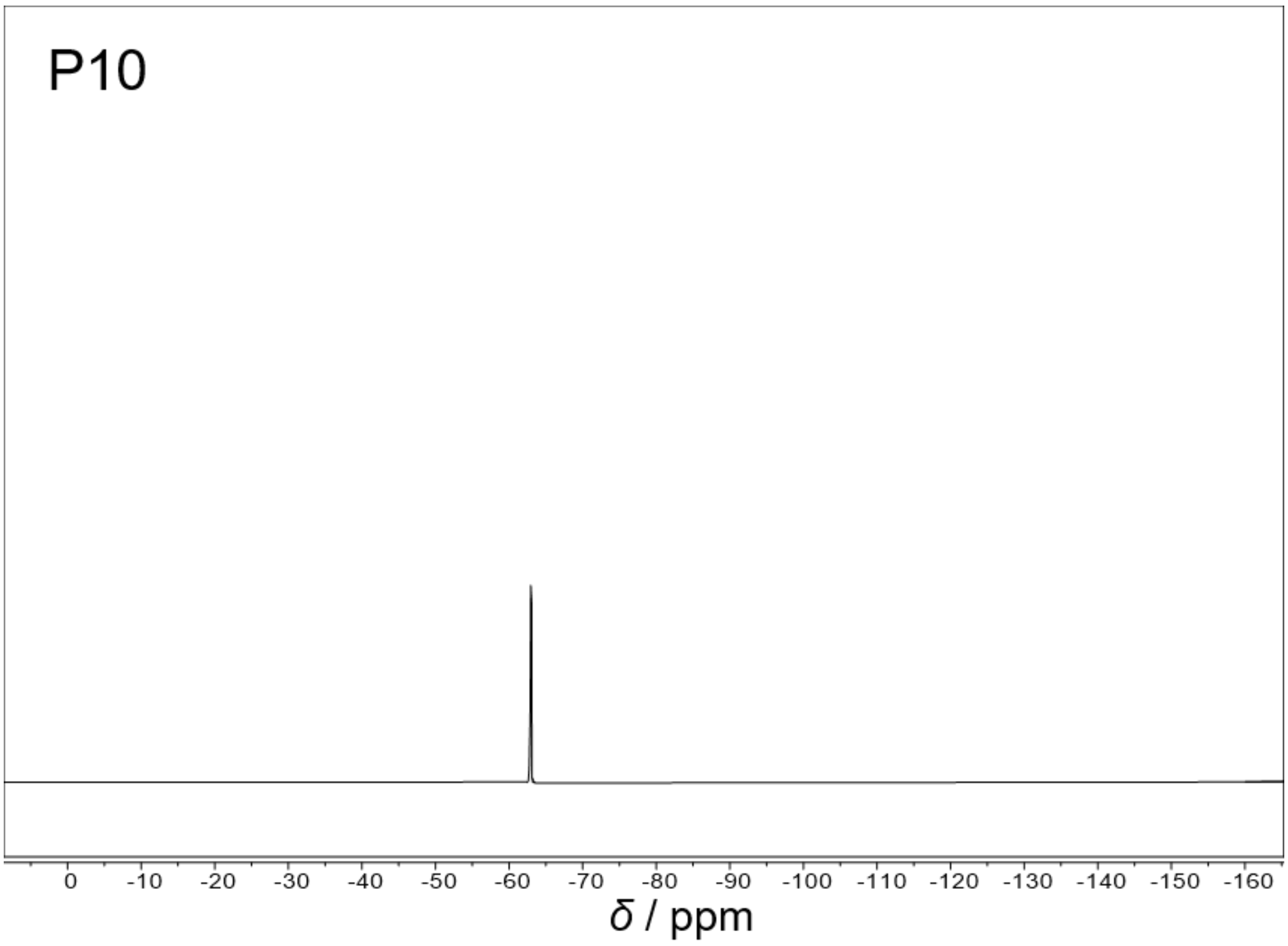

Figure S46. ^{19}F NMR spectrum of **P10** recorded in $CDCl_3$ at 282 MHz.

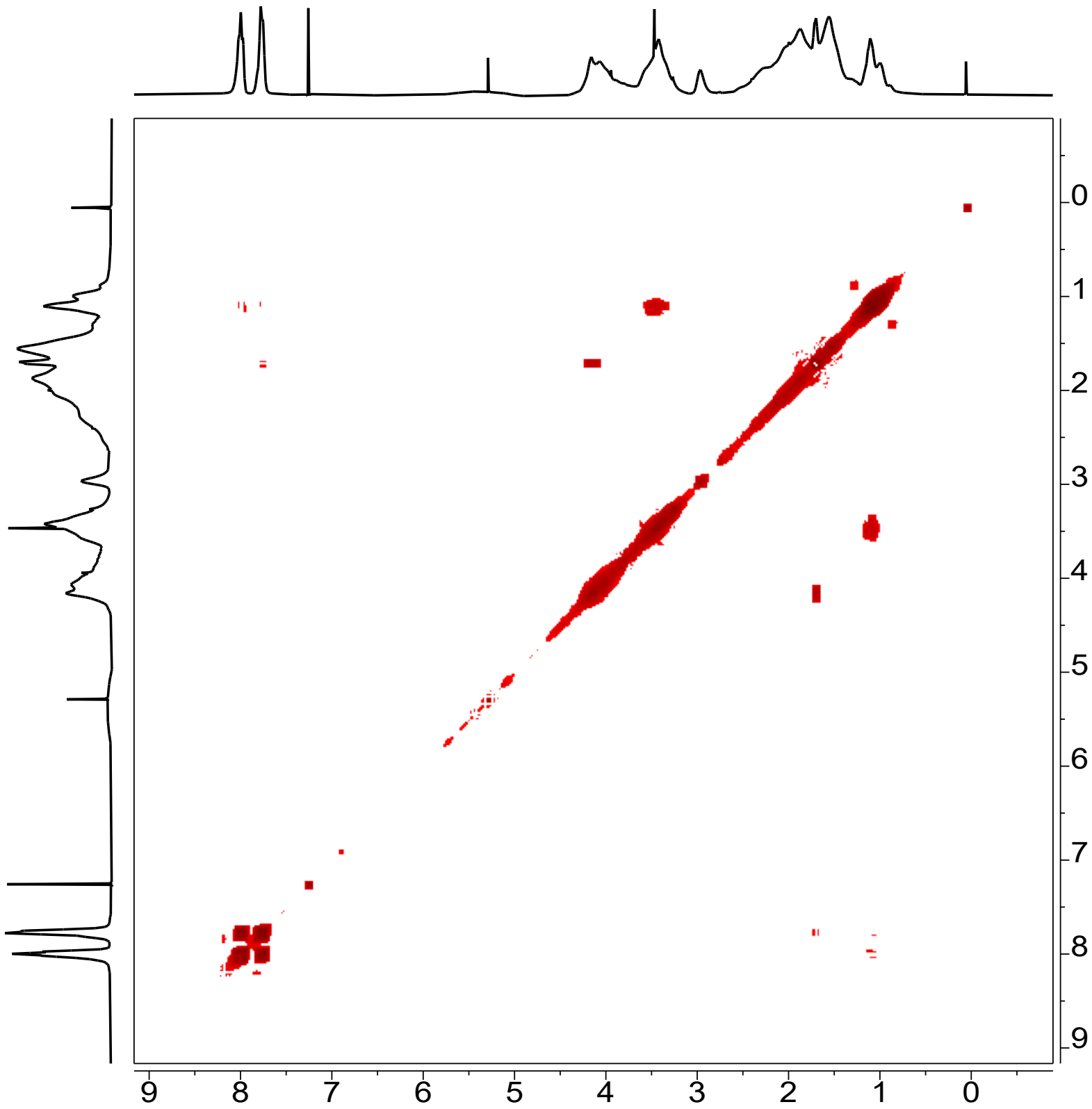

Figure S47. ^{1}H-^{1}H COSY NMR spectrum of **P10** recorded in $CDCl_3$.

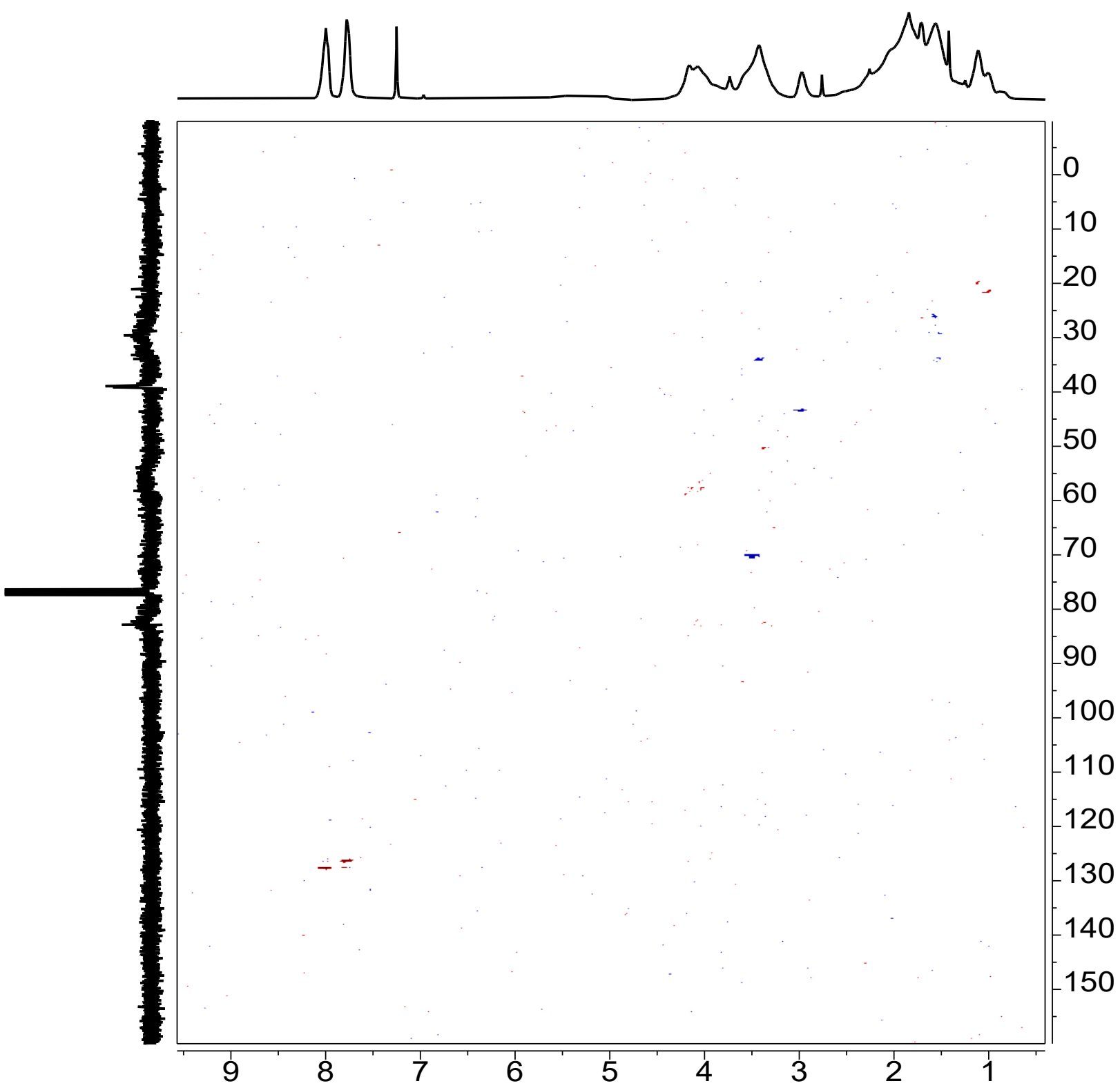

Figure S48. ^{1}H-^{13}C HSQC NMR spectrum of **P10** recorded in $CDCl_3$.

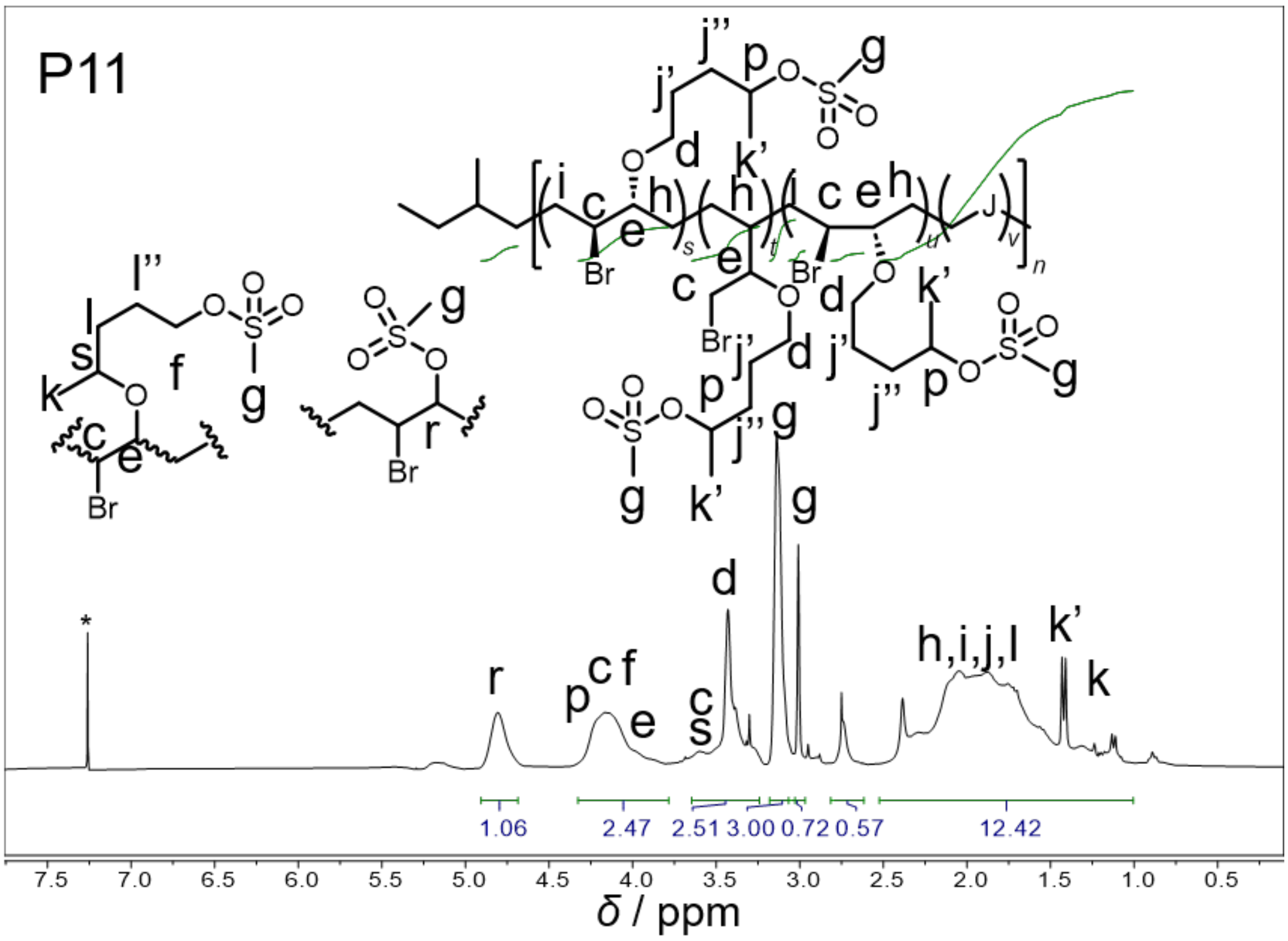

Figure S49. ^{1}H NMR spectrum of **P11** recorded in $CDCl_3$ at 300 MHz. The residual solvent signal is marked with an asterisk.

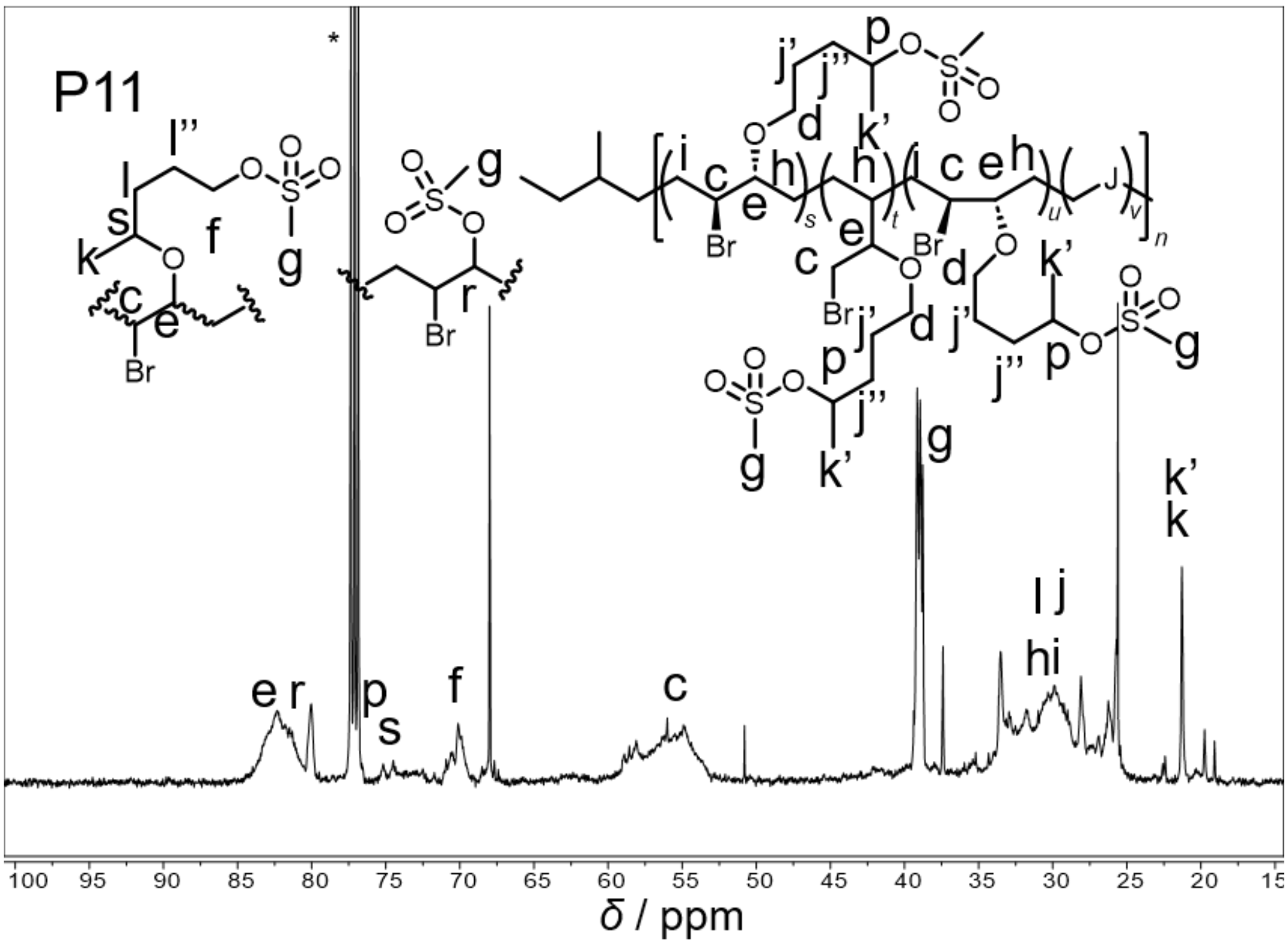

Figure S50. ^{13}C NMR spectrum of **P11** recorded in $CDCl_3$ at 75 MHz. The residual solvent signal is marked with an asterisk.

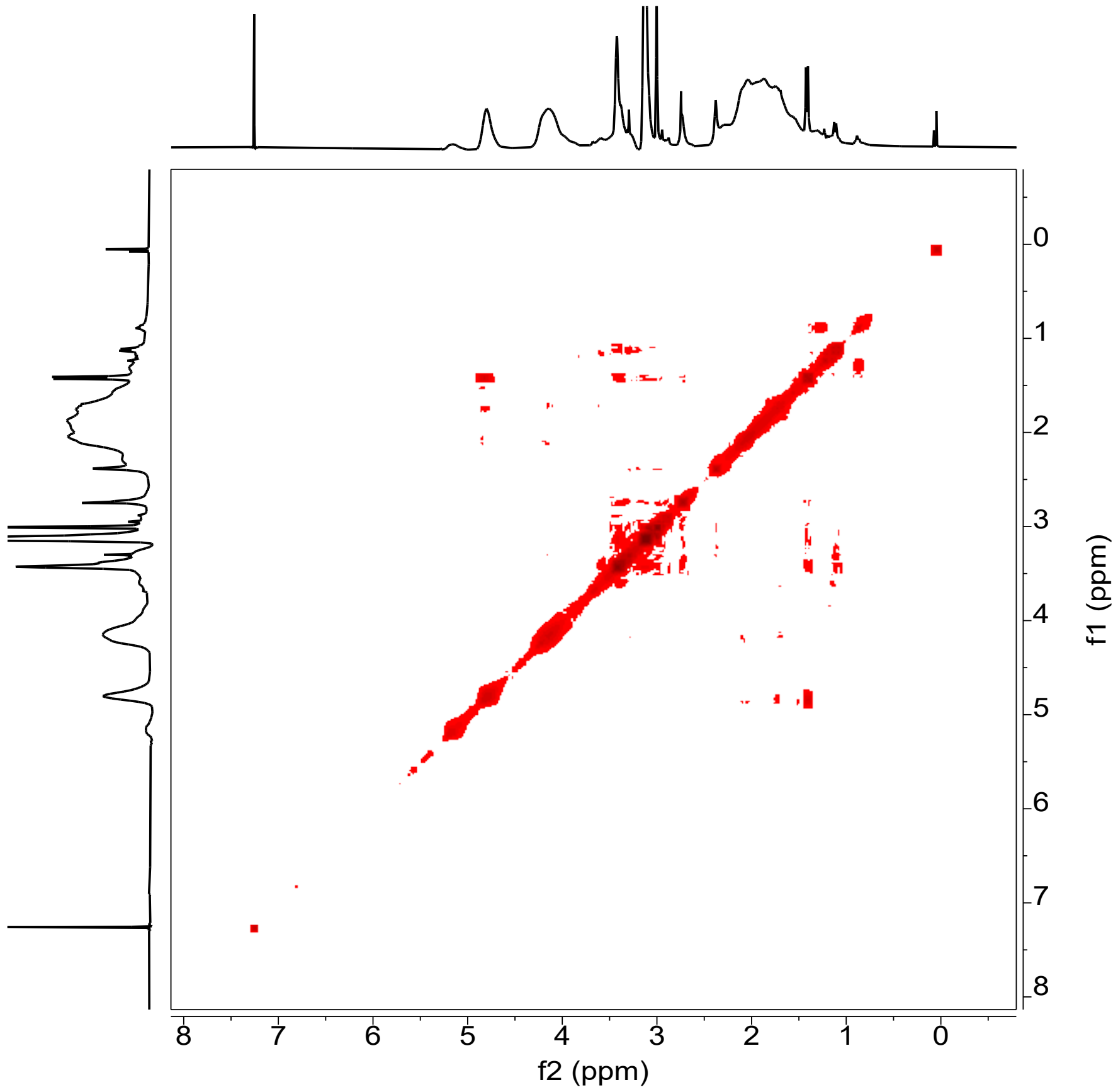

Figure S51. ¹H-¹H COSY NMR spectrum of **P11** recorded in $CDCl_3$.

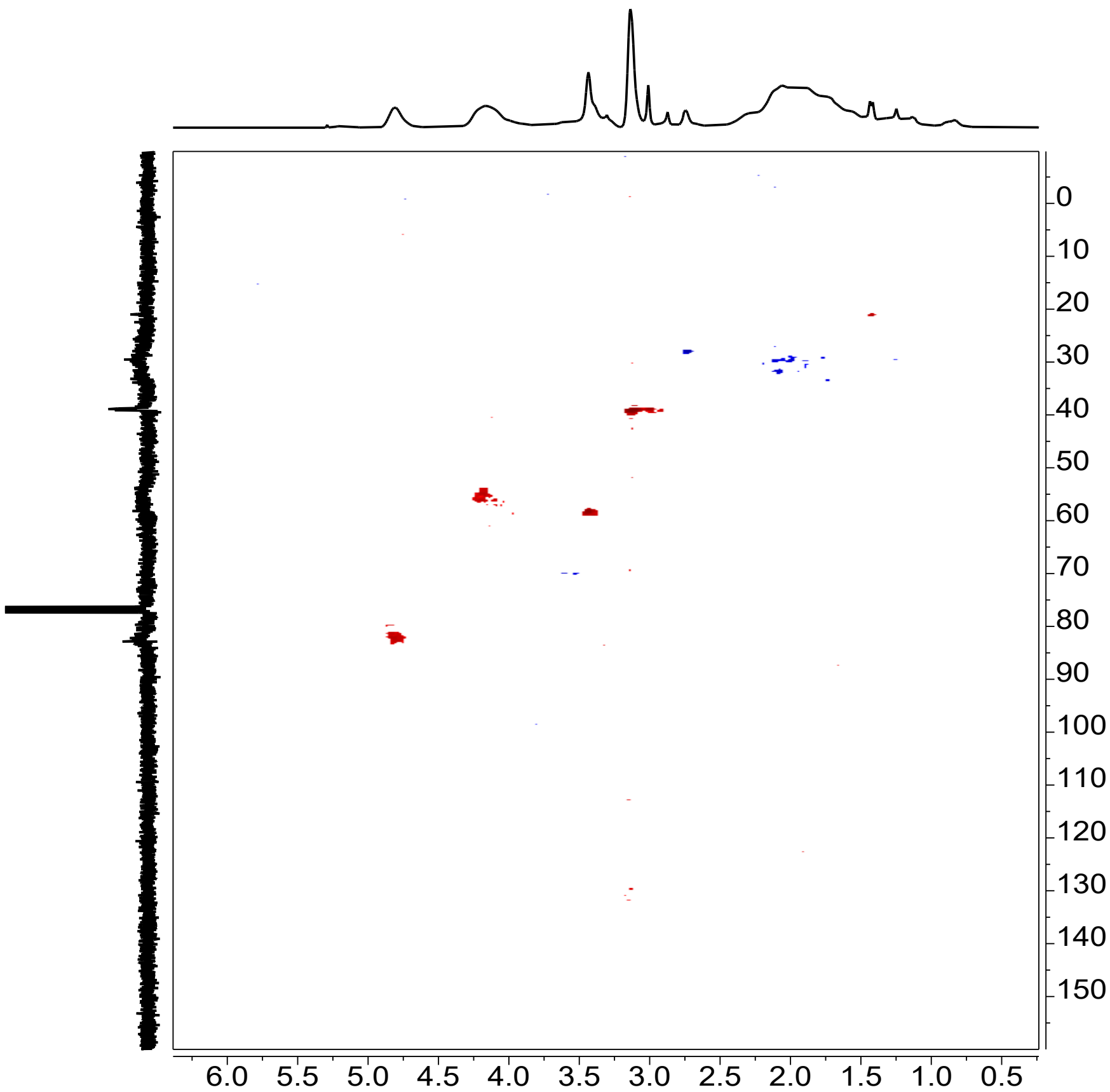

Figure S52. ¹H-¹³C HSQC NMR spectrum of **P13** recorded in $CDCl_3$.

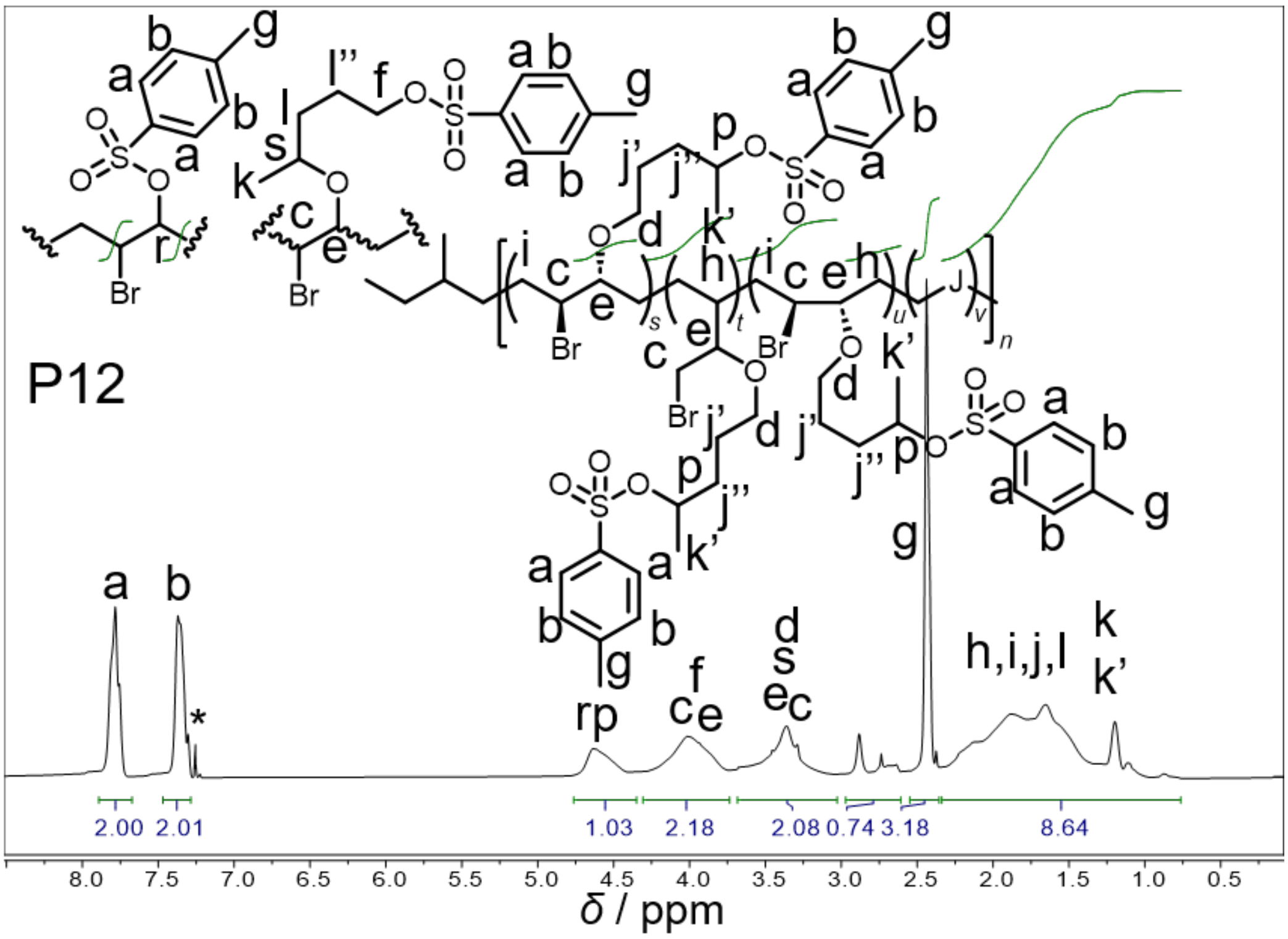

Figure S53. ^{1}H NMR spectrum of **P12** recorded in $CDCl_3$ at 300 MHz. The residual solvent signal is marked with an asterisk.

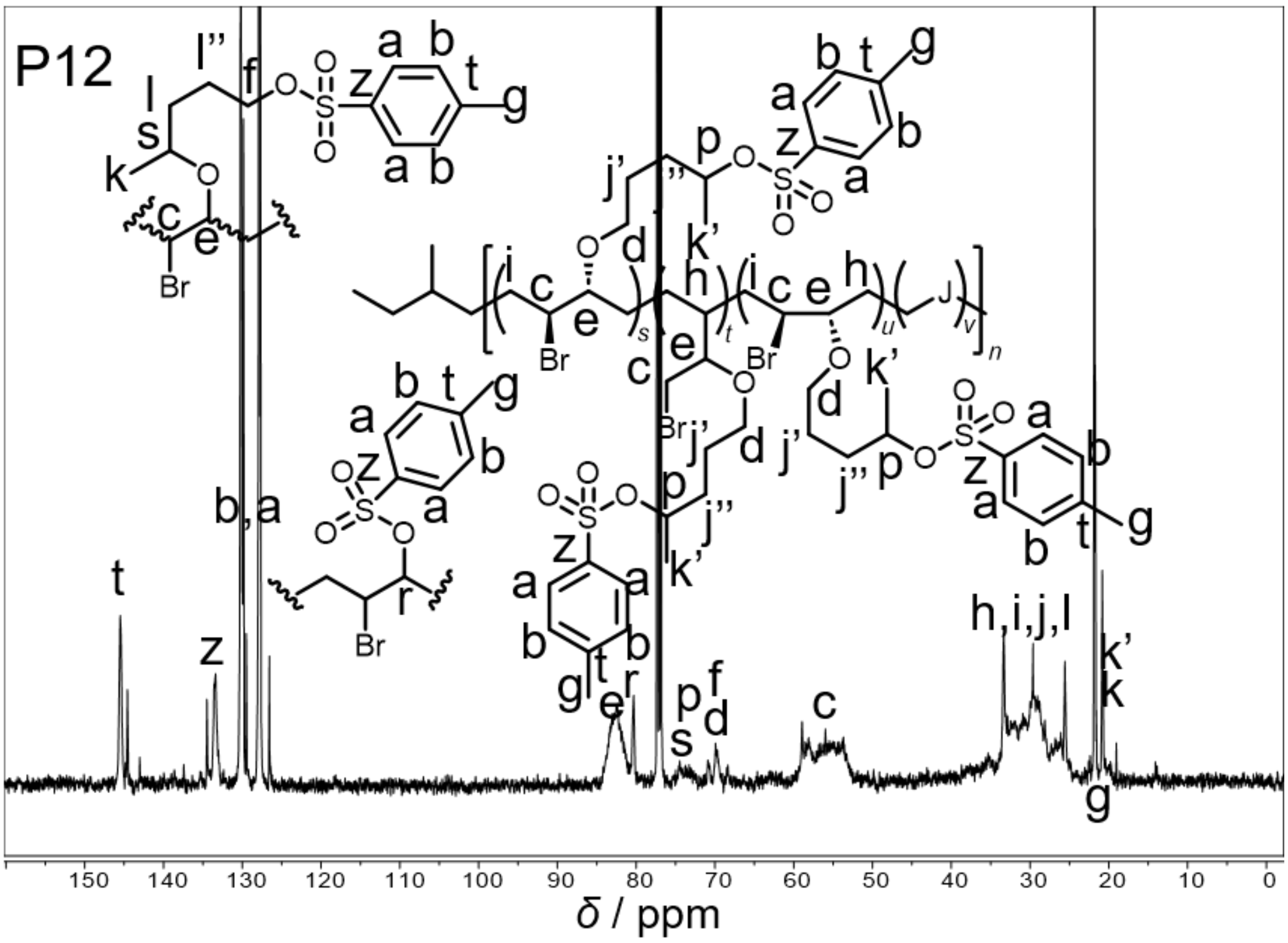

Figure S54. ^{13}C NMR spectrum of **P12** recorded in $CDCl_3$ at 75 MHz. The residual solvent signal is marked with an asterisk.

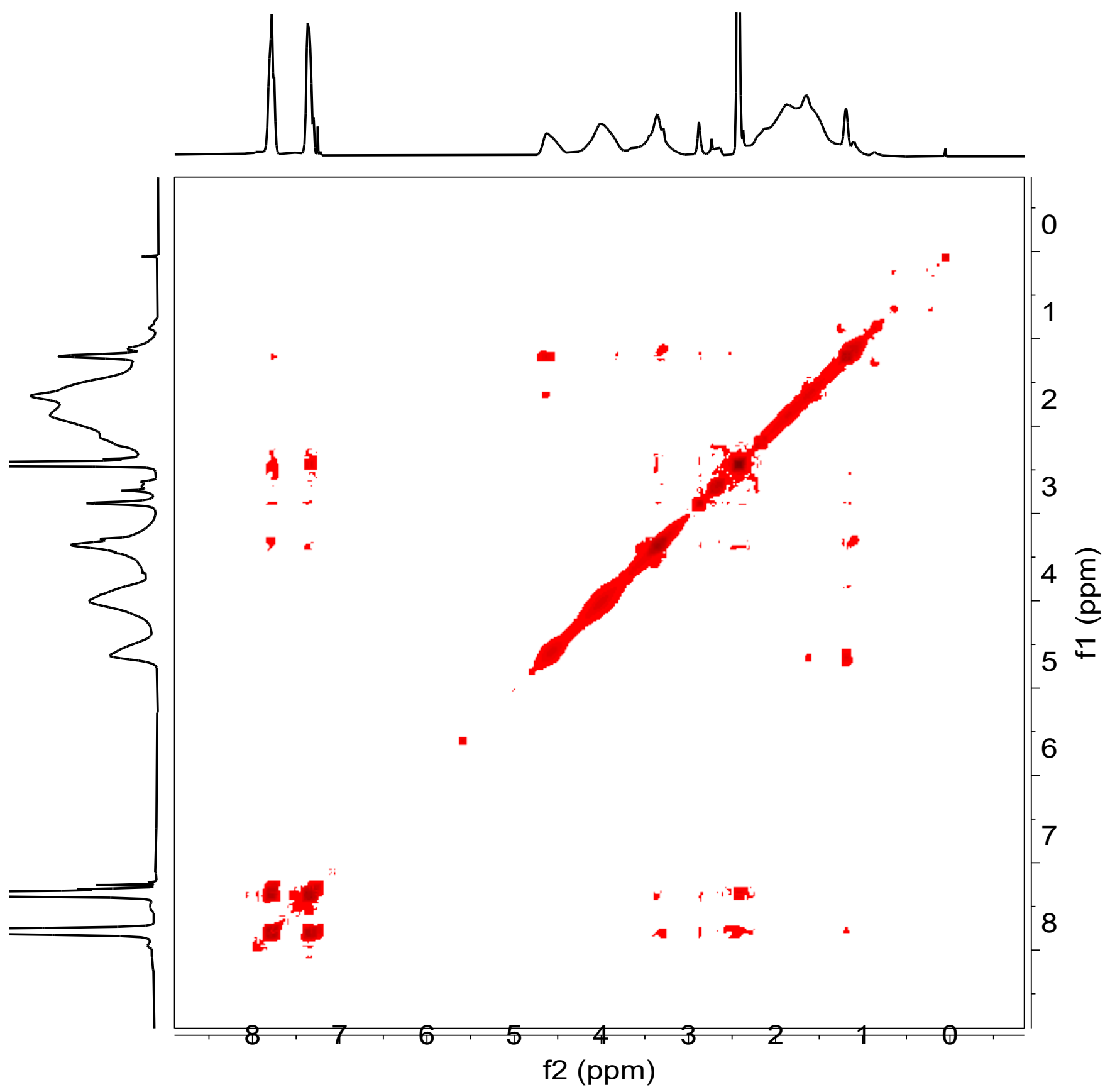

Figure S55. ^1H-^1H COSY NMR spectrum of **P12** recorded in $CDCl_3$.

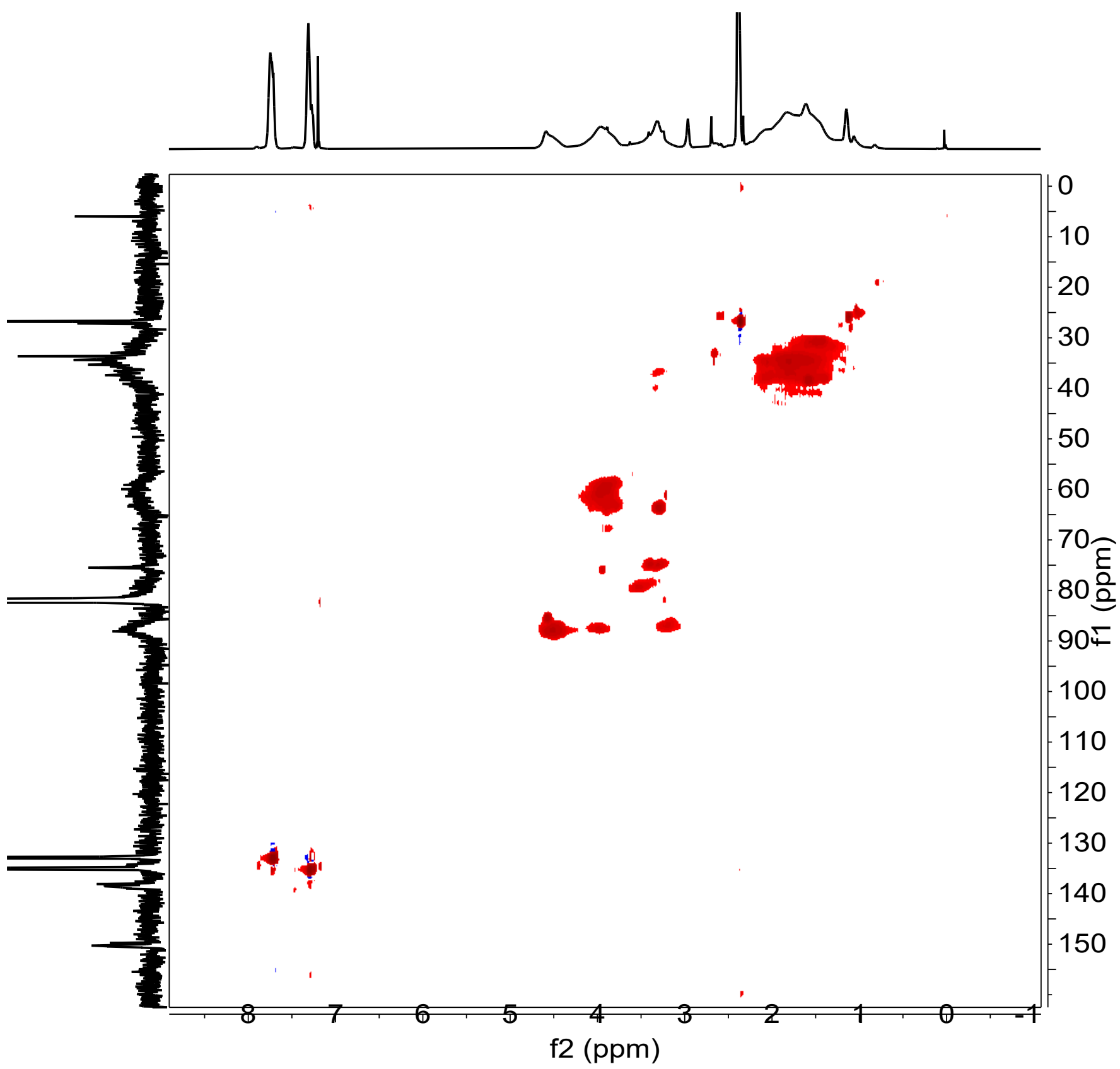

Figure S56. ^1H-^{13}C HSQC NMR spectrum of **P12** recorded in $CDCl_3$.

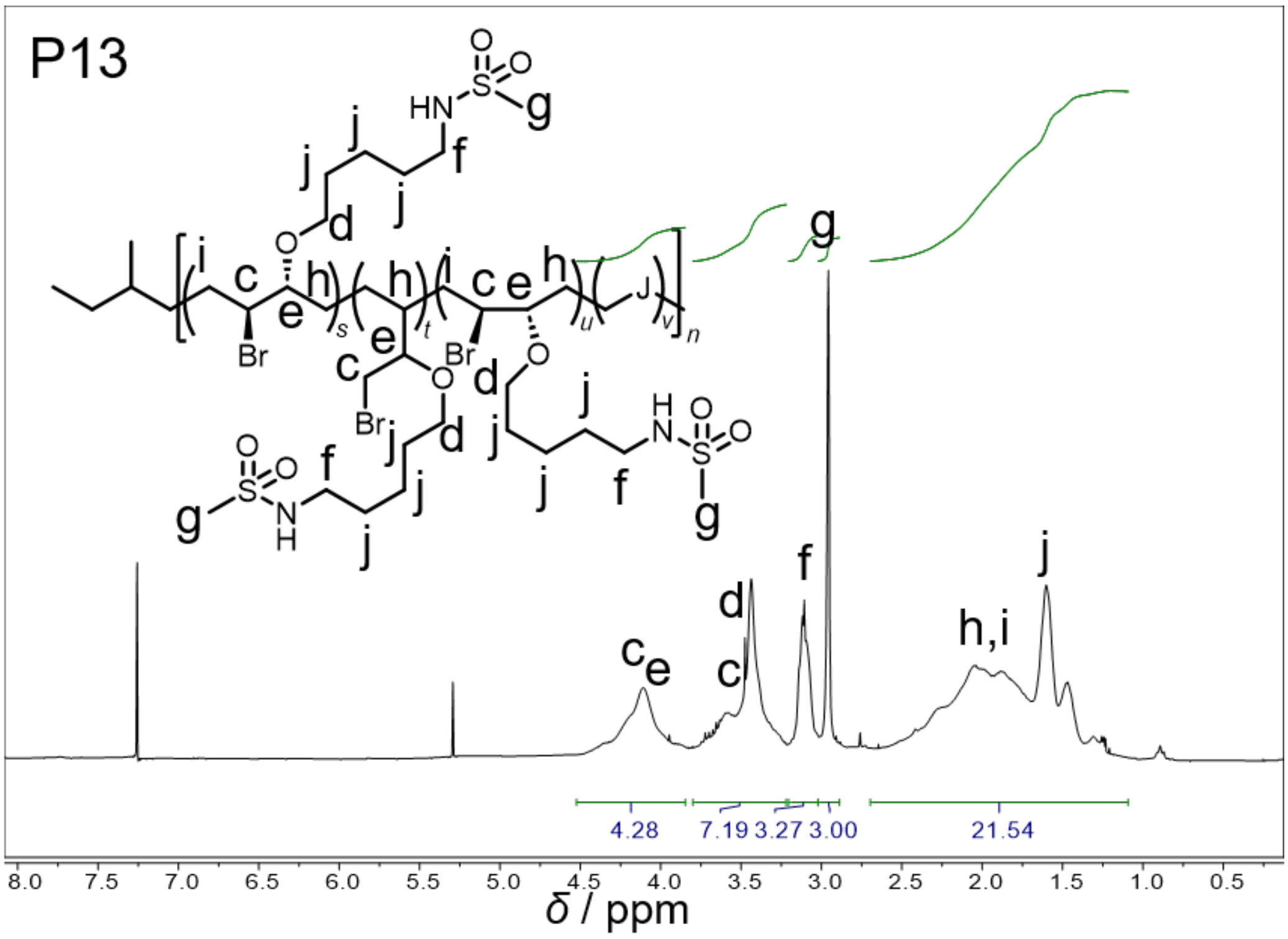

Figure S57. ^{1}H NMR spectrum of **P13** recorded in $CDCl_3$ at 300 MHz. The residual solvent signal is marked with an asterisk.

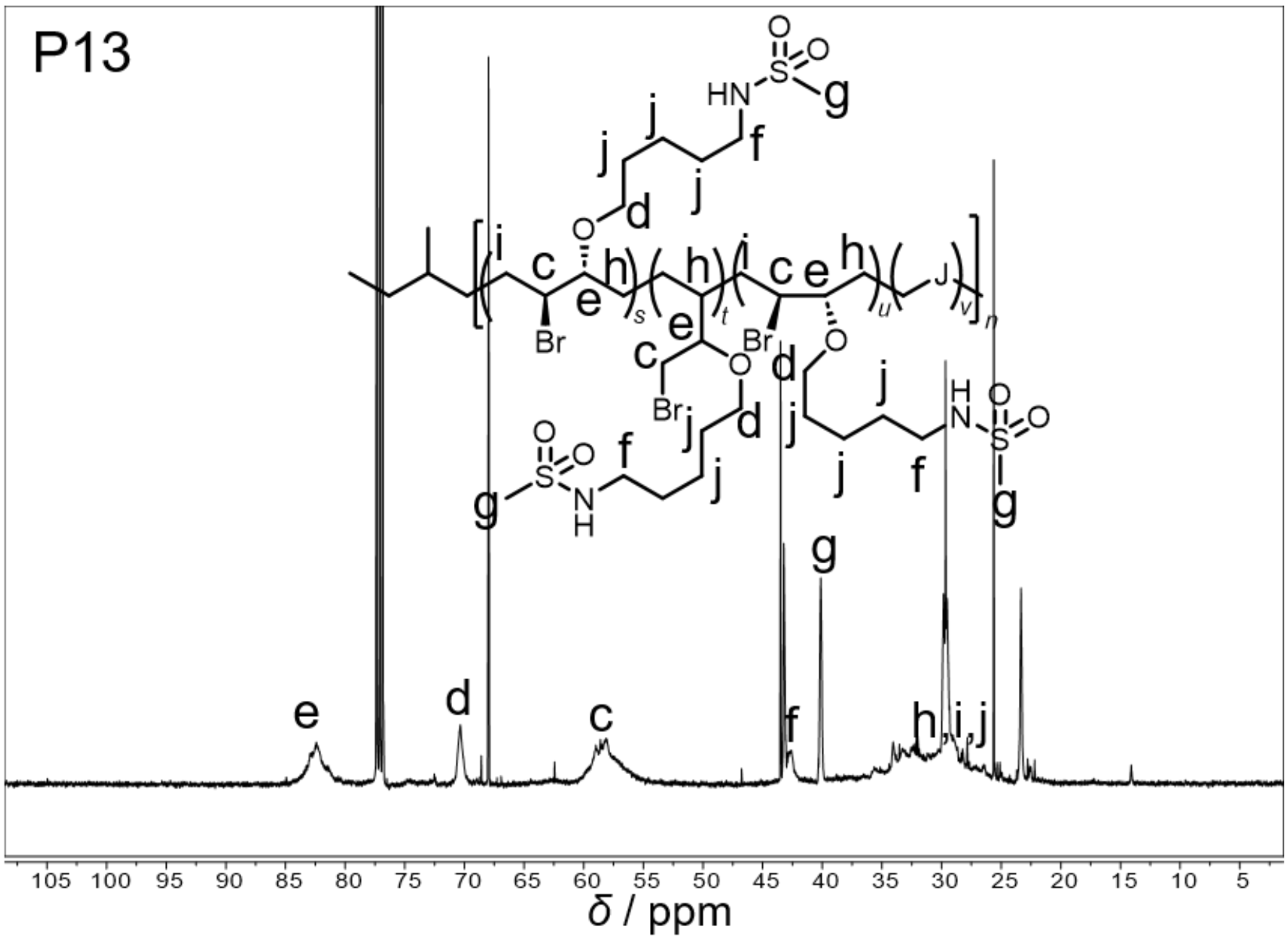

Figure S58. ^{13}C NMR spectrum of **P13** recorded in $CDCl_3$ at 75 MHz. The residual solvent signal is marked with an asterisk.

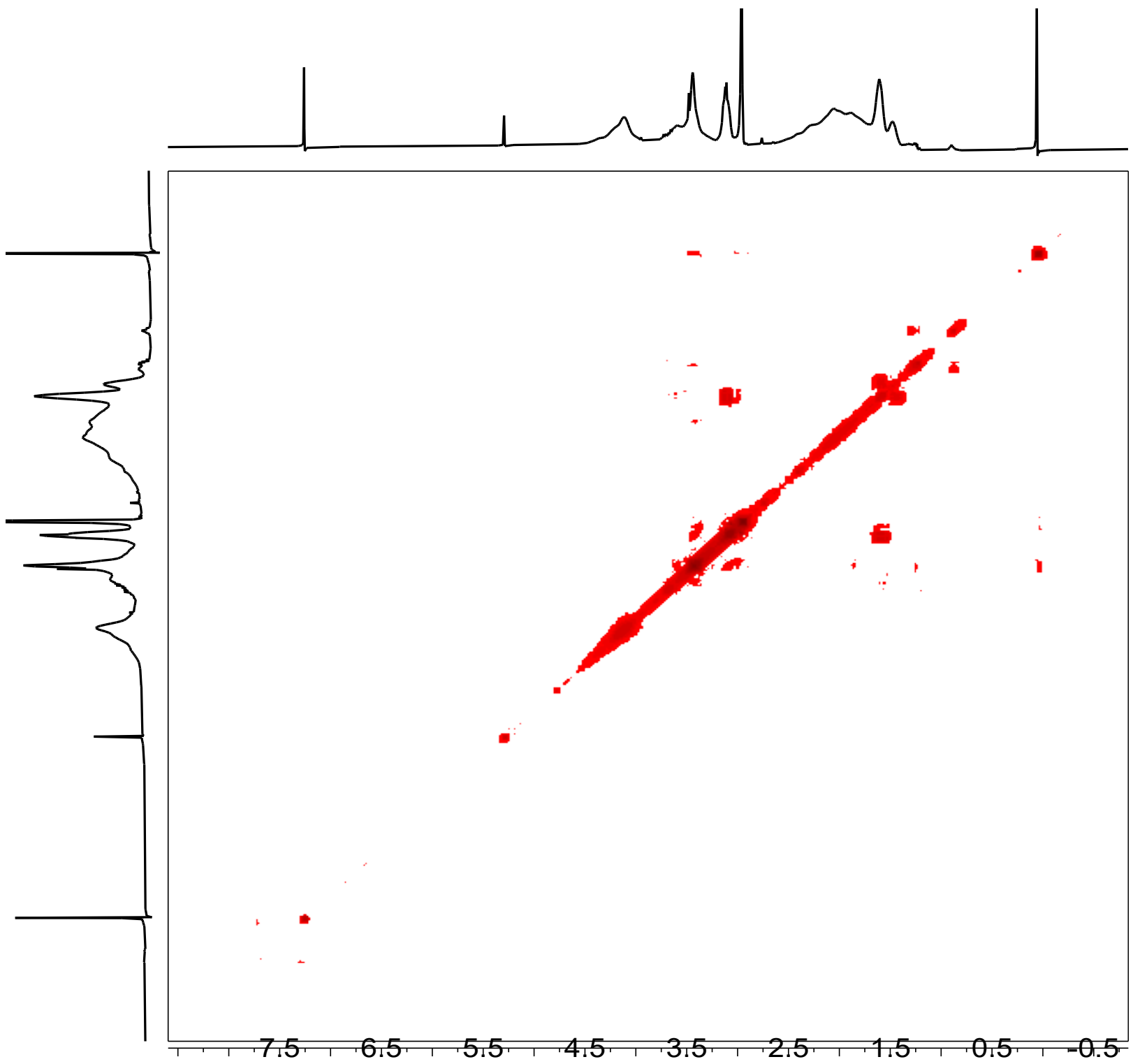

Figure S59. ^1H-^1H COSY NMR spectrum of **P13** recorded in $CDCl_3$.

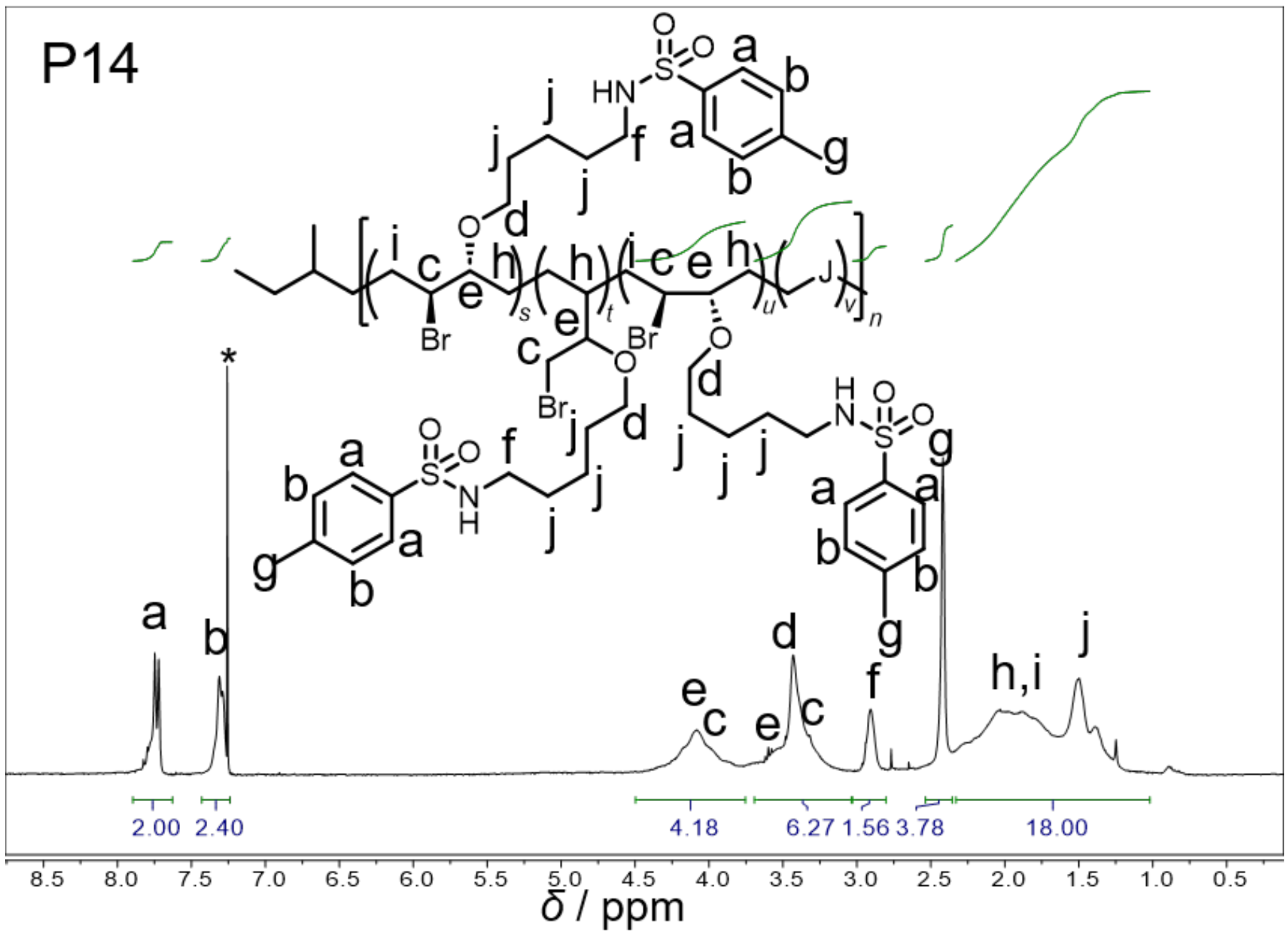

Figure S60. ^1H NMR spectrum of **P14** recorded in $CDCl_3$ at 300 MHz. The residual solvent signal is marked with an asterisk.

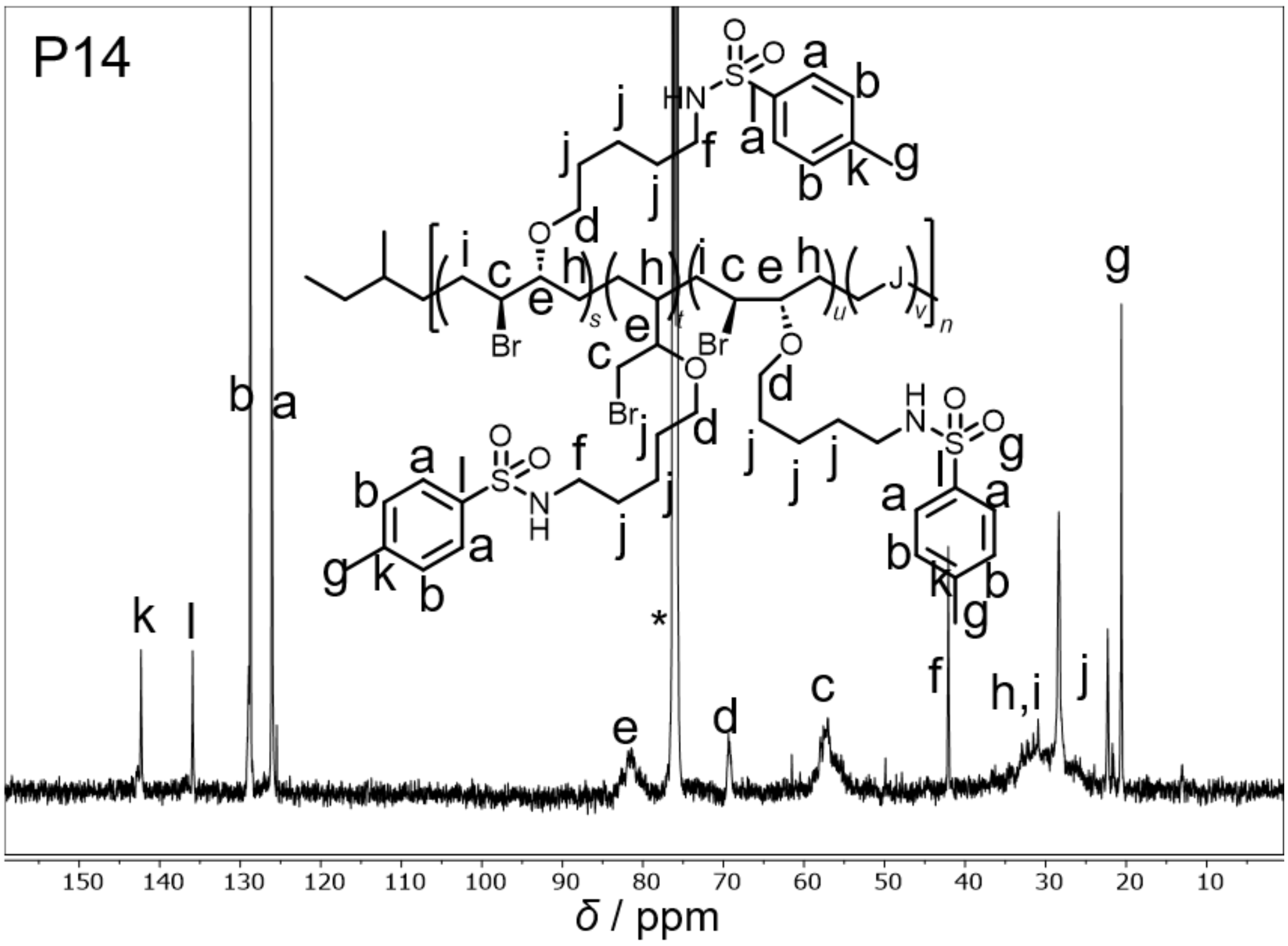

Figure S61. ^{13}C NMR spectrum of **P14** recorded in $CDCl_3$ at 75 MHz. The residual solvent signal is marked with an asterisk.

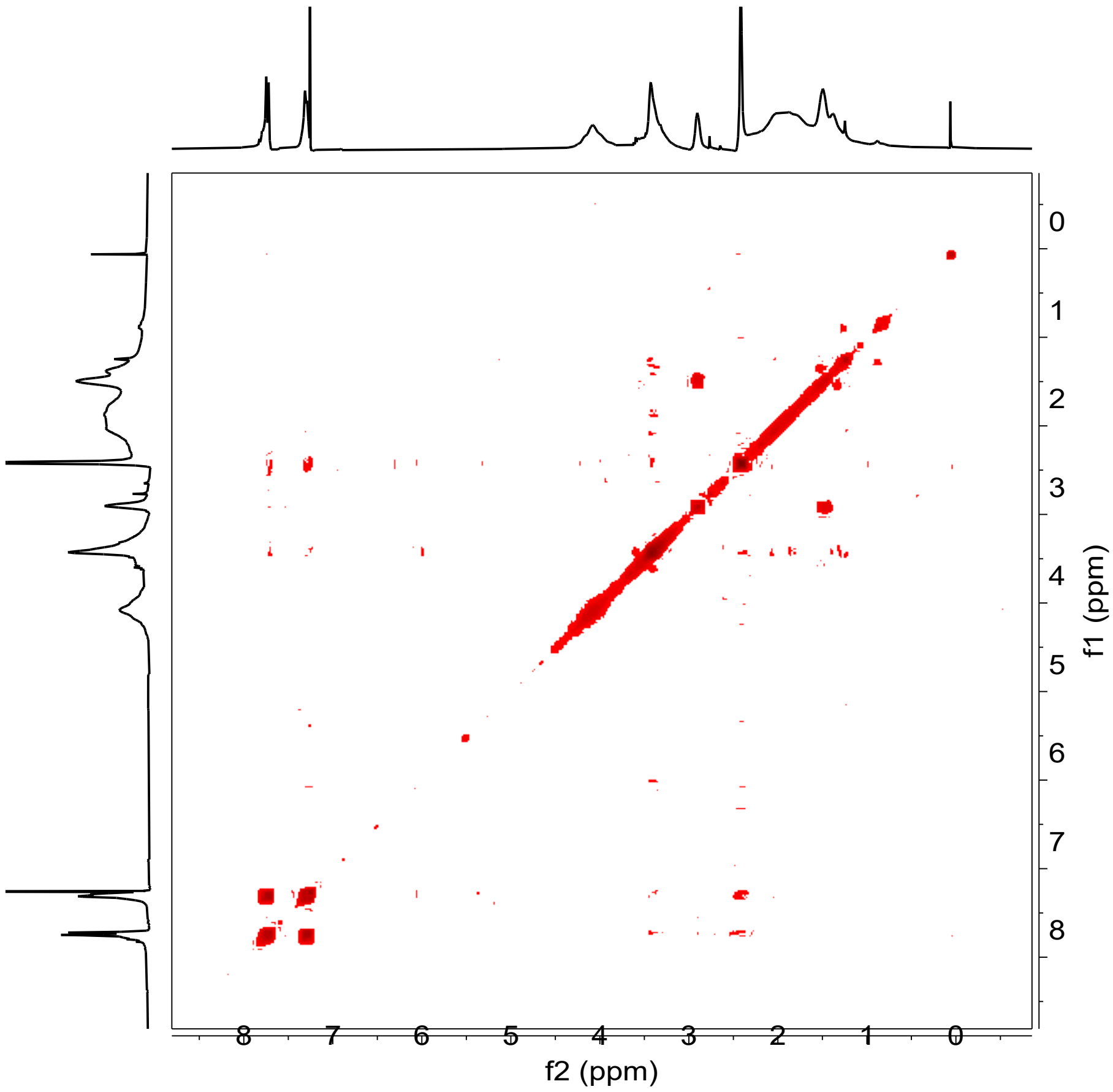

Figure S62. ^{1}H-^{1}H COSY NMR spectrum of **P14** recorded in $CDCl_3$.

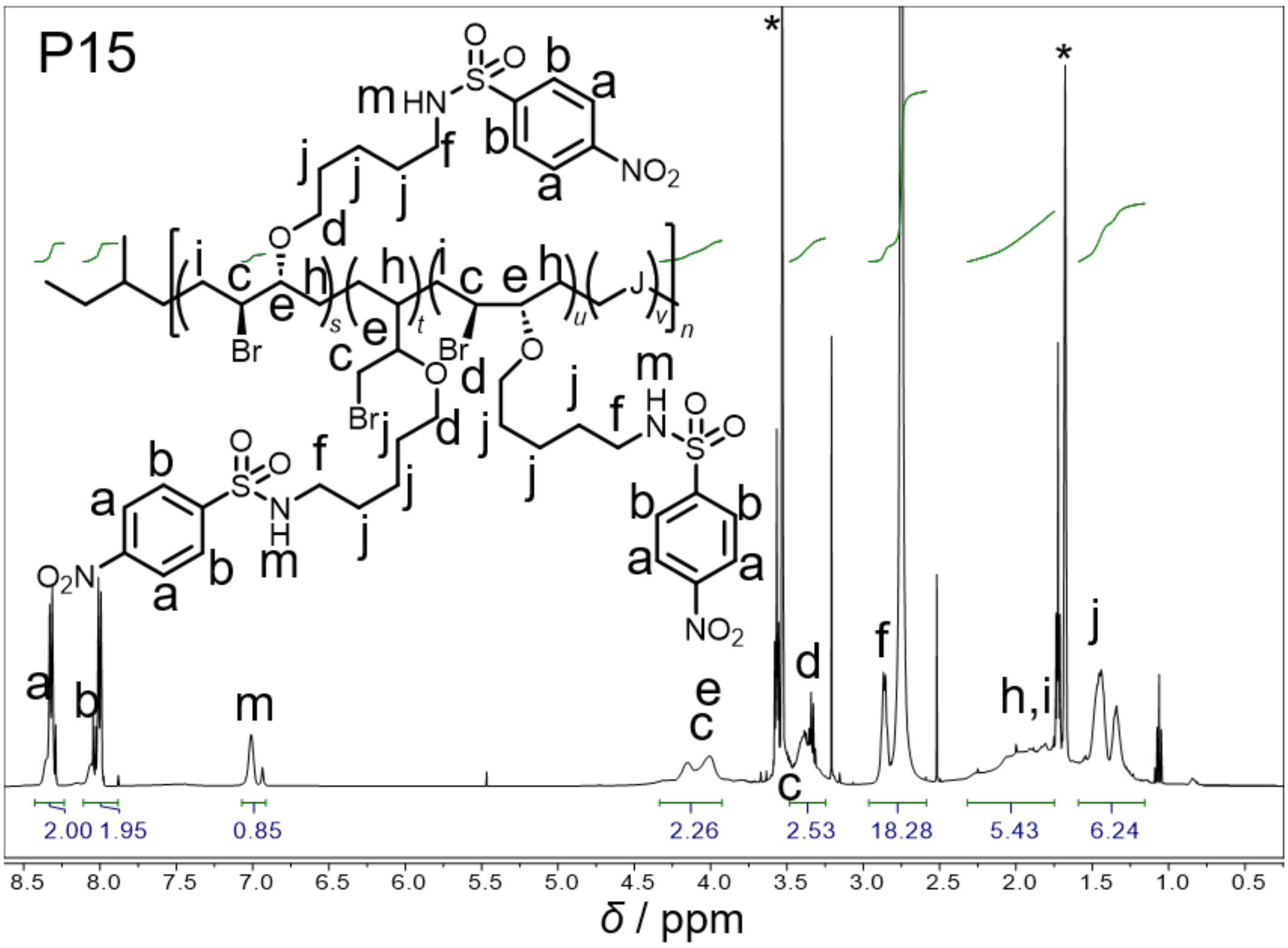

Figure S63. ^{1}H NMR spectrum of **P15** recorded in THF-d8 at 300 MHz. The residual solvent signal is marked with an asterisk.

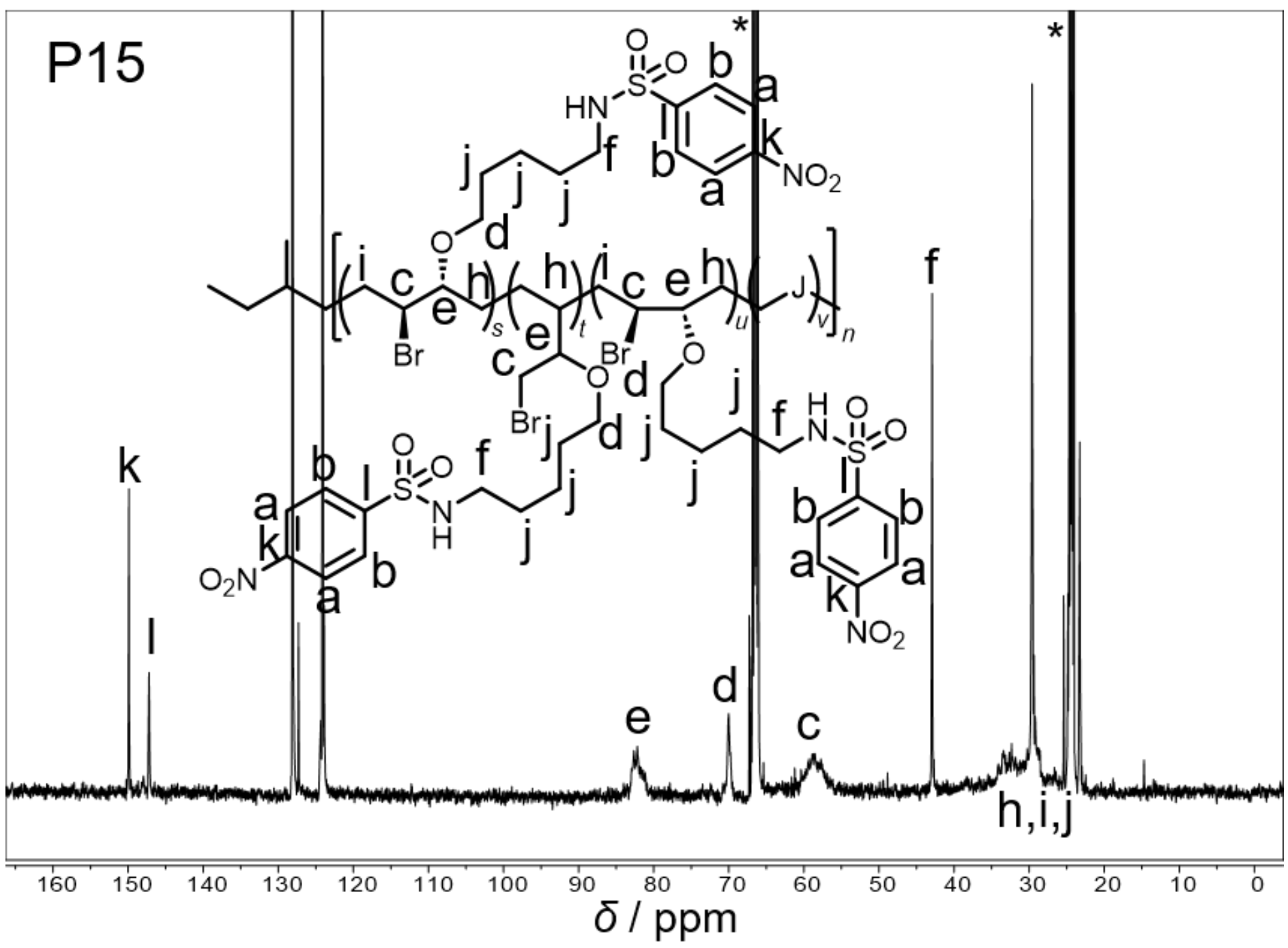

Figure S64. ^{13}C NMR spectrum of **P15** recorded in THF-d8 at 75 MHz. The residual solvent signal is marked with an asterisk.

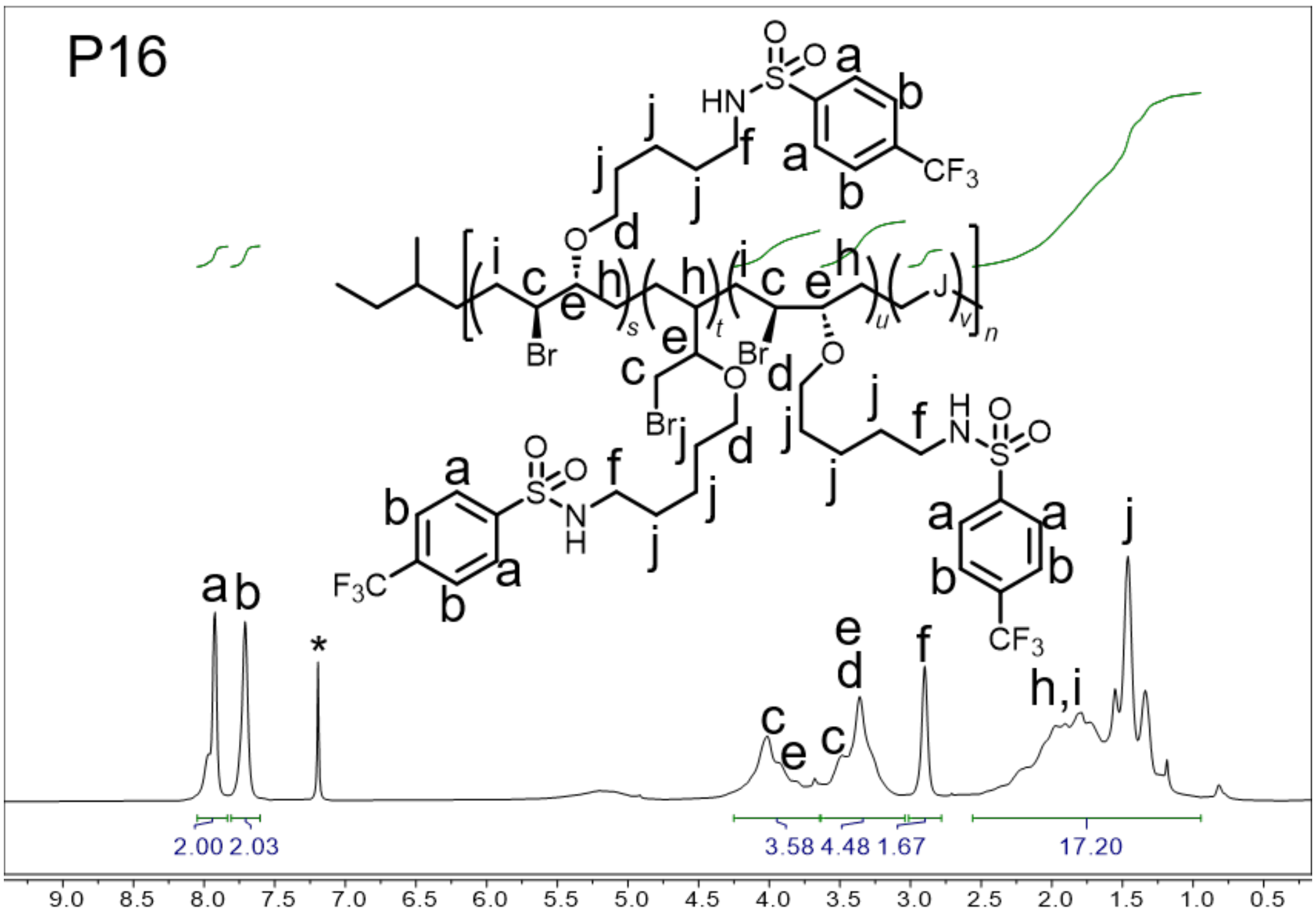

Figure S65. ^{1}H NMR spectrum of **P16** recorded in $CDCl_3$ at 300 MHz. The residual solvent signal is marked with an asterisk.

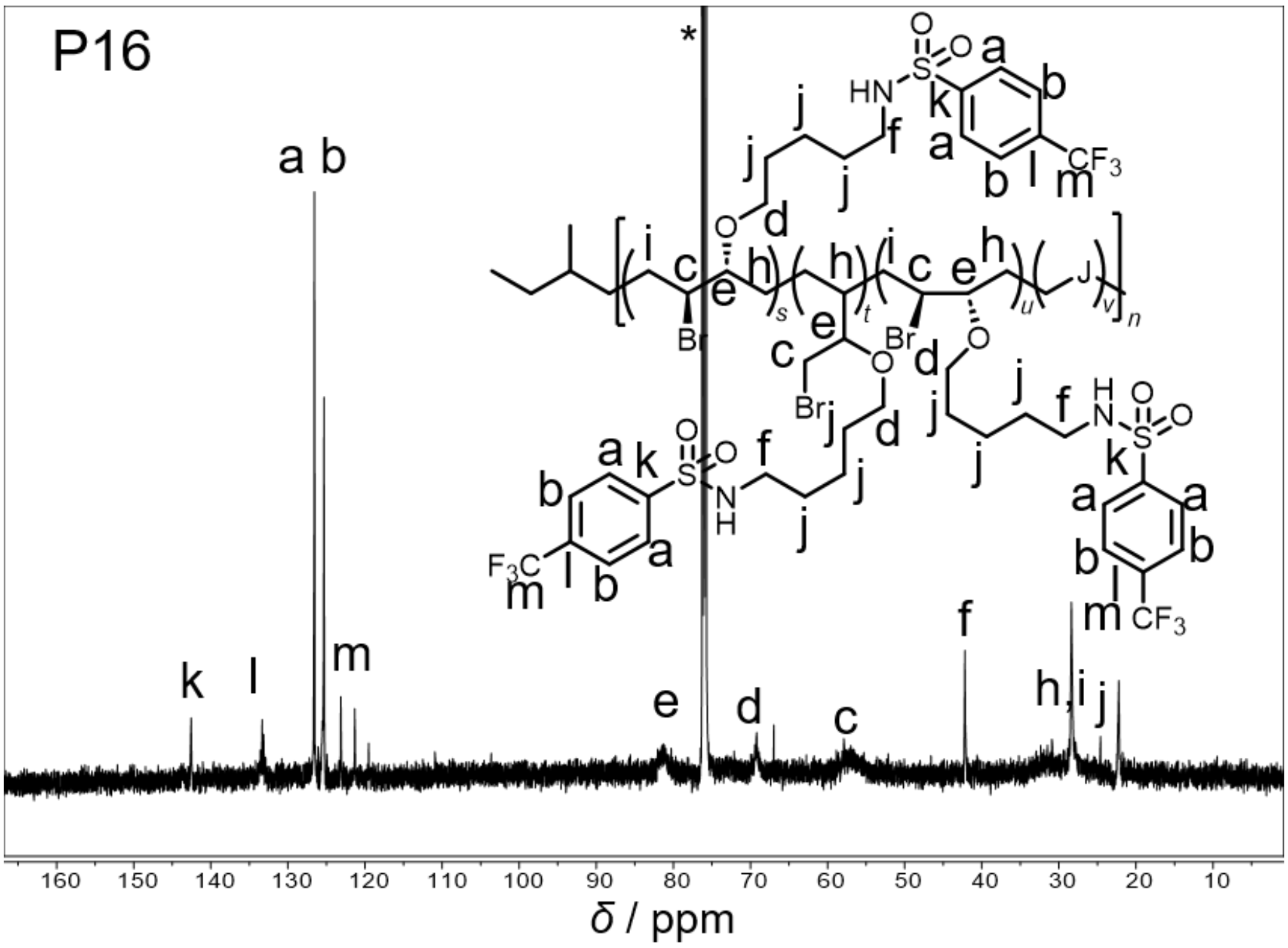

Figure S66. ^{13}C NMR spectrum of **P16** recorded in $CDCl_3$ at 75 MHz. The residual solvent signal is marked with an asterisk.

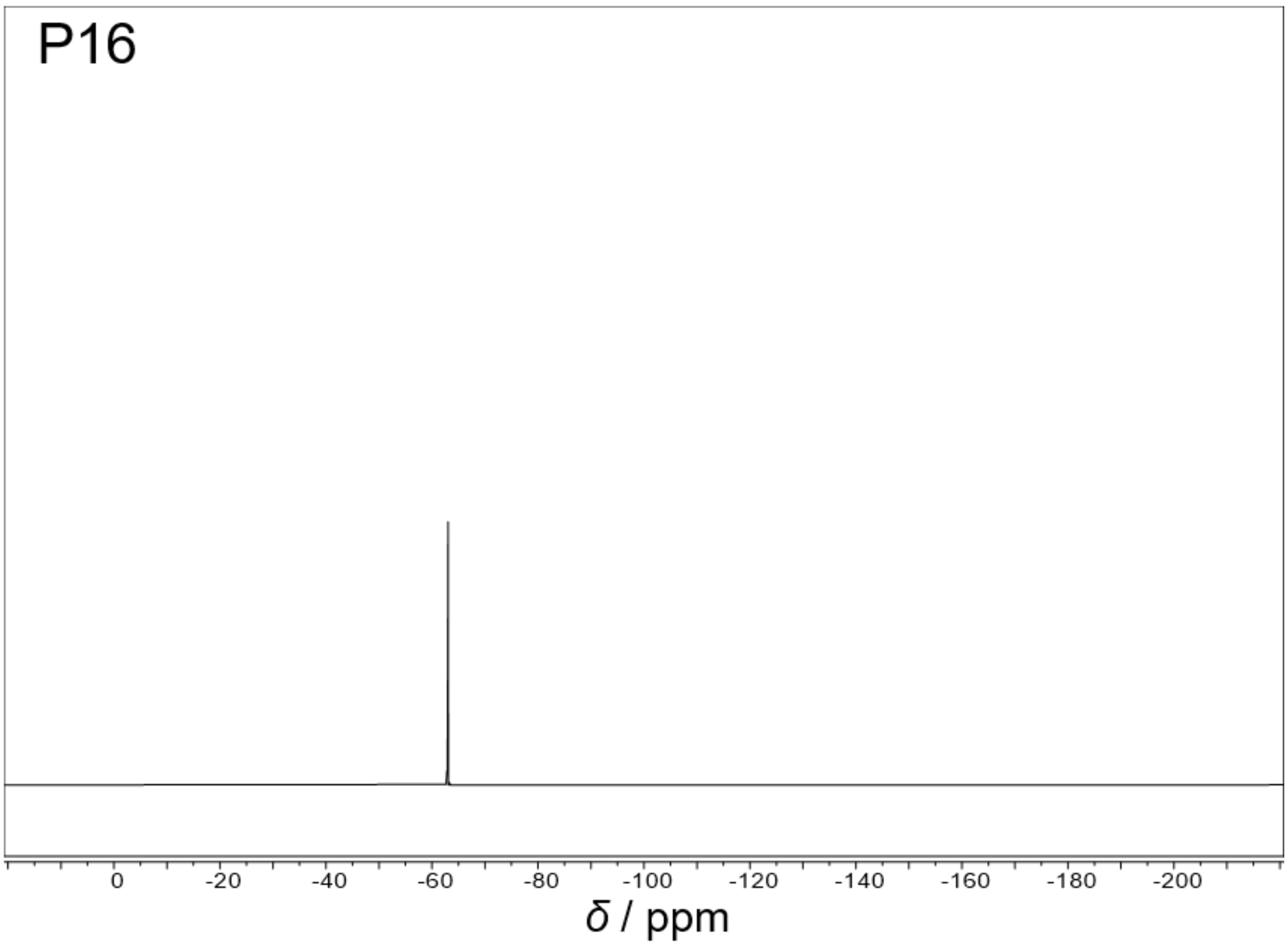

Figure S67. ^{19}F NMR spectrum of **P16** recorded in $CDCl_3$ at 282 MHz.

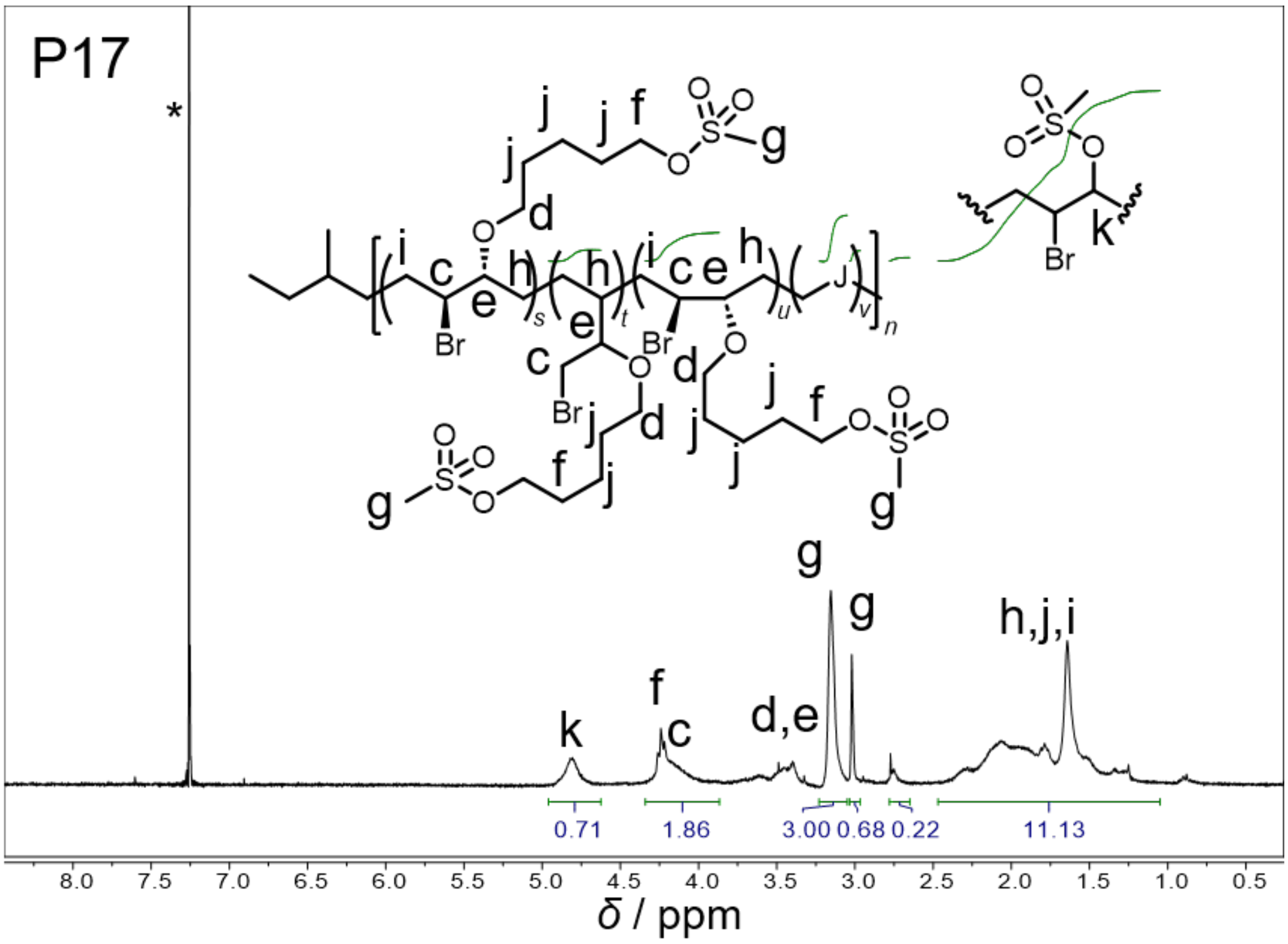

Figure S68. ^{1}H NMR spectrum of **P17** recorded in $CDCl_3$ at 300 MHz. The residual solvent signal is marked with an asterisk.

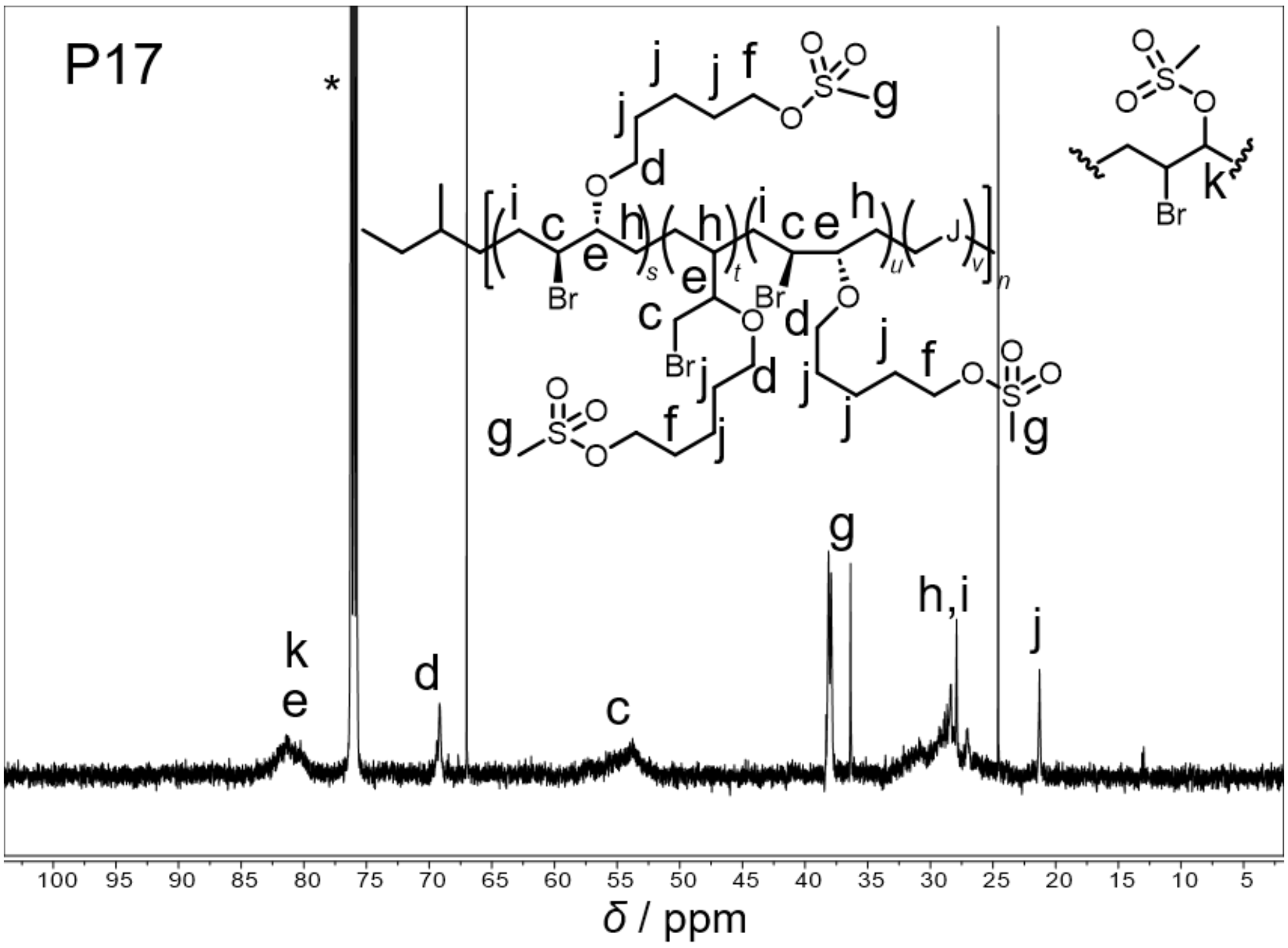

Figure S69. ^{13}C NMR spectrum of **P17** recorded in $CDCl_3$ at 75 MHz. The residual solvent signal is marked with an asterisk.

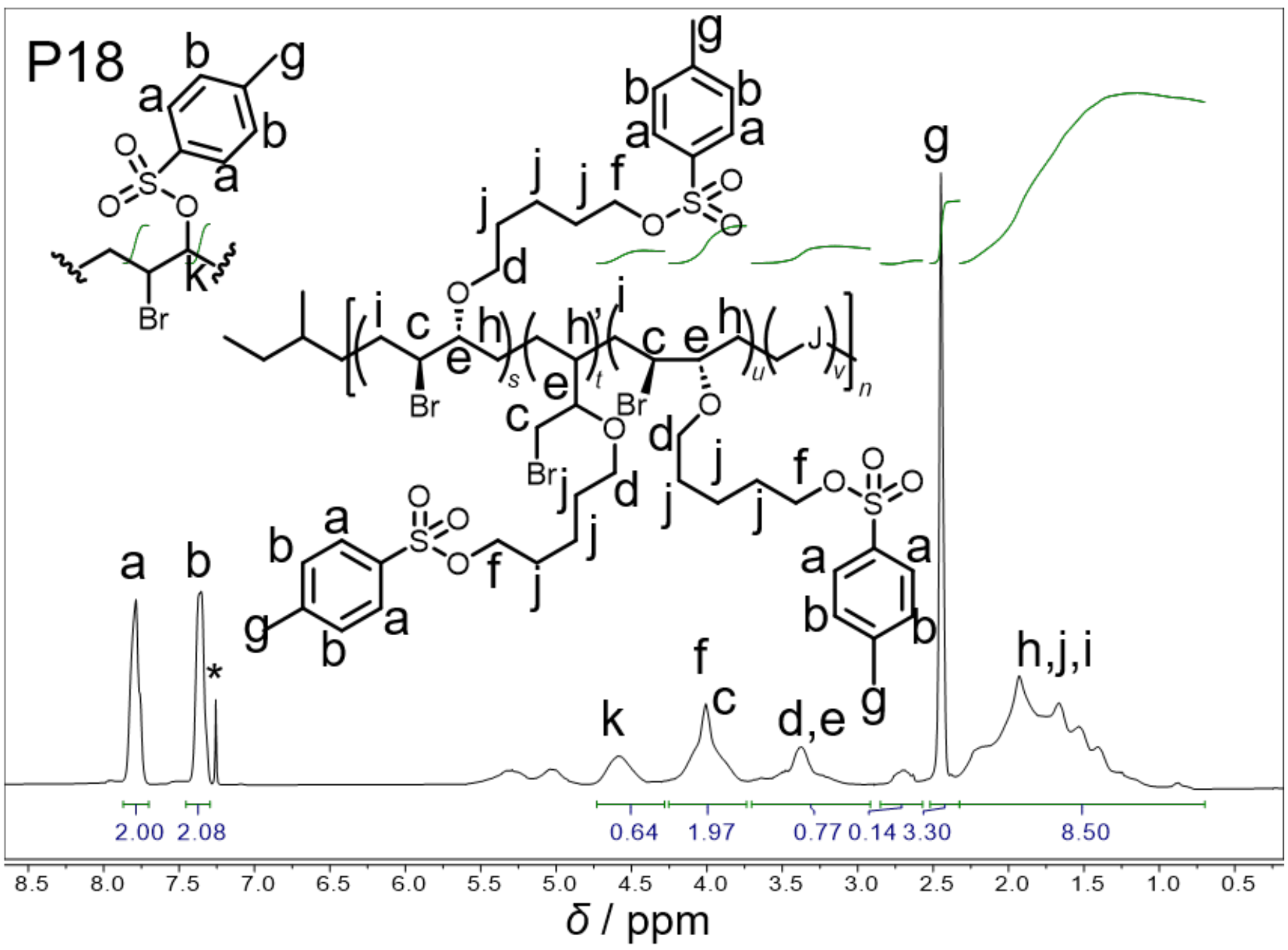

Figure S70. ^{1}H NMR spectrum of **P18** recorded in $CDCl_3$ at 300 MHz. The residual solvent signal is marked with an asterisk.

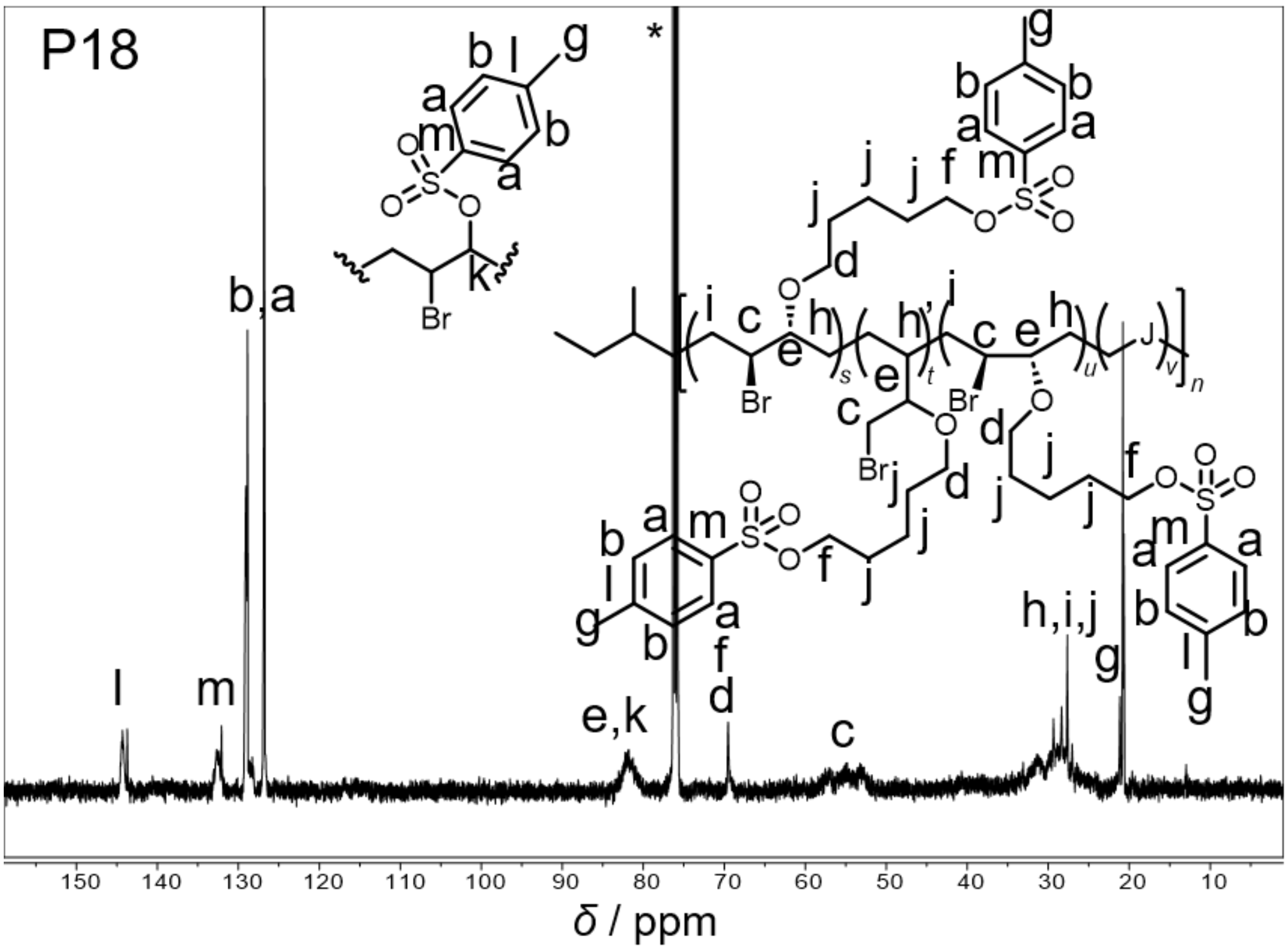

Figure S71. ^{13}C NMR spectrum of **P18** recorded in $CDCl_3$ at 75 MHz. The residual solvent signal is marked with an asterisk.

C.2 **FTIR spectra**

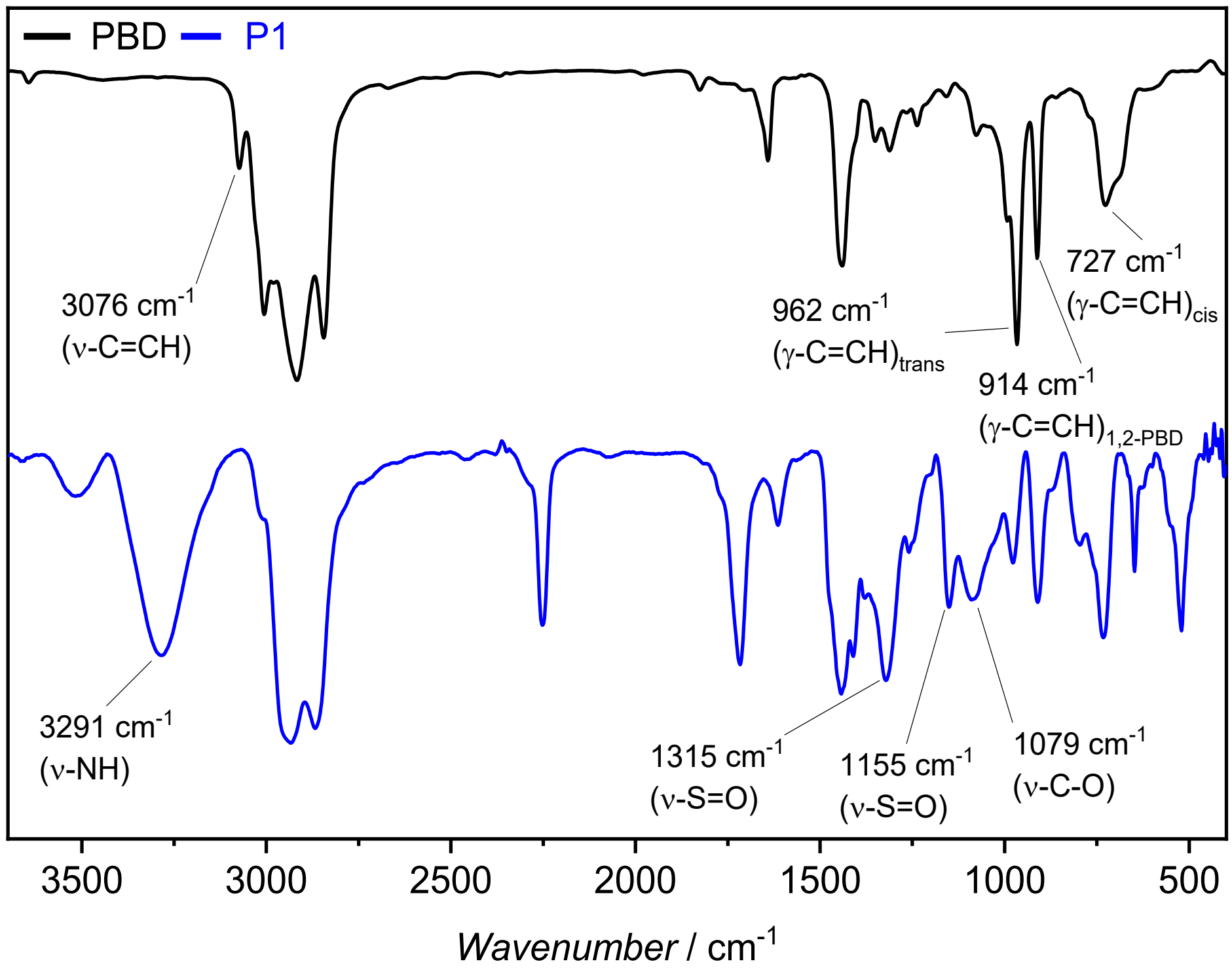

Figure S72. FTIR spectra of pristine PBD (black, top) and **P1** (blue, bottom) recorded in transmission mode as thin films on KBr plates.

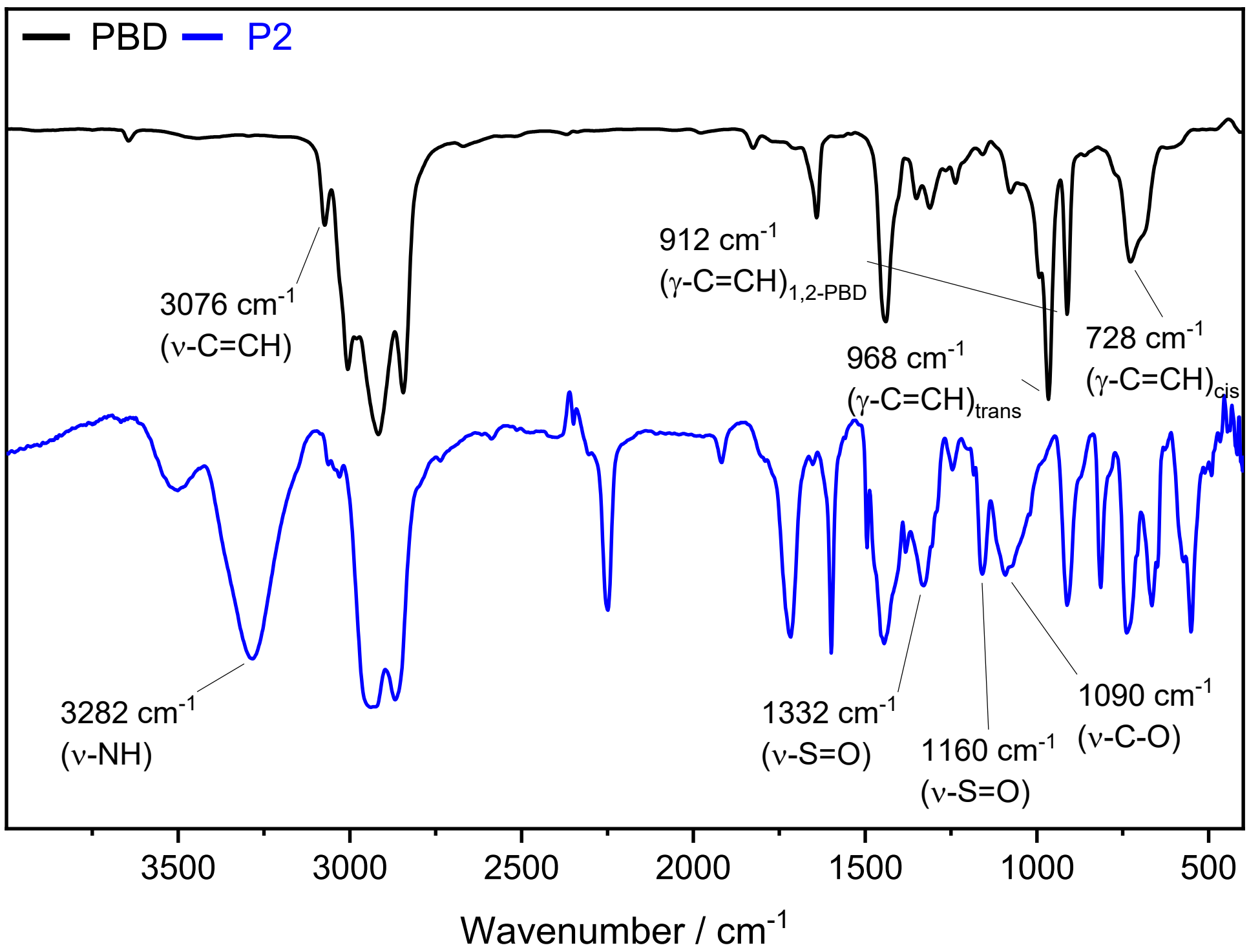

Figure S73. FTIR spectra of pristine PBD (black, top) and **P2** (blue, bottom) recorded in transmission mode as thin films on KBr plates.

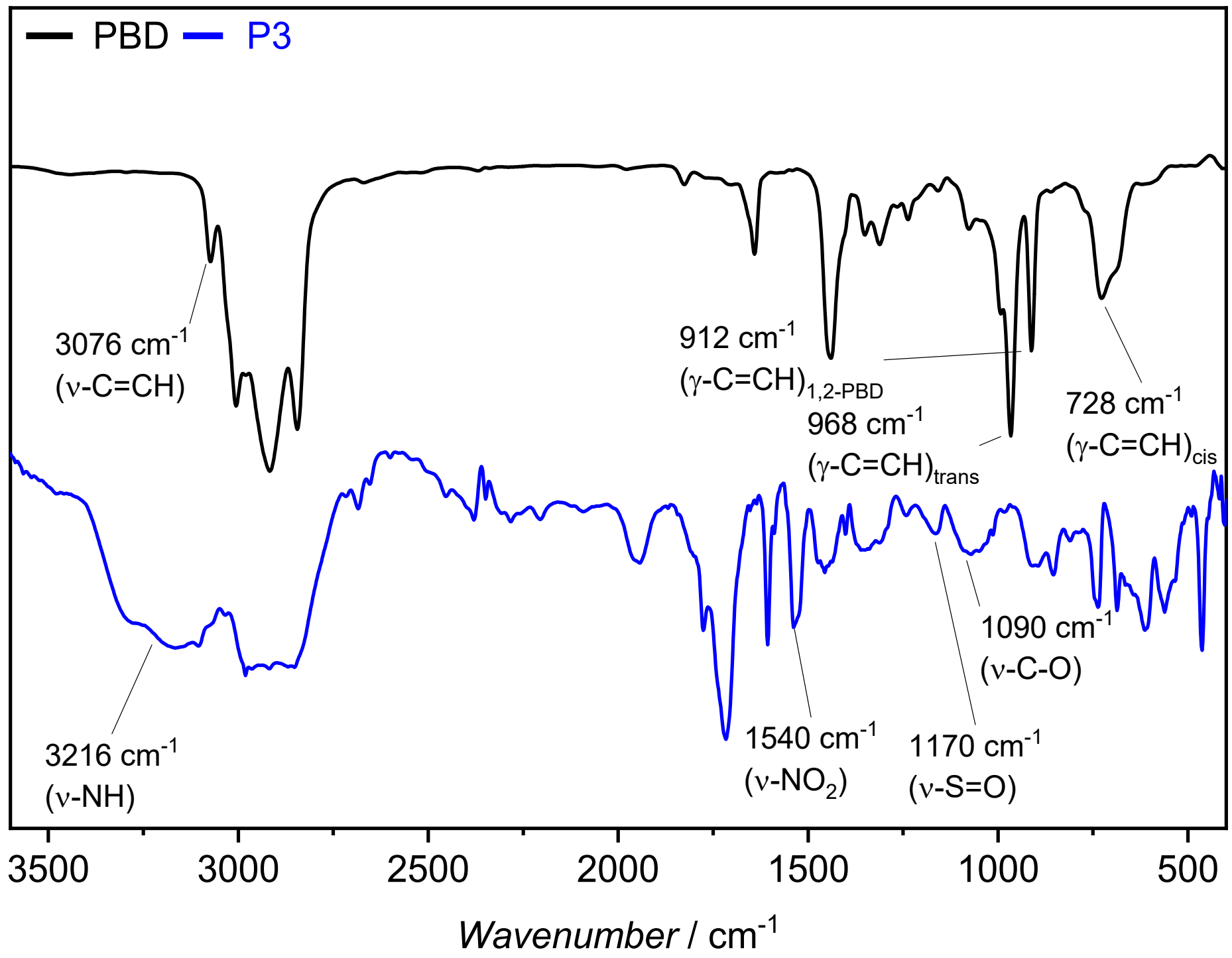

Figure S74. FTIR spectra of pristine PBD (black, top) and **P3** (blue, bottom) recorded in transmission mode as thin films on KBr plates.

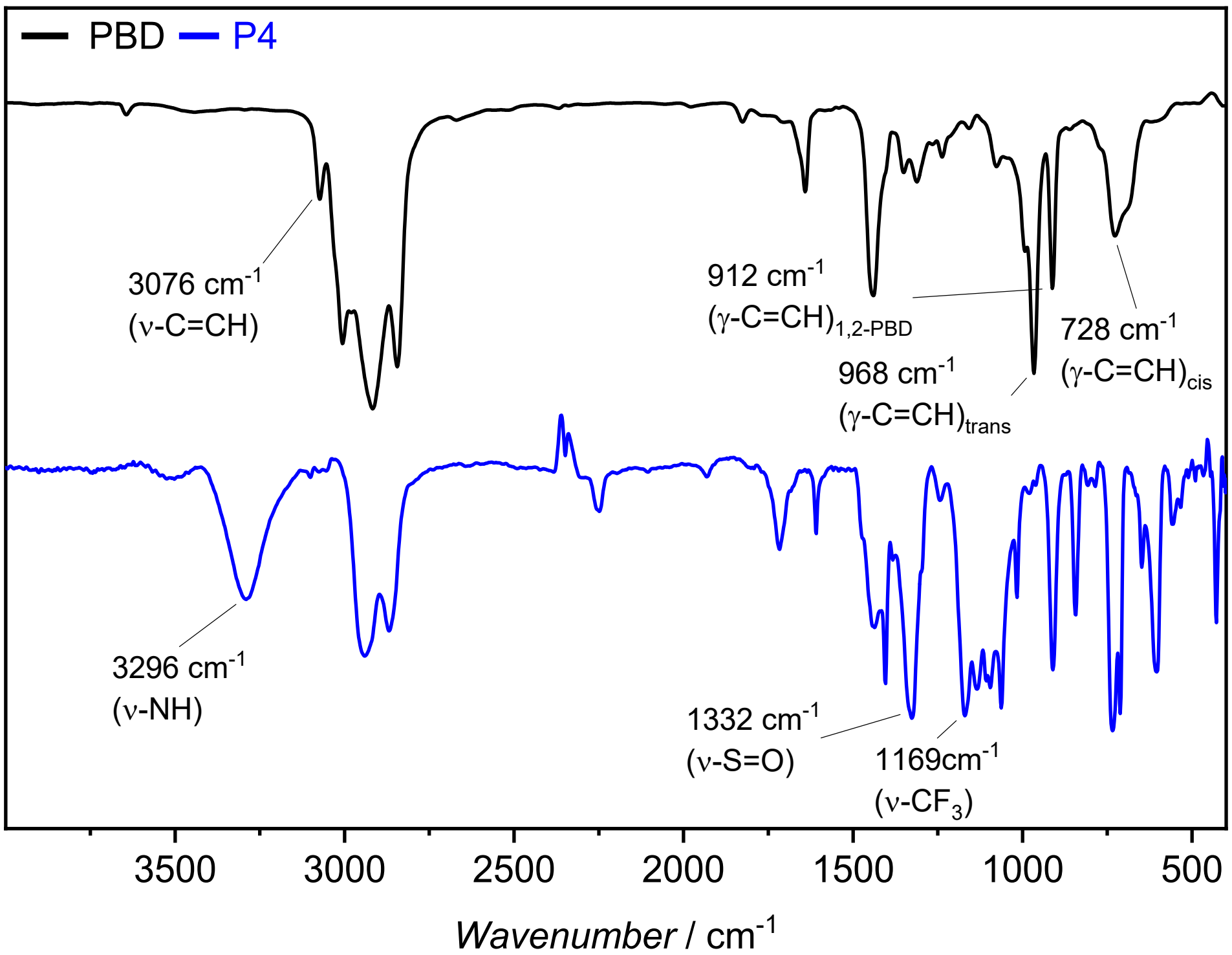

Figure S75. FTIR spectra of pristine PBD (black, top) and **P4** (blue, bottom) recorded in transmission mode as thin films on KBr plates.

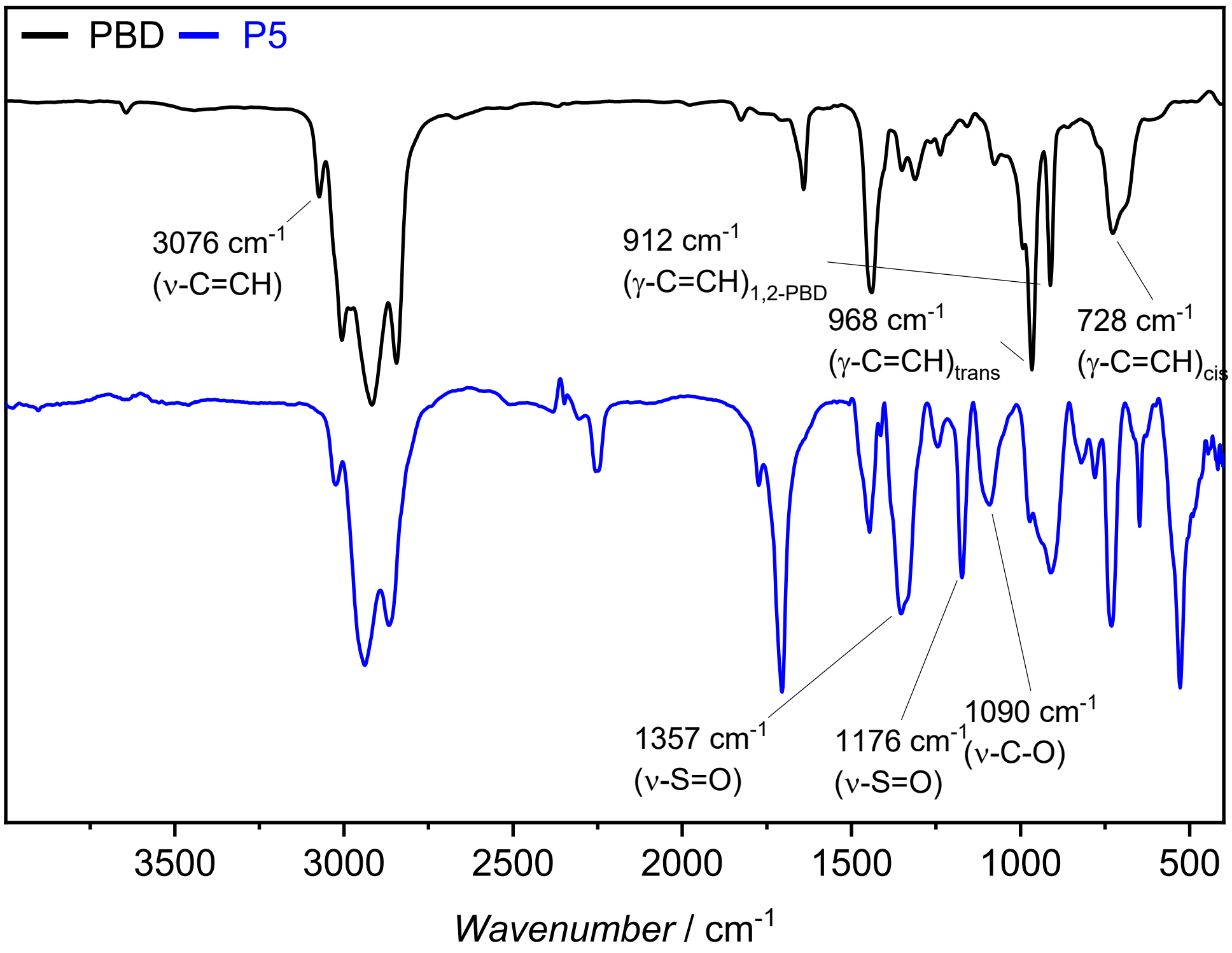

Figure S76. FTIR spectra of pristine PBD (black, top) and **P5** (blue, bottom) recorded in transmission mode as thin films on KBr plates.

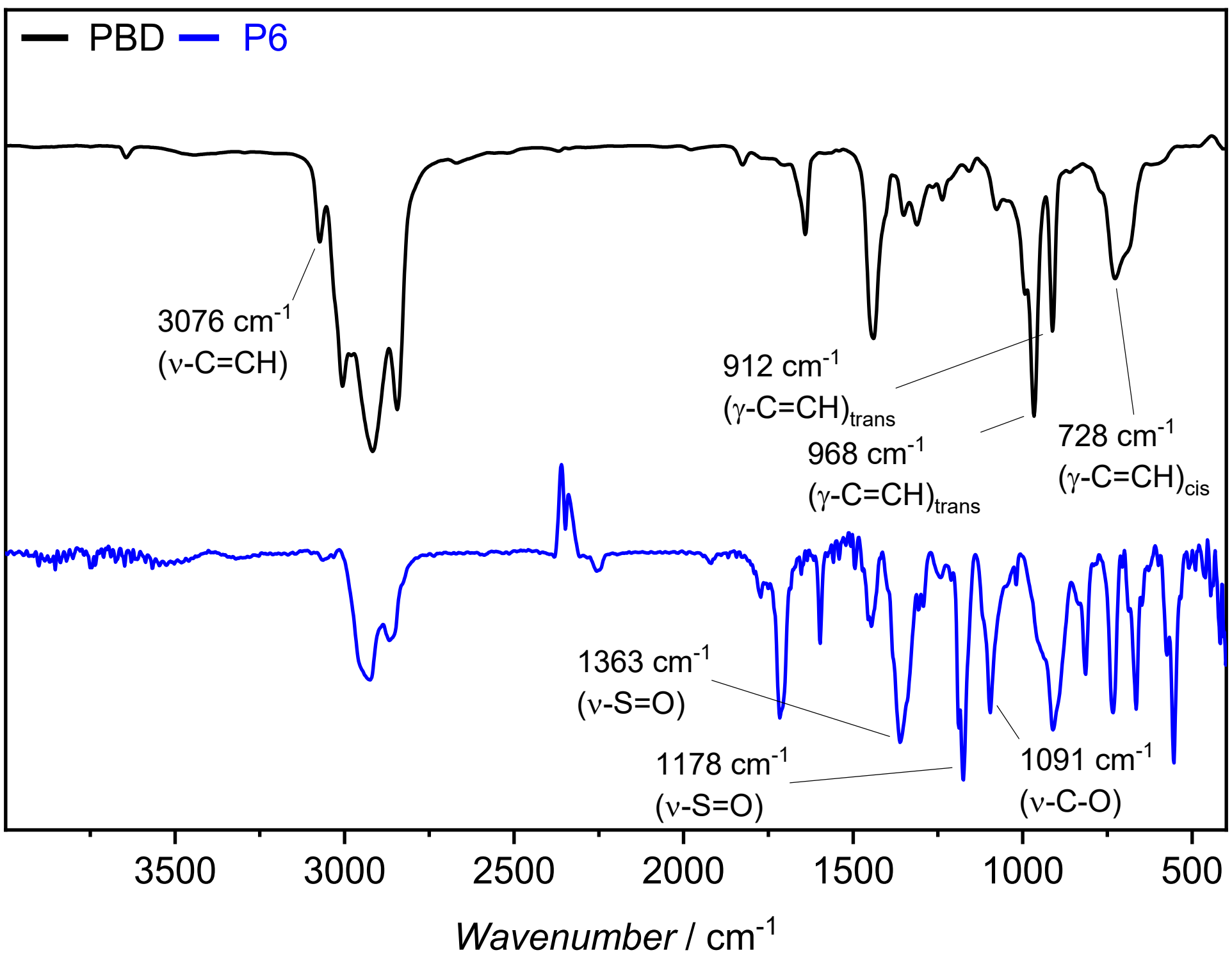

Figure S77. FTIR spectra of pristine PBD (black, top) and **P6** (blue, bottom) recorded in transmission mode as thin films on KBr plates.

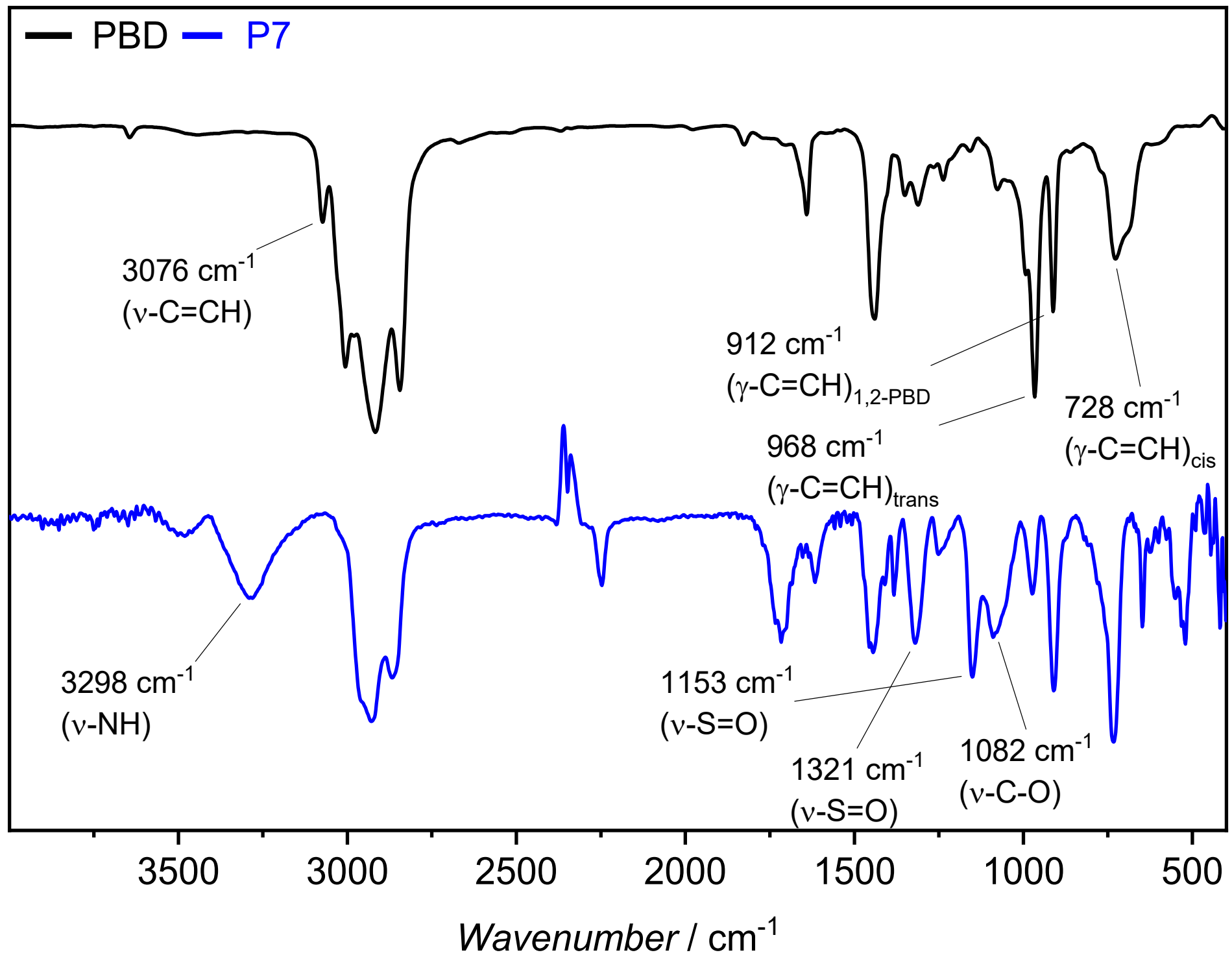

Figure S78. FTIR spectra of pristine PBD (black, top) and **P7** (blue, bottom) recorded in transmission mode as thin films on KBr plates.

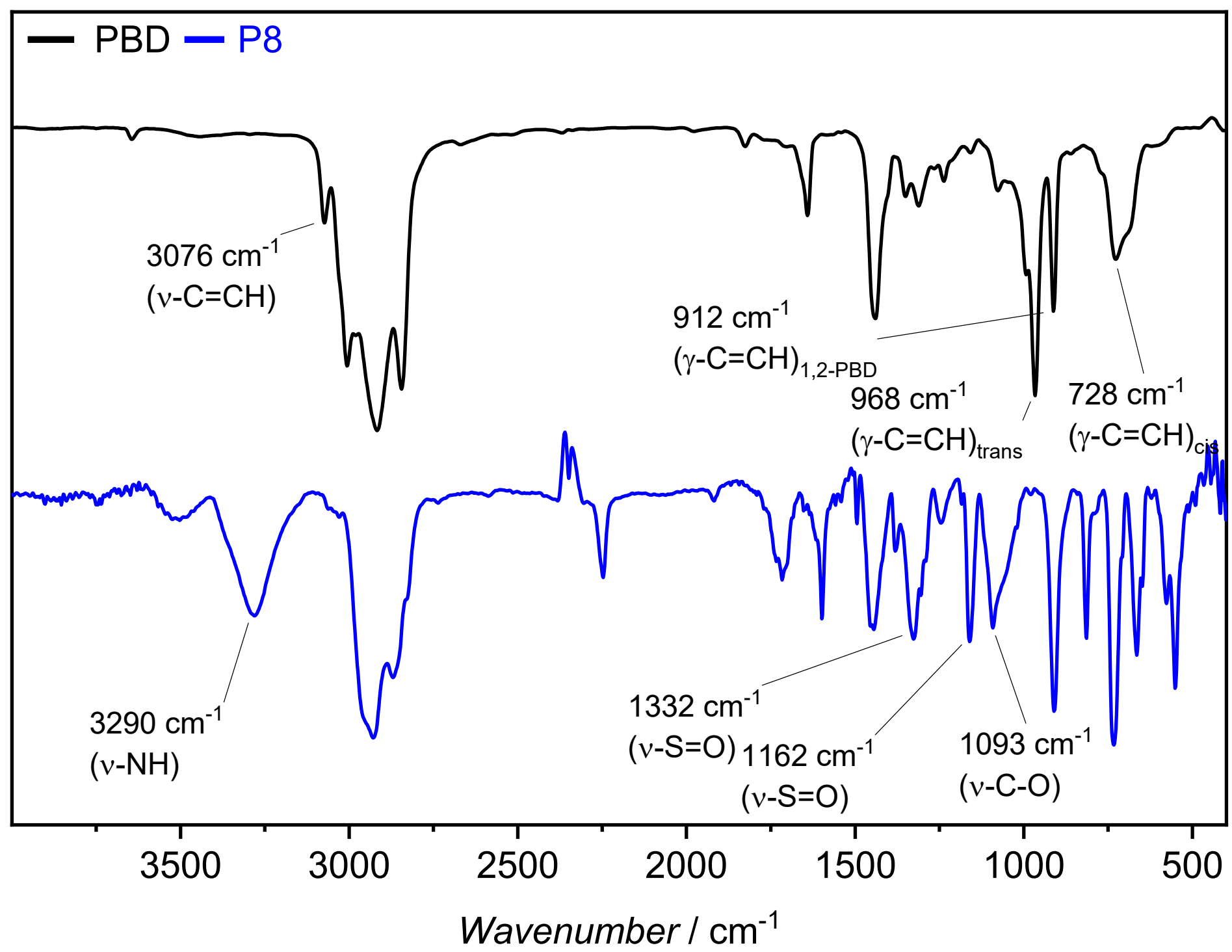

Figure S79. FTIR spectra of pristine PBD (black, top) and **P8** (blue, bottom) recorded in transmission mode as thin films on KBr plates.

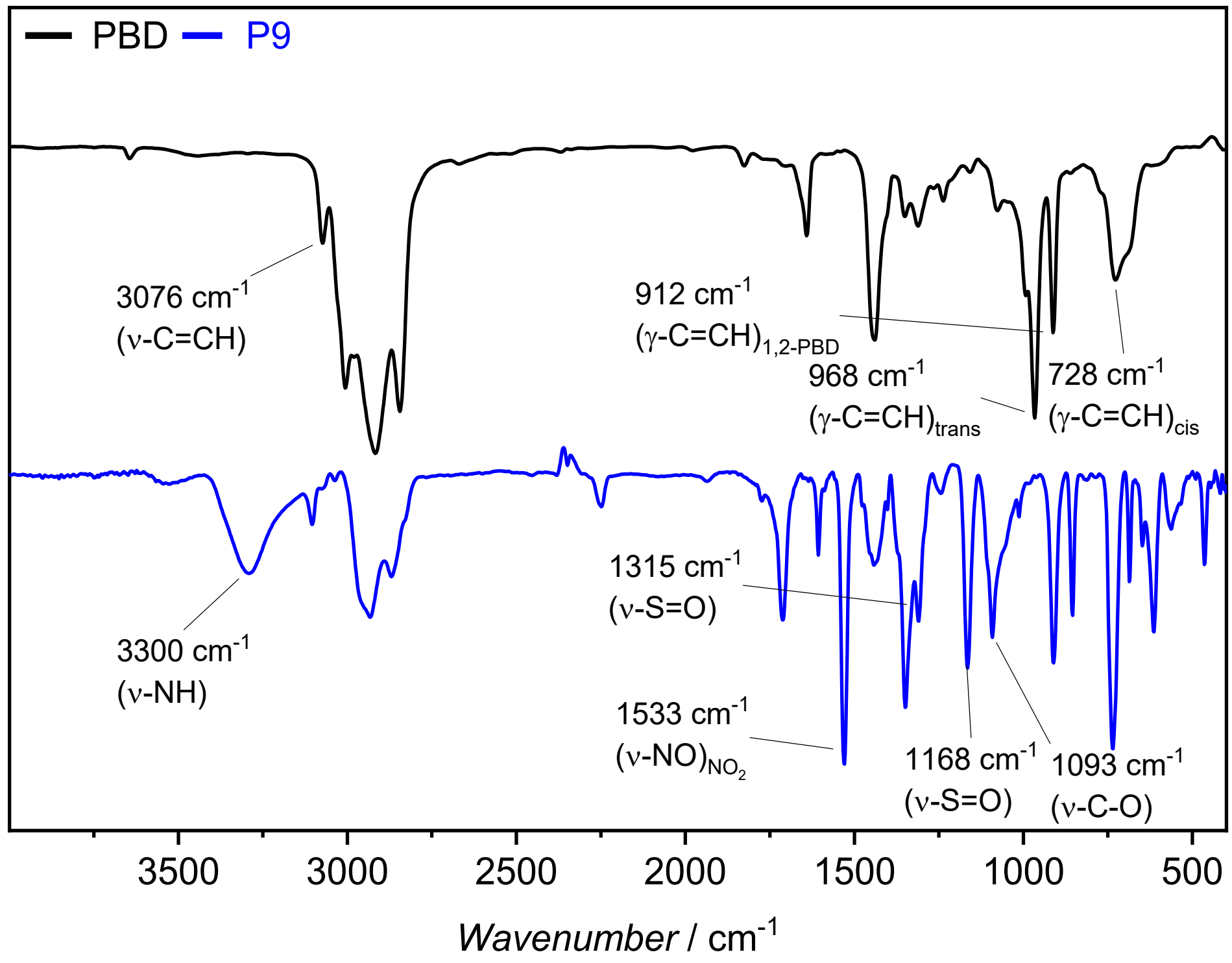

Figure S80. FTIR spectra of pristine PBD (black, top) and **P9** (blue, bottom) recorded in transmission mode as thin films on KBr plates.

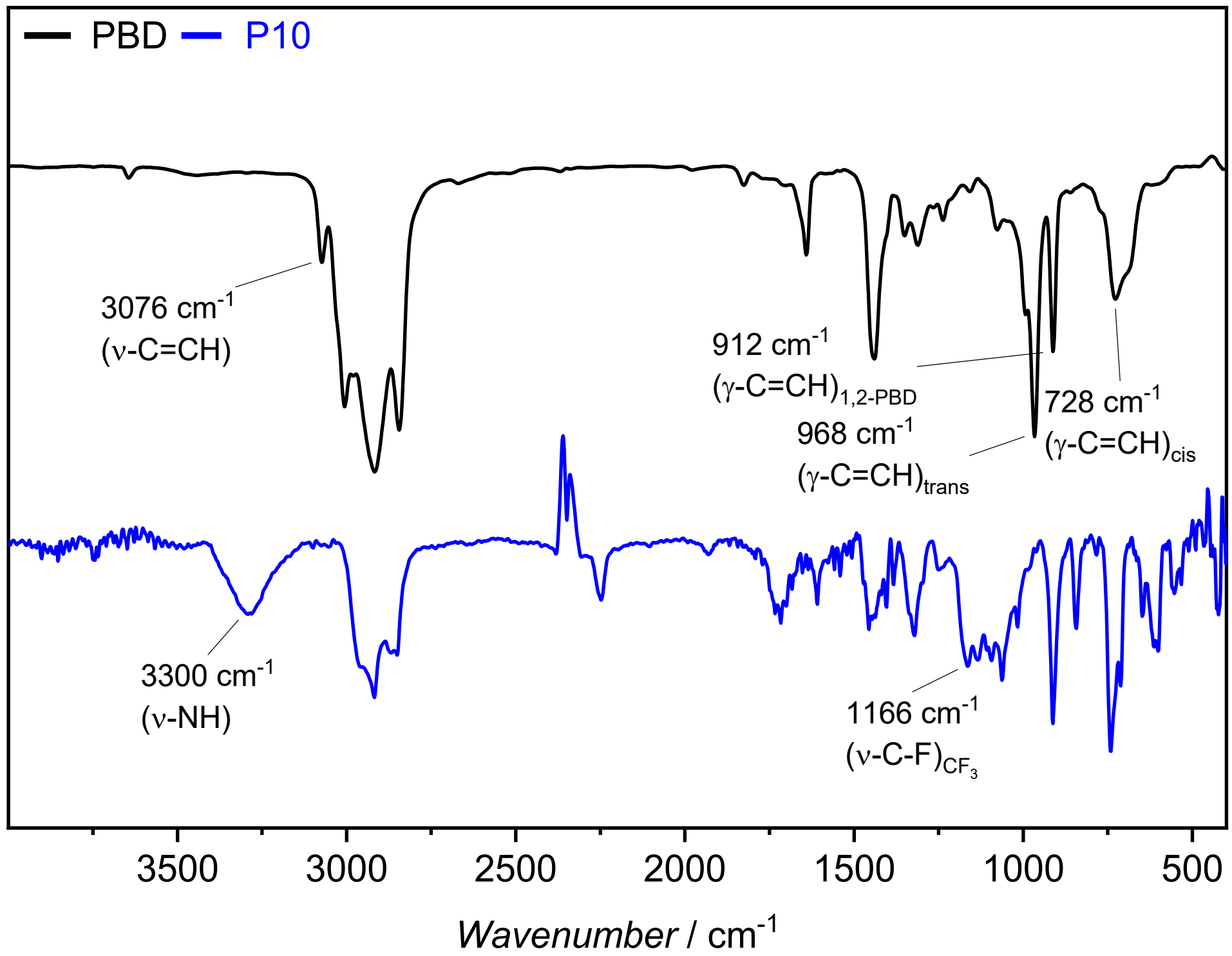

Figure S81. FTIR spectra of pristine PBD (black, top) and **P10** (blue, bottom) recorded in transmission mode as thin films on KBr plates.

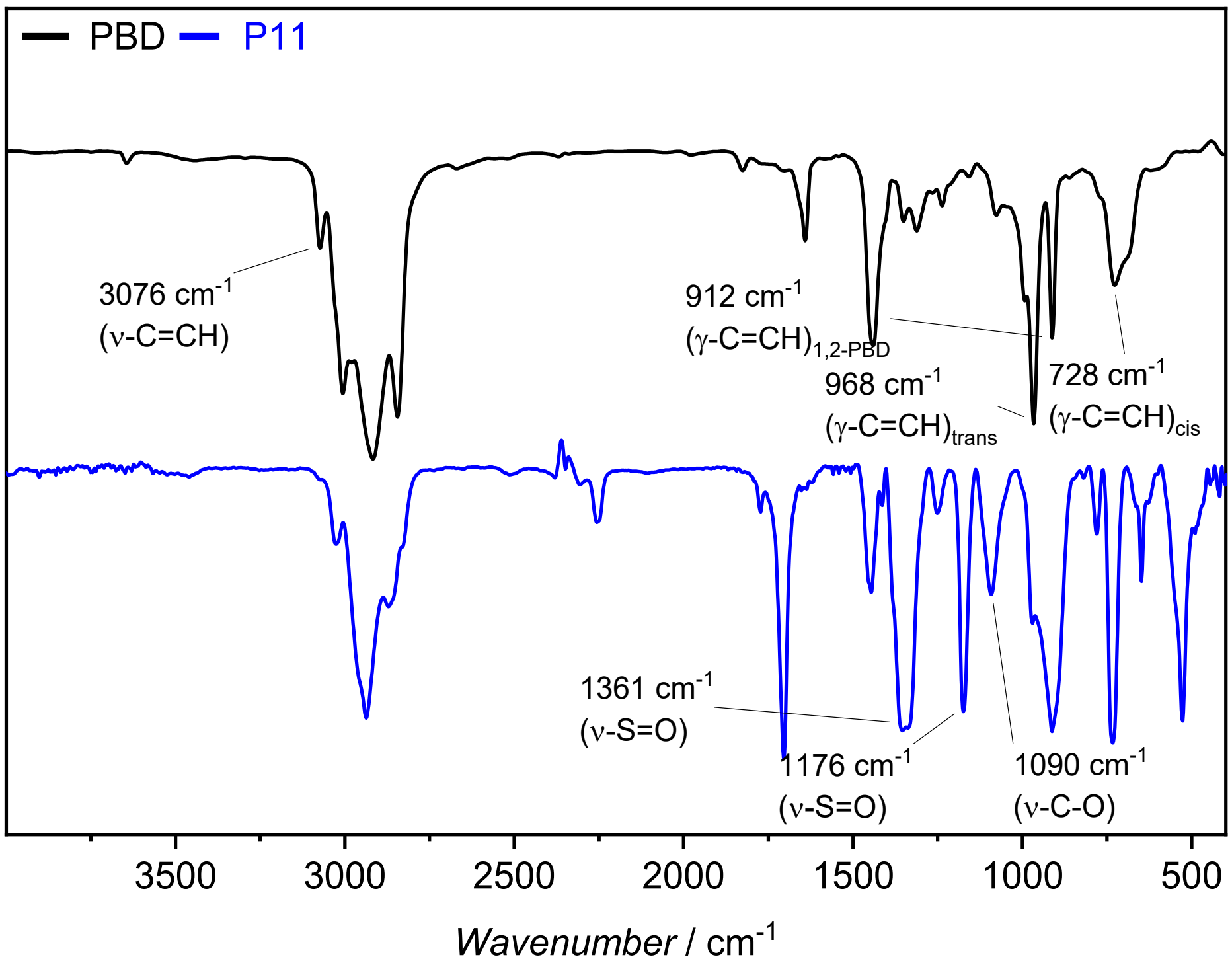

Figure S82. FTIR spectra of pristine PBD (black, top) and **P11** (blue, bottom) recorded in transmission mode as thin films on KBr plates.

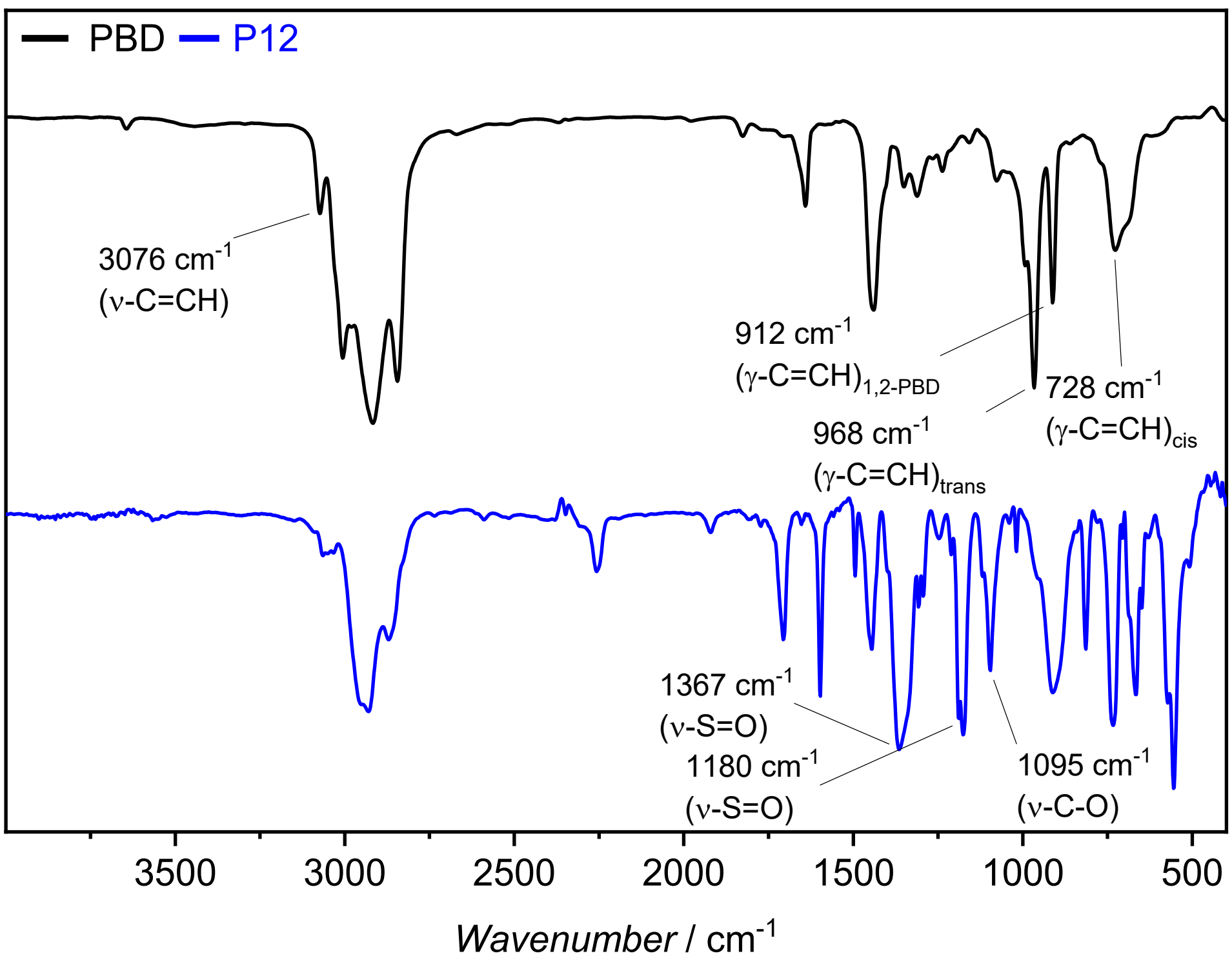

Figure S83. FTIR spectra of pristine PBD (black, top) and **P12** (blue, bottom) recorded in transmission mode as thin films on KBr plates.

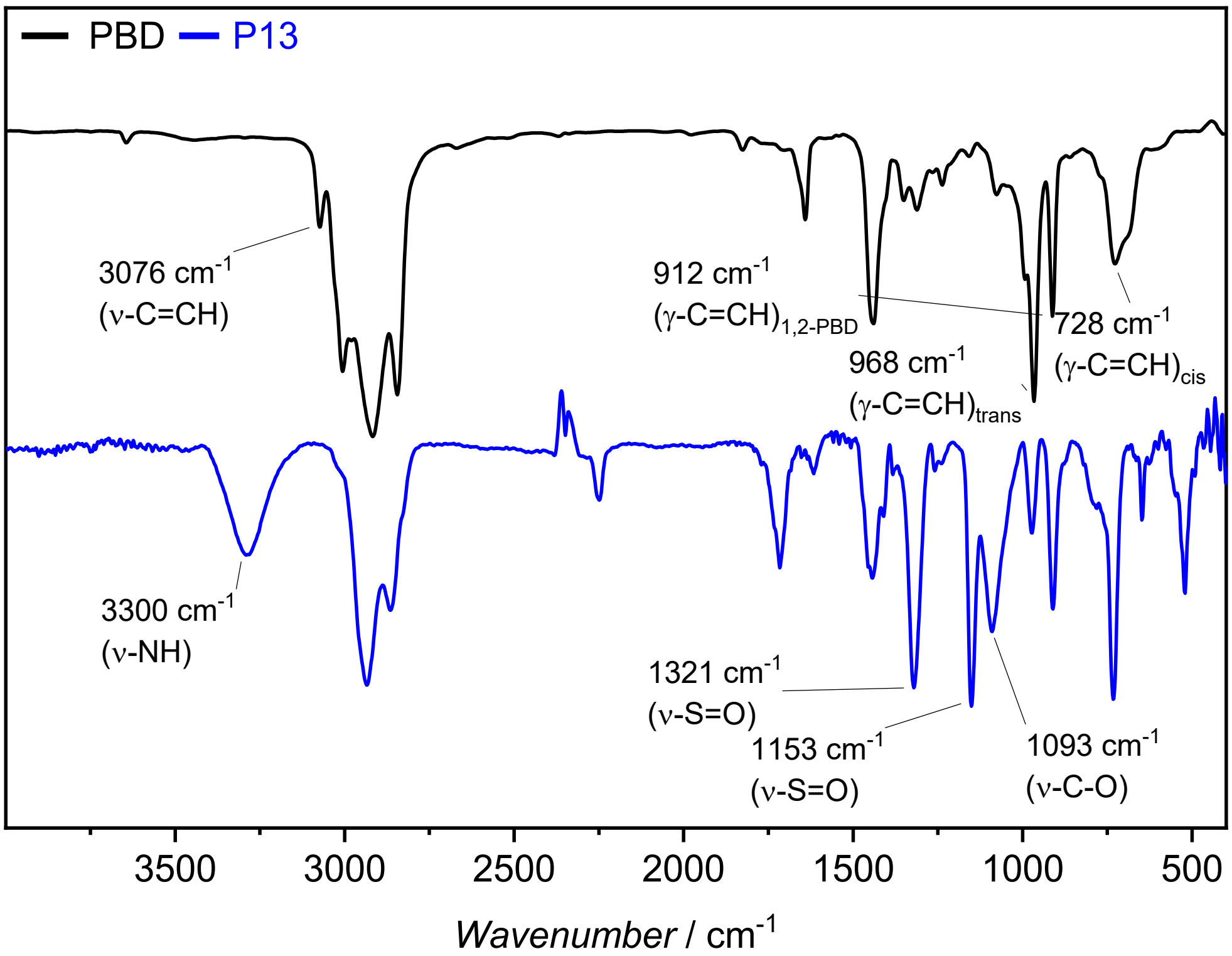

Figure S84. FTIR spectra of pristine PBD (black, top) and **P13** (blue, bottom) recorded in transmission mode as thin films on KBr plates.

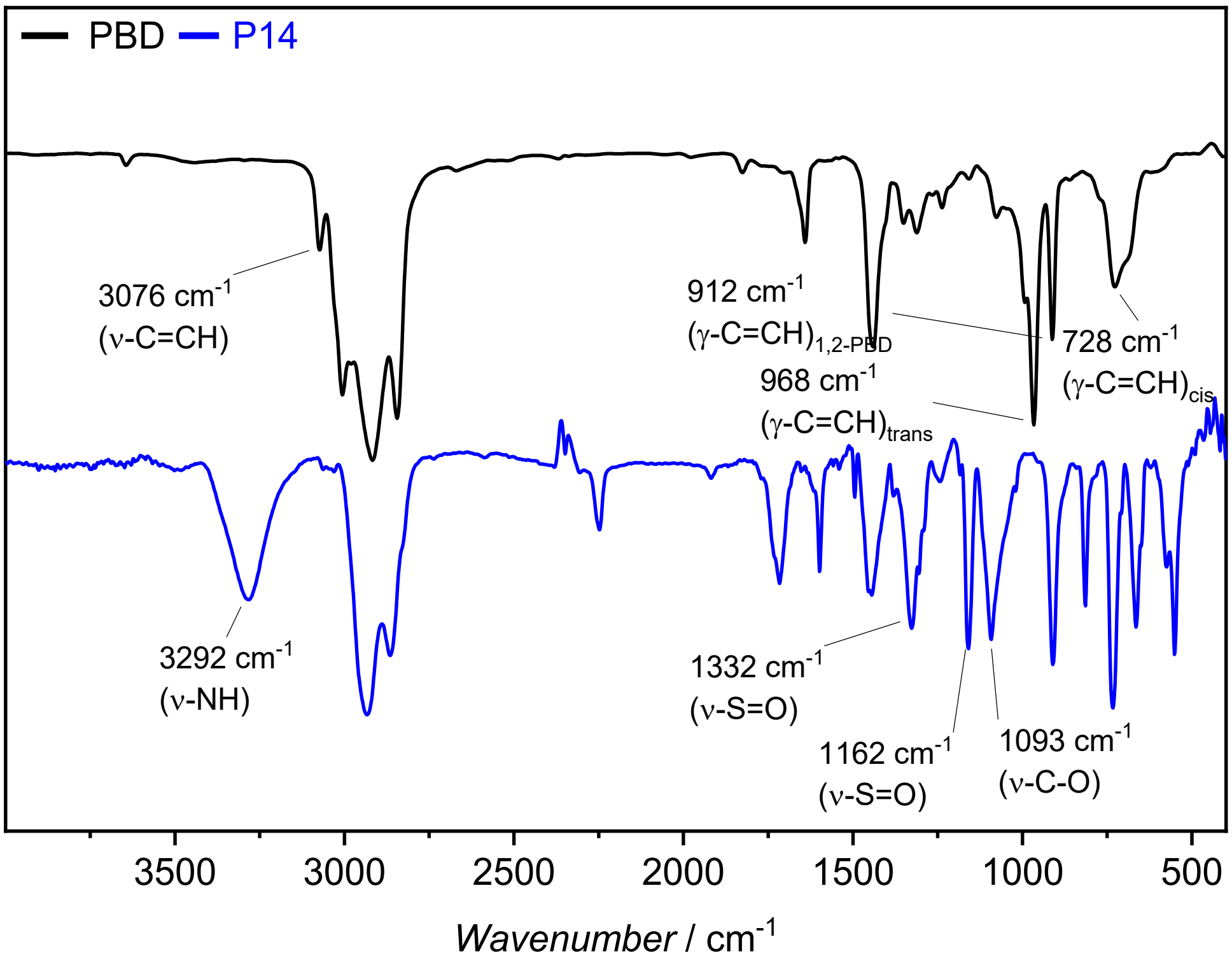

Figure S85. FTIR spectra of pristine PBD (black, top) and **P14** (blue, bottom) recorded in transmission mode as thin films on KBr plates.

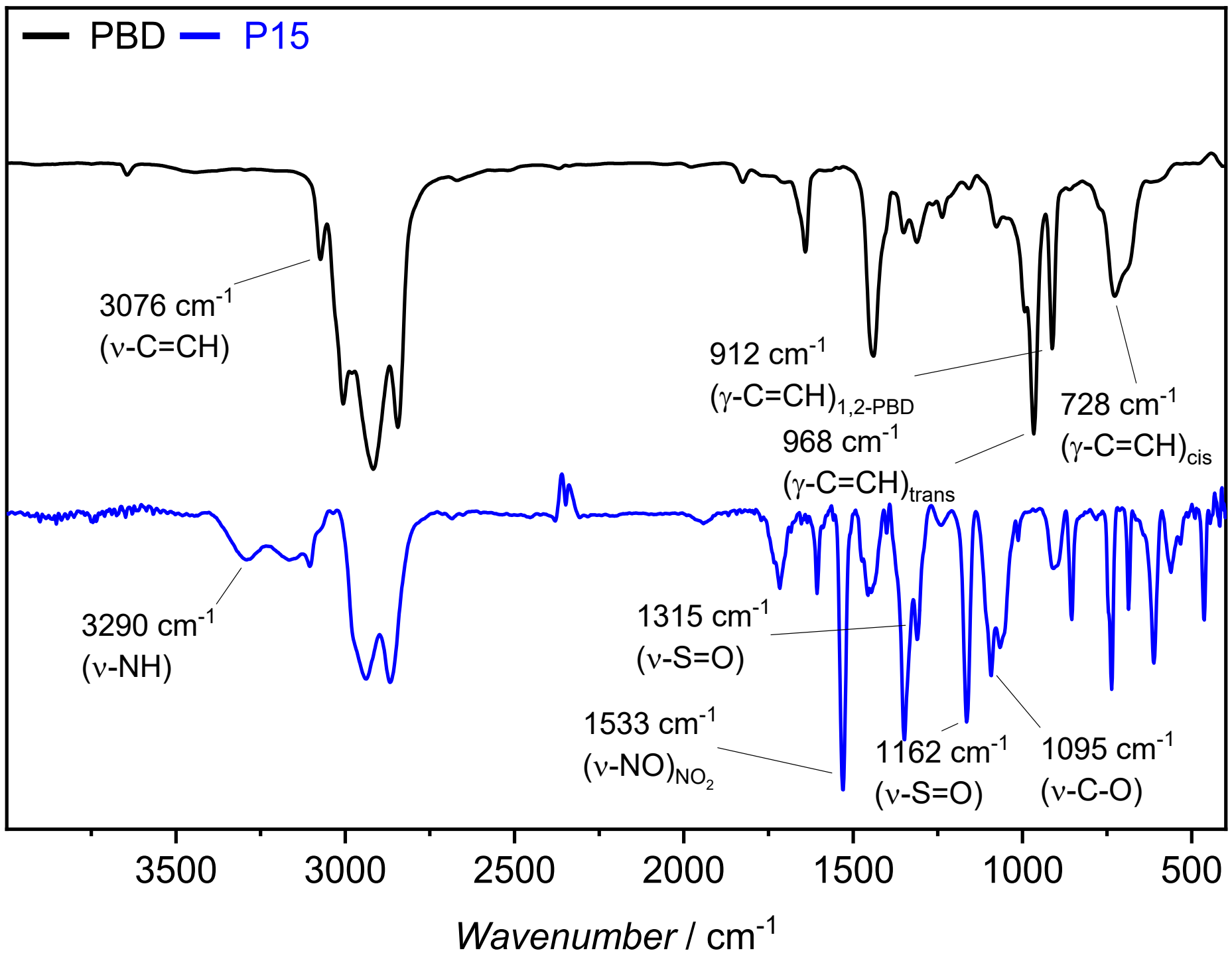

Figure S86. FTIR spectra of pristine PBD (black, top) and **P15** (blue, bottom) recorded in transmission mode as thin films on KBr plates.

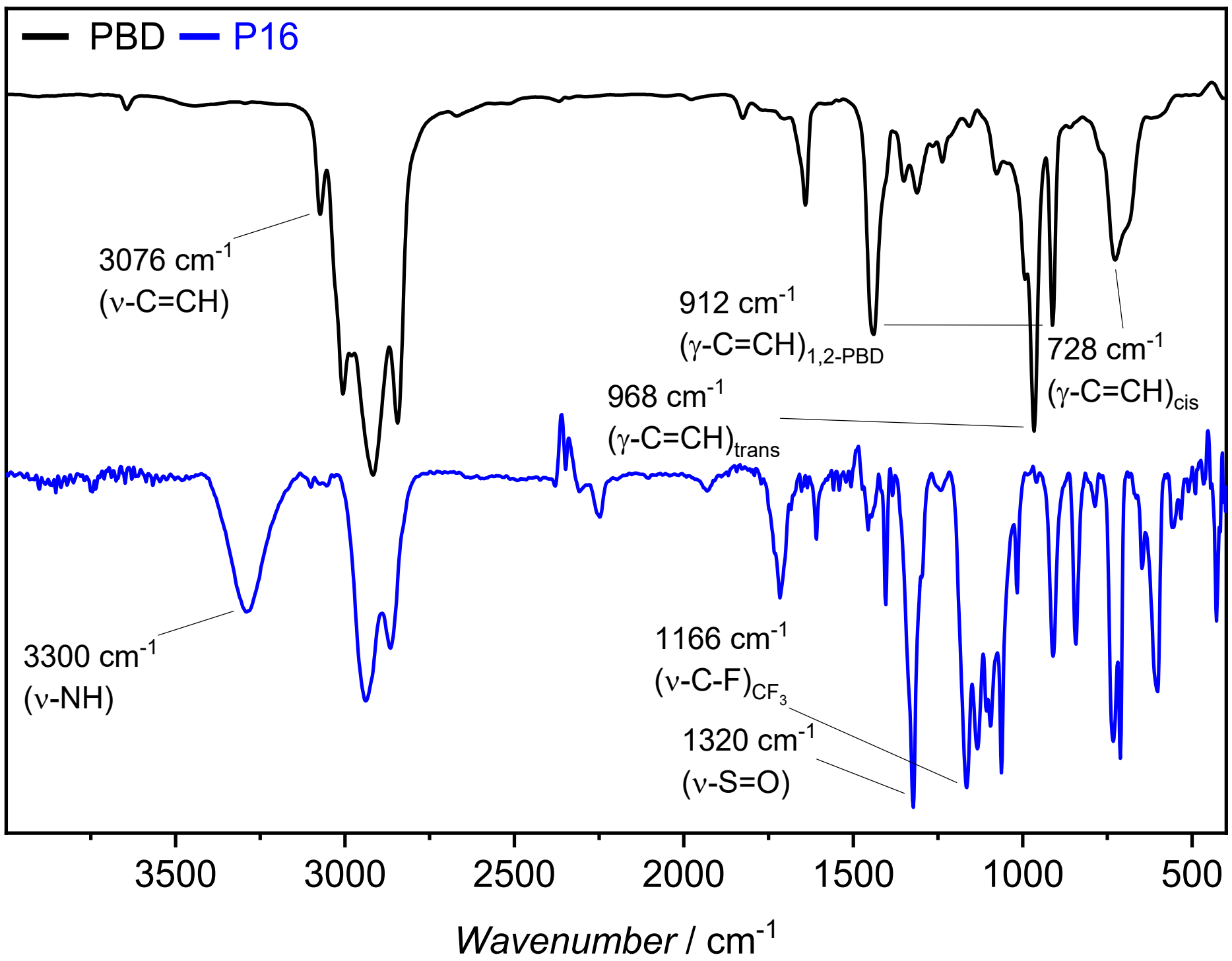

Figure S87. FTIR spectra of pristine PBD (black, top) and **P16** (blue, bottom) recorded in transmission mode as thin films on KBr plates.

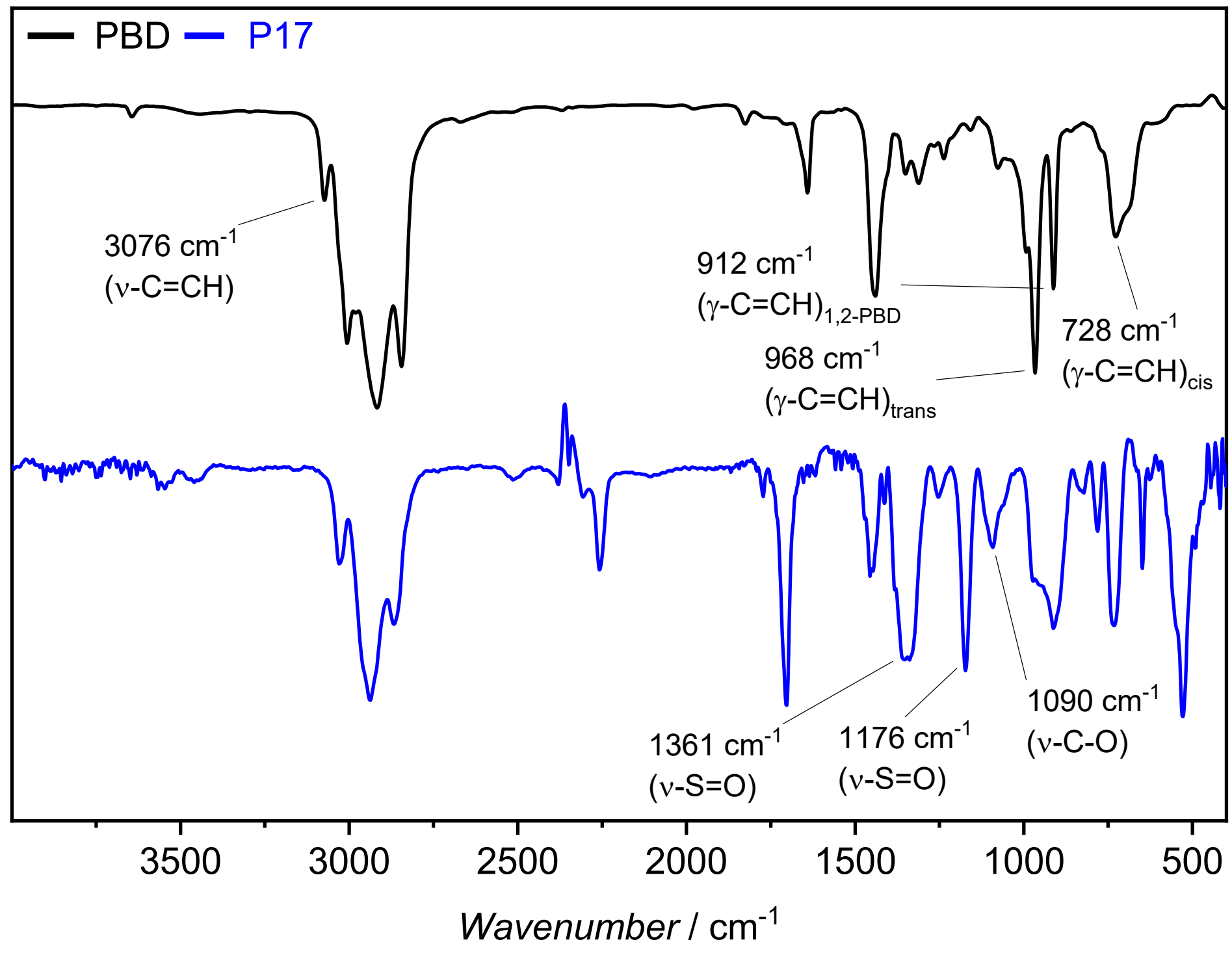

Figure S88. FTIR spectra of pristine PBD (black, top) and **P17** (blue, bottom) recorded in transmission mode as thin films on KBr plates.

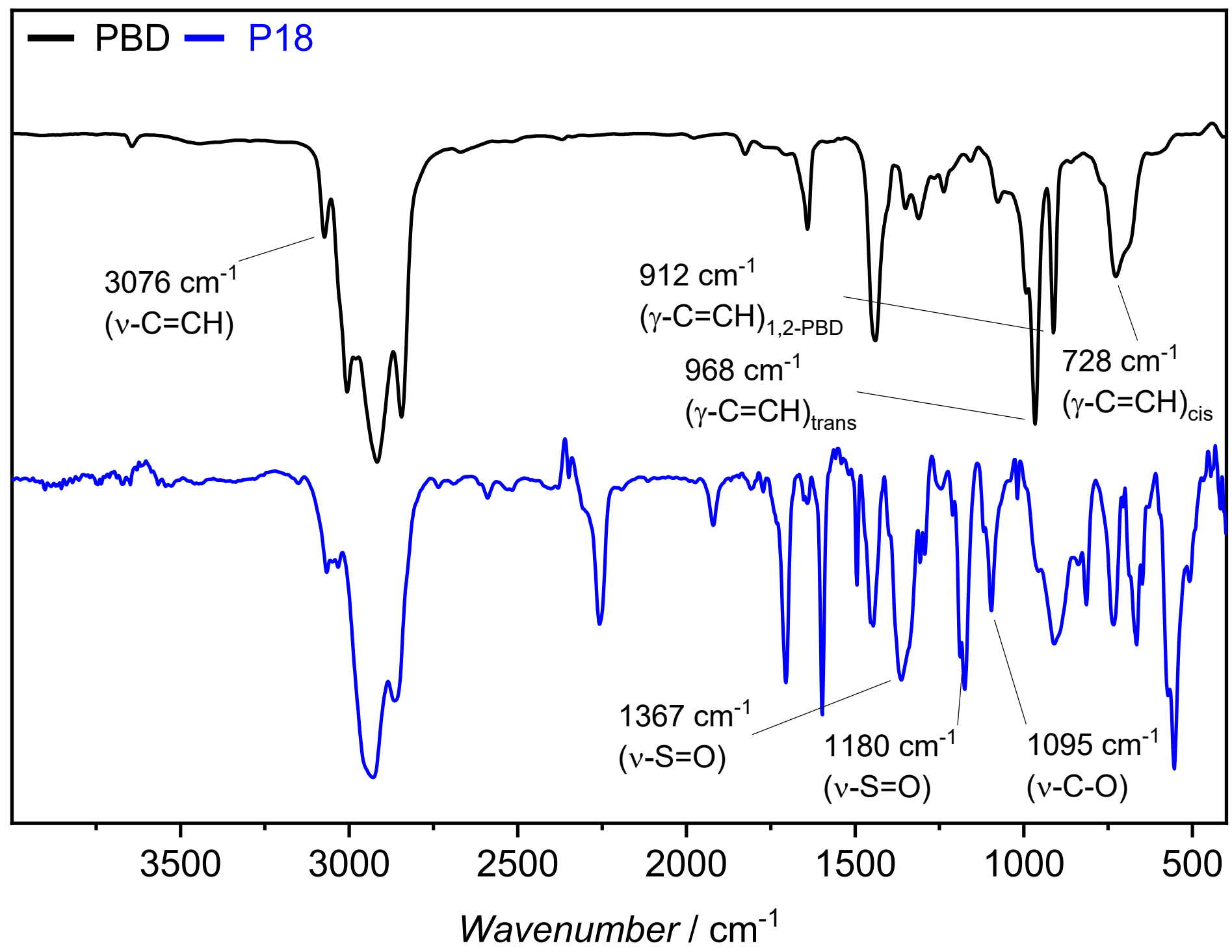

Figure S89. FTIR spectra of pristine PBD (black, top) and **P18** (blue, bottom) recorded in transmission mode as thin films on KBr plates.

C.3 SEC traces

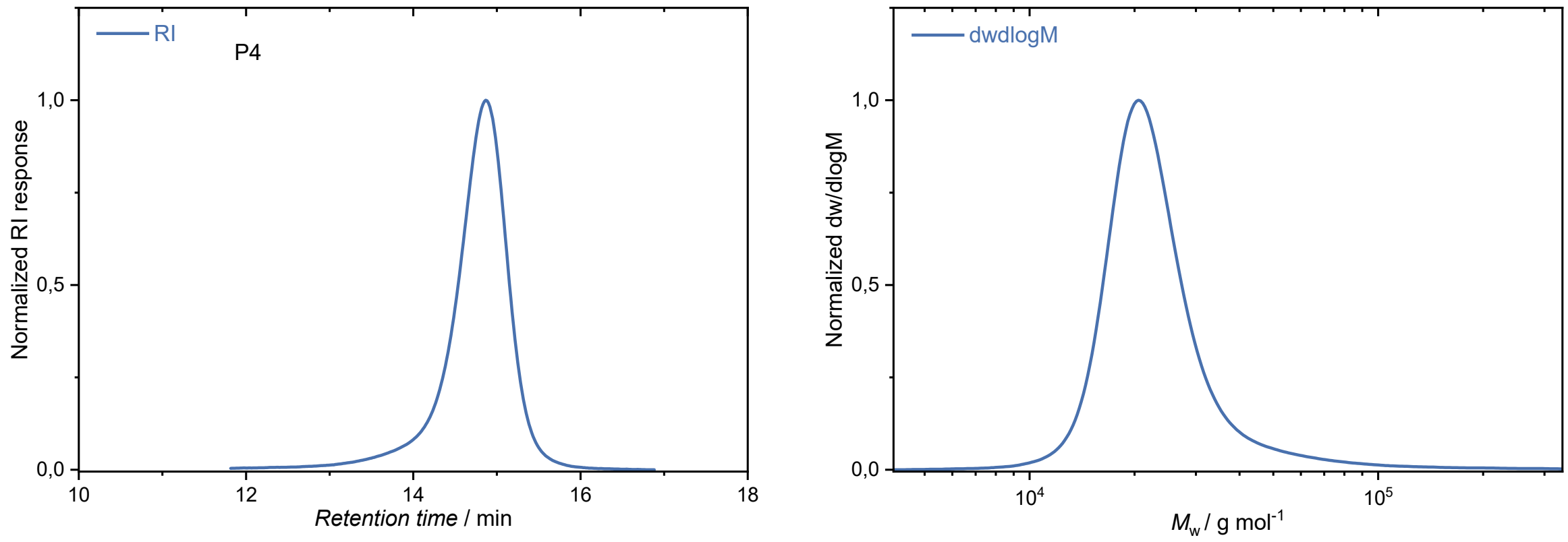

Figure S90. SEC analysis of **P4** recorded in THF, showing the normalized RI trace (left) and the corresponding molar mass distribution (right).

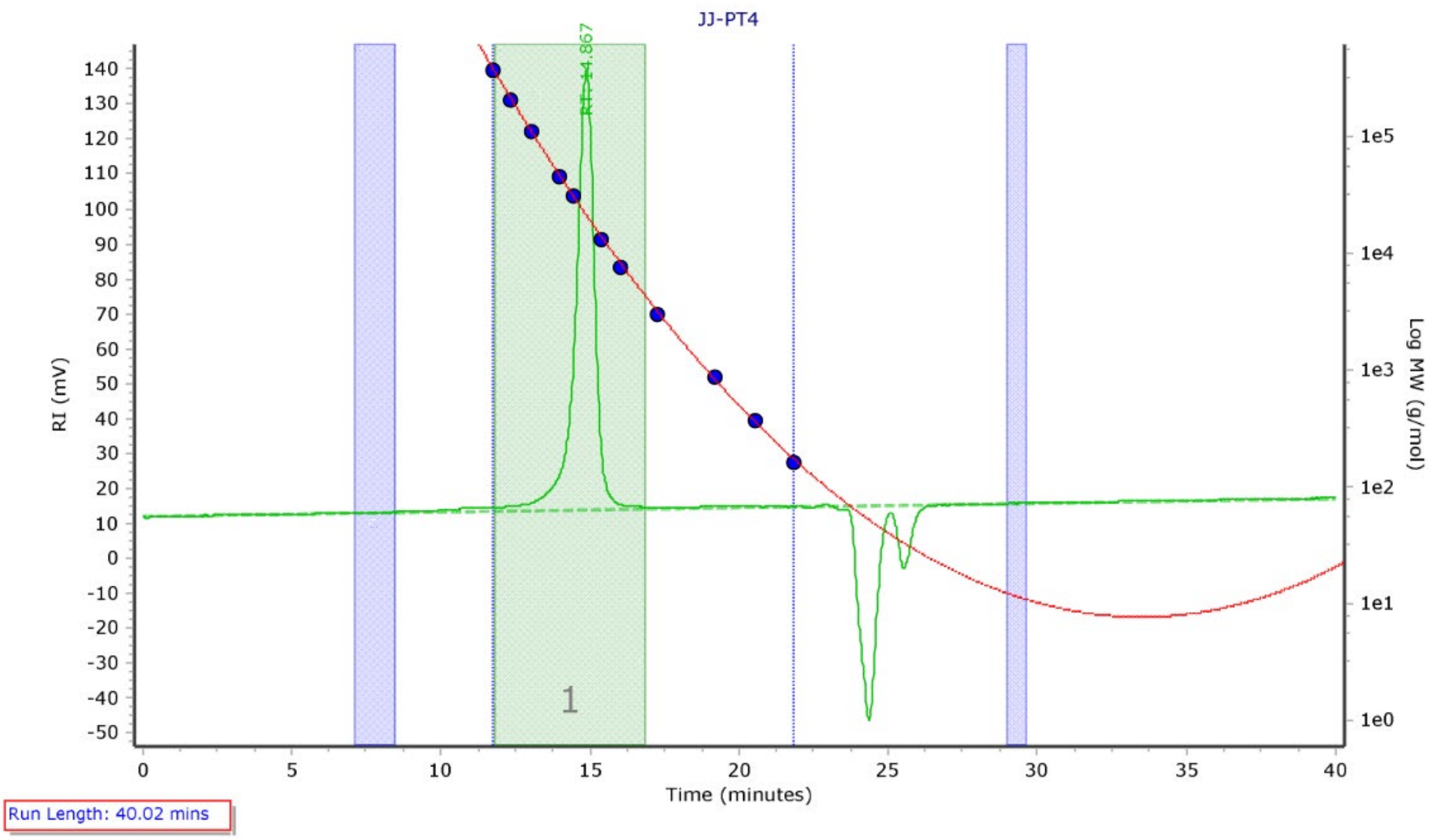

Figure S91. SEC chromatogram of **P4** in THF showing the RI trace (green), the integration region (green shaded area), and the corresponding polystyrene calibration curve (blue points; red fit).

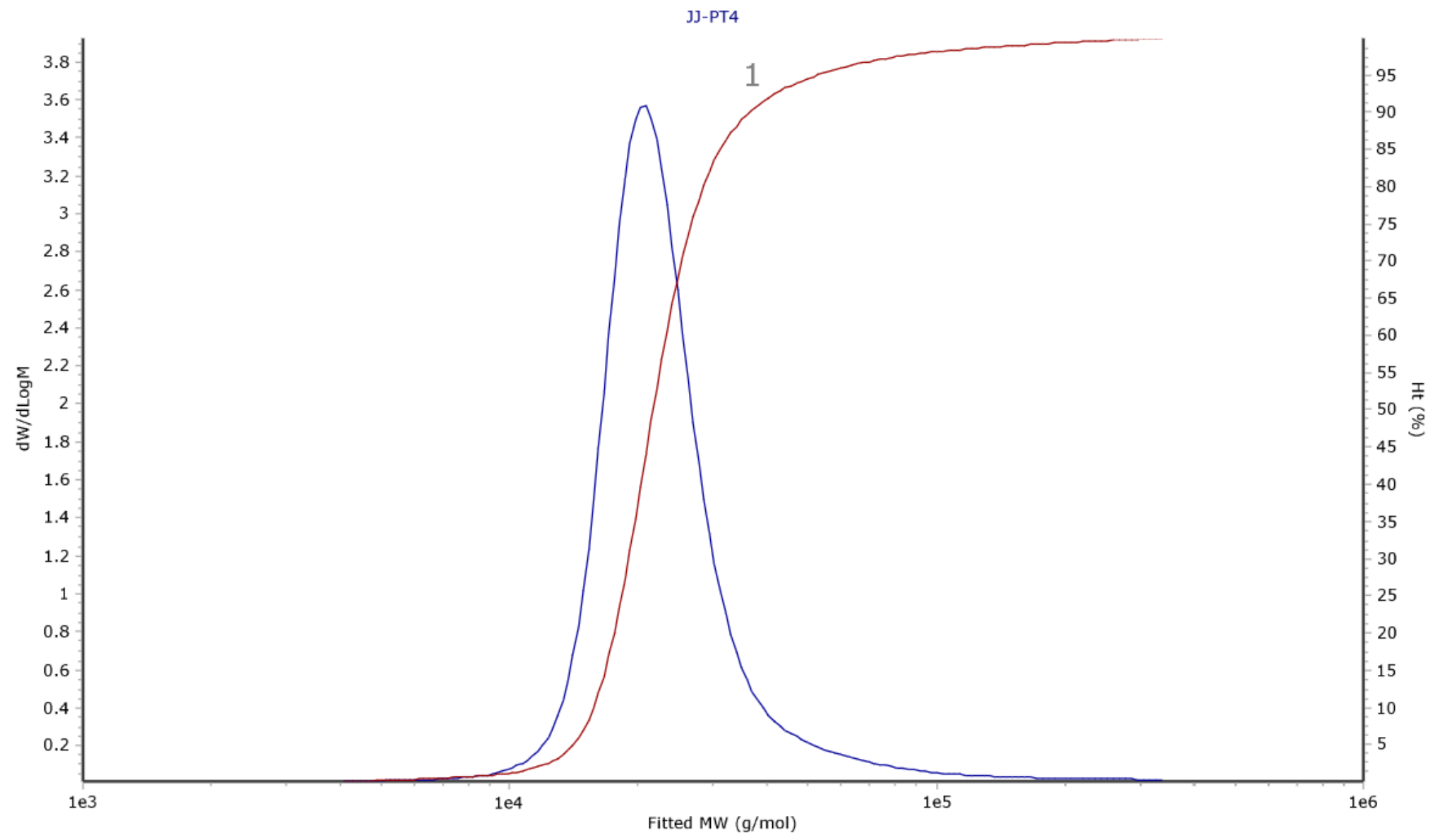

Figure S92. SEC-derived apparent molar-mass distribution of **P4** in THF, showing the differential distribution (blue) and cumulative distribution (red).

Table S2. SEC peak-integration parameters for **P4** in THF.

Peak Information

	Start (mins)	End (mins)
Baseline region 1	7.11667	8.48333
Baseline region 2	28.96667	29.65000
Peak 1	11.81667	16.90000

Peak Trace Information

Peak	Trace	Peak Max RT (mins)	Peak Area (mV.s)	Peak Height (mV)
Peak 1	RI	14.86667	5722.980	125.679
Peak 1	VS DP	14.86667	2785.436	37.600
Peak 1	VS IP	14.73333	35.444	0.999
Peak 1	UV 280nm	14.88333	7547.520	148.490

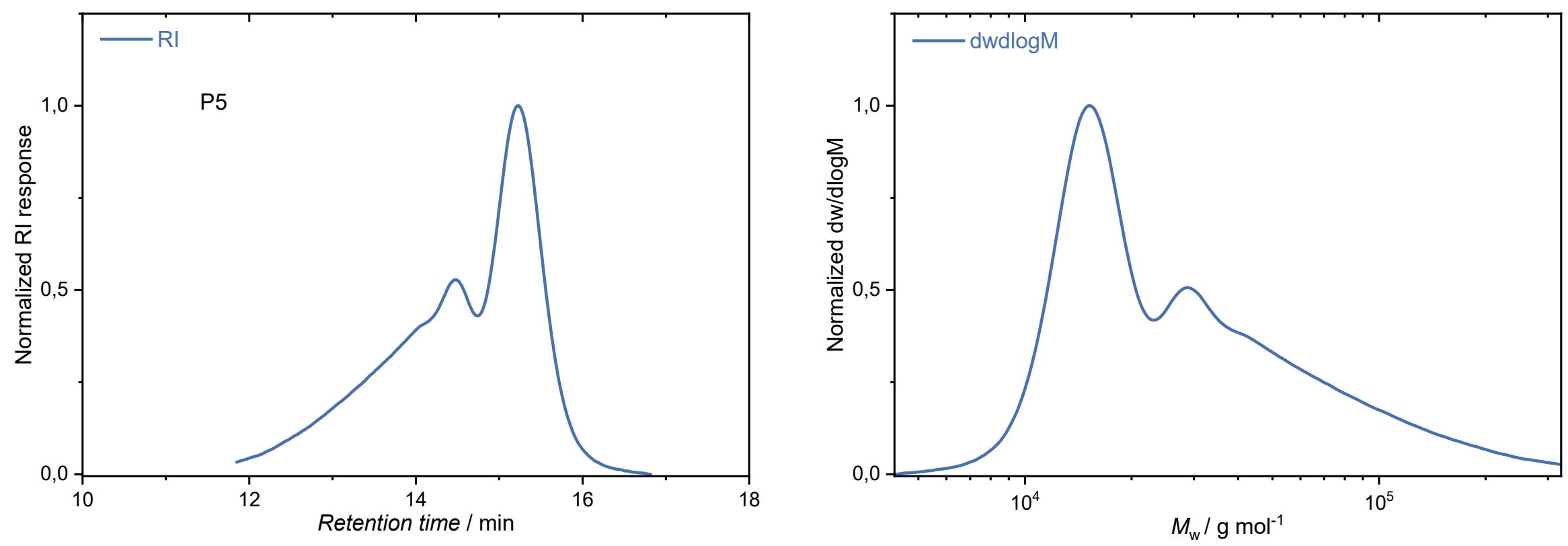

Figure S93. SEC analysis of **P5** recorded in THF, showing the normalized RI trace (left) and the corresponding molar mass distribution (right).

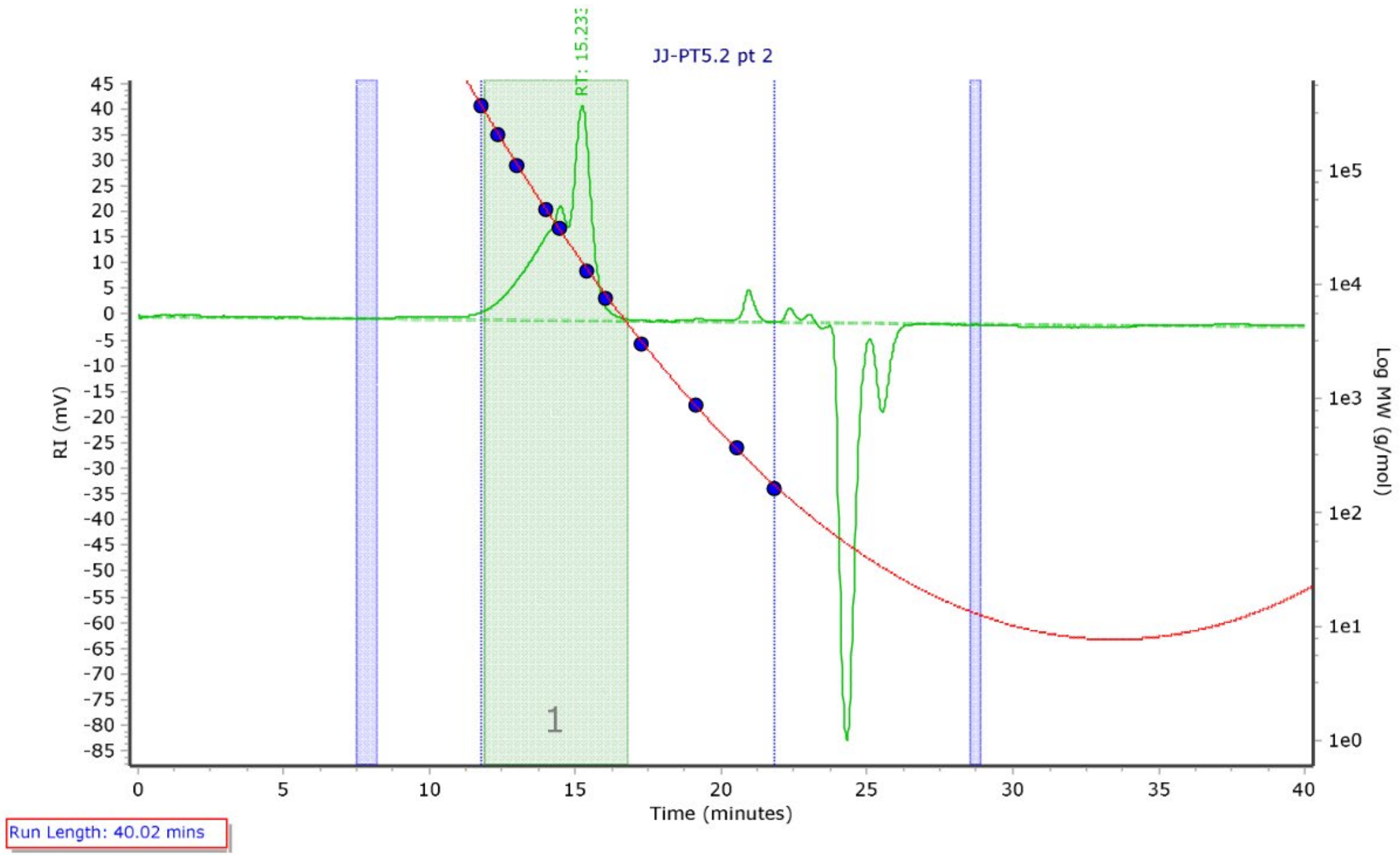

Figure S94. SEC chromatogram of **P5** in THF showing the RI trace (green), the integration region (green shaded area), and the corresponding polystyrene calibration curve (blue points; red fit).

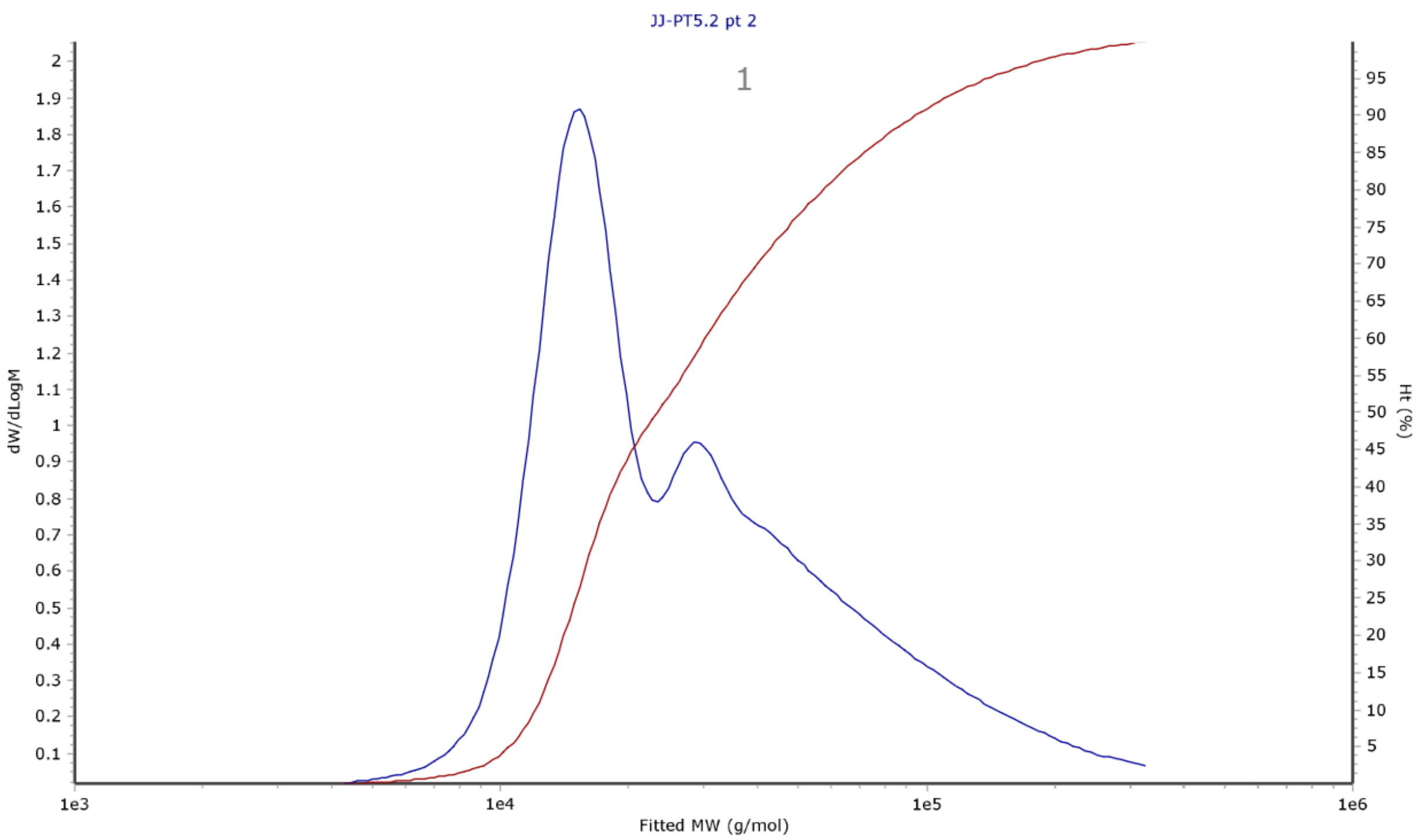

Figure S95. SEC-derived apparent molar-mass distribution of **P5** in THF, showing the differential distribution (blue) and cumulative distribution (red).

Table S3. SEC peak-integration parameters for **P5** in THF.

Peak Information

	Start (mins)	End (mins)
Baseline region 1	7.46667	8.23333
Baseline region 2	28.56667	28.95000
Peak 1	11.85000	16.83333

Peak Trace Information

Peak	Trace	Peak Max RT (mins)	Peak Area (mV.s)	Peak Height (mV)
Peak 1	RI	15.23333	3714.591	41.830
Peak 1	VS DP	15.21667	2235.319	13.997
Peak 1	VS IP	15.00000	33.502	0.352
Peak 1	UV 280nm	15.23333	976.845	8.730

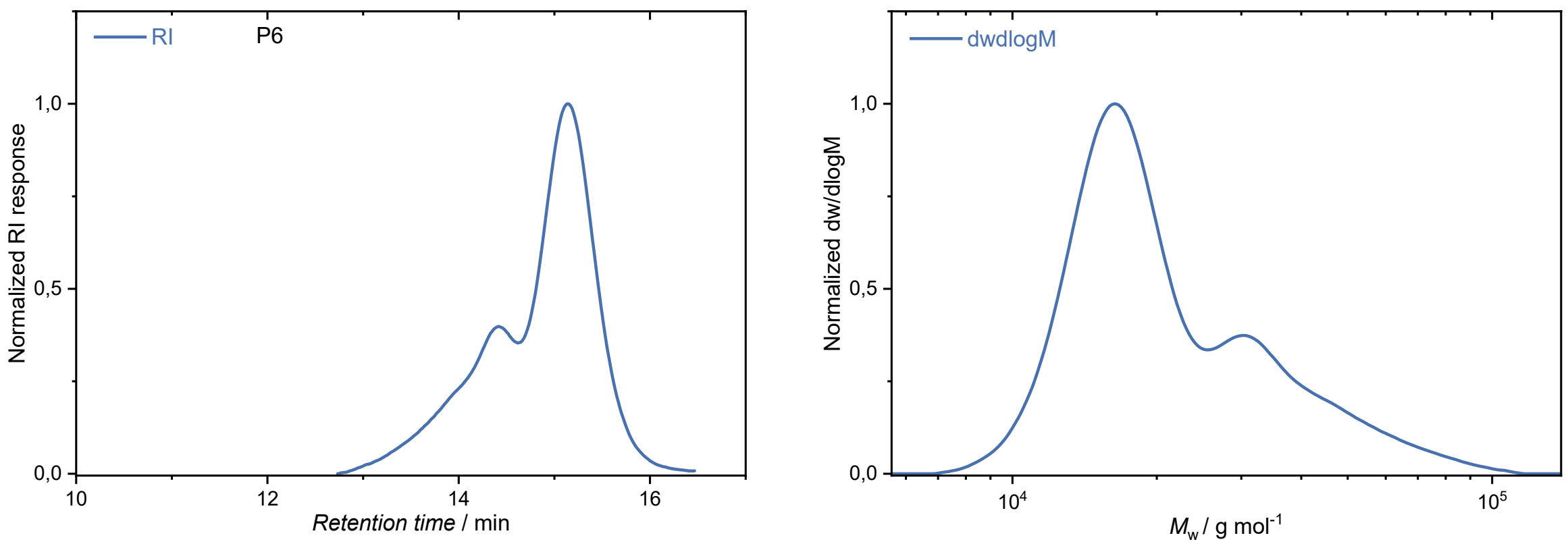

Figure S96. SEC analysis of **P6** recorded in THF, showing the normalized RI trace (left) and the corresponding molar mass distribution (right).

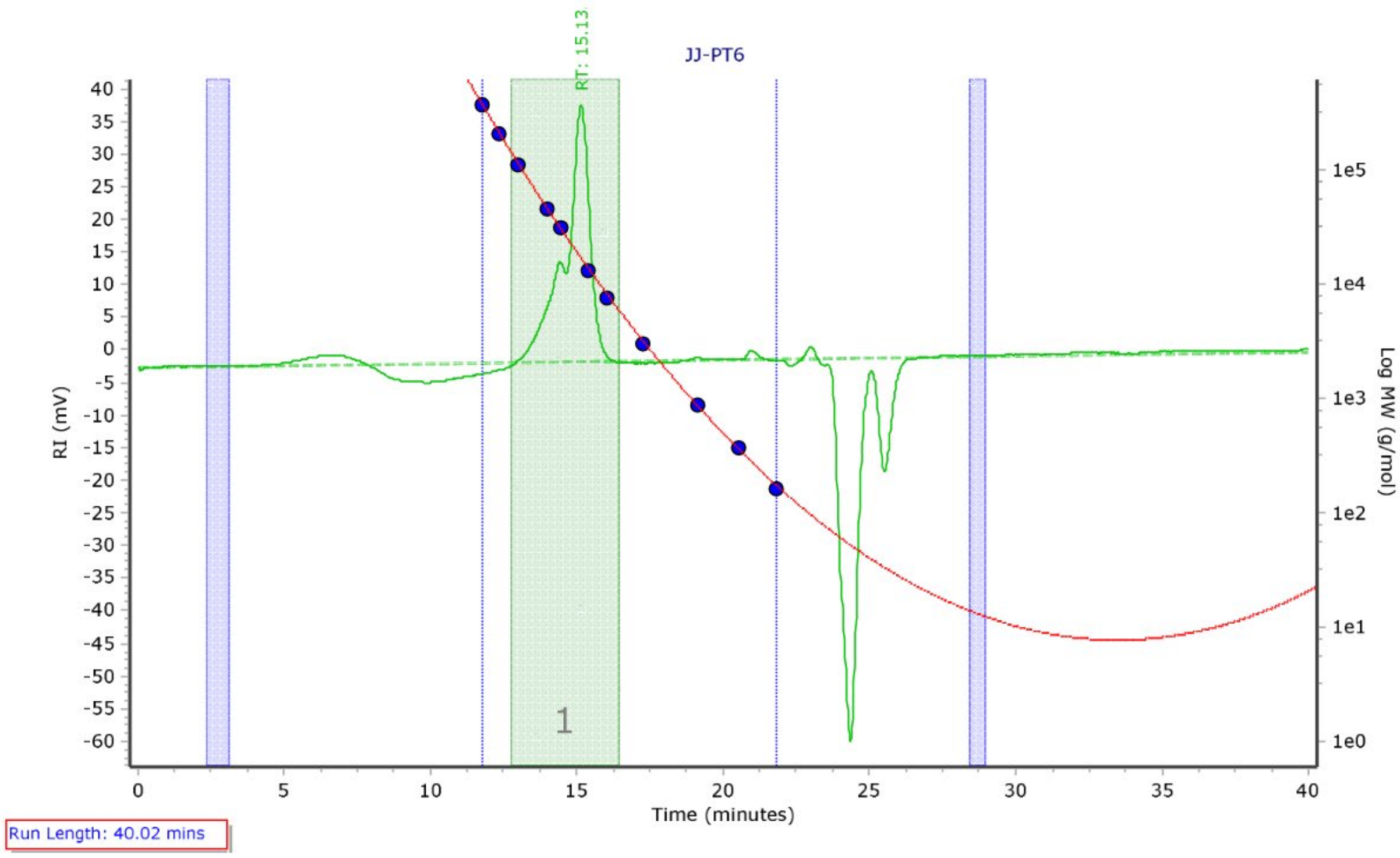

Figure S97. SEC chromatogram of **P6** in THF showing the RI trace (green), the integration region (green shaded area), and the corresponding polystyrene calibration curve (blue points; red fit).

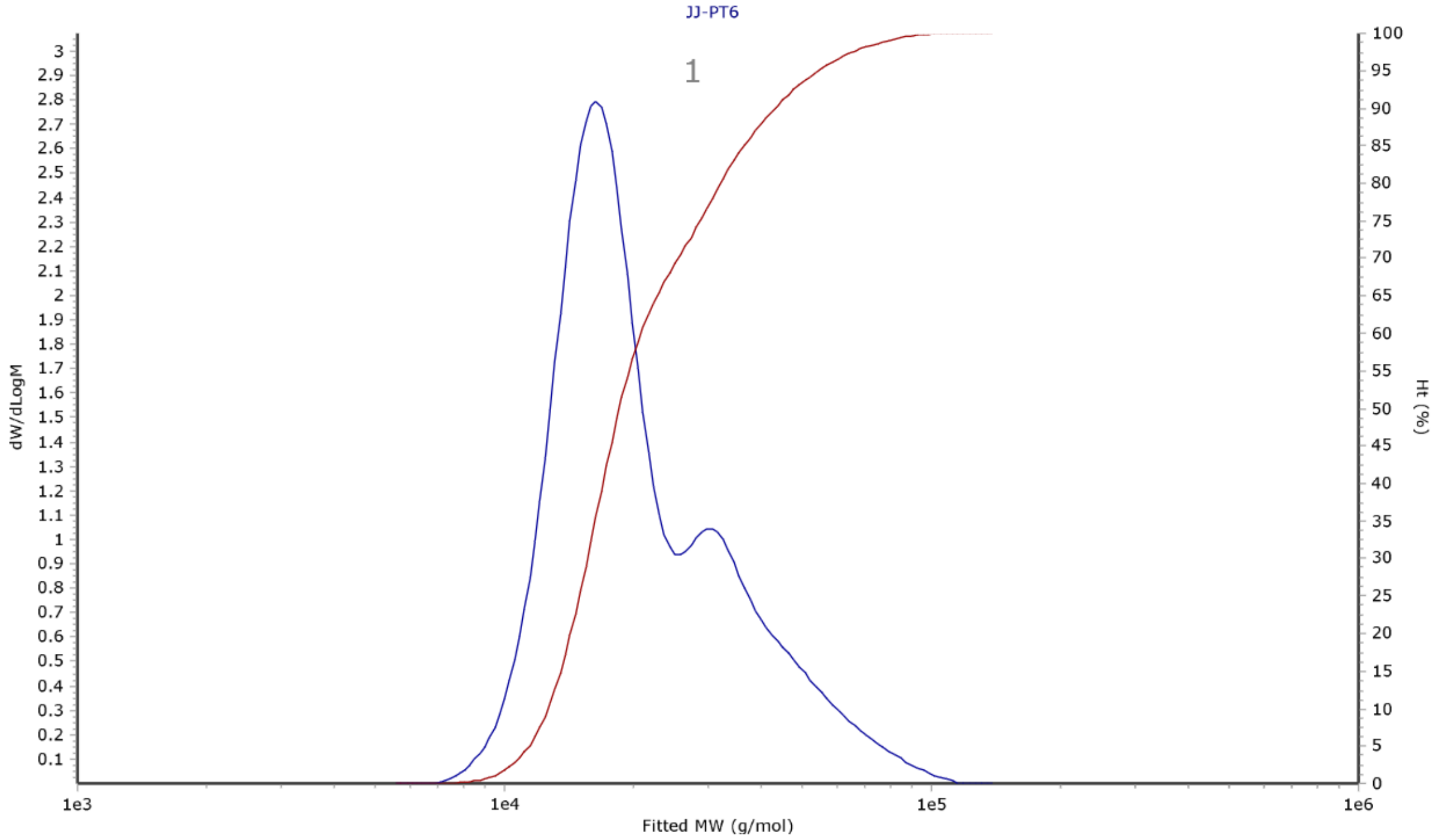

Figure S98. SEC-derived apparent molar-mass distribution of **P6** in THF, showing the differential distribution (blue) and cumulative distribution (red).

Table S4. SEC peak-integration parameters for **P6** in THF.

Peak Information

	Start (mins)	End (mins)
Baseline region 1	2.33333	3.16667
Baseline region 2	28.40000	29.01667
Peak 1	12.73333	16.48333

Peak Trace Information

Peak	Trace	Peak Max RT (mins)	Peak Area (mV.s)	Peak Height (mV)
Peak 1	RI	15.13333	2323.273	39.300
Peak 1	VS DP	15.15000	1173.284	10.644
Peak 1	VS IP	16.08333	14.885	-0.259
Peak 1	UV 280nm	15.15000	1071.607	16.451

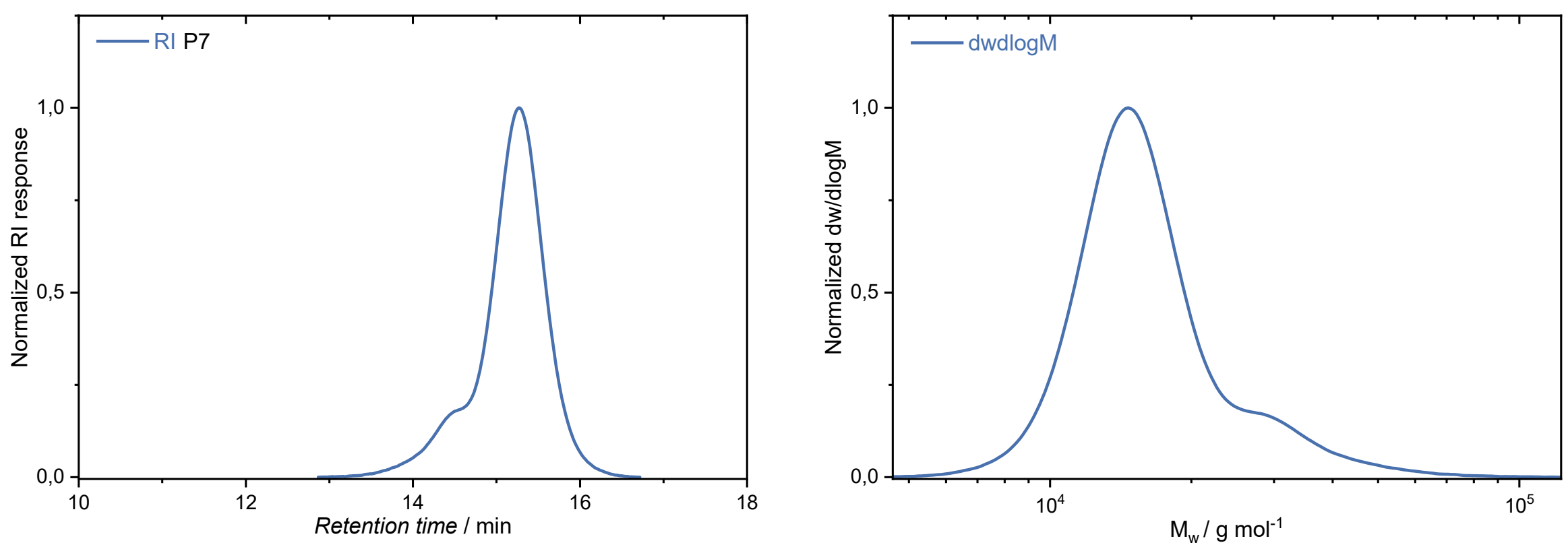

Figure S99. SEC analysis of **P7** recorded in THF, showing the normalized RI trace (left) and the corresponding molar mass distribution (right).

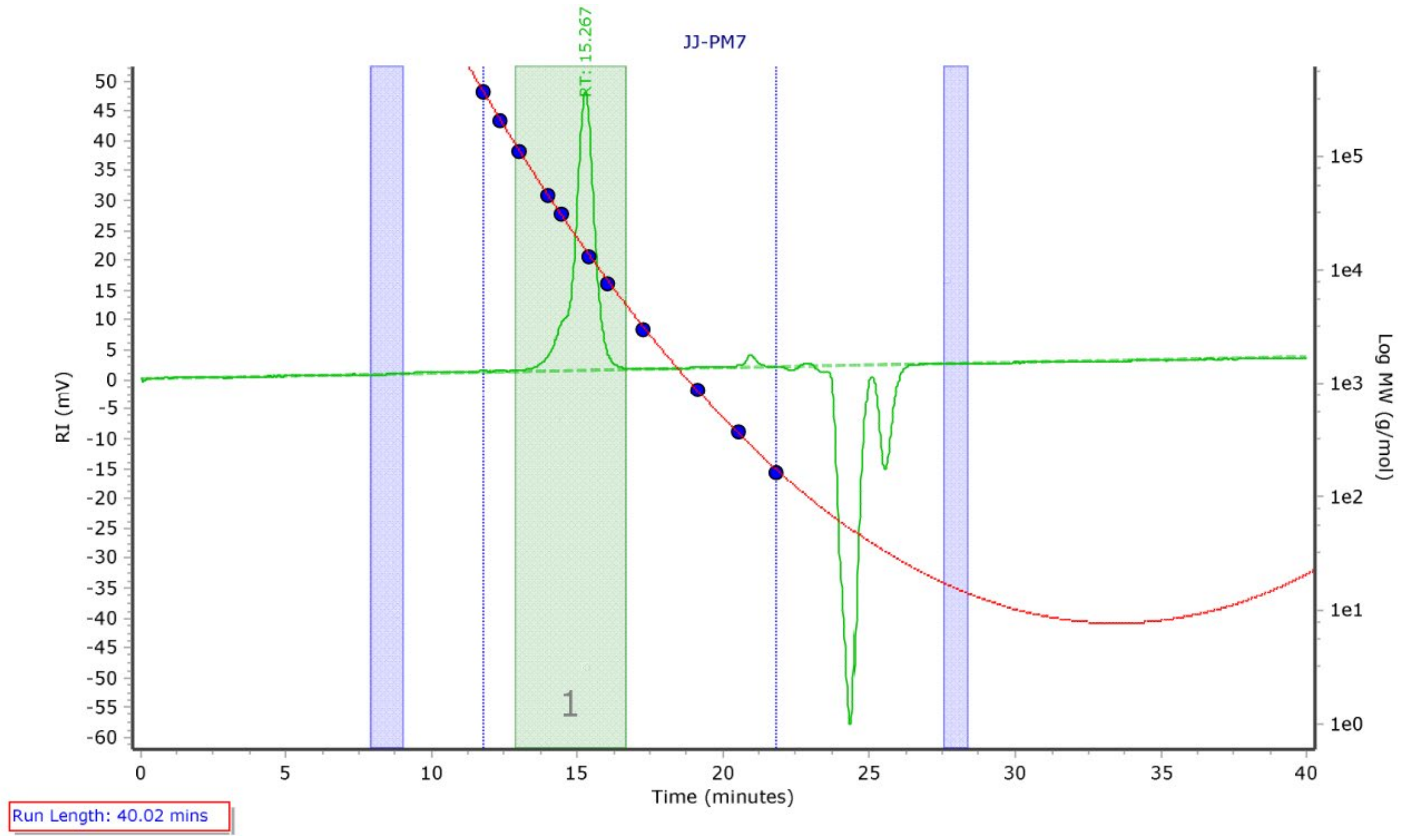

Figure S100. SEC chromatogram of **P7** in THF showing the RI trace (green), the integration region (green shaded area), and the corresponding polystyrene calibration curve (blue points; red fit).

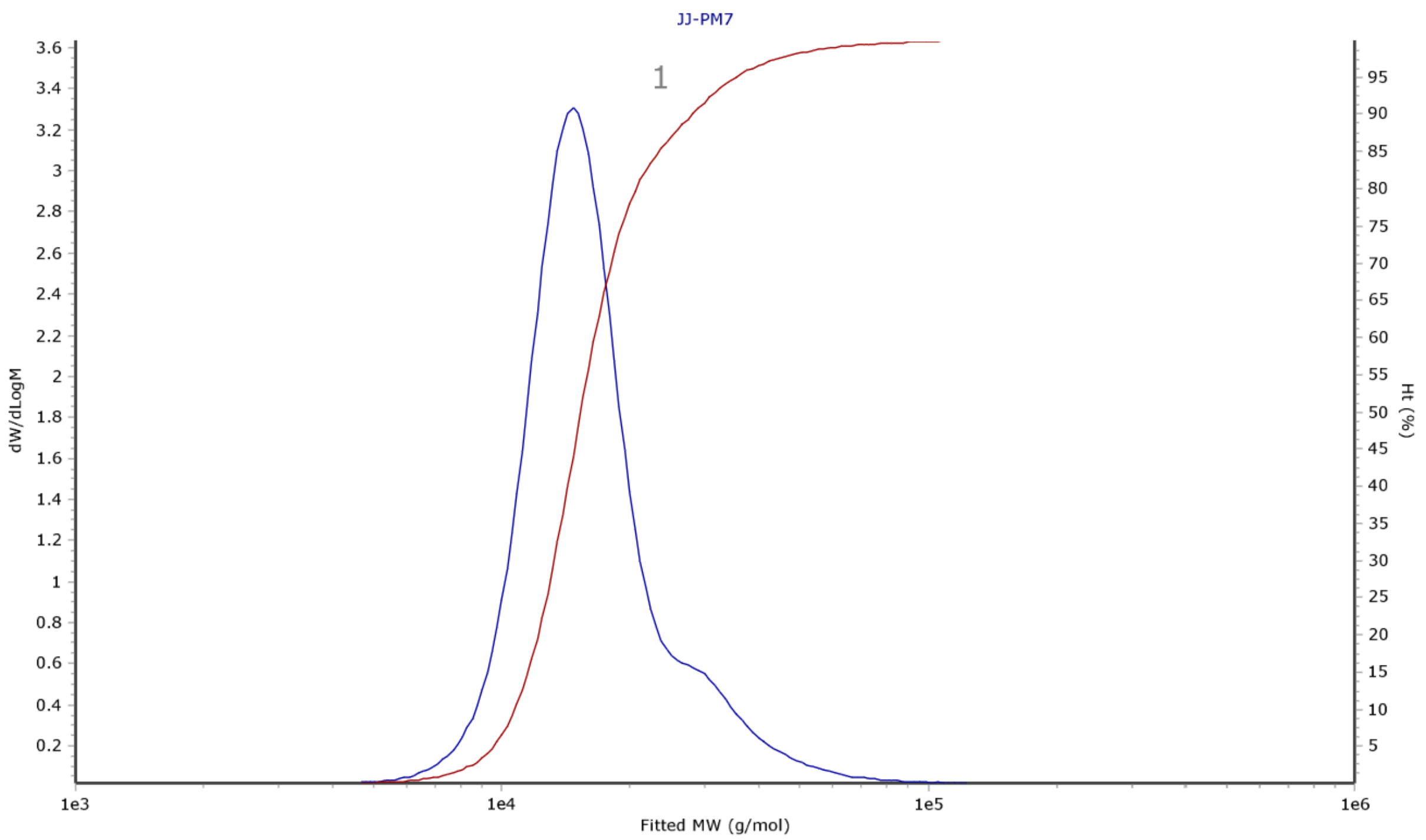

Figure S101. SEC-derived apparent molar-mass distribution of **P7** in THF, showing the differential distribution (blue) and cumulative distribution (red).

Table S5. SEC peak-integration parameters for **P7** in THF.

Peak Information

	Start (mins)	End (mins)
Baseline region 1	7.88333	9.08333
Baseline region 2	27.53333	28.40000
Peak 1	12.86667	16.73333

Peak Trace Information

Peak	Trace	Peak Max RT (mins)	Peak Area (mV.s)	Peak Height (mV)
Peak 1	RI	15.26667	2346.369	46.635
Peak 1	VS DP	15.26667	779.094	11.317
Peak 1	VS IP	15.16667	44.397	0.545
Peak 1	UV 280nm	15.28333	1539.166	28.571

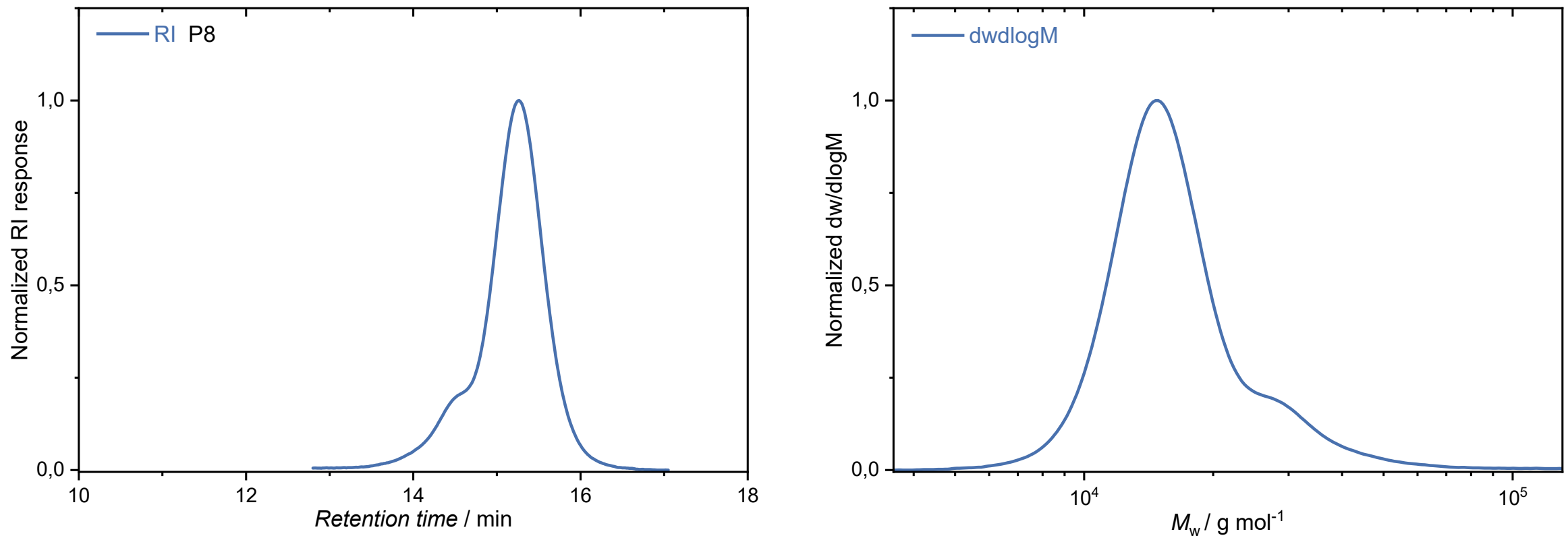

Figure S102. SEC analysis of **P8** recorded in THF, showing the normalized RI trace (left) and the corresponding molar mass distribution (right).

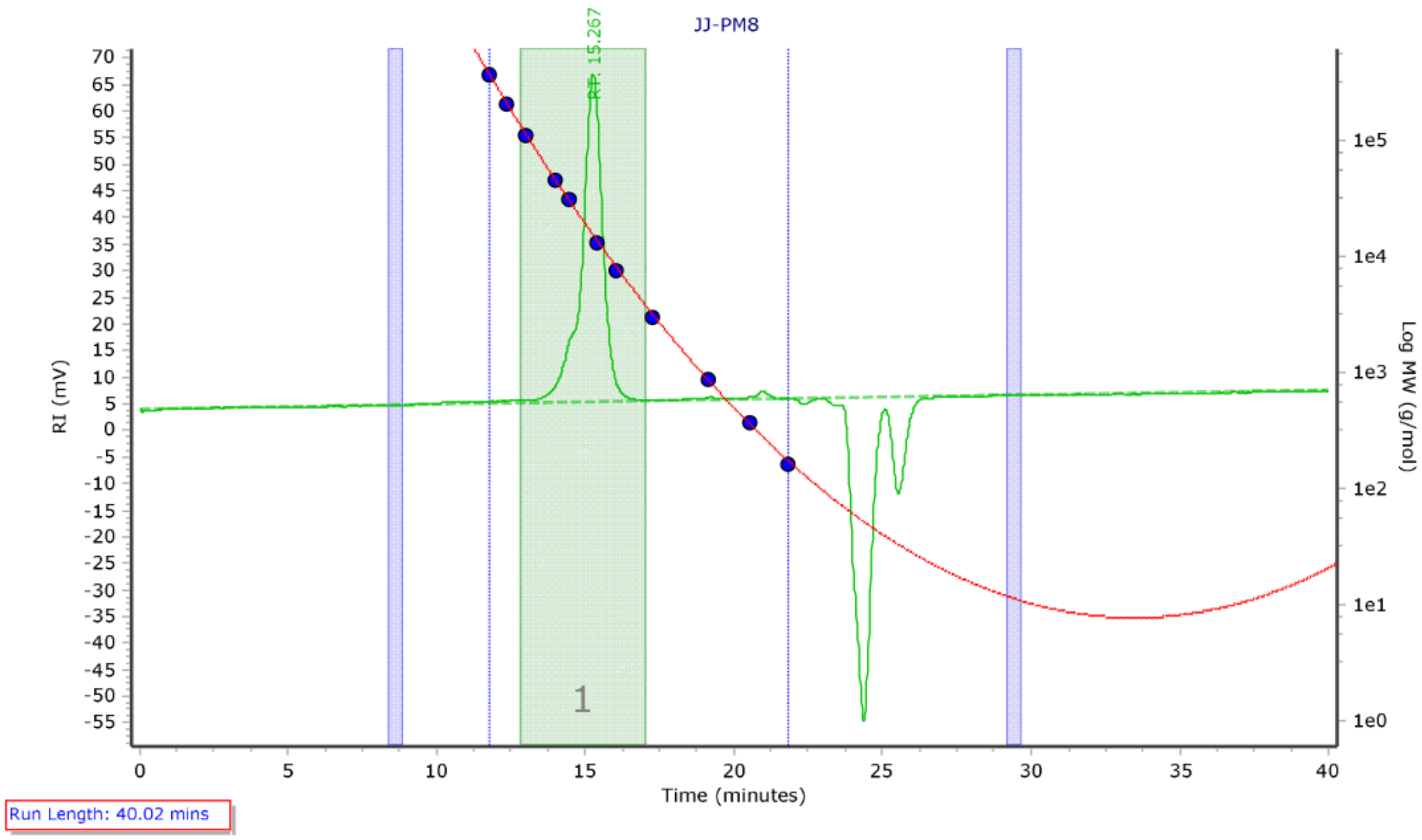

Figure S103. SEC chromatogram of **P8** in THF showing the RI trace (green), the integration region (green shaded area), and the corresponding polystyrene calibration curve (blue points; red fit).

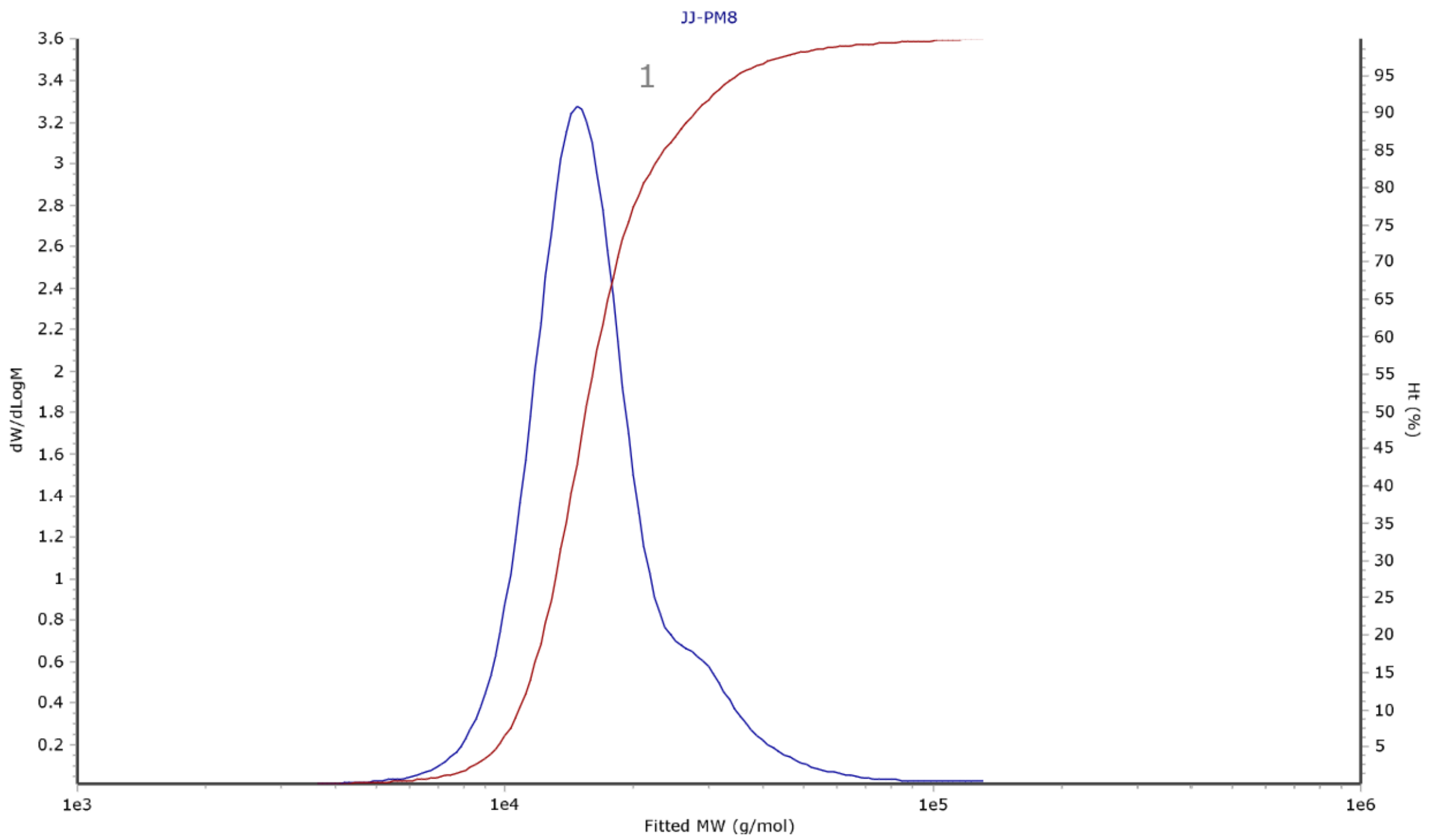

Figure S104. SEC-derived apparent molar-mass distribution of **P8** in THF, showing the differential distribution (blue) and cumulative distribution (red).

Table S6. SEC peak-integration parameters for **P8** in THF.

Peak Information

	Start (mins)	End (mins)
Baseline region 1	8.36667	8.91667
Baseline region 2	29.18333	29.73333
Peak 1	12.80000	17.06667

Peak Trace Information

Peak	Trace	Peak Max RT (mins)	Peak Area (mV.s)	Peak Height (mV)
Peak 1	RI	15.26667	3120.480	61.512
Peak 1	VS DP	15.26667	1181.372	14.920
Peak 1	VS IP	15.26667	18.526	0.454
Peak 1	UV 280nm	15.25000	2008.321	36.333

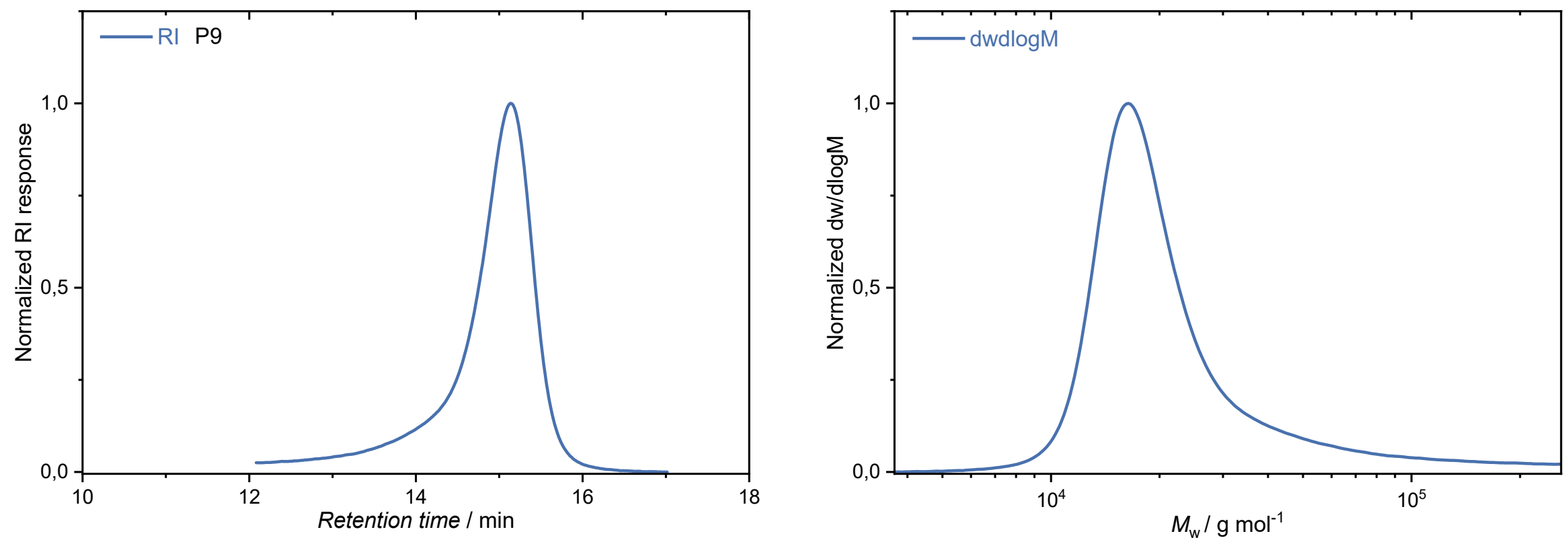

Figure S105. SEC analysis of **P9** recorded in THF, showing the normalized RI trace (left) and the corresponding molar mass distribution (right).

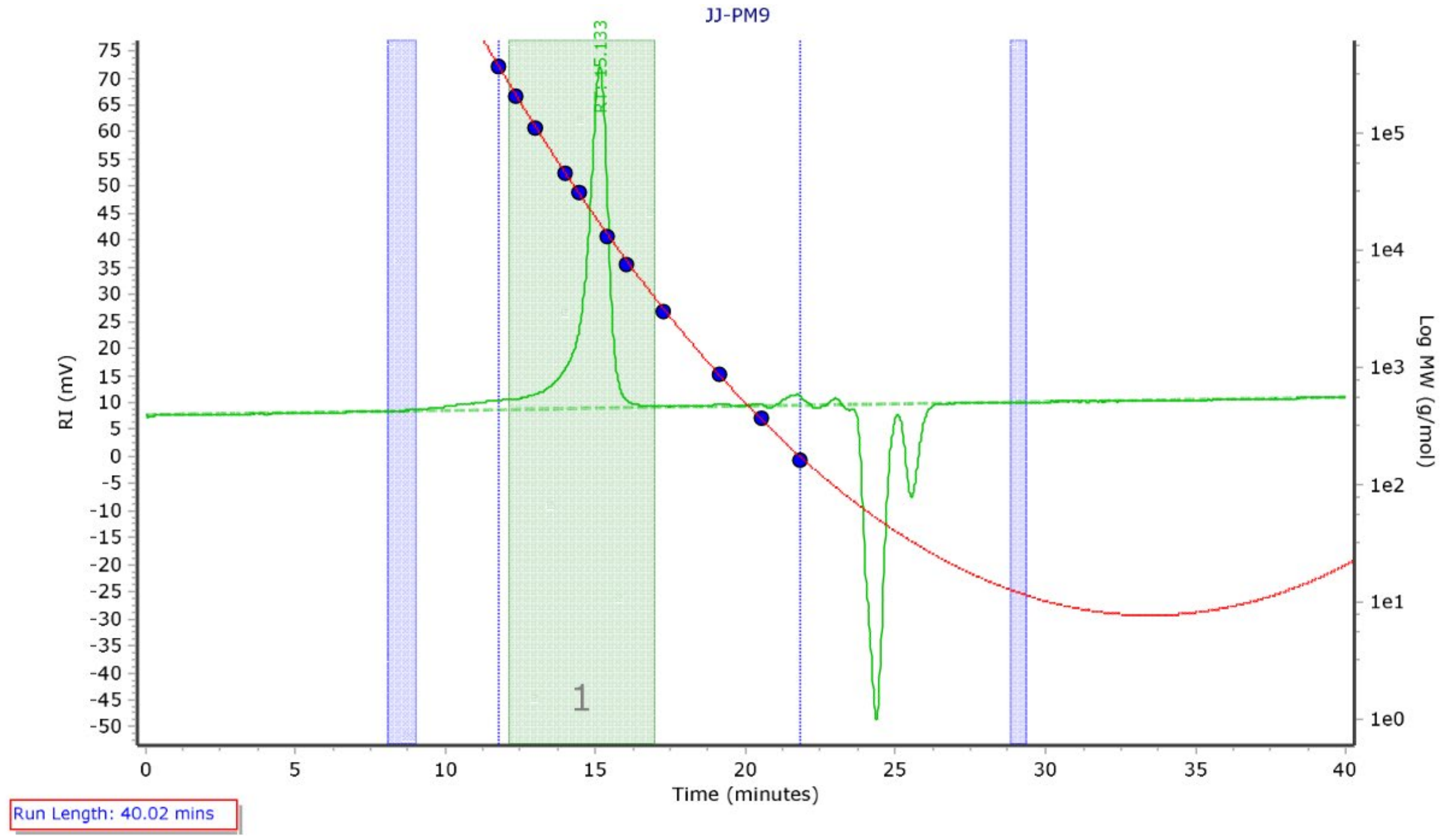

Figure S106. SEC chromatogram of **P9** in THF showing the RI trace (green), the integration region (green shaded area), and the corresponding polystyrene calibration curve (blue points; red fit).

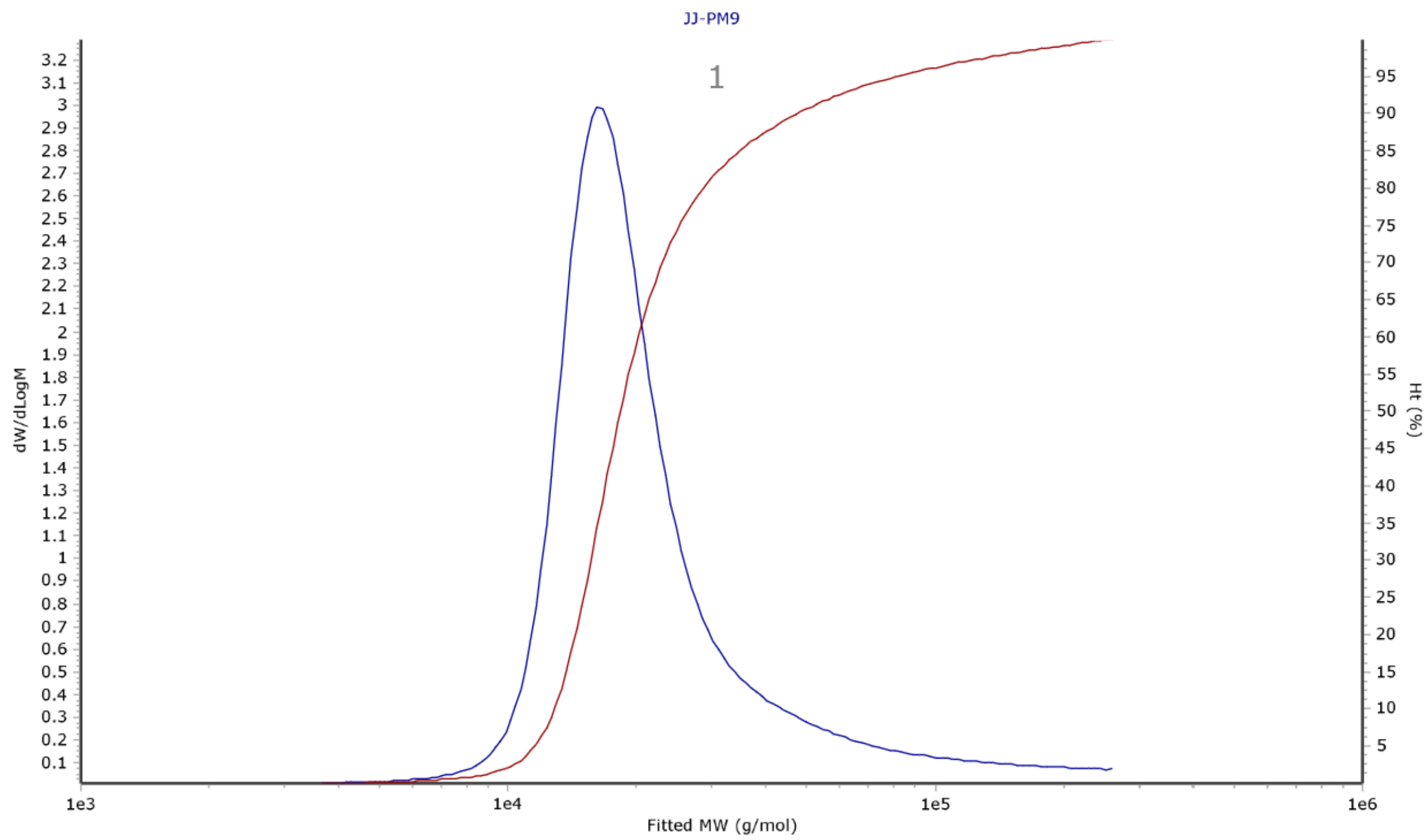

Figure S107. SEC-derived apparent molar-mass distribution of **P9** in THF, showing the differential distribution (blue) and cumulative distribution (red).

Table S7. SEC peak-integration parameters for **P9** in THF.

Peak Information

	Start (mins)	End (mins)
Baseline region 1	8.08333	9.05000
Baseline region 2	28.81667	29.40000
Peak 1	12.08333	17.03333

Peak Trace Information

Peak	Trace	Peak Max RT (mins)	Peak Area (mV.s)	Peak Height (mV)
Peak 1	RI	15.13333	3481.002	63.127
Peak 1	VS DP	15.13333	1223.870	14.587
Peak 1	VS IP	14.91667	24.711	0.498
Peak 1	UV 280nm	15.15000	63991.134	1010.338

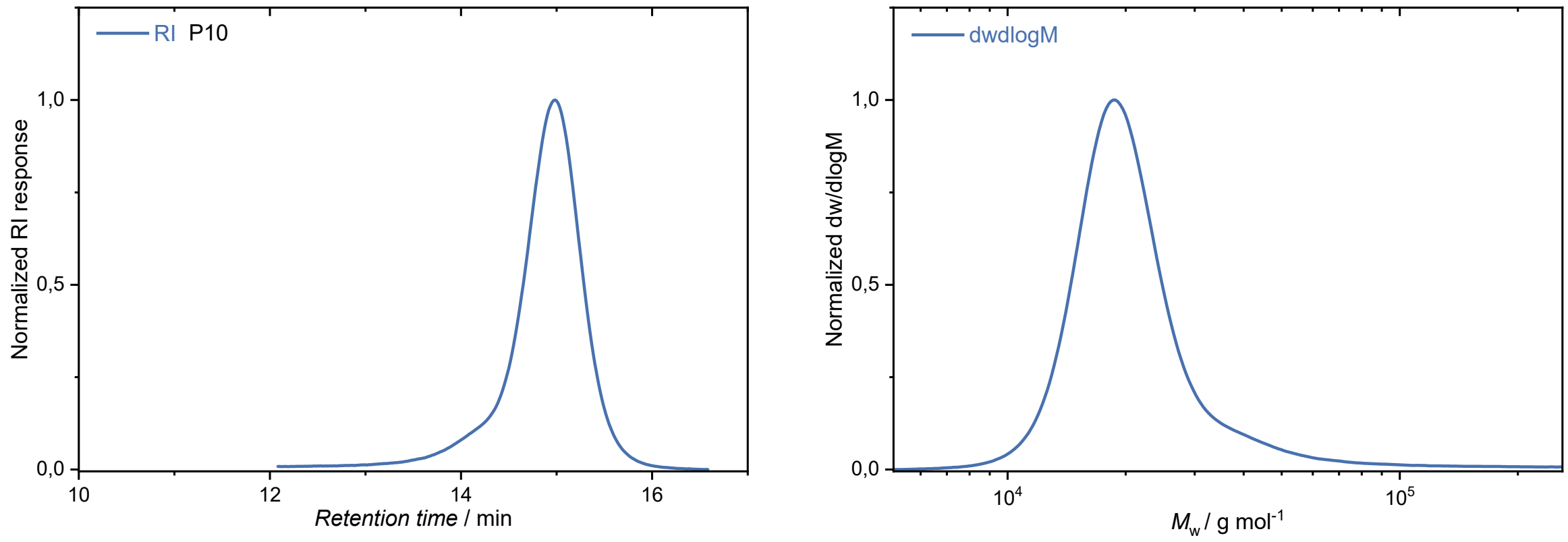

Figure S108. SEC analysis of **P10** recorded in THF, showing the normalized RI trace (left) and the corresponding molar mass distribution (right).

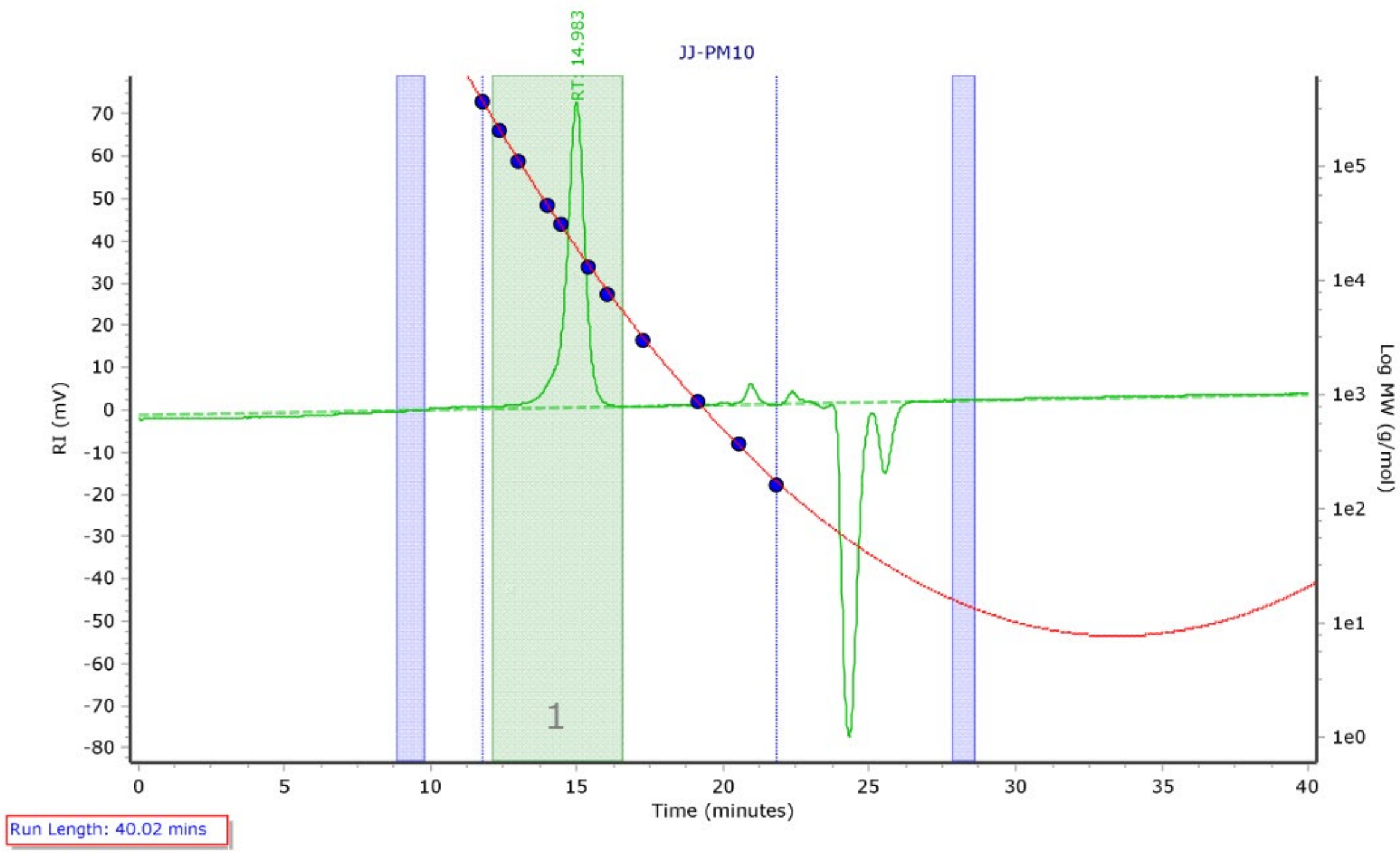

Figure S109. SEC chromatogram of **P10** in THF showing the RI trace (green), the integration region (green shaded area), and the corresponding polystyrene calibration curve (blue points; red fit).

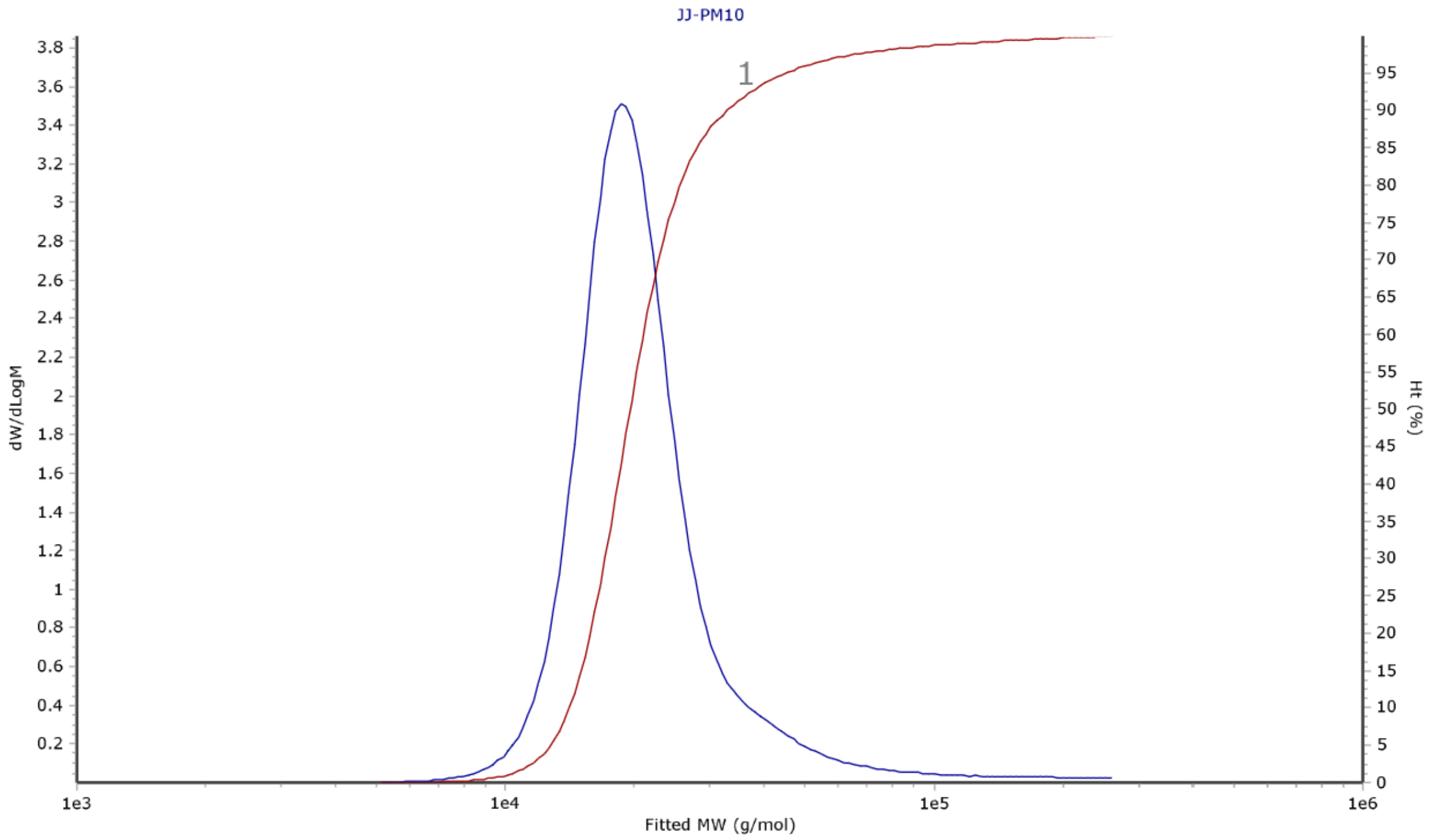

Figure S110. SEC-derived apparent molar-mass distribution of **P10** in THF, showing the differential distribution (blue) and cumulative distribution (red).

Table S8. SEC peak-integration parameters for **P10** in THF.

Peak Information

	Start (mins)	End (mins)
Baseline region 1	8.85000	9.81667
Baseline region 2	27.85000	28.68333
Peak 1	12.08333	16.60000

Peak Trace Information

Peak	Trace	Peak Max RT (mins)	Peak Area (mV.s)	Peak Height (mV)
Peak 1	RI	14.98333	3373.074	72.372
Peak 1	VS DP	14.98333	1109.628	19.243
Peak 1	VS IP	14.88333	22.636	0.672
Peak 1	UV 280nm	14.98333	5222.927	104.605

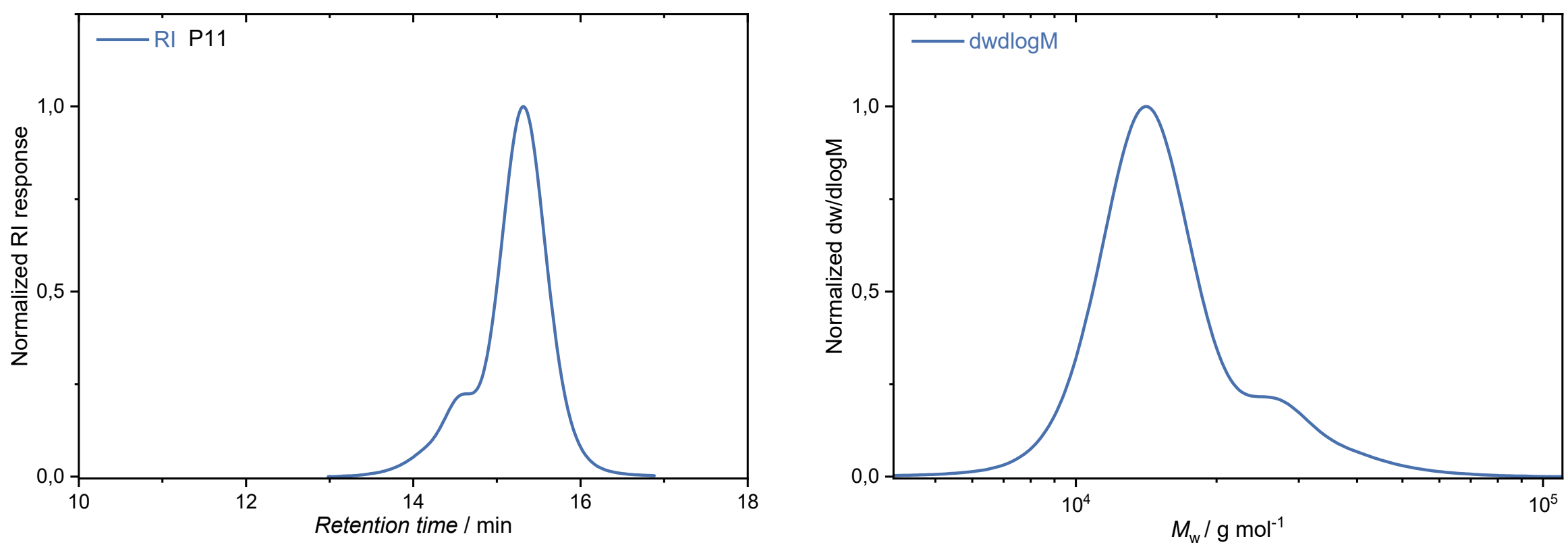

Figure S111. SEC analysis of **P11** recorded in THF, showing the normalized RI trace (left) and the corresponding molar mass distribution (right).

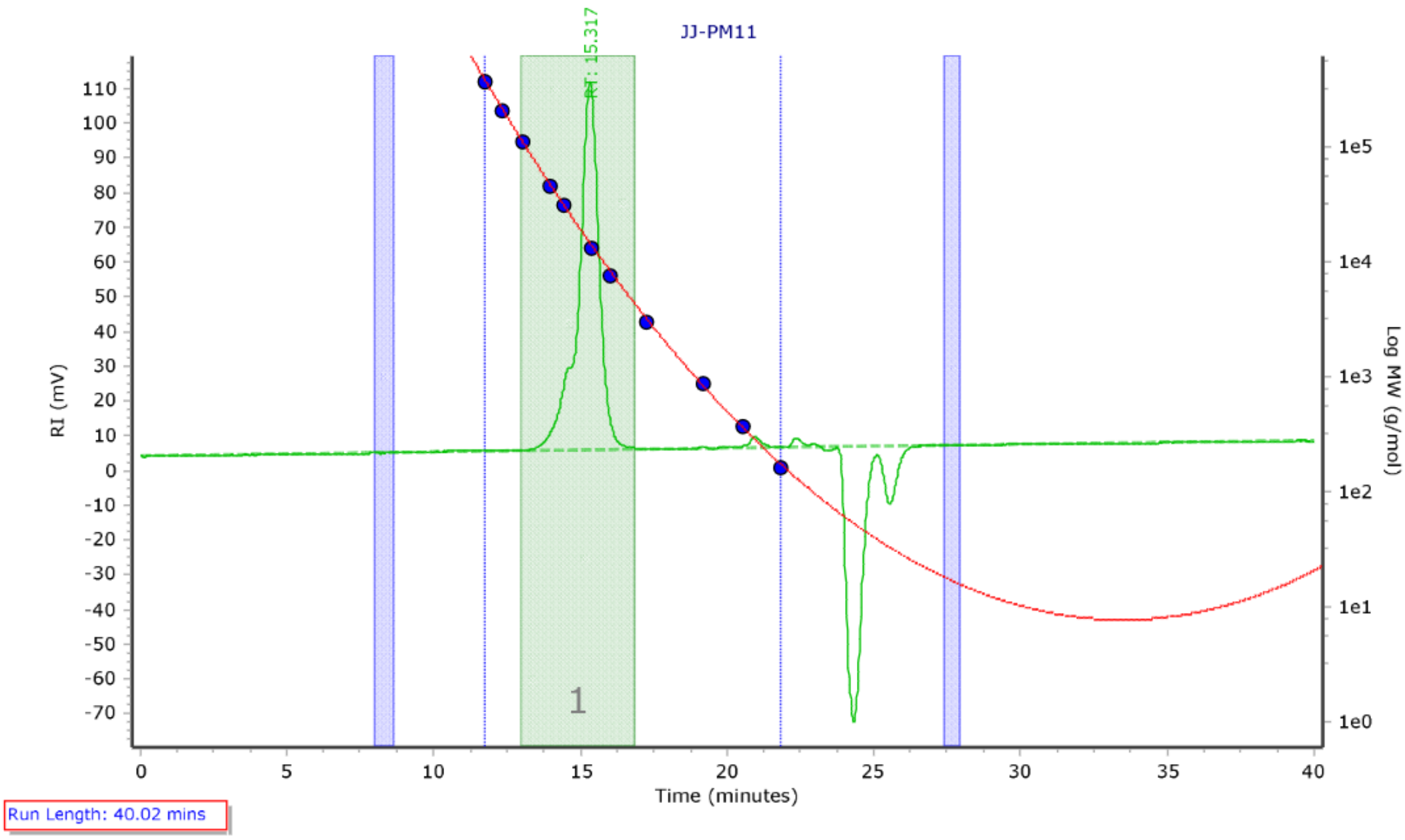

Figure S112. SEC chromatogram of **P11** in THF showing the RI trace (green), the integration region (green shaded area), and the corresponding polystyrene calibration curve (blue points; red fit).

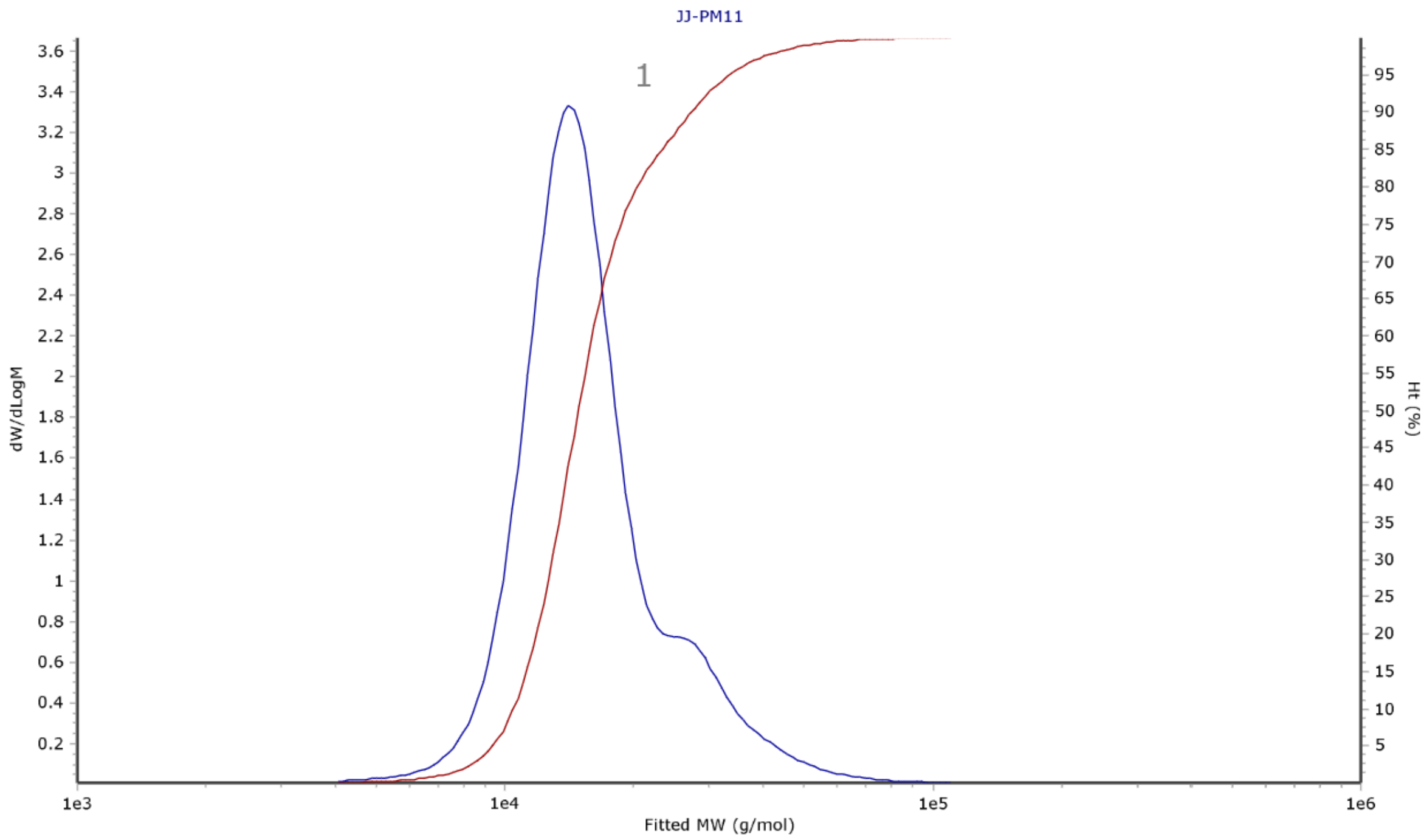

Figure S113. SEC-derived apparent molar-mass distribution of **P11** in THF, showing the differential distribution (blue) and cumulative distribution (red).

Table S9. SEC peak-integration parameters for **P11** in THF.

Peak Information

	Start (mins)	End (mins)
Baseline region 1	7.96667	8.66667
Baseline region 2	27.41667	28.00000
Peak 1	12.98333	16.90000

Peak Trace Information

Peak	Trace	Peak Max RT (mins)	Peak Area (mV.s)	Peak Height (mV)
Peak 1	RI	15.31667	5297.351	106.044
Peak 1	VS DP	15.31667	1609.703	28.532
Peak 1	VS IP	15.28333	46.626	1.025
Peak 1	UV 280nm	15.33333	689.645	12.581

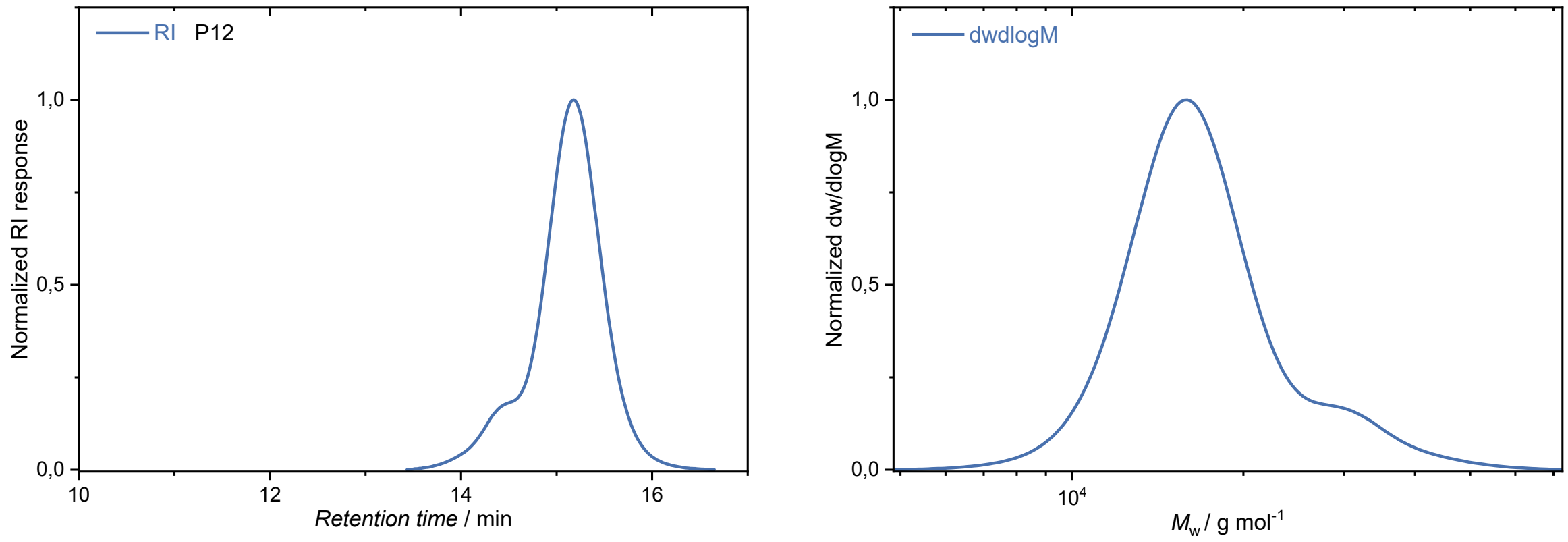

Figure S114. SEC analysis of **P12** recorded in THF, showing the normalized RI trace (left) and the corresponding molar mass distribution (right).

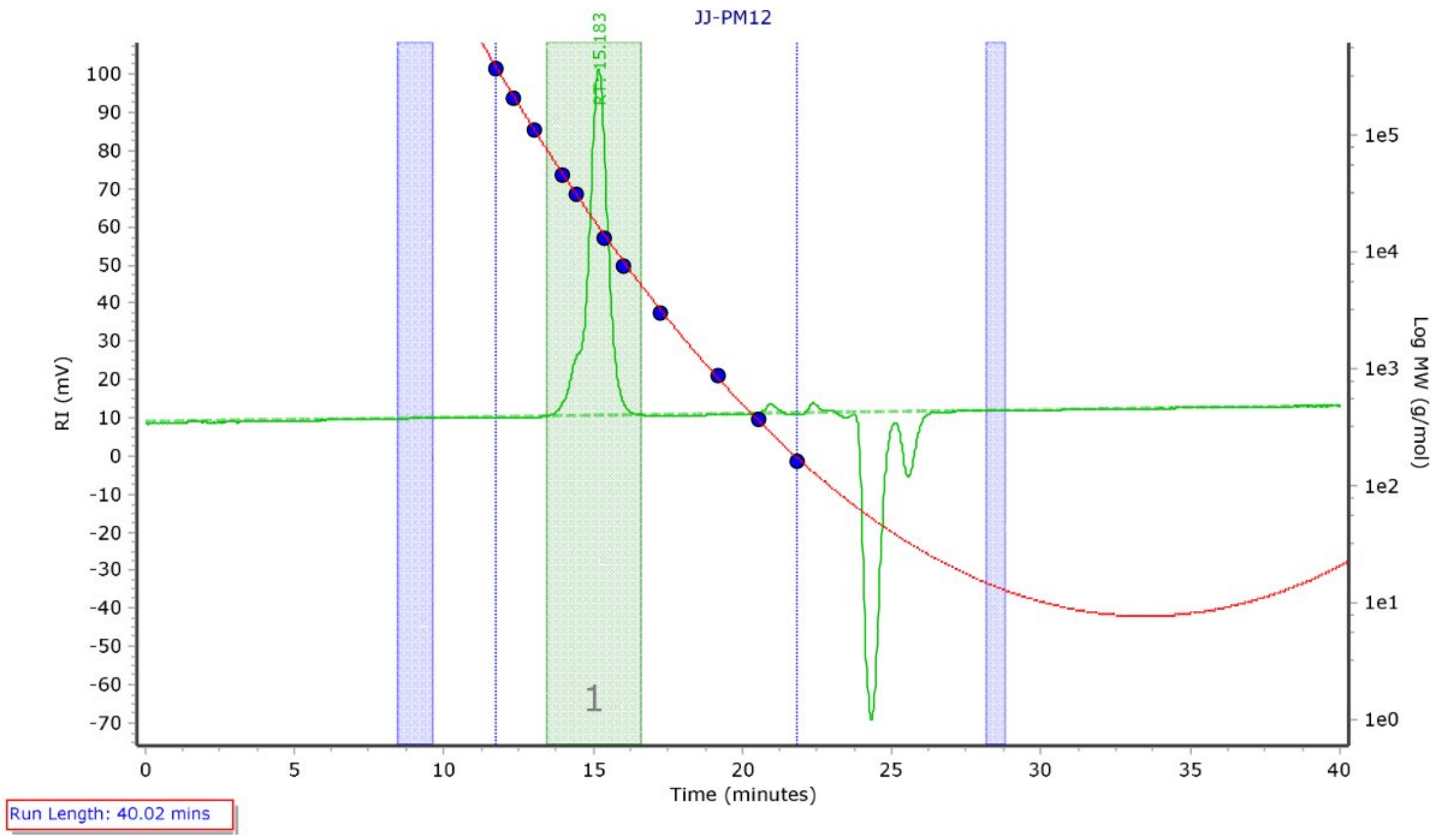

Figure S115. SEC chromatogram of **P12** in THF showing the RI trace (green), the integration region (green shaded area), and the corresponding polystyrene calibration curve (blue points; red fit).

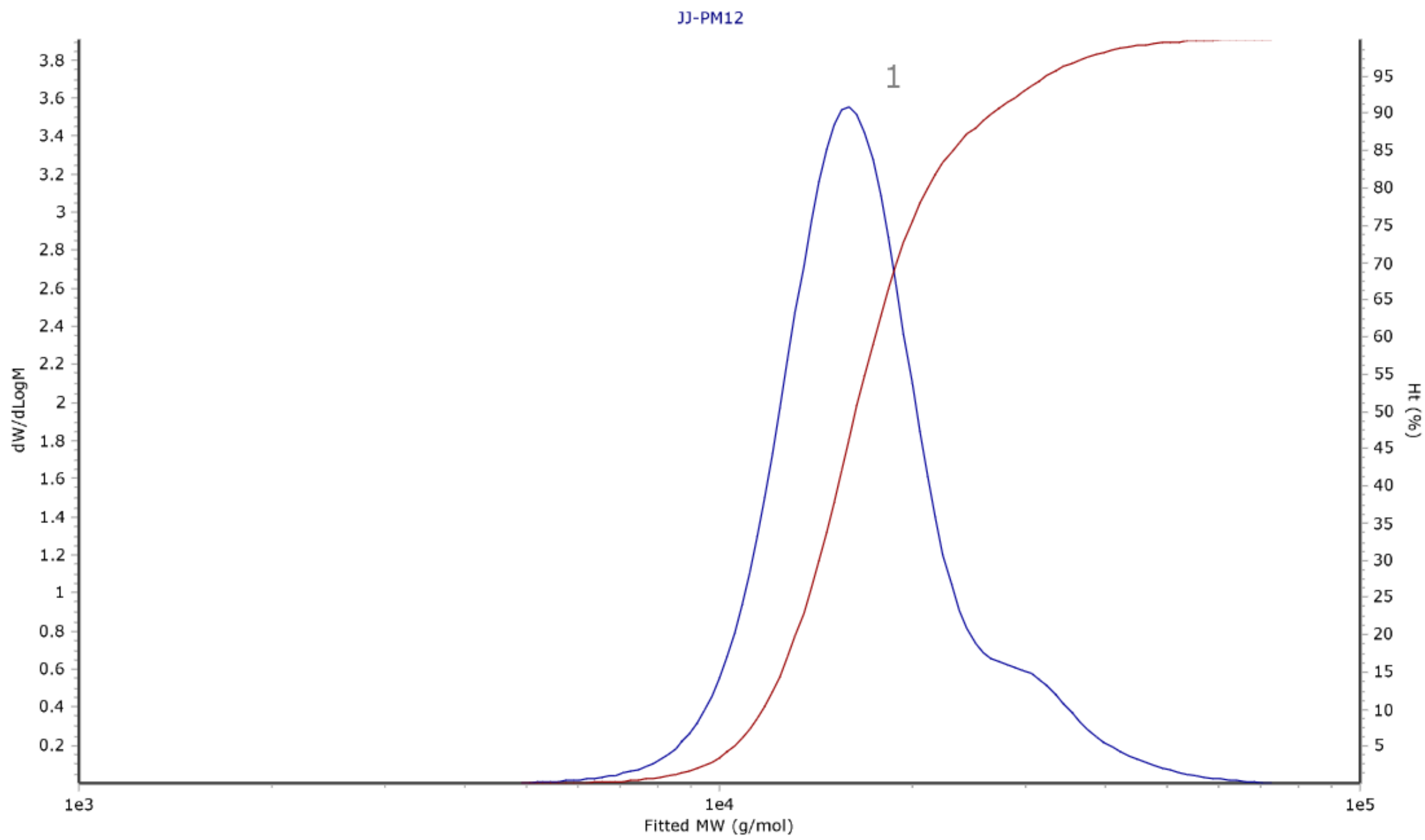

Figure S116. SEC-derived apparent molar-mass distribution of **P12** in THF, showing the differential distribution (blue) and cumulative distribution (red).

Table S10. SEC peak-integration parameters for **P12** in THF.

Peak Information

	Start (mins)	End (mins)
Baseline region 1	8.45000	9.68333
Baseline region 2	28.15000	28.86667
Peak 1	13.43333	16.66667

Peak Trace Information

Peak	Trace	Peak Max RT (mins)	Peak Area (mV.s)	Peak Height (mV)
Peak 1	RI	15.18333	4238.152	90.979
Peak 1	VS DP	15.16667	1090.312	19.704
Peak 1	VS IP	15.11667	22.660	0.634
Peak 1	UV 280nm	15.18333	1872.447	38.154

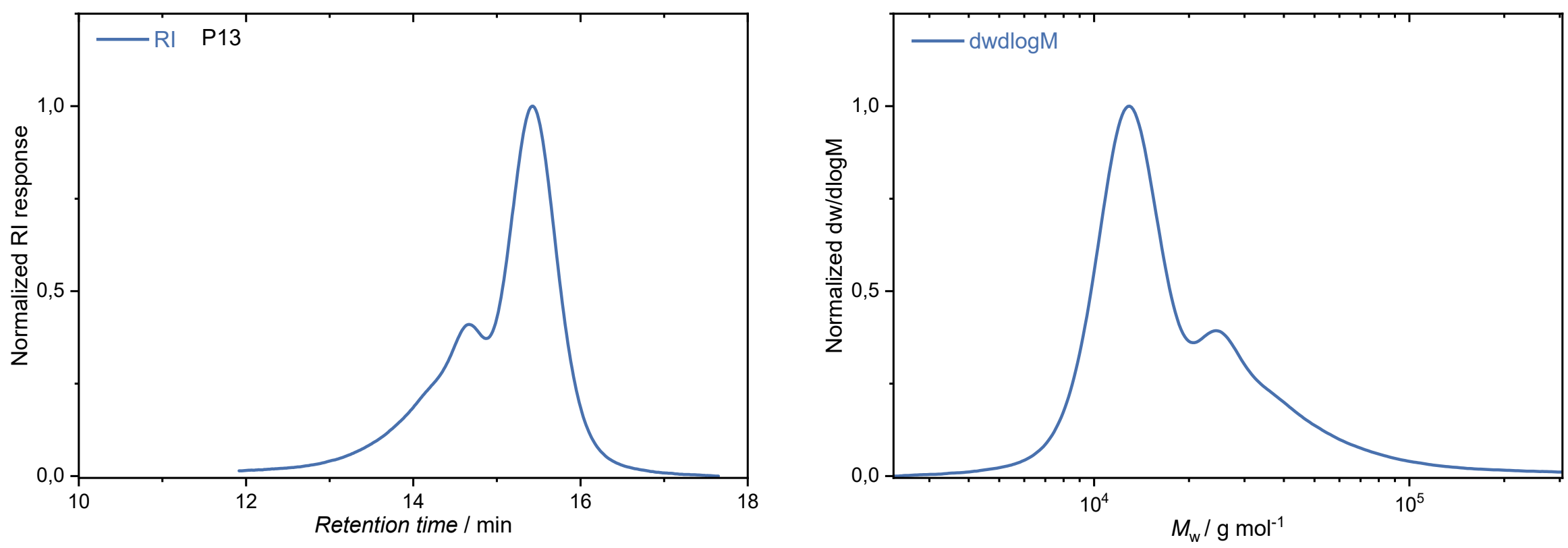

Figure S117. SEC analysis of **P13** recorded in THF, showing the normalized RI trace (left) and the corresponding molar mass distribution (right).

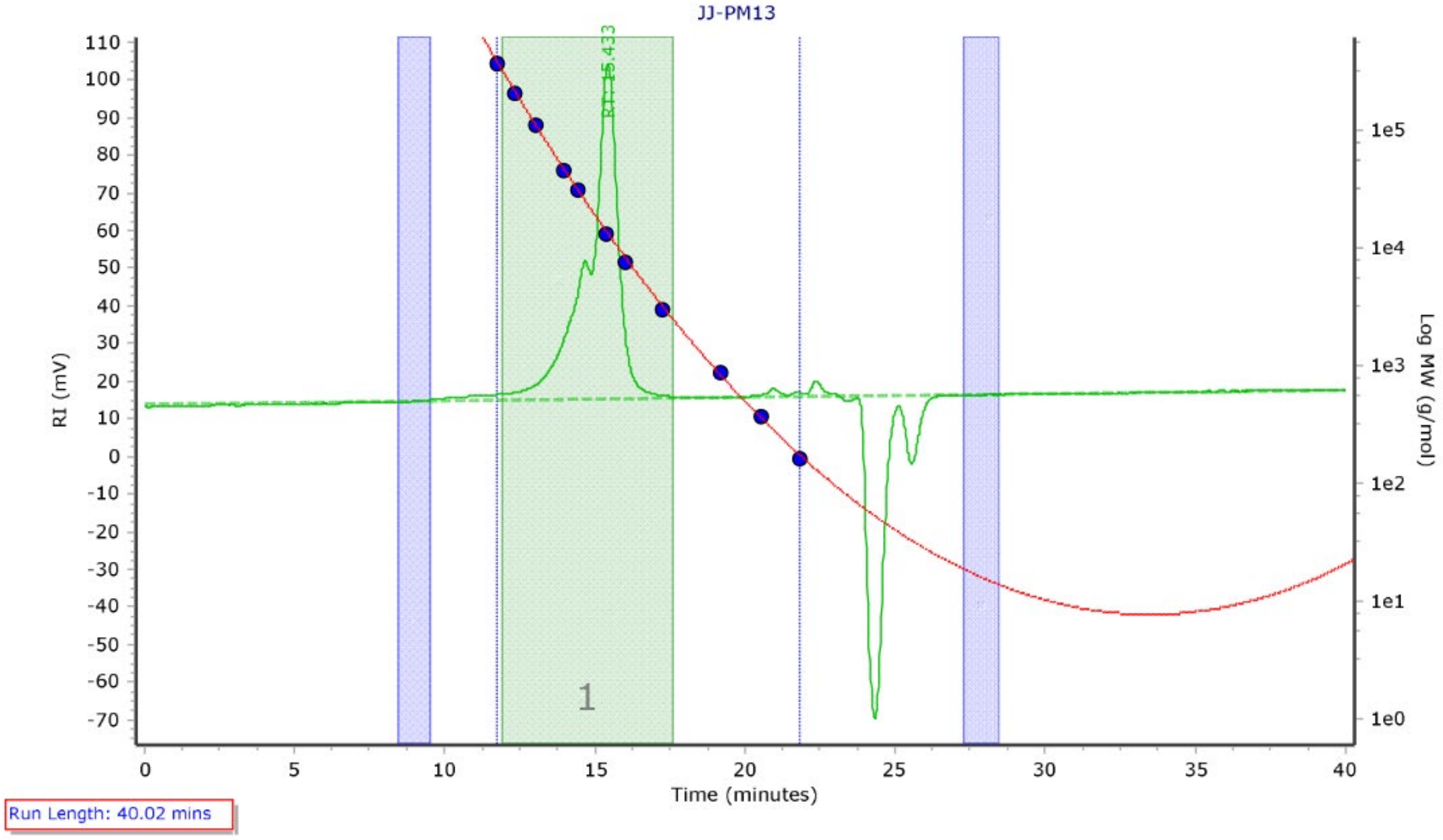

Figure S118. SEC chromatogram of **P13** in THF showing the RI trace (green), the integration region (green shaded area), and the corresponding polystyrene calibration curve (blue points; red fit).

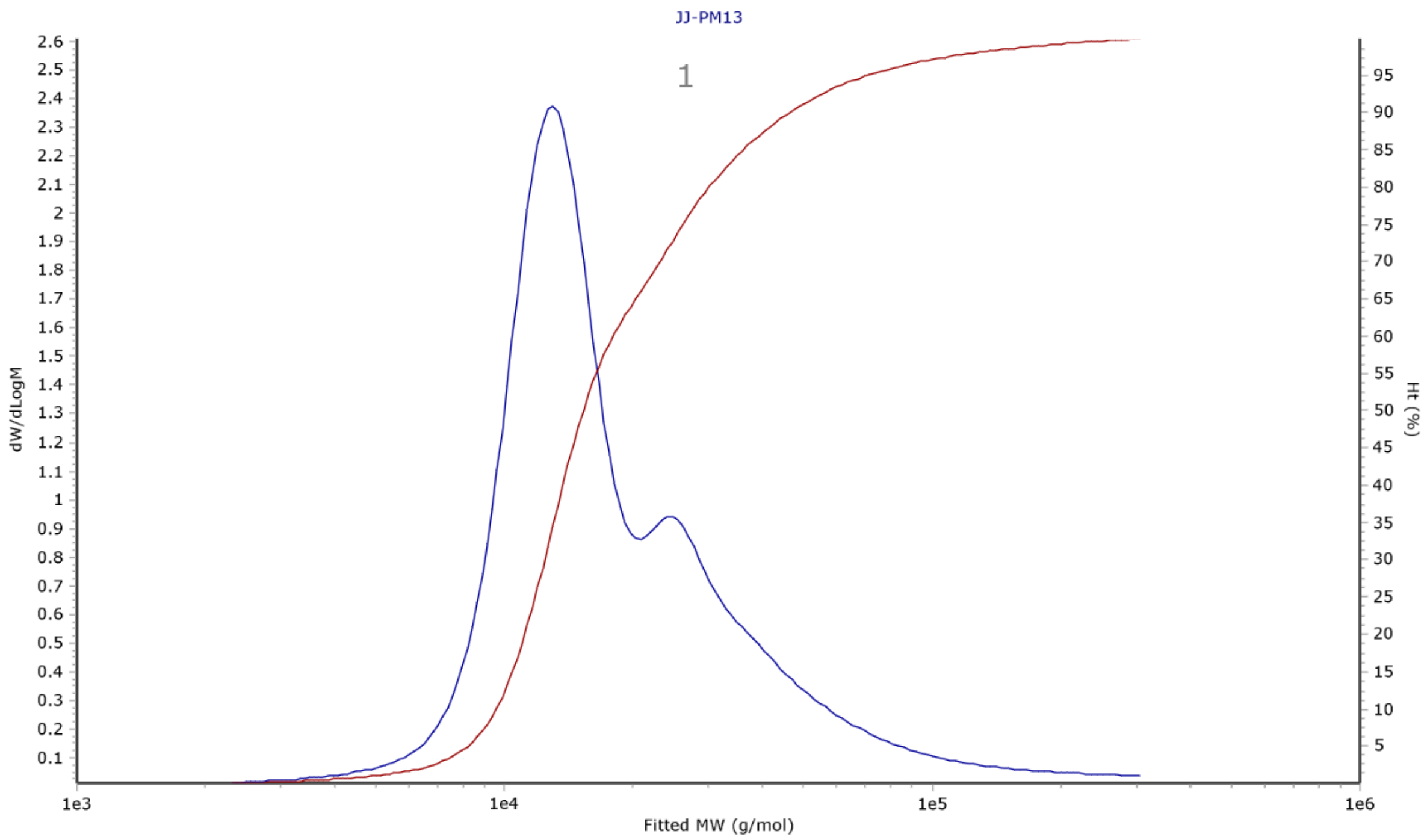

Figure S119. SEC-derived apparent molar-mass distribution of **P13** in THF, showing the differential distribution (blue) and cumulative distribution (red).

Table S11. SEC peak-integration parameters for **P13** in THF.

Peak Information

	Start (mins)	End (mins)
Baseline region 1	8.45000	9.55000
Baseline region 2	27.28333	28.48333
Peak 1	11.91667	17.66667

Peak Trace Information

Peak	Trace	Peak Max RT (mins)	Peak Area (mV.s)	Peak Height (mV)
Peak 1	RI	15.43333	6298.326	89.104
Peak 1	VS DP	15.41667	2089.609	21.415
Peak 1	VS IP	15.30000	62.443	0.818
Peak 1	UV 280nm	15.43333	4404.021	55.333

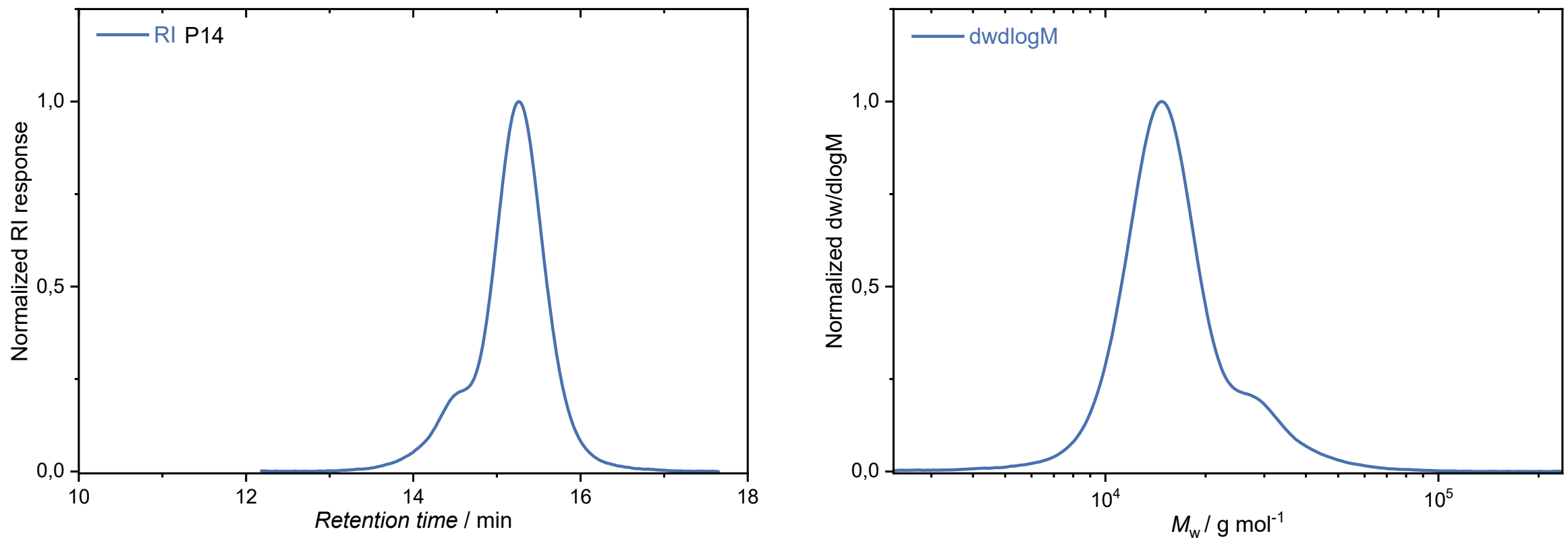

Figure S120. SEC analysis of **P14** recorded in THF, showing the normalized RI trace (left) and the corresponding molar mass distribution (right).

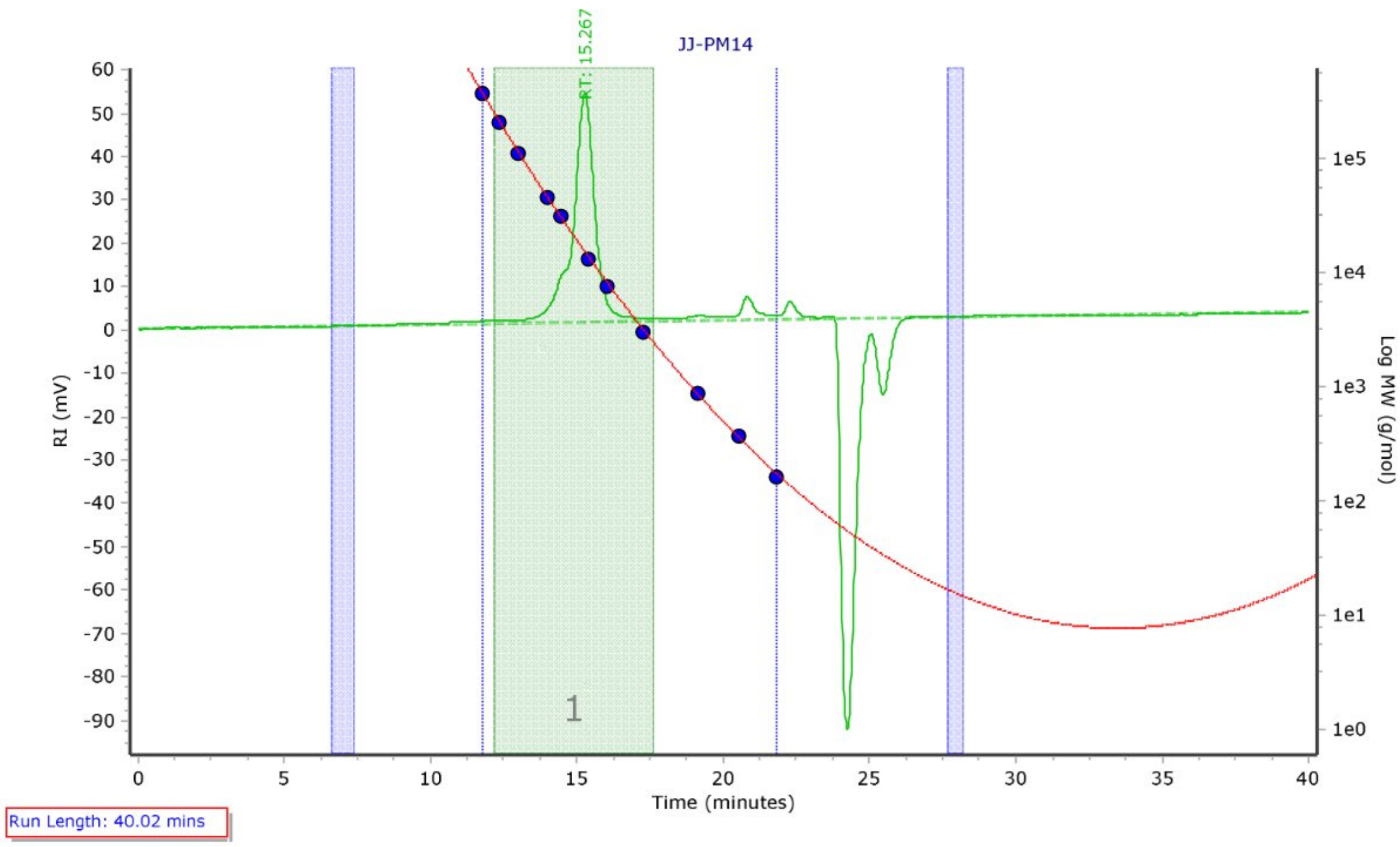

Figure S121. SEC chromatogram of **P14** in THF showing the RI trace (green), the integration region (green shaded area), and the corresponding polystyrene calibration curve (blue points; red fit).

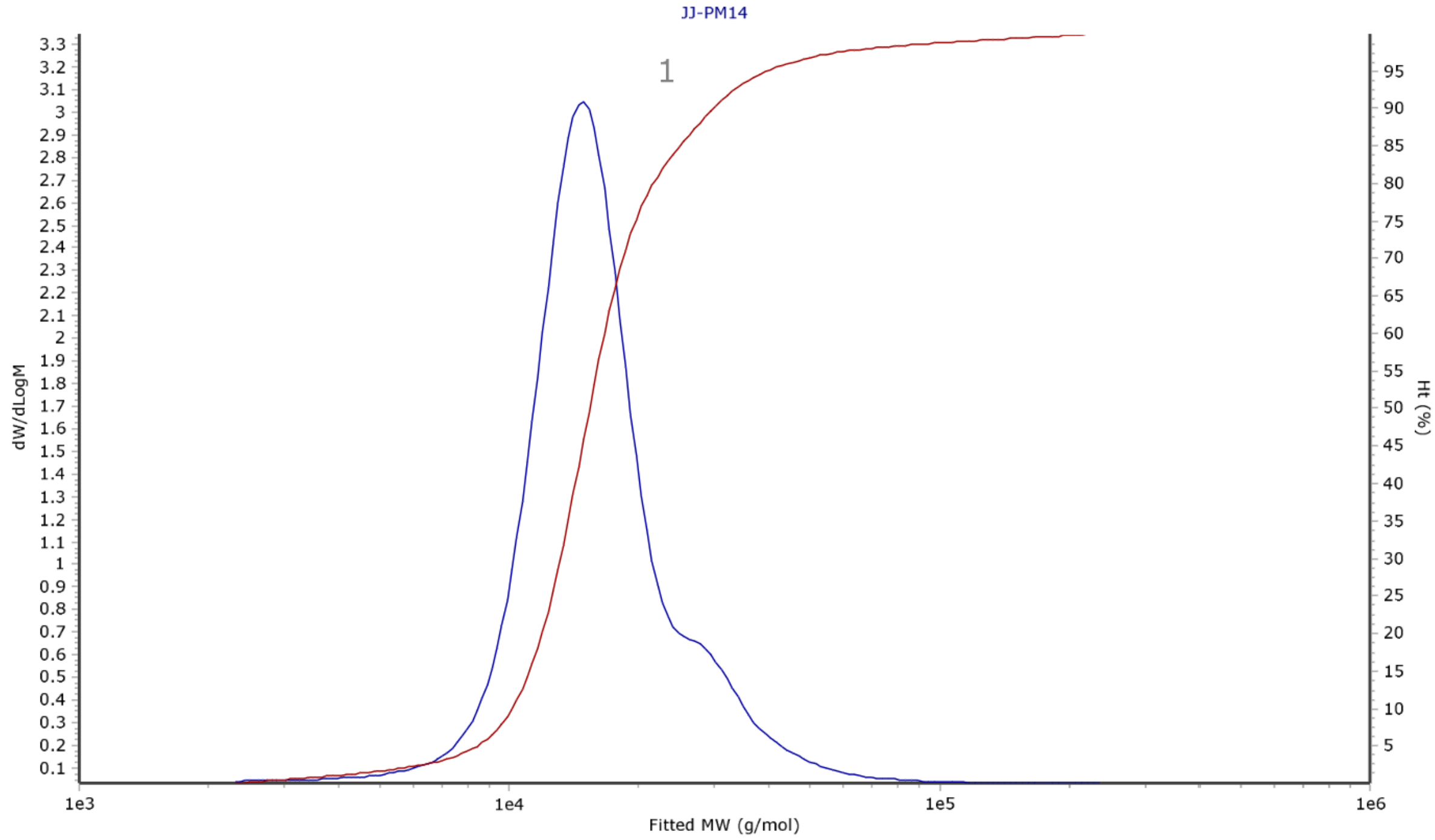

Figure S122. SEC-derived apparent molar-mass distribution of **P14** in THF, showing the differential distribution (blue) and cumulative distribution (red).

Table S12. SEC peak-integration parameters for **P14** in THF.

Peak Information

	Start (mins)	End (mins)
Baseline region 1	6.61667	7.40000
Baseline region 2	27.63333	28.26667
Peak 1	12.18333	17.66667

Peak Trace Information

Peak	Trace	Peak Max RT (mins)	Peak Area (mV.s)	Peak Height (mV)
Peak 1	RI	15.26667	2878.815	52.729
Peak 1	VS DP	15.28333	826.700	11.740
Peak 1	VS IP	15.15000	18.058	0.449
Peak 1	UV 280nm	15.26667	1895.067	34.064

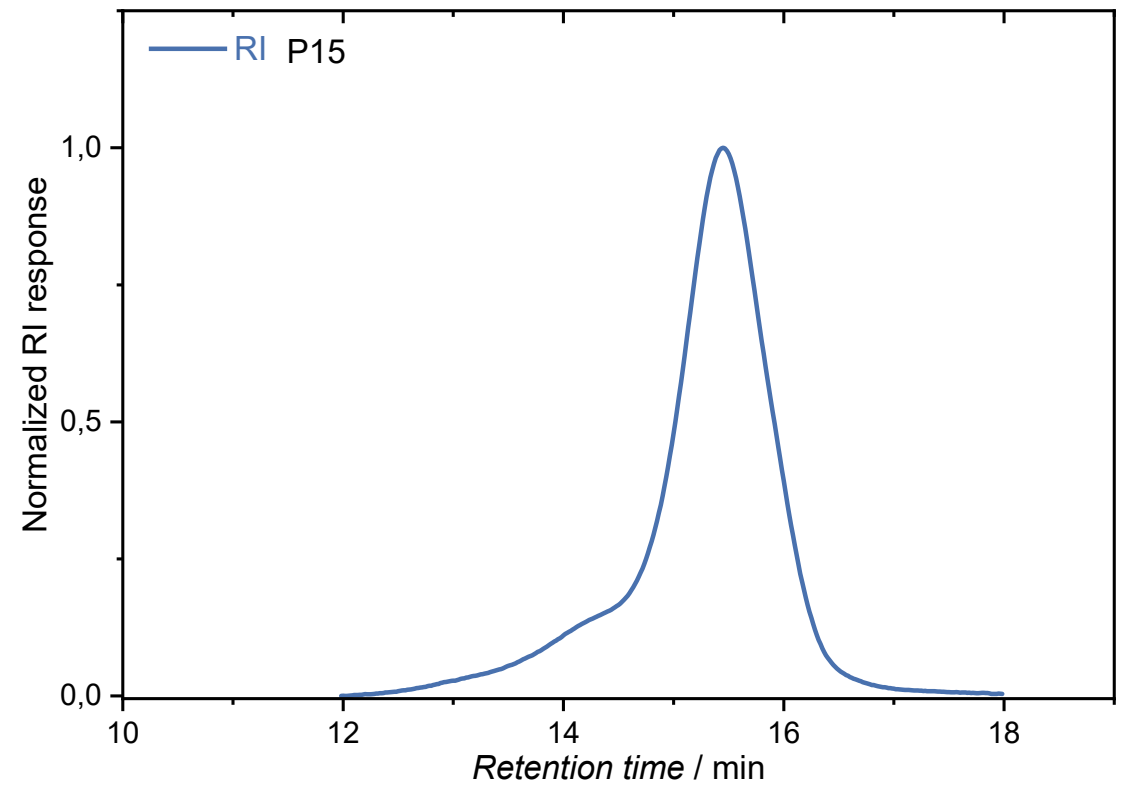

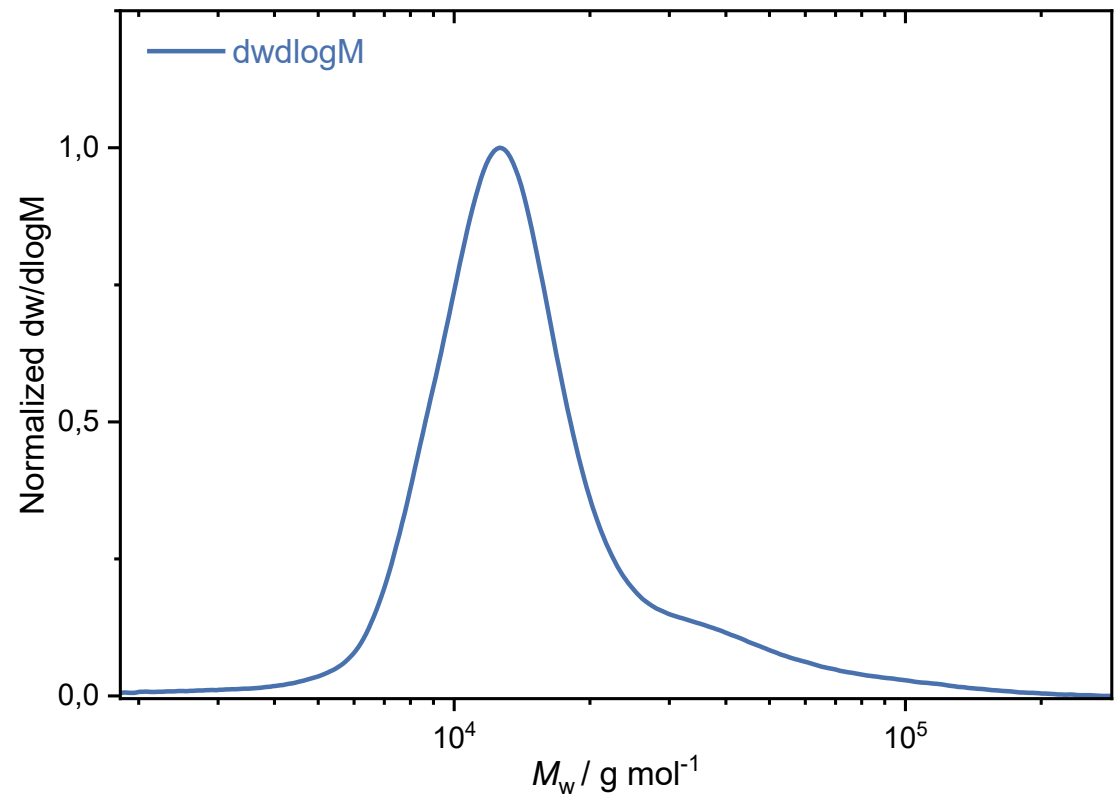

Figure S123. SEC analysis of **P15** recorded in THF, showing the normalized RI trace (left) and the corresponding molar mass distribution (right).

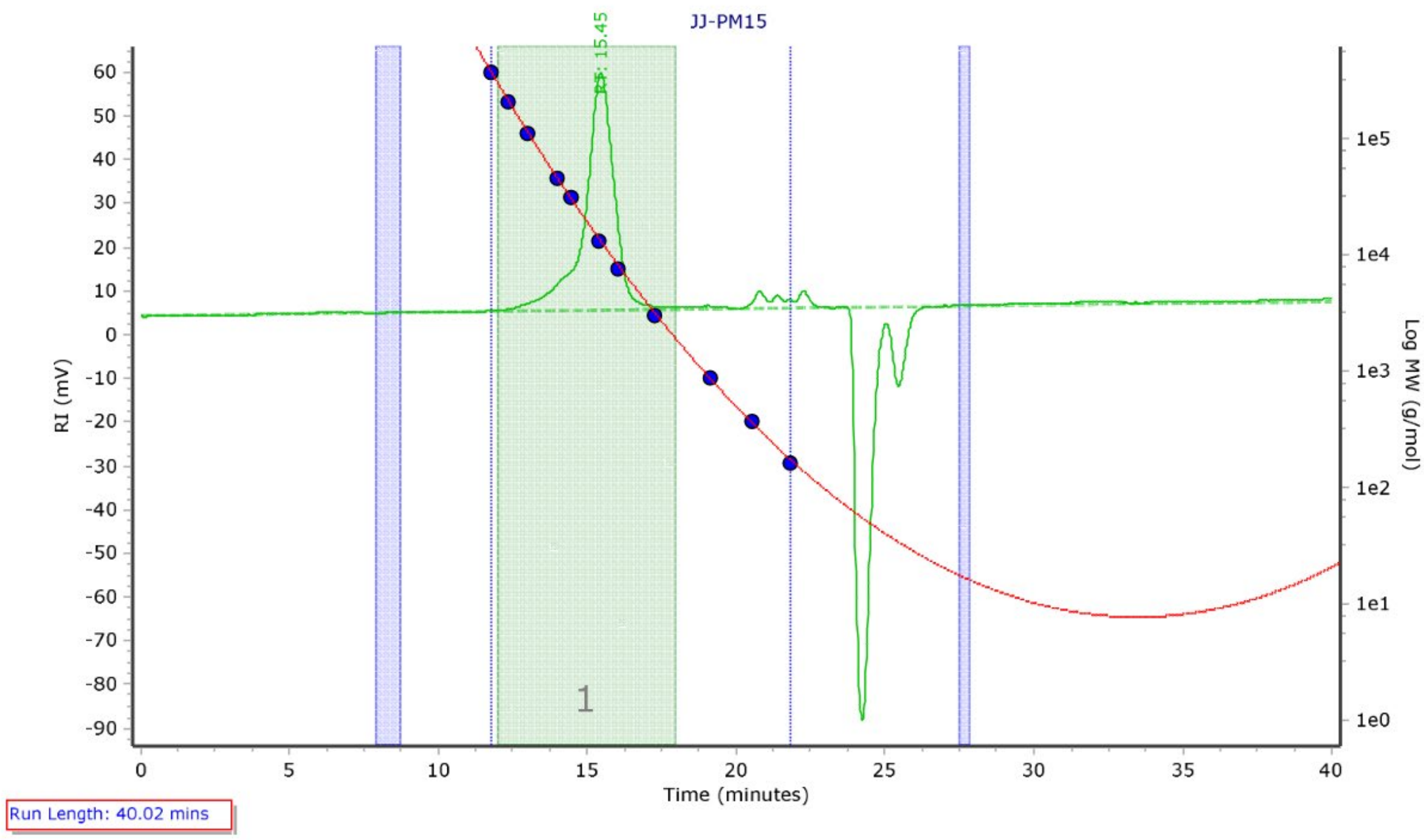

Figure S124. SEC chromatogram of **P15** in THF showing the RI trace (green), the integration region (green shaded area), and the corresponding polystyrene calibration curve (blue points; red fit).

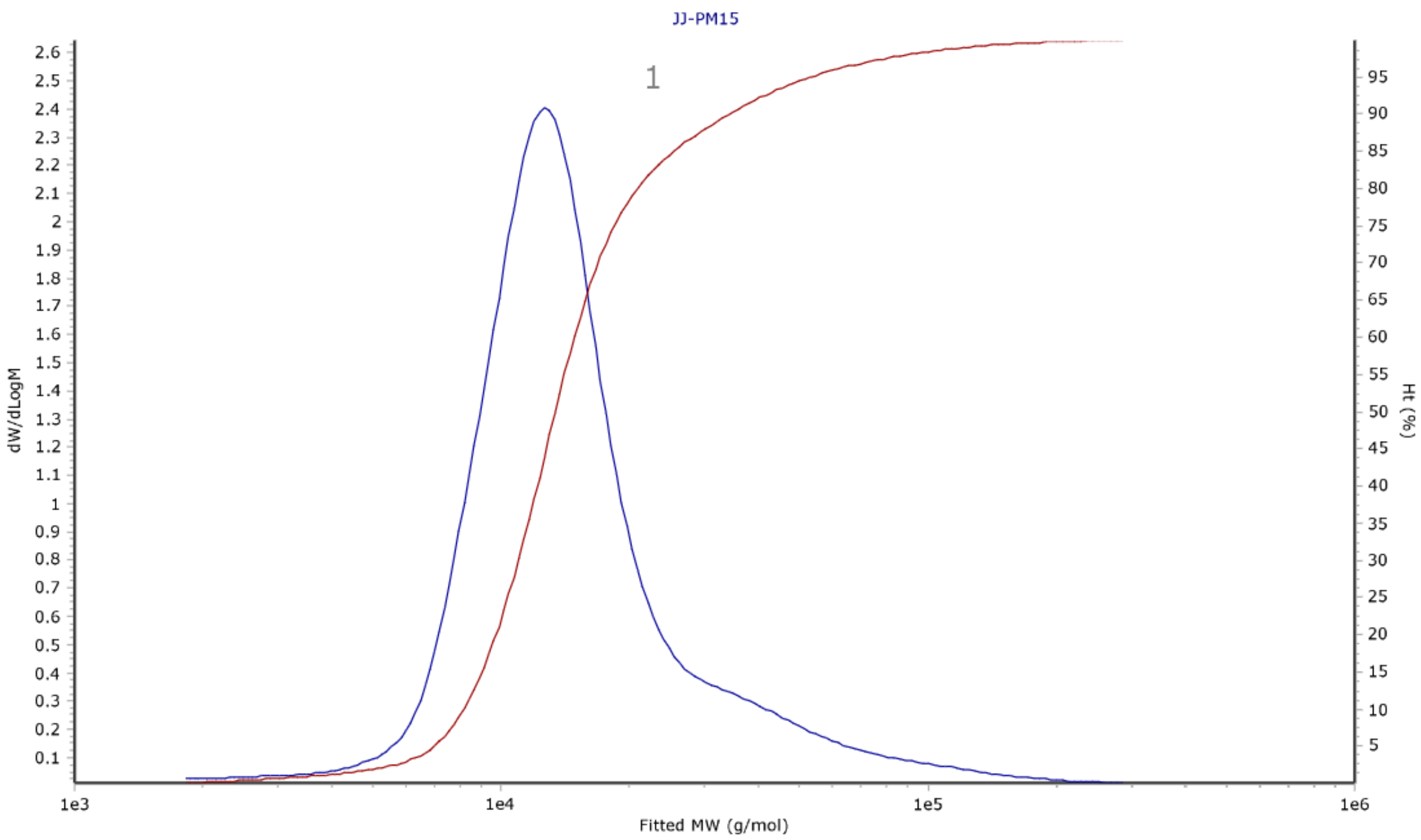

Figure S125. SEC-derived apparent molar-mass distribution of **P15** in THF, showing the differential distribution (blue) and cumulative distribution (red).

Table S13. SEC peak-integration parameters for **P15** in THF.

Peak Information

	Start (mins)	End (mins)
Baseline region 1	7.91667	8.75000
Baseline region 2	27.50000	27.91667
Peak 1	11.98333	18.00000

Peak Trace Information

Peak	Trace	Peak Max RT (mins)	Peak Area (mV.s)	Peak Height (mV)
Peak 1	RI	15.45000	3799.178	54.359
Peak 1	VS DP	15.45000	1177.691	12.533
Peak 1	VS IP	15.28333	25.582	0.406
Peak 1	UV 280nm	15.46667	77479.610	1028.103

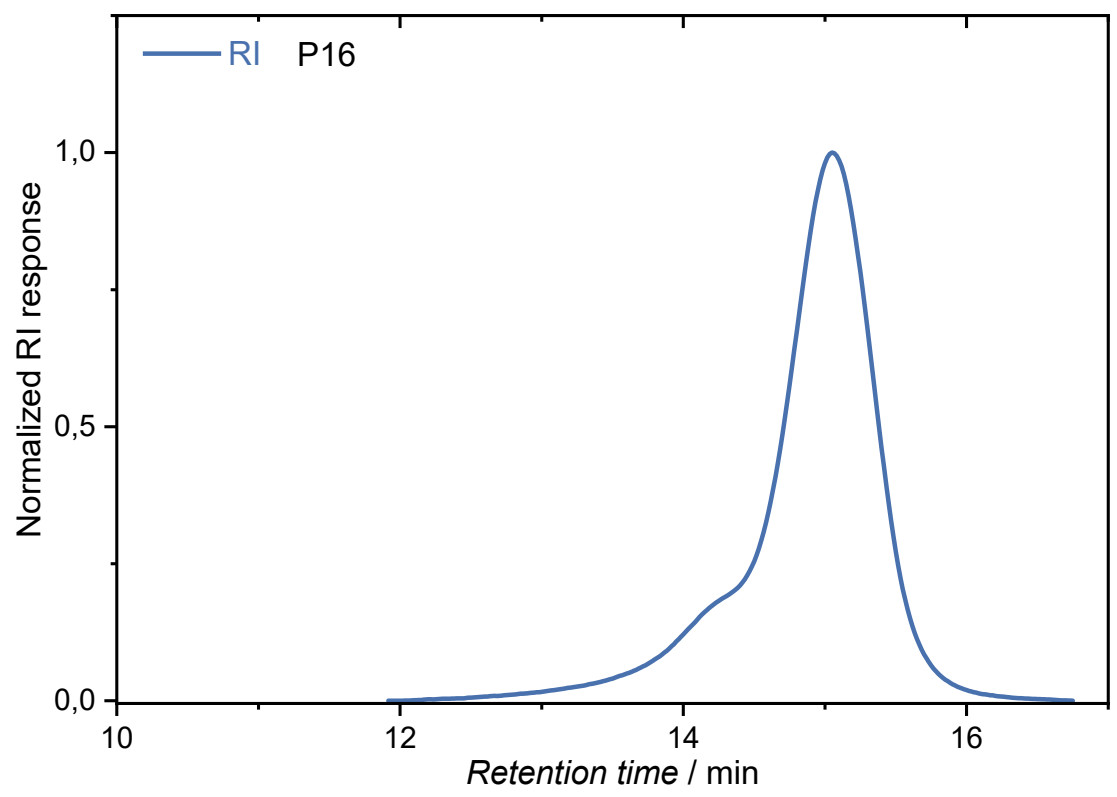

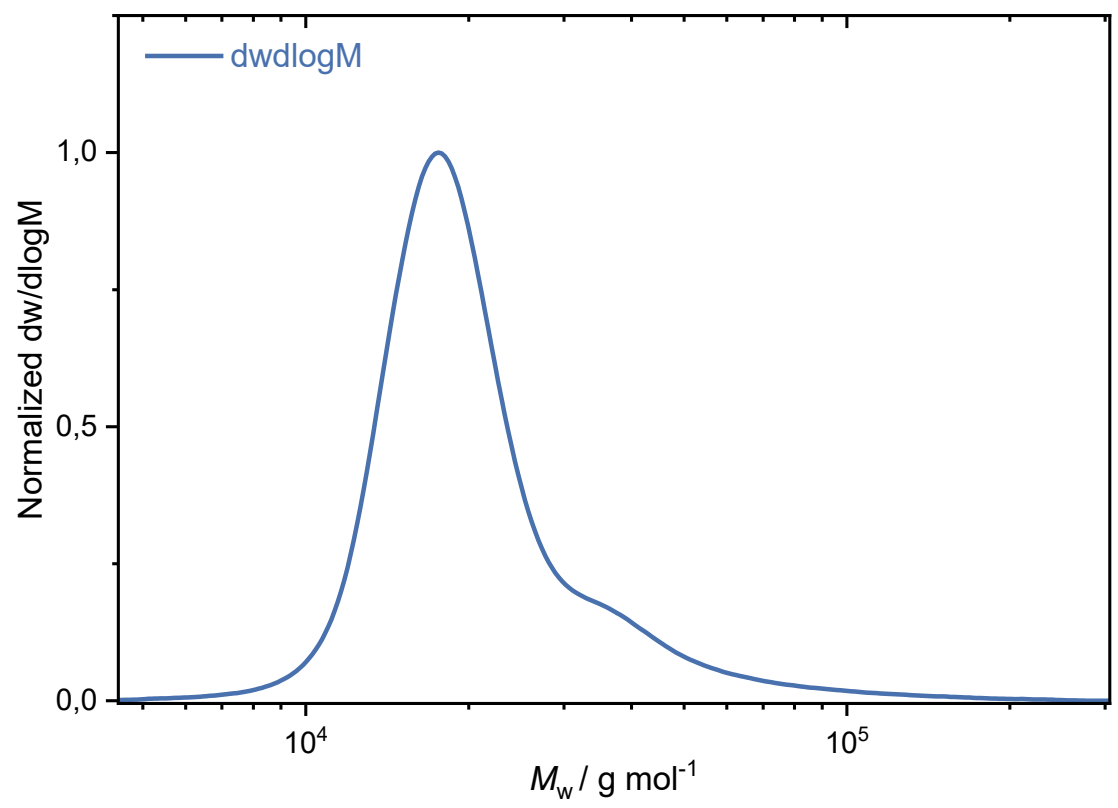

Figure S126. SEC analysis of **P16** recorded in THF, showing the normalized RI trace (left) and the corresponding molar mass distribution (right).

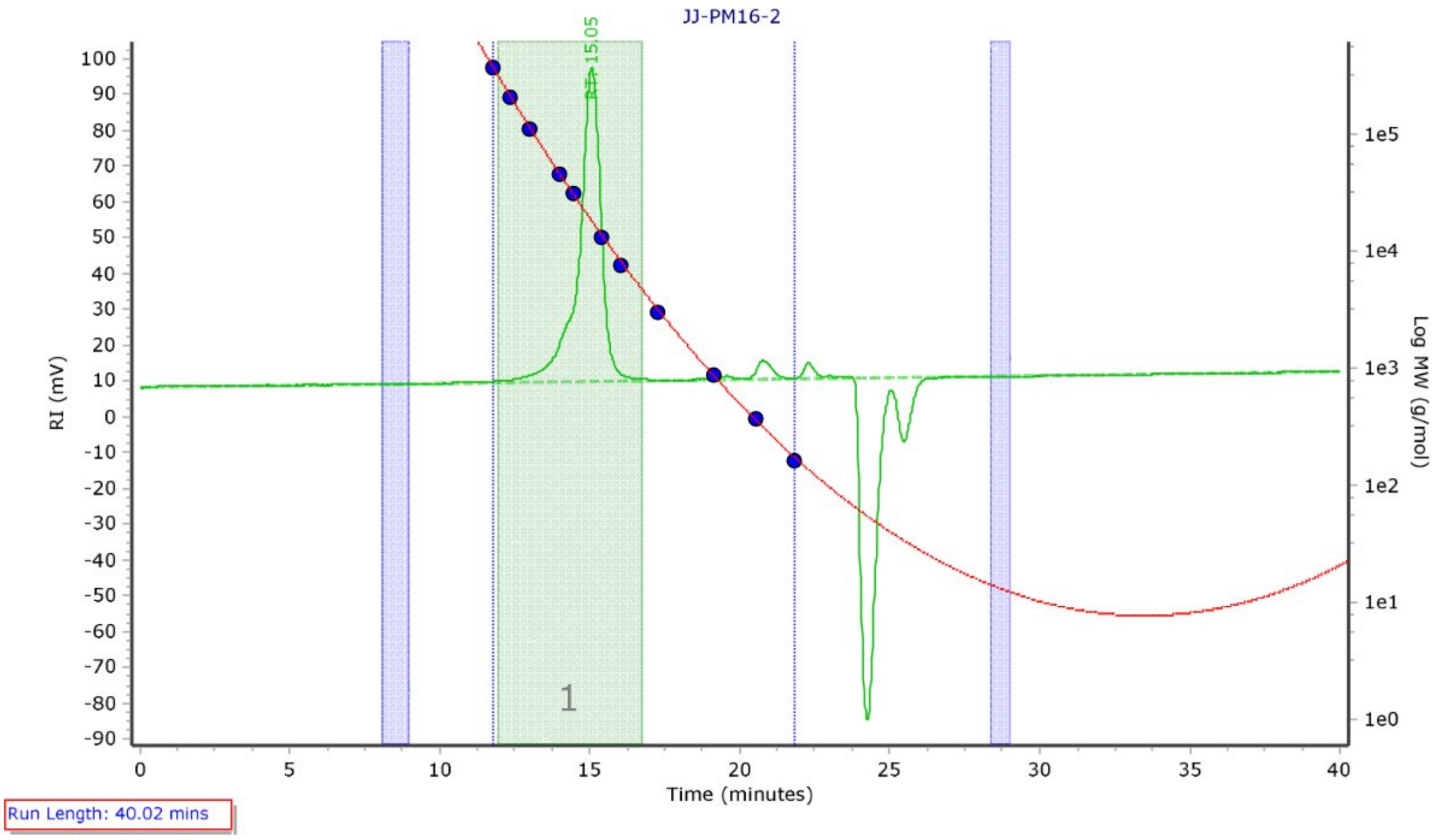

Figure S127. SEC chromatogram of **P16** in THF showing the RI trace (green), the integration region (green shaded area), and the corresponding polystyrene calibration curve (blue points; red fit).

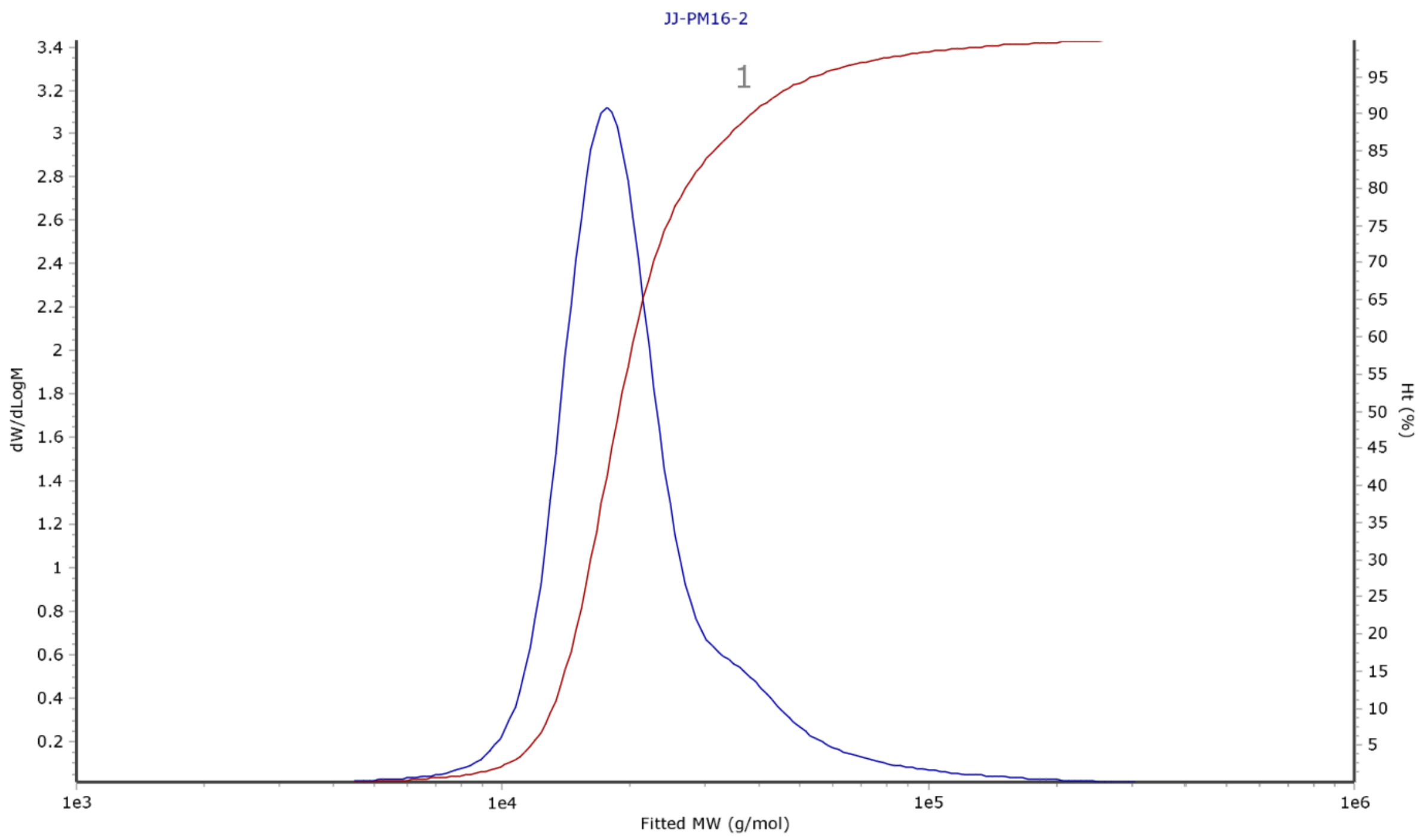

Figure S128. SEC-derived apparent molar-mass distribution of **P16** in THF, showing the differential distribution (blue) and cumulative distribution (red).

Table S14. SEC peak-integration parameters for **P16** in THF.

Peak Information

	Start (mins)	End (mins)
Baseline region 1	8.05000	8.98333
Baseline region 2	28.36667	29.05000
Peak 1	11.91667	16.76667

Peak Trace Information

Peak	Trace	Peak Max RT (mins)	Peak Area (mV.s)	Peak Height (mV)
Peak 1	RI	15.05000	4615.600	87.609
Peak 1	VS DP	15.06667	2023.193	26.184
Peak 1	VS IP	14.95000	33.899	0.755
Peak 1	UV 280nm	15.06667	6977.708	119.909

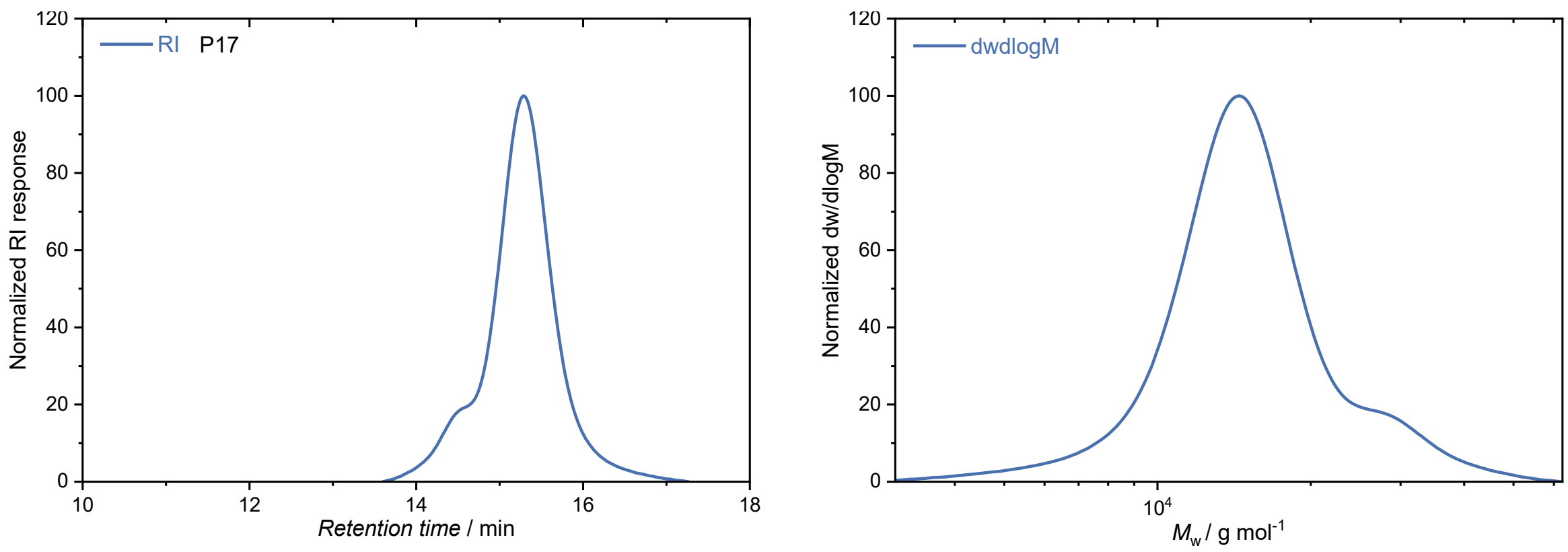

Figure S129. SEC analysis of **P17** recorded in THF, showing the normalized RI trace (left) and the corresponding molar mass distribution (right).

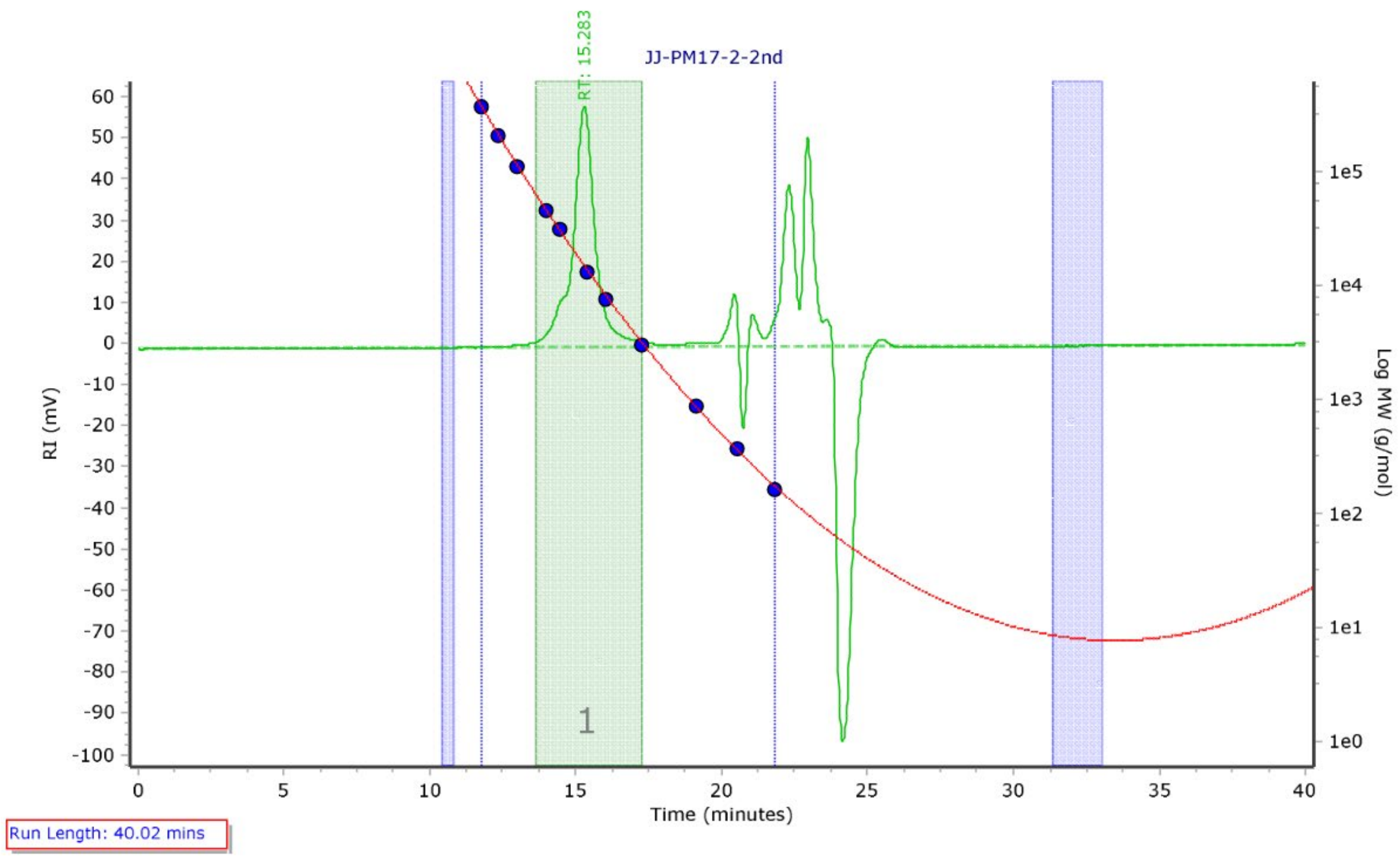

Figure S130. SEC chromatogram of **P17** in THF showing the RI trace (green), the integration region (green shaded area), and the corresponding polystyrene calibration curve (blue points; red fit).

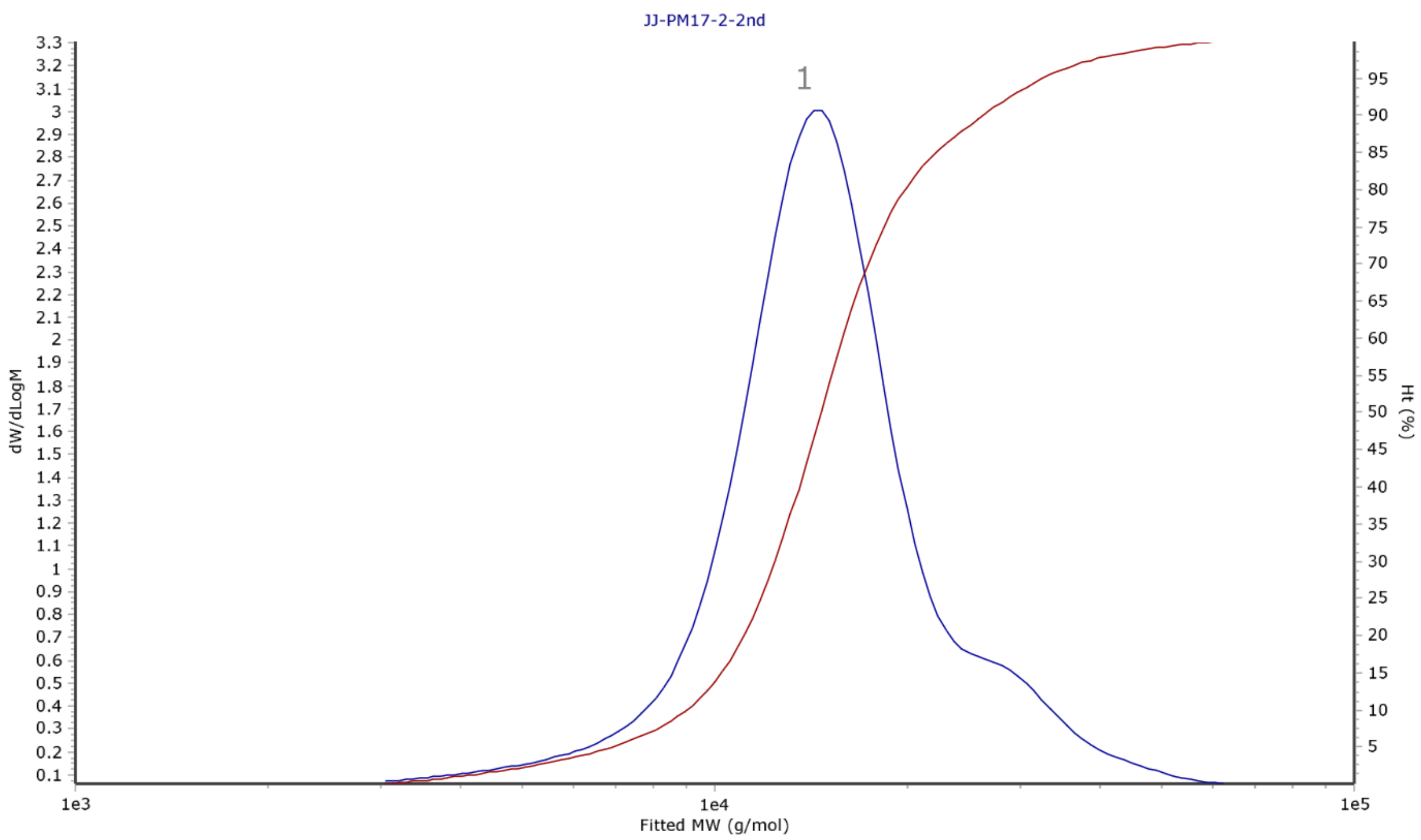

Figure S131. SEC-derived apparent molar-mass distribution of **P17** in THF, showing the differential distribution (blue) and cumulative distribution (red).

Table S15. SEC peak-integration parameters for **P17** in THF.

Peak Information

	Start (mins)	End (mins)
Baseline region 1	10.40000	10.88333
Baseline region 2	31.36667	33.11667
Peak 1	13.60000	17.28333

Peak Trace Information

Peak	Trace	Peak Max RT (mins)	Peak Area (mV.s)	Peak Height (mV)
Peak 1	RI	15.28333	3236.725	58.552
Peak 1	VS DP	15.30000	1033.373	15.936
Peak 1	VS IP	15.23333	37.235	0.633
Peak 1	UV 280nm	15.31667	2358.581	36.981

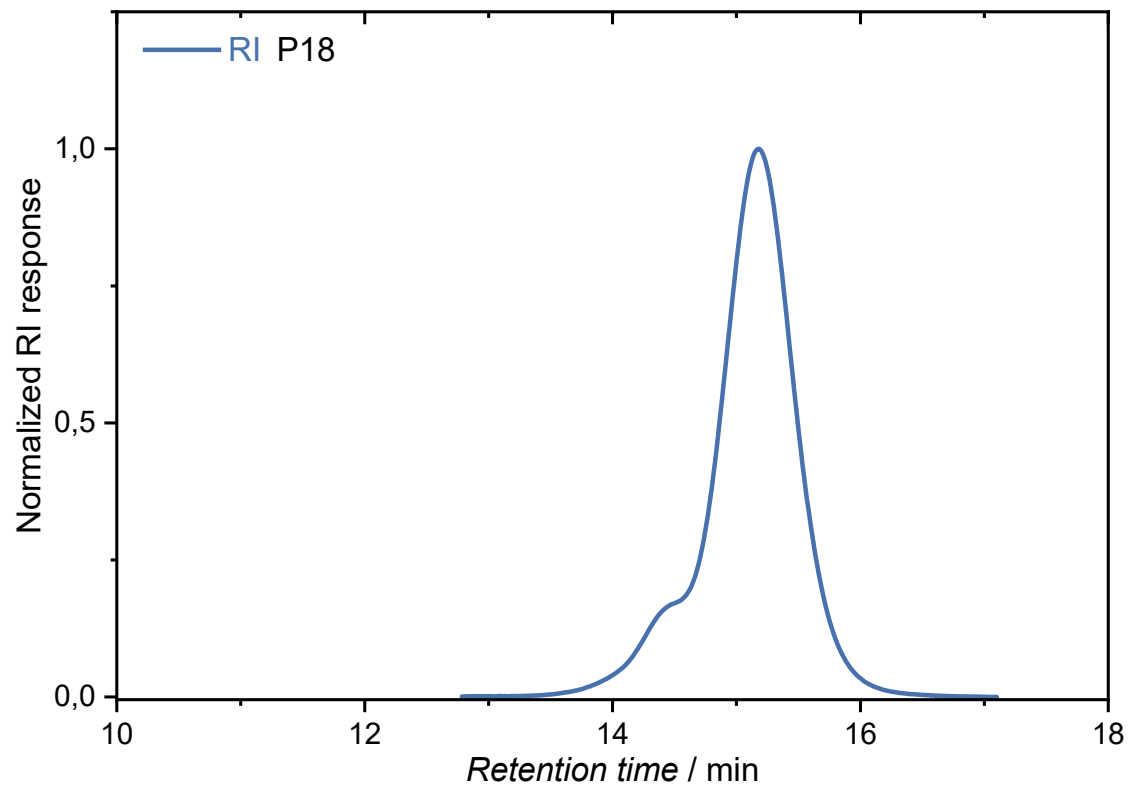

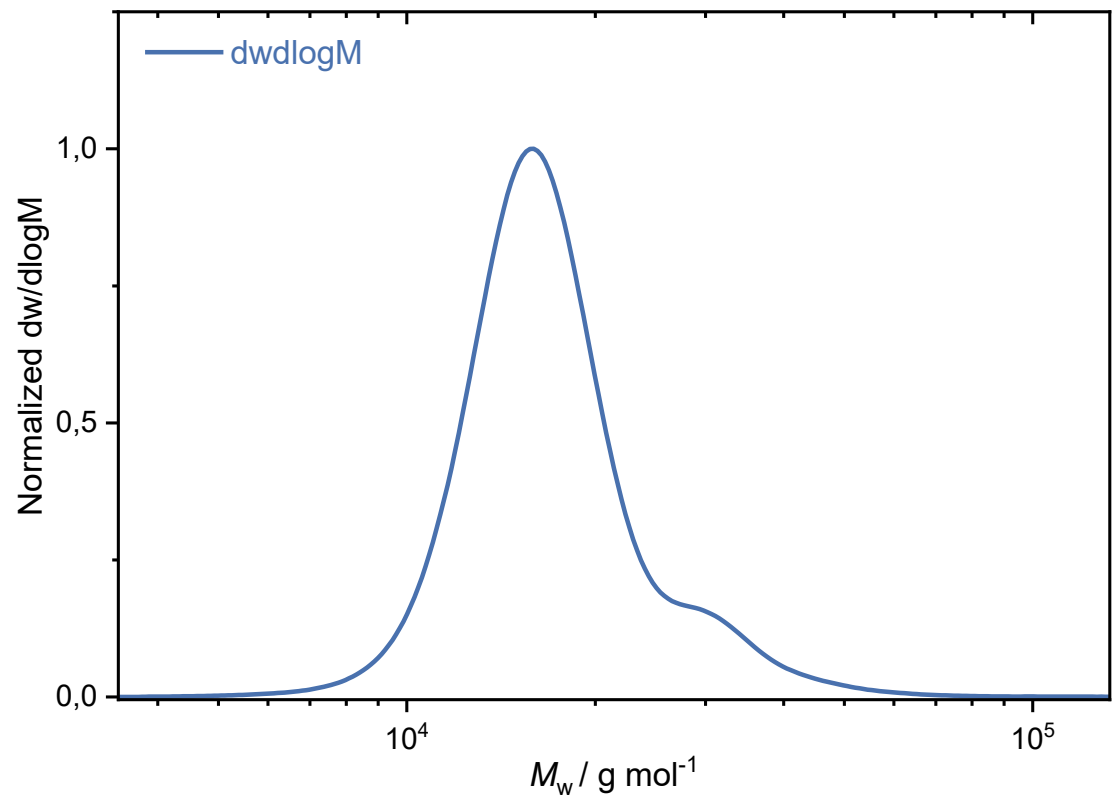

Figure S132. SEC analysis of **P18** recorded in THF, showing the normalized RI trace (left) and the corresponding molar mass distribution (right).

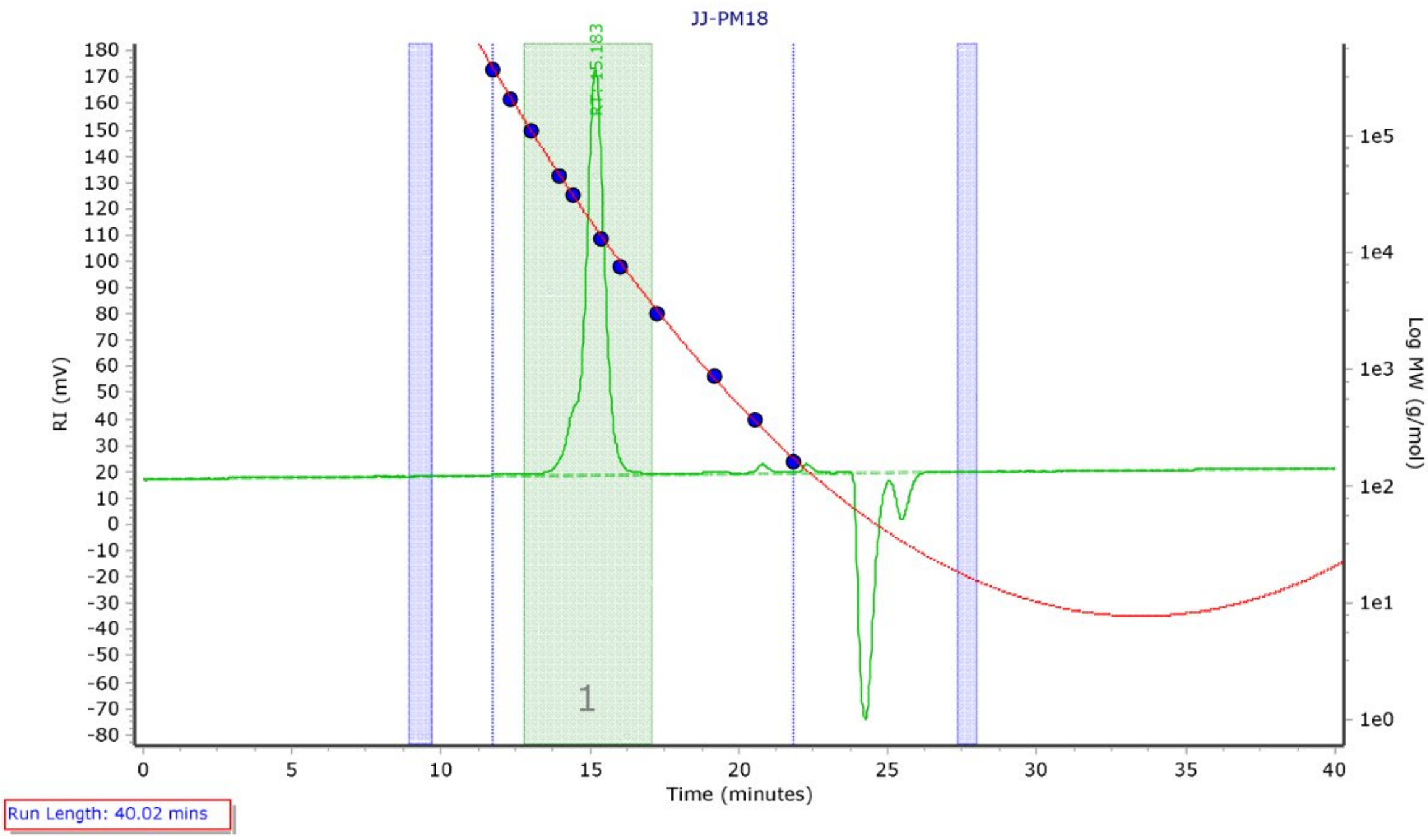

Figure S133. SEC chromatogram of **P18** in THF showing the RI trace (green), the integration region (green shaded area), and the corresponding polystyrene calibration curve (blue points; red fit).

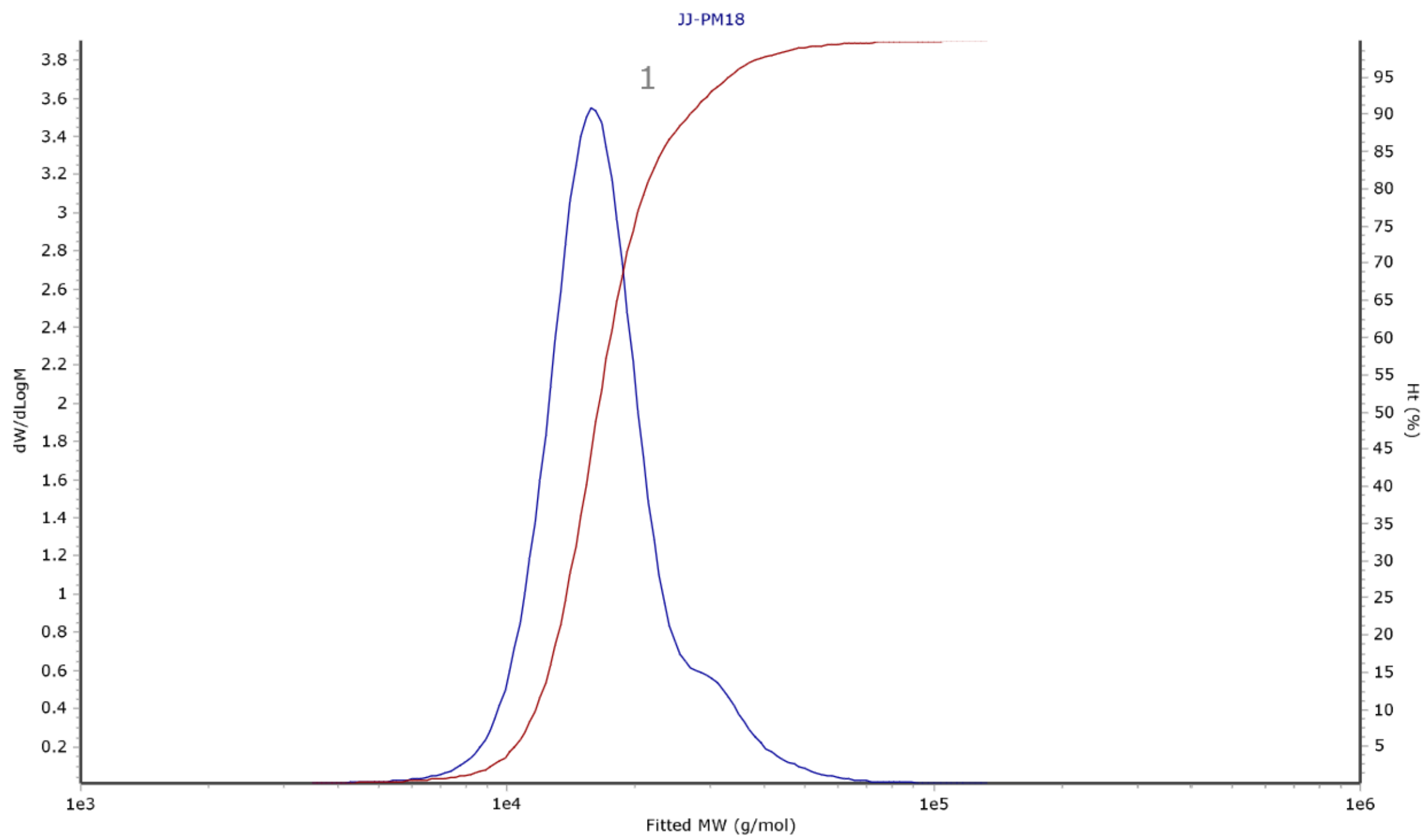

Figure S134. SEC-derived apparent molar-mass distribution of **P18** in THF, showing the differential distribution (blue) and cumulative distribution (red).

Table S16. SEC peak-integration parameters for **P18** in THF.

Peak Information

	Start (mins)	End (mins)
Baseline region 1	8.93333	9.71667
Baseline region 2	27.31667	28.05000
Peak 1	12.78333	17.11667

Peak Trace Information

Peak	Trace	Peak Max RT (mins)	Peak Area (mV.s)	Peak Height (mV)
Peak 1	RI	15.18333	7180.174	154.065
Peak 1	VS DP	15.18333	1843.350	34.935
Peak 1	VS IP	15.13333	52.494	1.158
Peak 1	UV 280nm	15.18333	3288.244	66.136

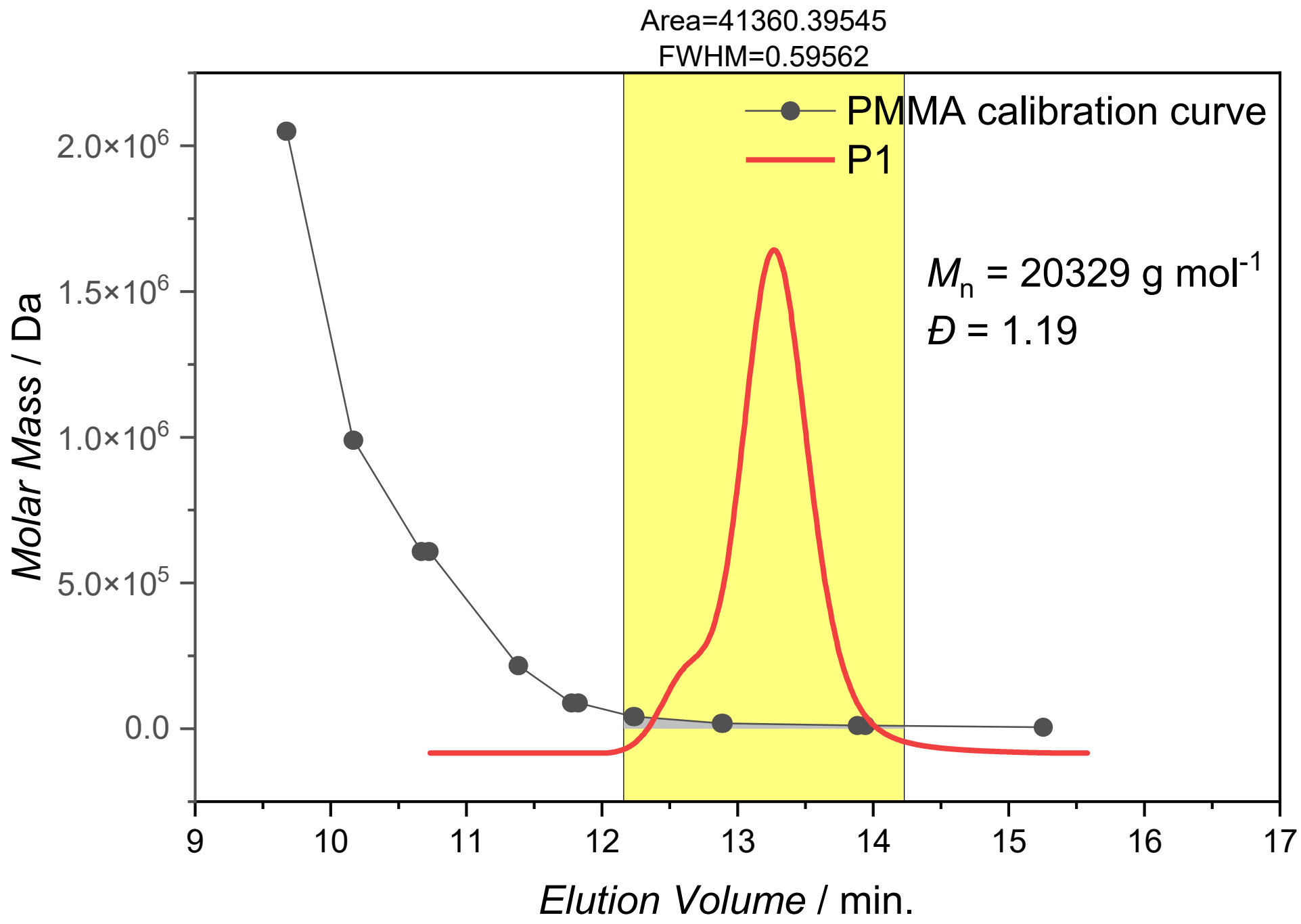

Figure S135. SEC analysis of **P1** recorded in DMAc, showing the PMMA calibration curve (black) and the corresponding RI trace (red) used for the calculation of the apparent number-average molar mass and dispersity. The highlighted region indicates the integration range used for the SEC analysis.

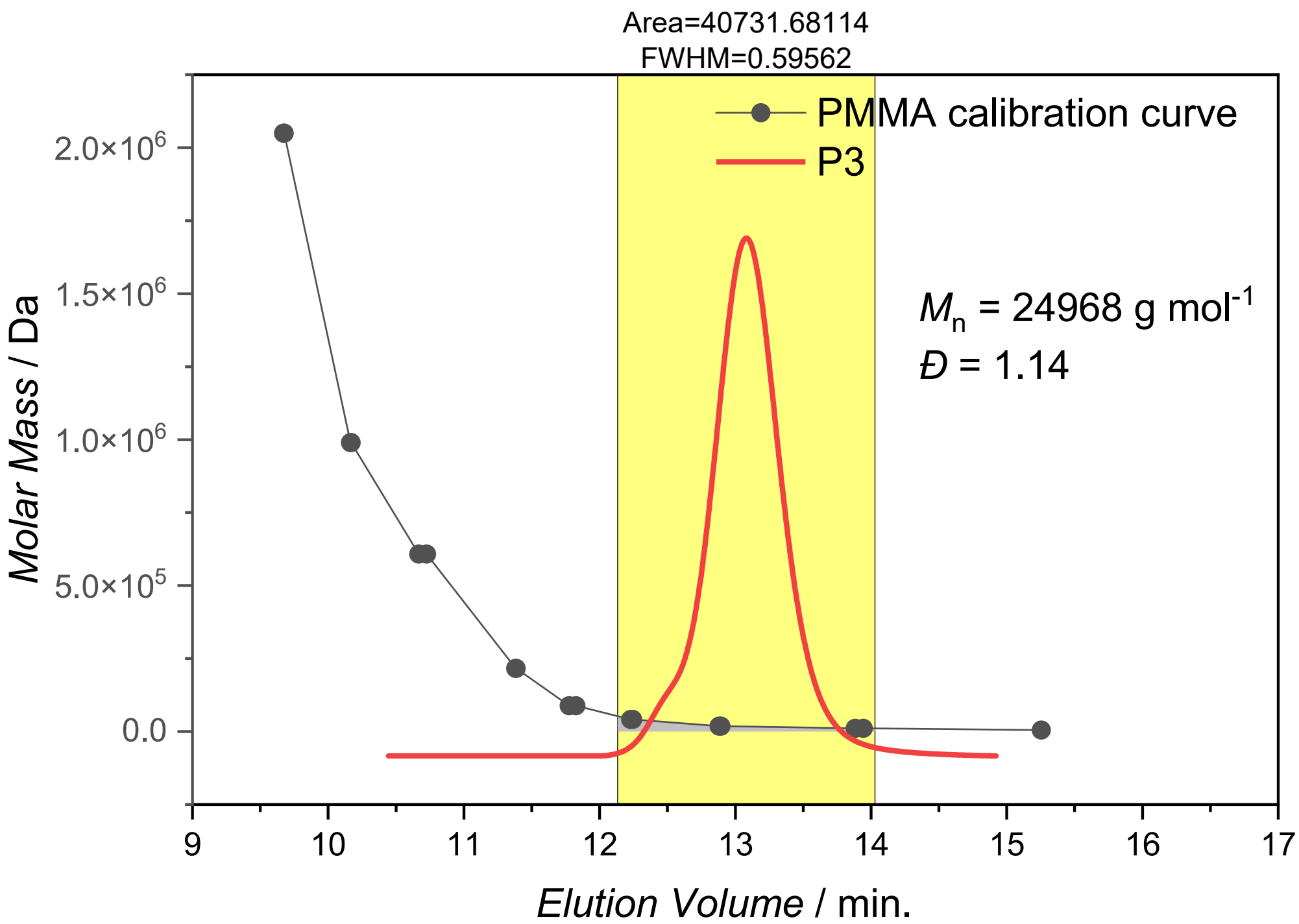

Figure S136. SEC analysis of **P3** recorded in DMAc, showing the PMMA calibration curve (black) and the corresponding RI trace (red) used for the calculation of the apparent number-average molar mass and dispersity. The highlighted region indicates the integration range used for the SEC analysis.

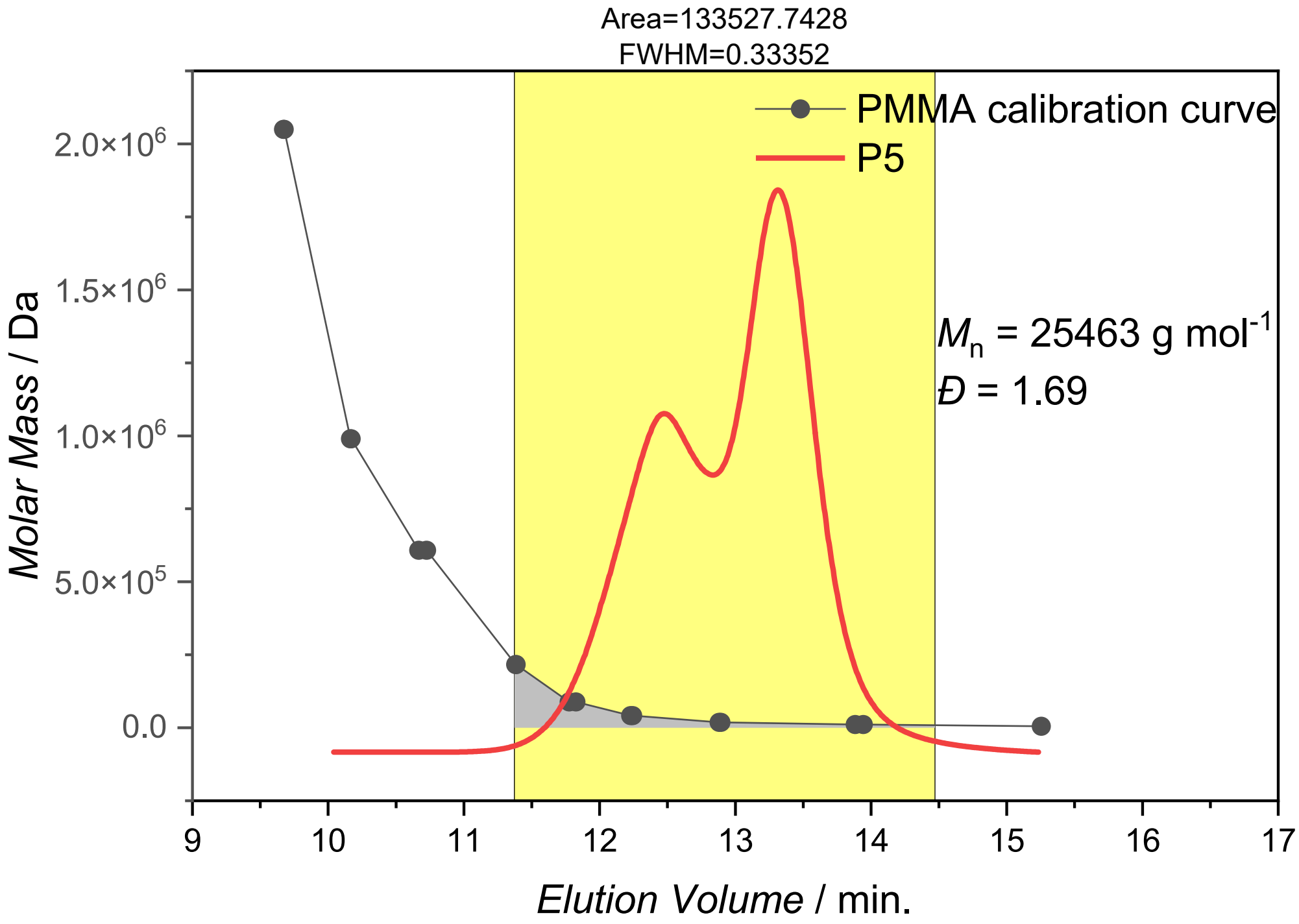

Figure S137. SEC analysis of **P5** recorded in DMAc, showing the PMMA calibration curve (black) and the corresponding RI trace (red) used for the calculation of the apparent number-average molar mass and dispersity. The highlighted region indicates the integration range used for the SEC analysis.

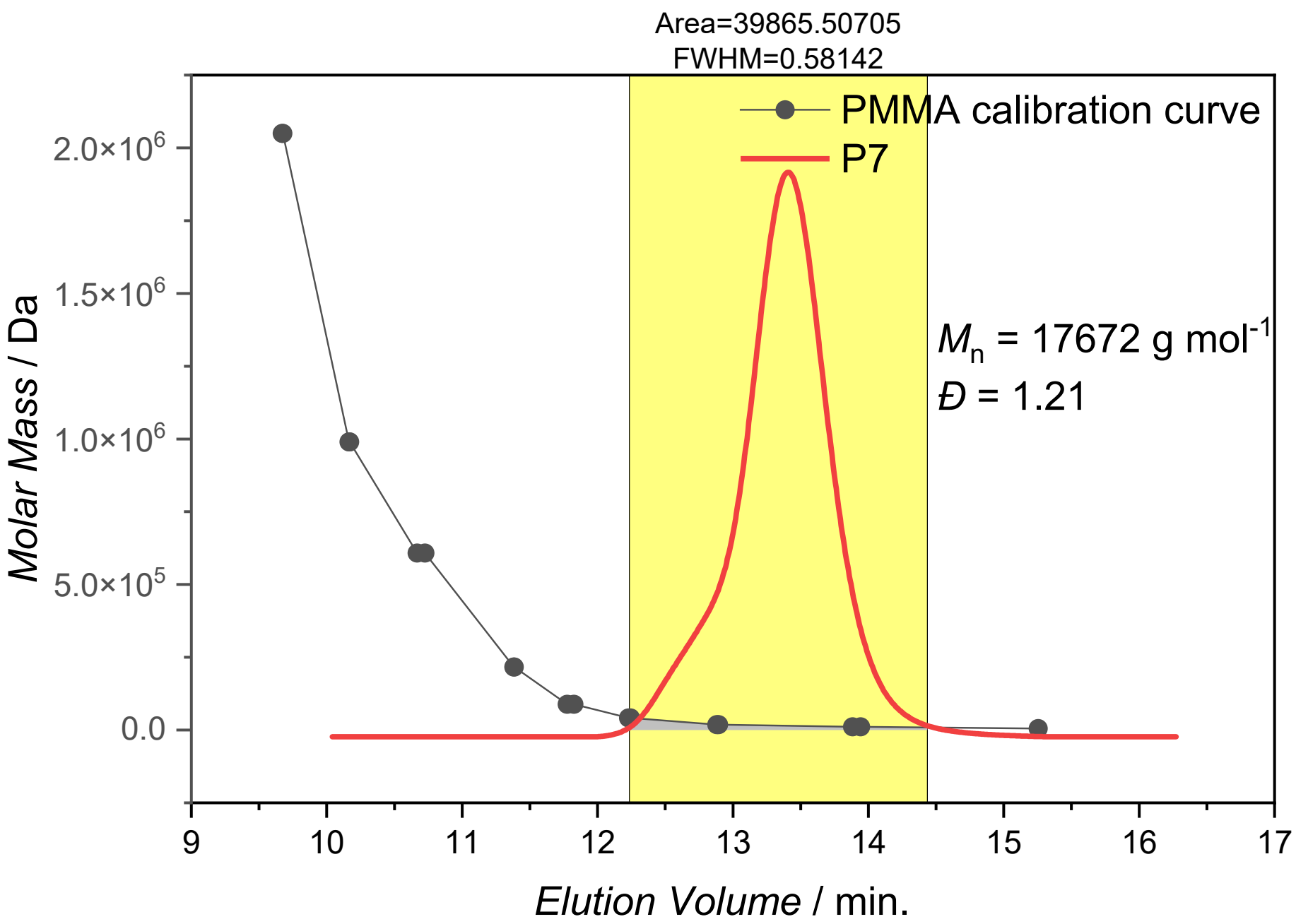

Figure S138. SEC analysis of **P7** recorded in DMAc, showing the PMMA calibration curve (black) and the corresponding RI trace (red) used for the calculation of the apparent number-average molar mass and dispersity. The highlighted region indicates the integration range used for the SEC analysis.

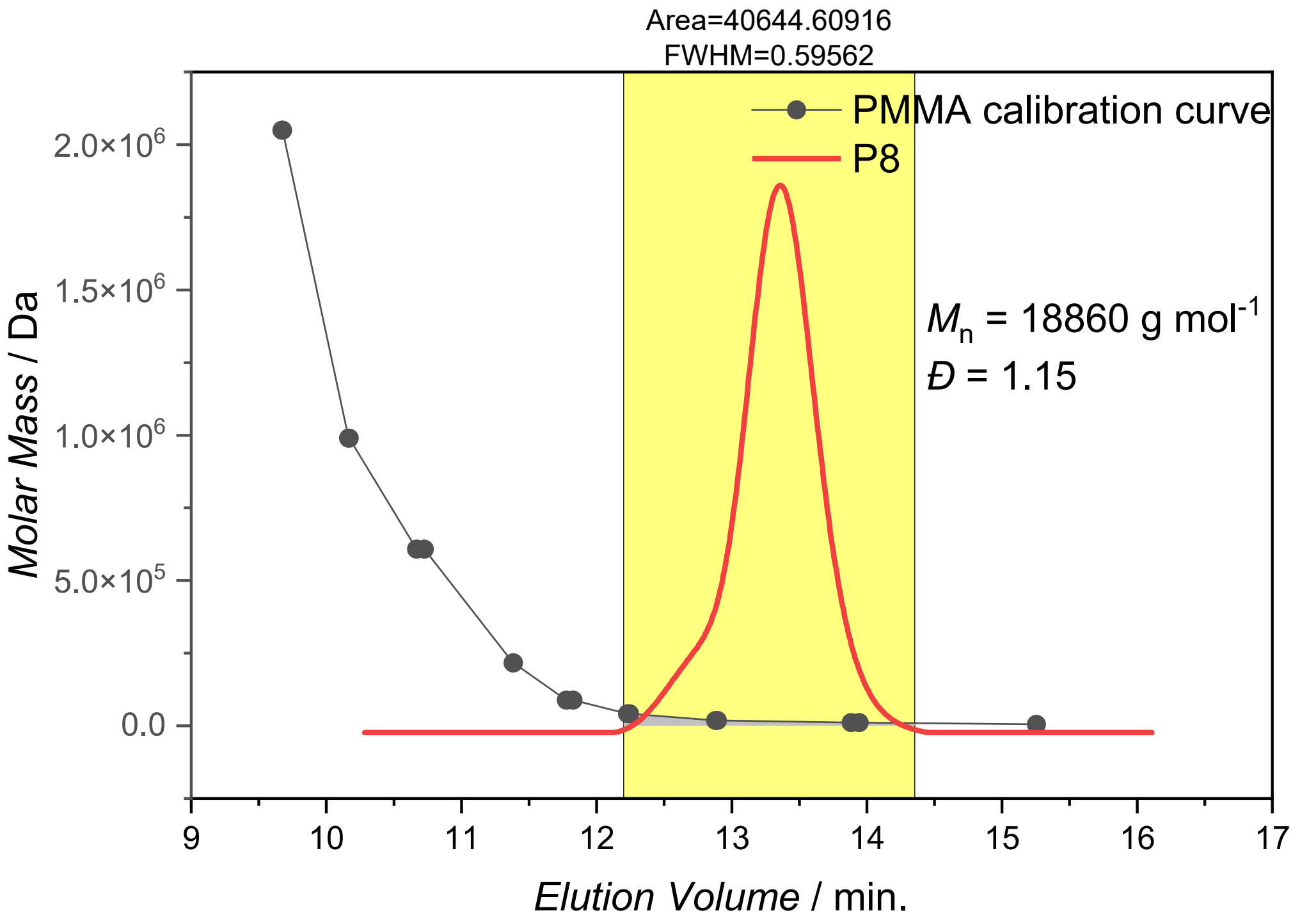

Figure S139. SEC analysis of **P8** recorded in DMAc, showing the PMMA calibration curve (black) and the corresponding RI trace (red) used for the calculation of the apparent number-average molar mass and dispersity. The highlighted region indicates the integration range used for the SEC analysis.

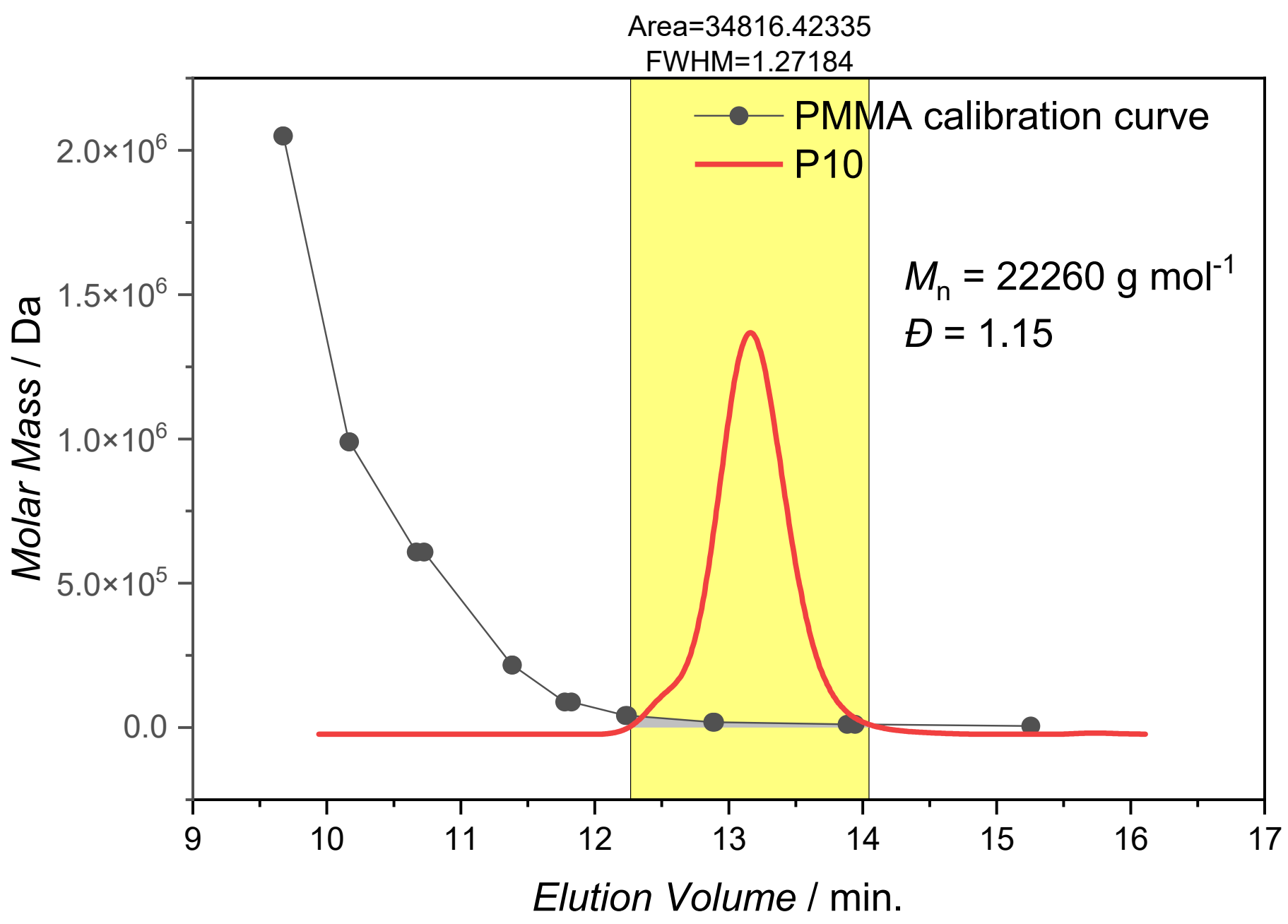

Figure S140. SEC analysis of **P10** recorded in DMAc, showing the PMMA calibration curve (black) and the corresponding RI trace (red) used for the calculation of the apparent number-average molar mass and dispersity. The highlighted region indicates the integration range used for the SEC analysis.

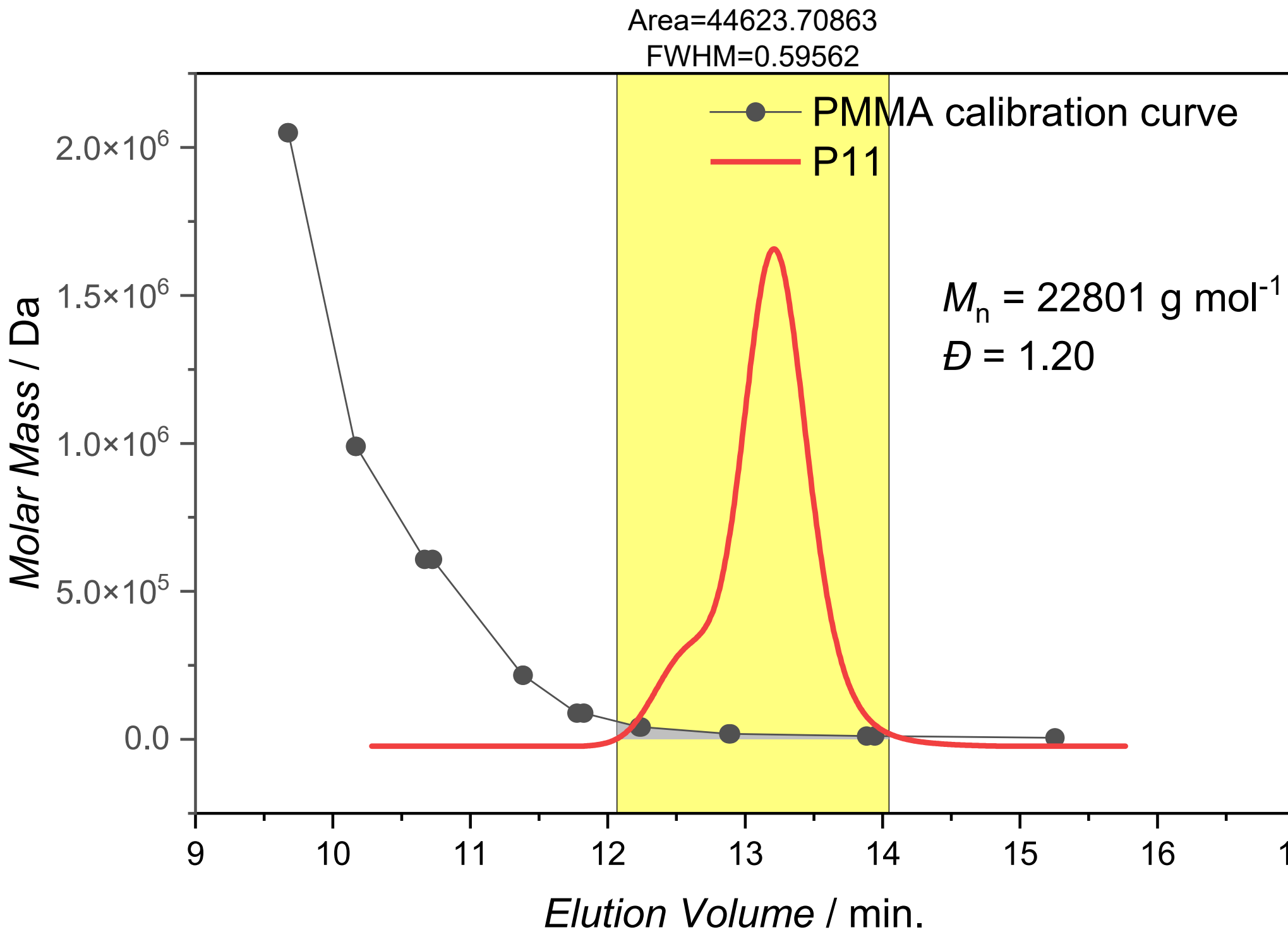

Figure S141. SEC analysis of **P11** recorded in DMAc, showing the PMMA calibration curve (black) and the corresponding RI trace (red) used for the calculation of the apparent number-average molar mass and dispersity. The highlighted region indicates the integration range used for the SEC analysis.

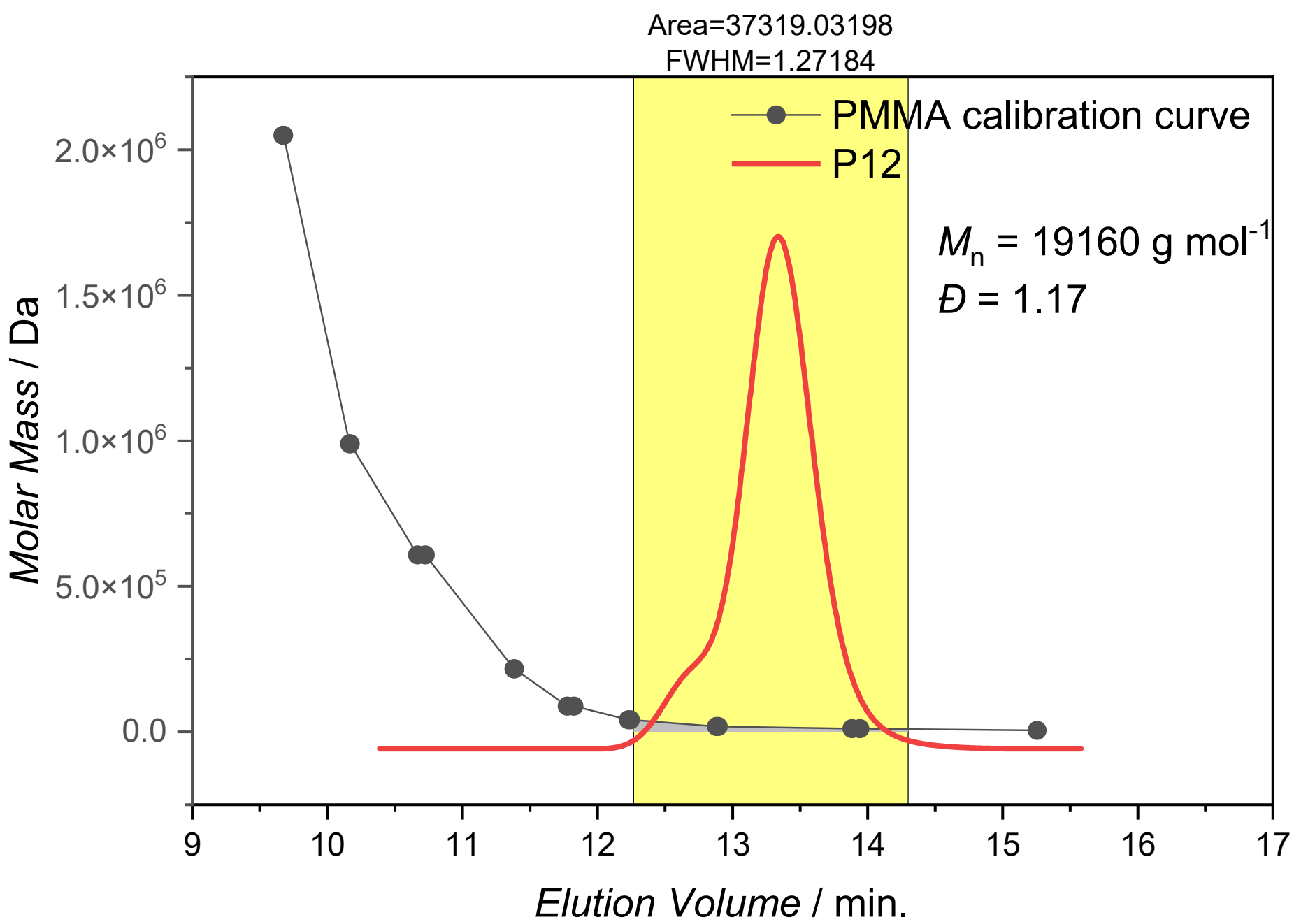

Figure S142. SEC analysis of **P12** recorded in DMAc, showing the PMMA calibration curve (black) and the corresponding RI trace (red) used for the calculation of the apparent number-average molar mass and dispersity. The highlighted region indicates the integration range used for the SEC analysis.

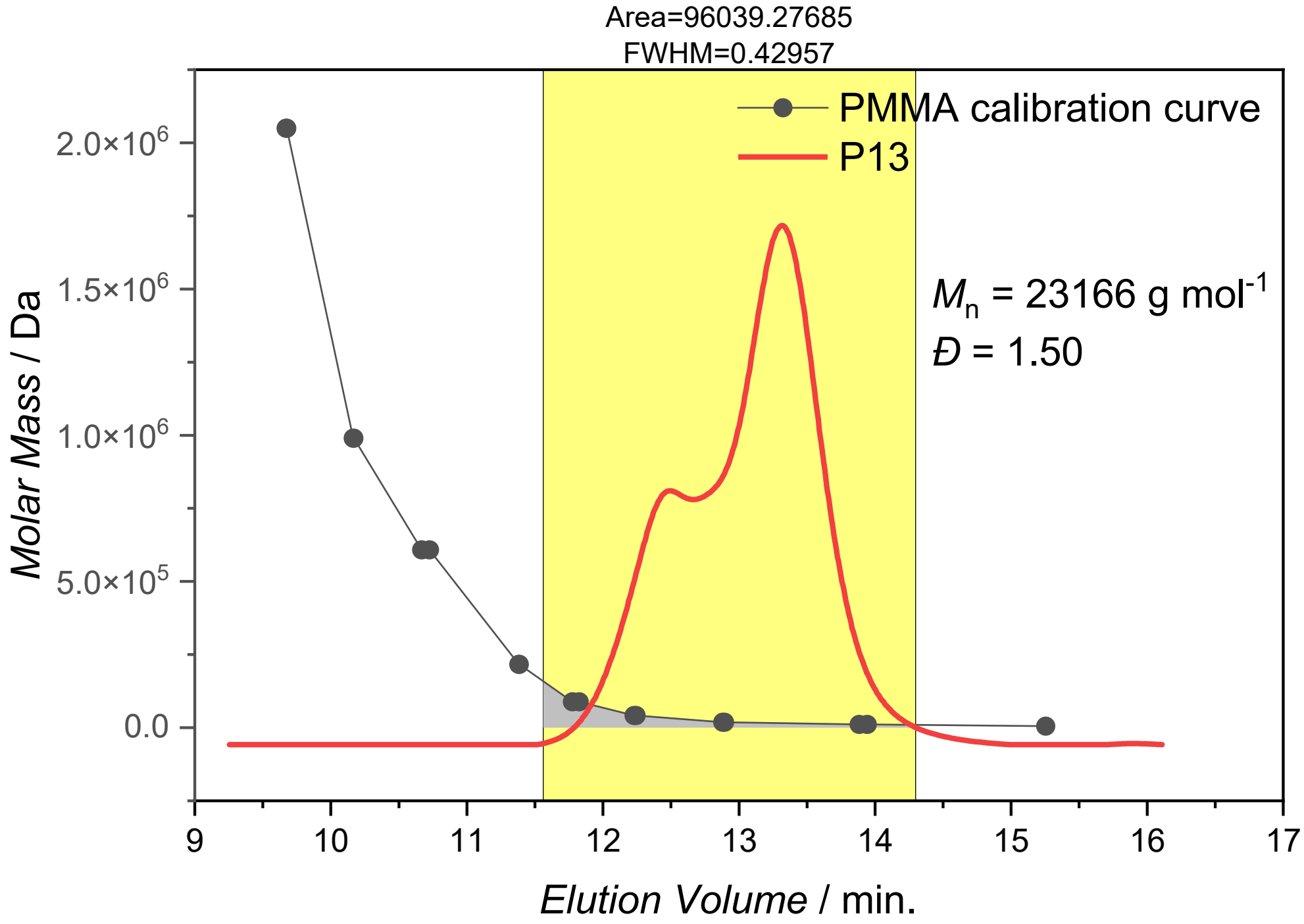

Figure S143. SEC analysis of **P13** recorded in DMAc, showing the PMMA calibration curve (black) and the corresponding RI trace (red) used for the calculation of the apparent number-average molar mass and dispersity. The highlighted region indicates the integration range used for the SEC analysis.

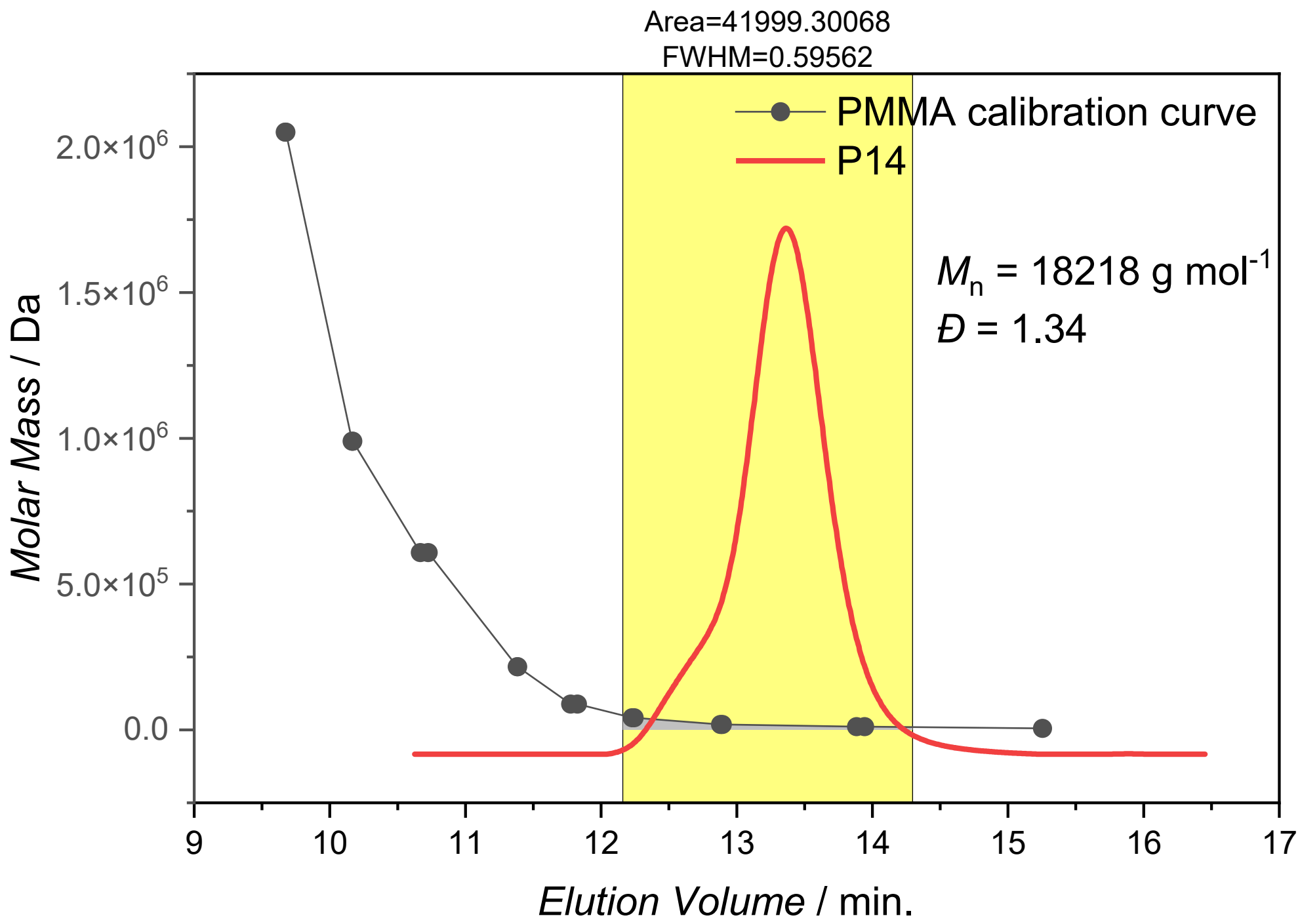

Figure S144. SEC analysis of **P14** recorded in DMAc, showing the PMMA calibration curve (black) and the corresponding RI trace (red) used for the calculation of the apparent number-average molar mass and dispersity. The highlighted region indicates the integration range used for the SEC analysis.

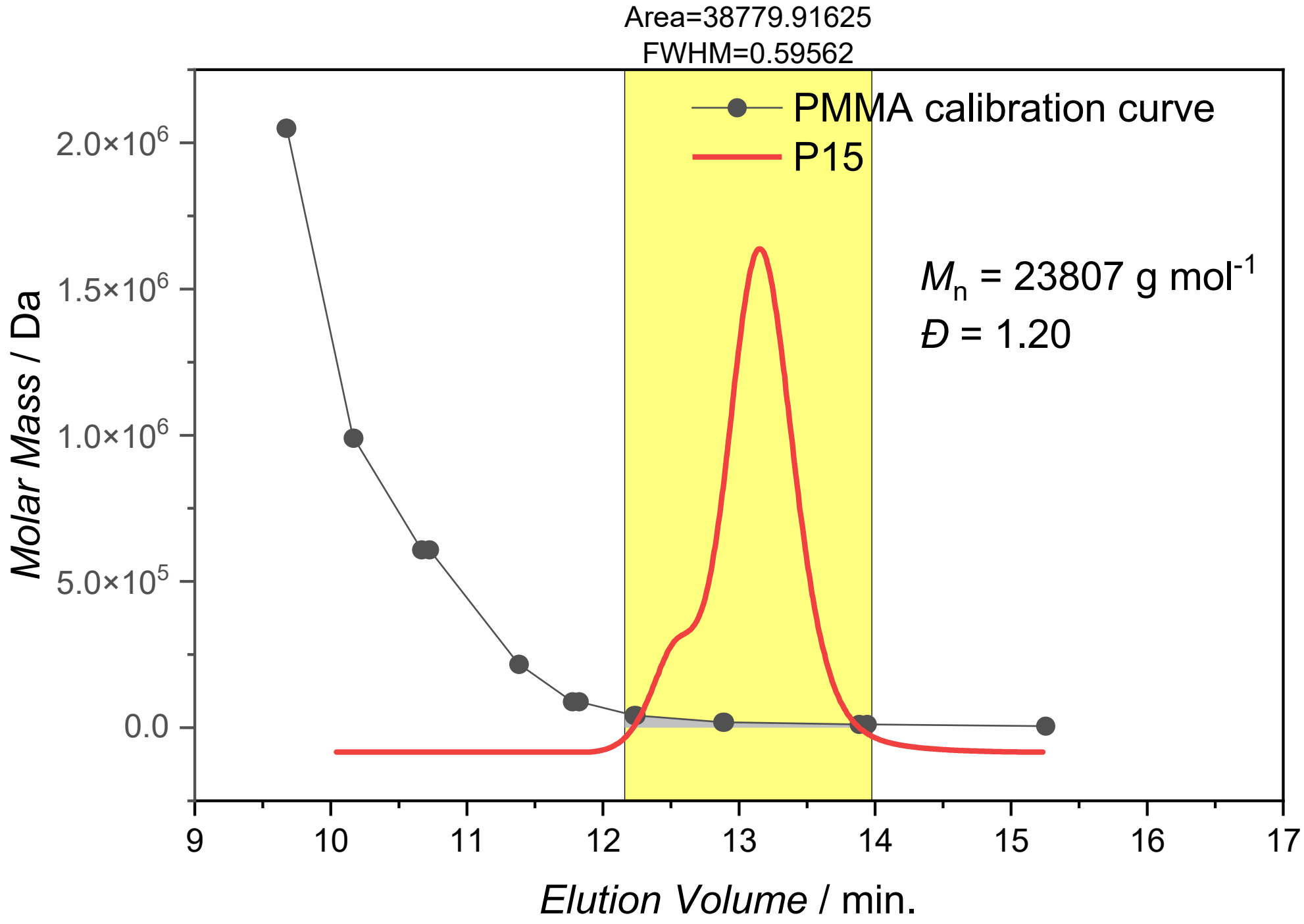

Figure S145. SEC analysis of **P15** recorded in DMAc, showing the PMMA calibration curve (black) and the corresponding RI trace (red) used for the calculation of the apparent number-average molar mass and dispersity. The highlighted region indicates the integration range used for the SEC analysis.

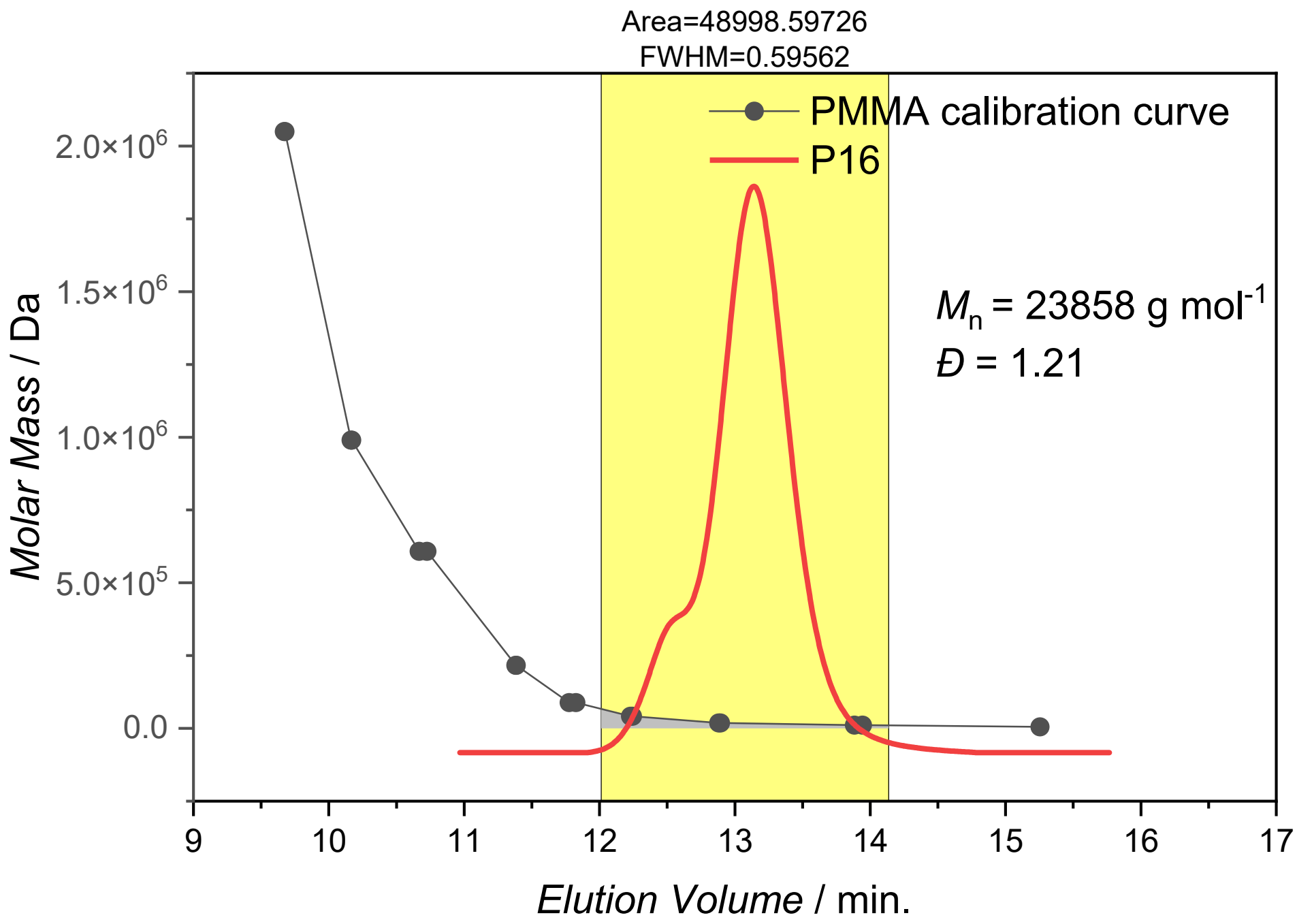

Figure S146. SEC analysis of **P16** recorded in DMAc, showing the PMMA calibration curve (black) and the corresponding RI trace (red) used for the calculation of the apparent number-average molar mass and dispersity. The highlighted region indicates the integration range used for the SEC analysis.

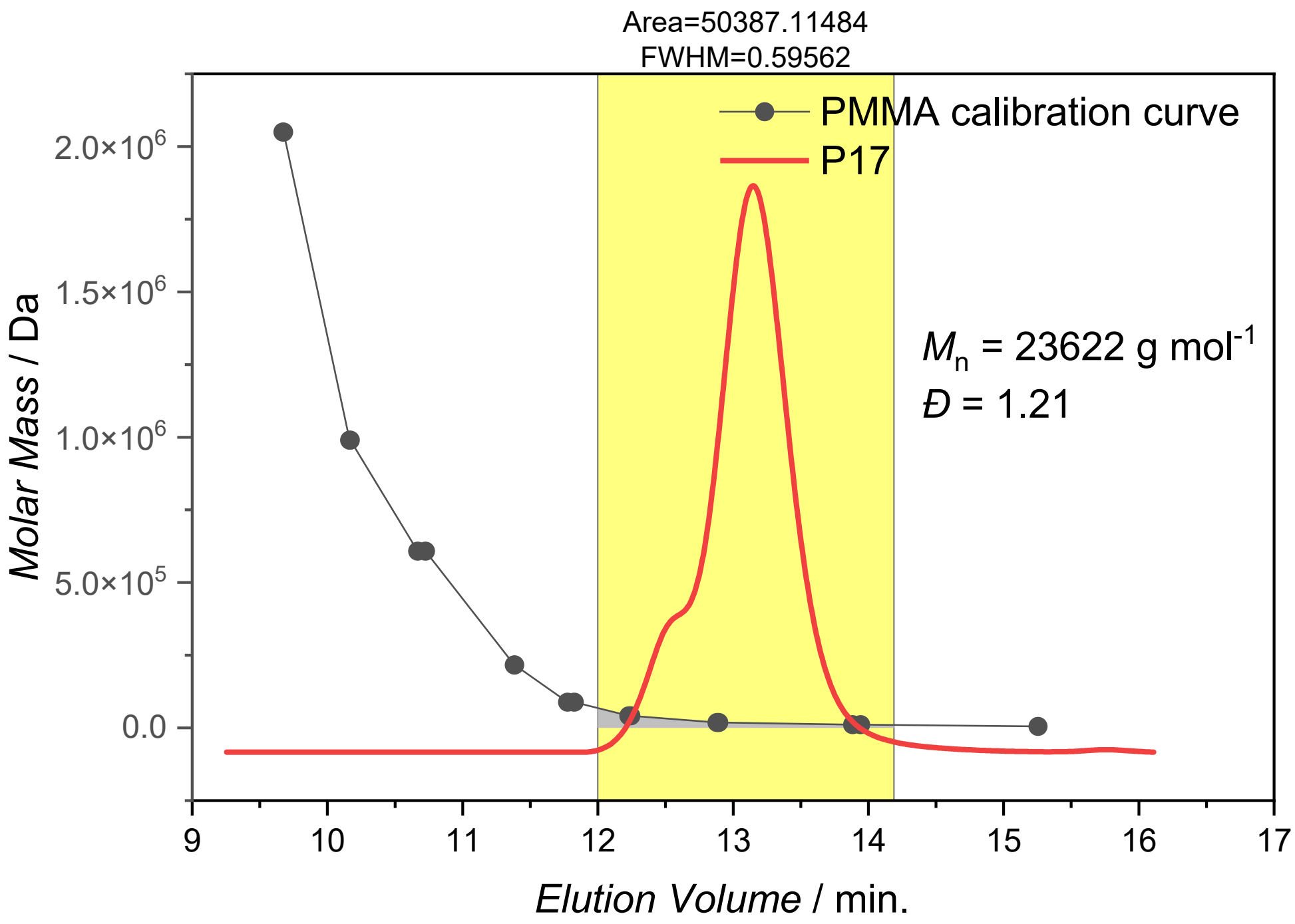

Figure S147. SEC analysis of **P17** recorded in DMAc, showing the PMMA calibration curve (black) and the corresponding RI trace (red) used for the calculation of the apparent number-average molar mass and dispersity. The highlighted region indicates the integration range used for the SEC analysis.

C.4 DSC analysis

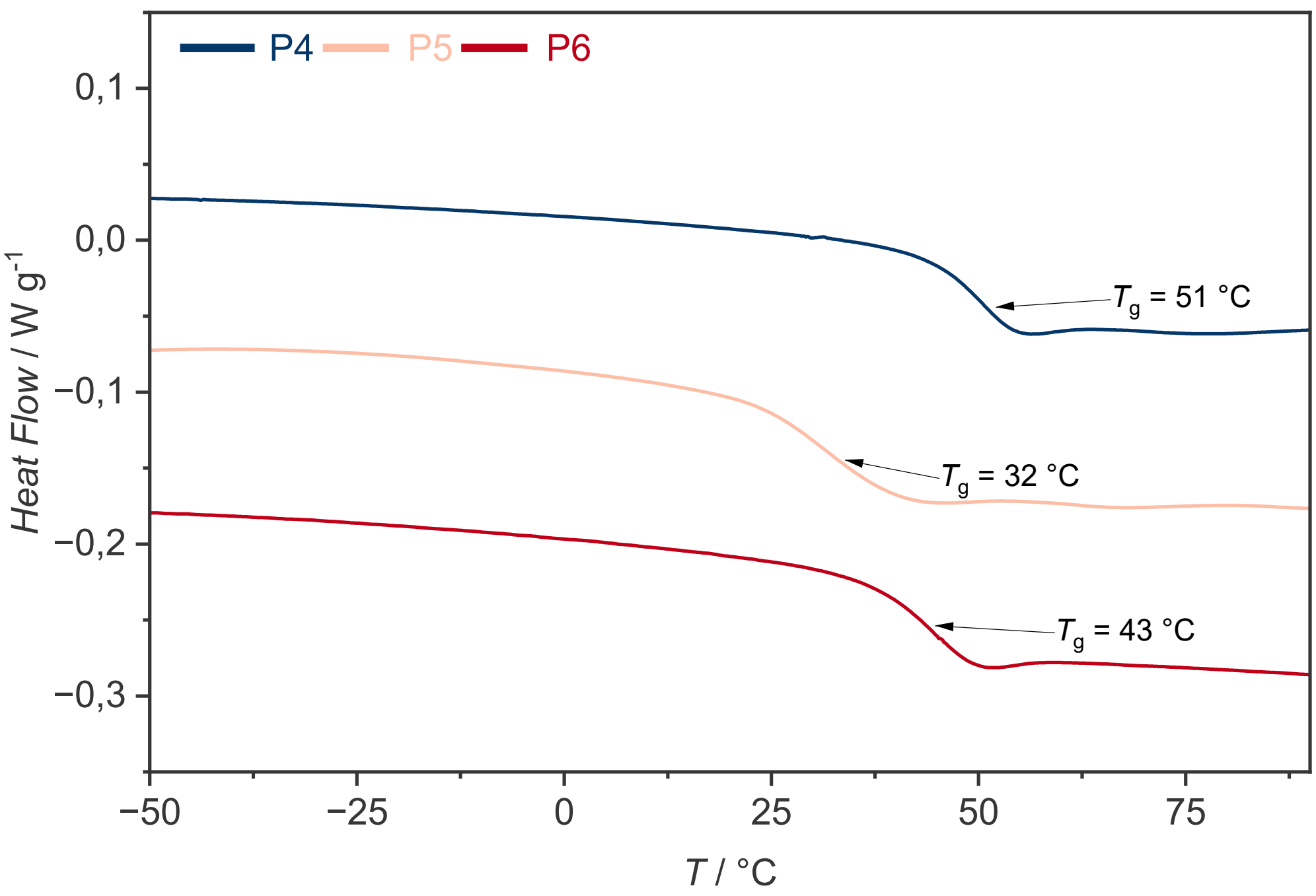

Figure S148. DSC thermograms of **P4-P6** recorded during the second heating cycle. Glass transition temperatures (T_g) were determined at the inflection point of the respective heat-capacity transition and are indicated in the plot.

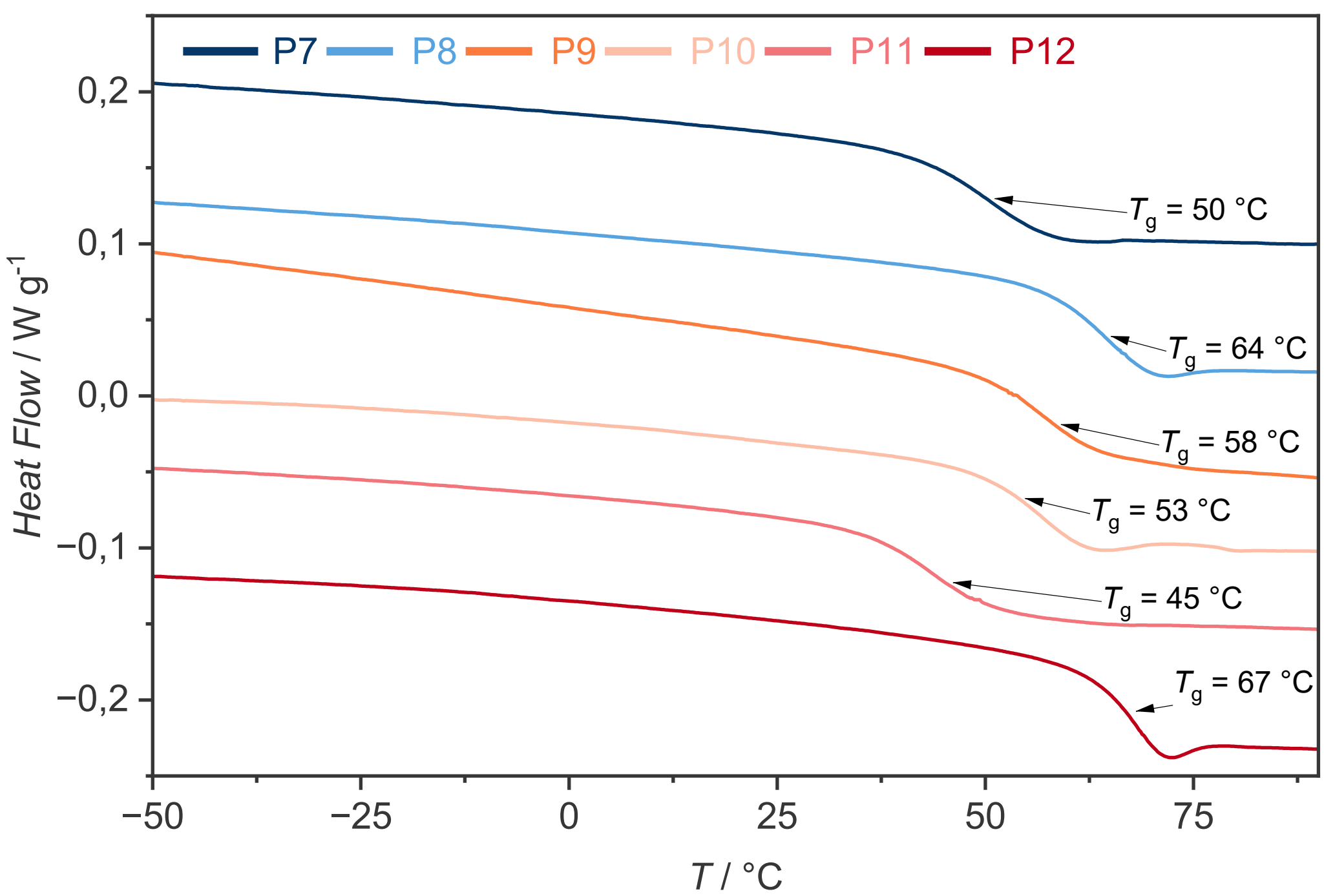

Figure S149. DSC thermograms of **P7-P12** recorded during the second heating cycle. Glass transition temperatures (T_g) were determined at the inflection point of the respective heat-capacity transition and are indicated in the plot.

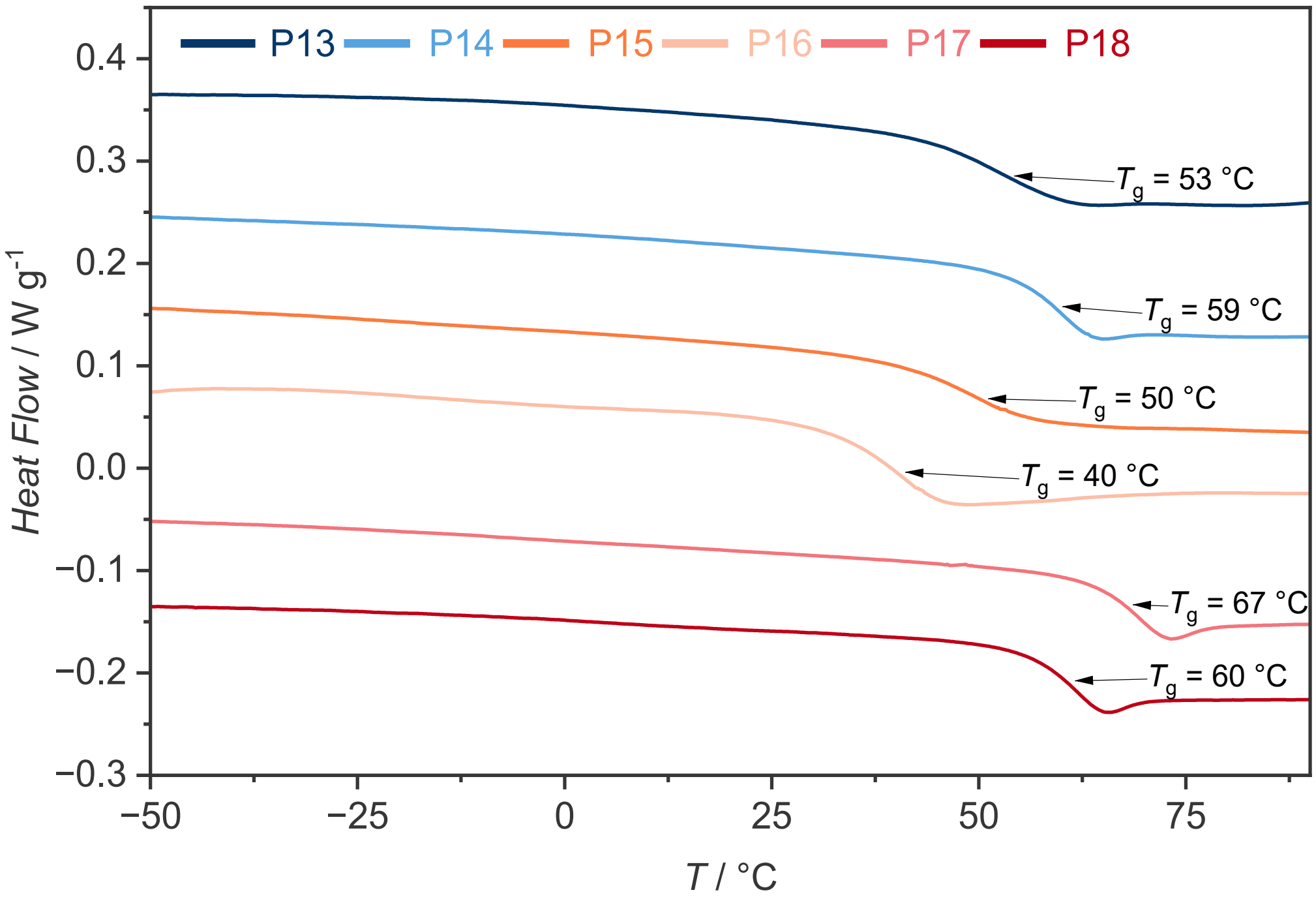

Figure S150. DSC thermograms of **P13-P18** recorded during the second heating cycle. Glass transition temperatures (T_g) were determined at the inflection point of the respective heat-capacity transition and are indicated in the plot.

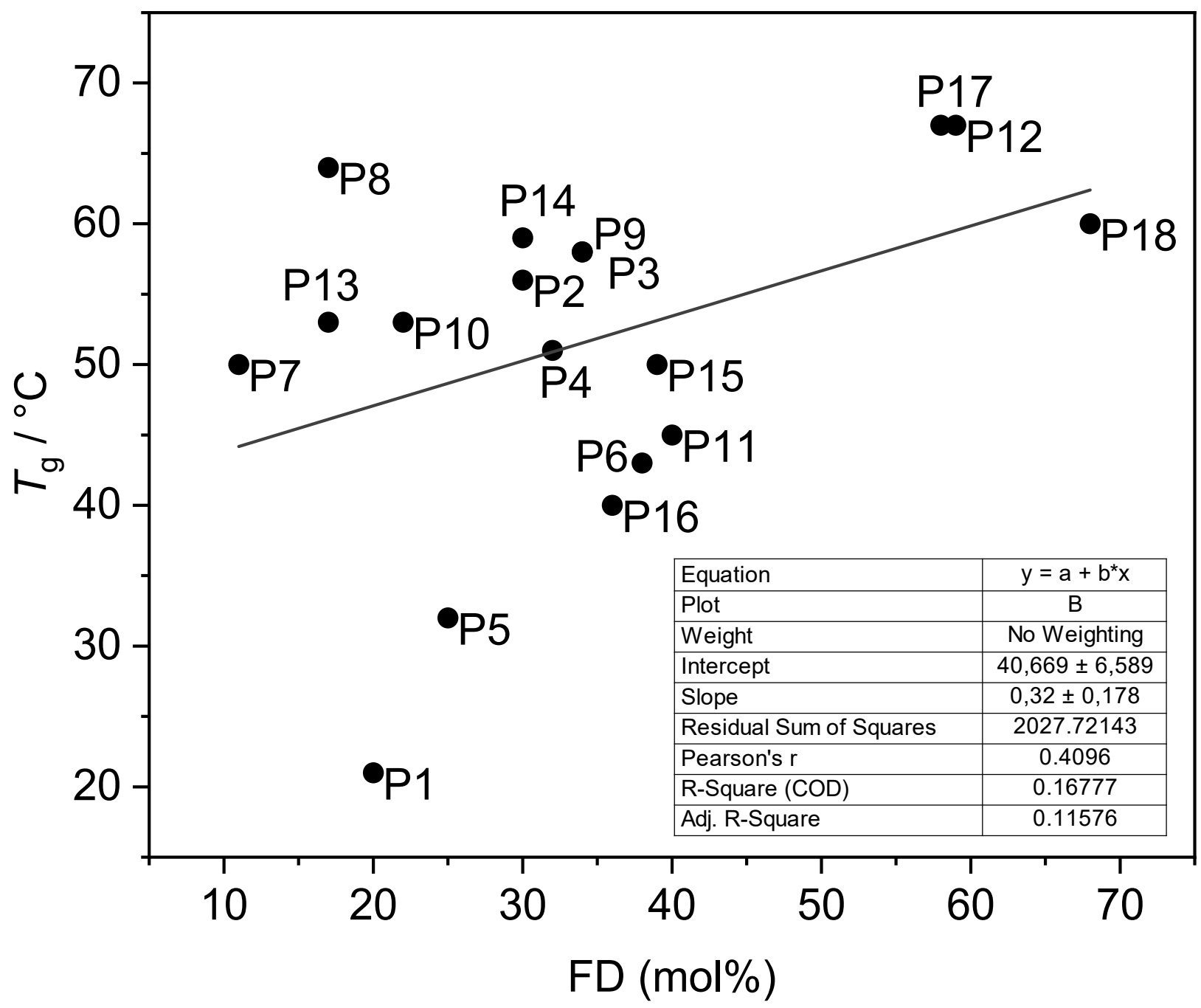

Figure S151. Relationship between average FD and T_g across **P1-P18**. No linear relationship was observed (R^2 = 0.17).

C.5 TGA analysis

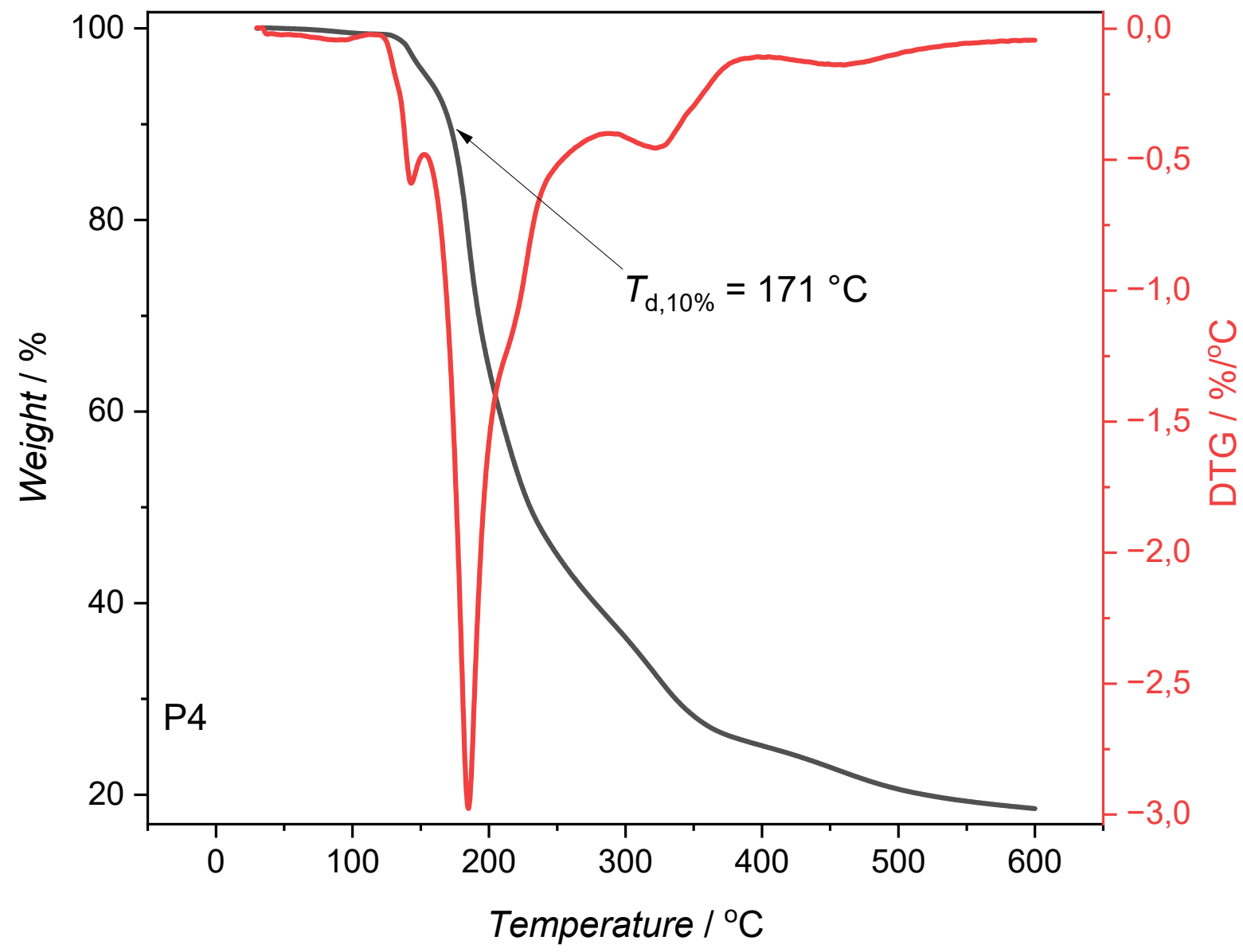

Figure S152. TGA thermogram and corresponding DTG trace of **P4**.

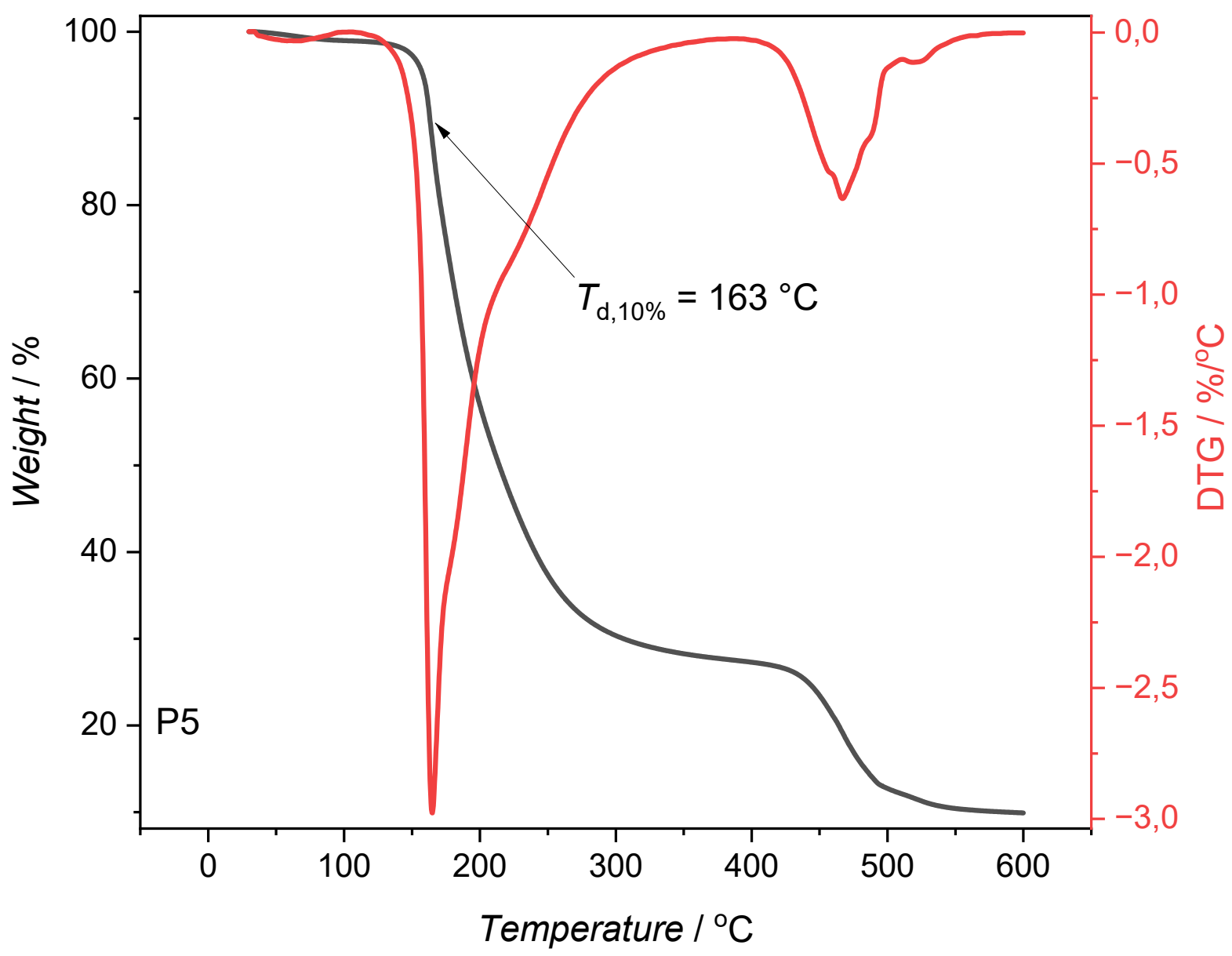

Figure S153. TGA thermogram and corresponding DTG trace of **P5**.

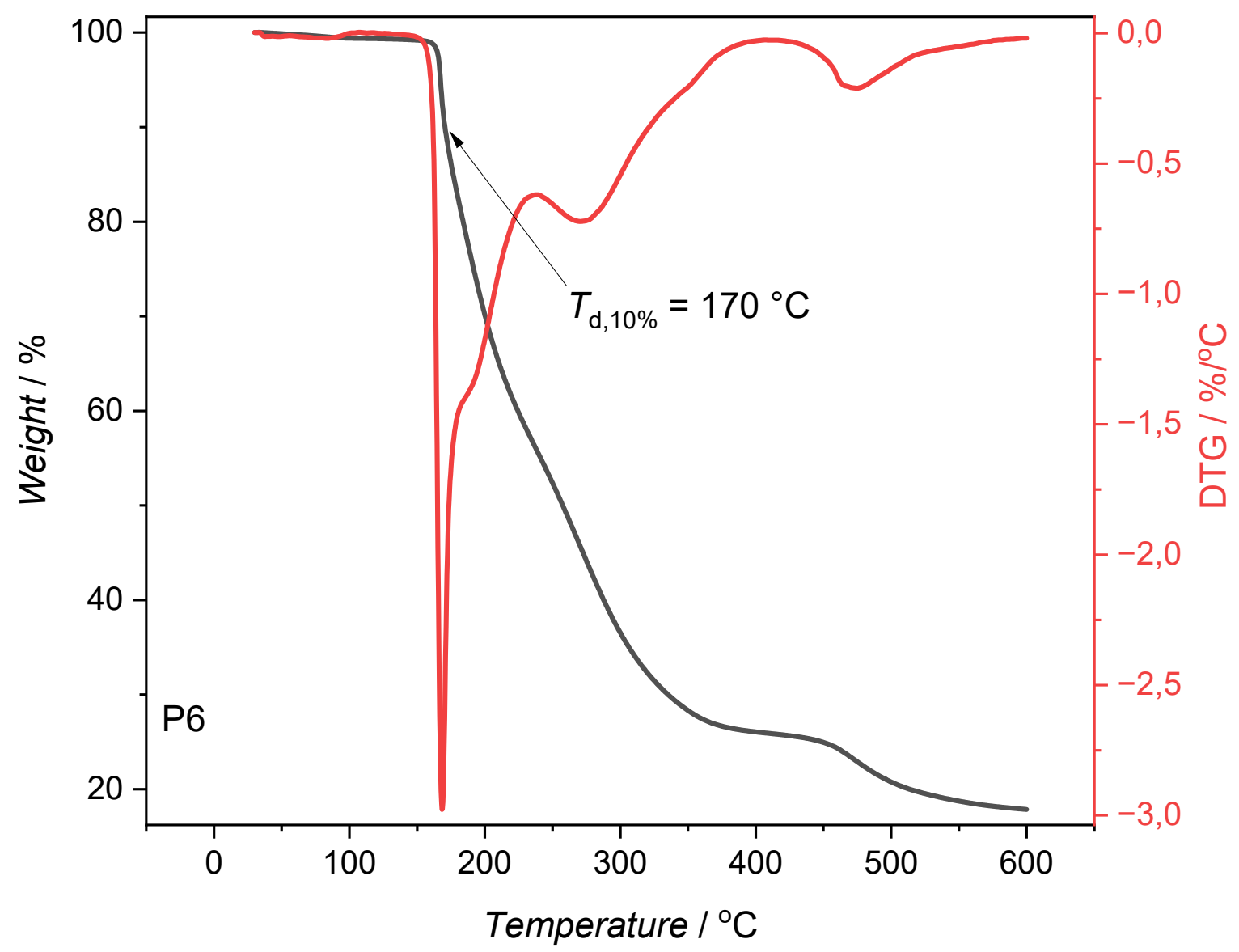

Figure S154. TGA thermogram and corresponding DTG trace of **P6**.

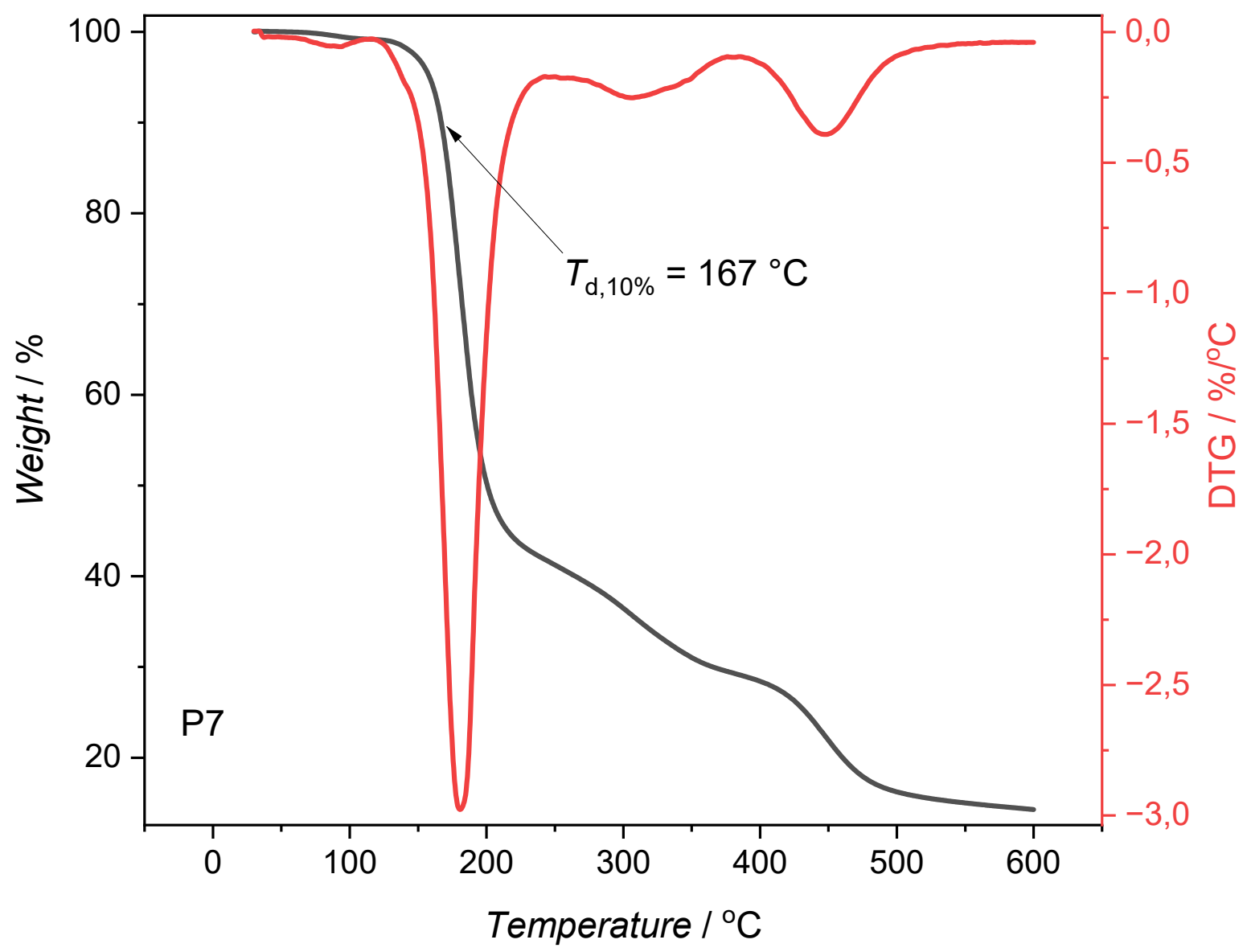

Figure S155. TGA thermogram and corresponding DTG trace of **P7**.

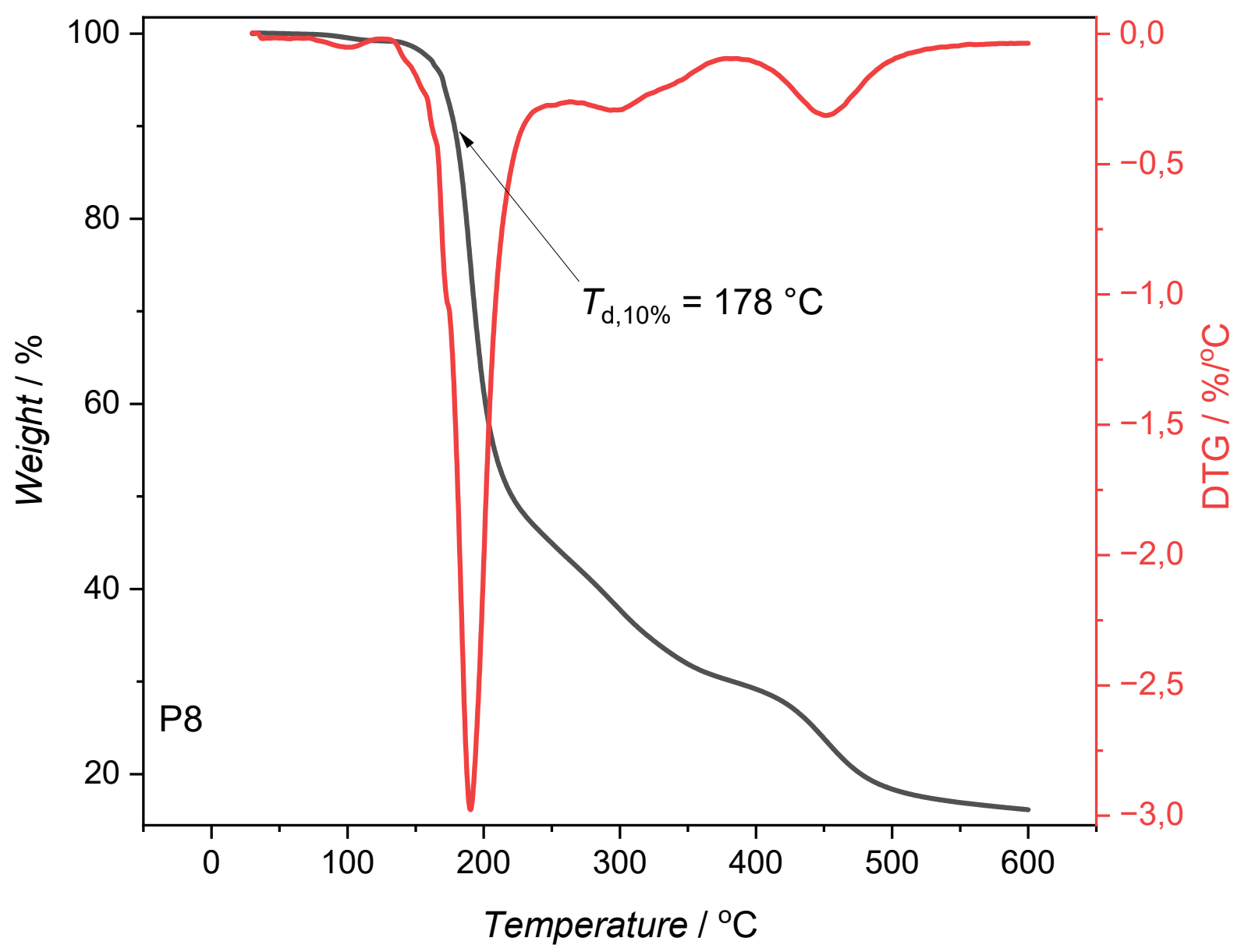

Figure S156. TGA thermogram and corresponding DTG trace of **P8**.

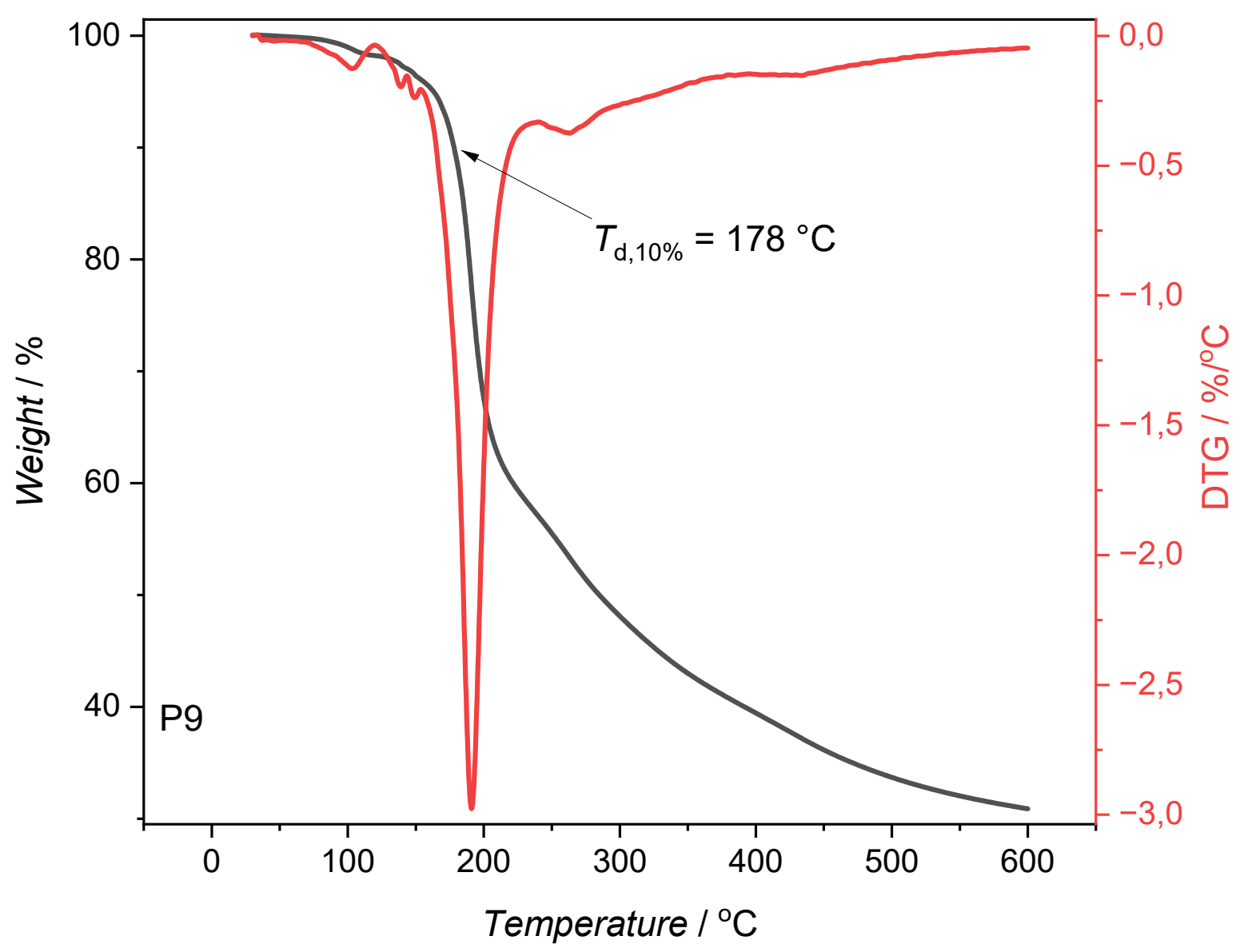

Figure S157. TGA thermogram and corresponding DTG trace of **P9**.

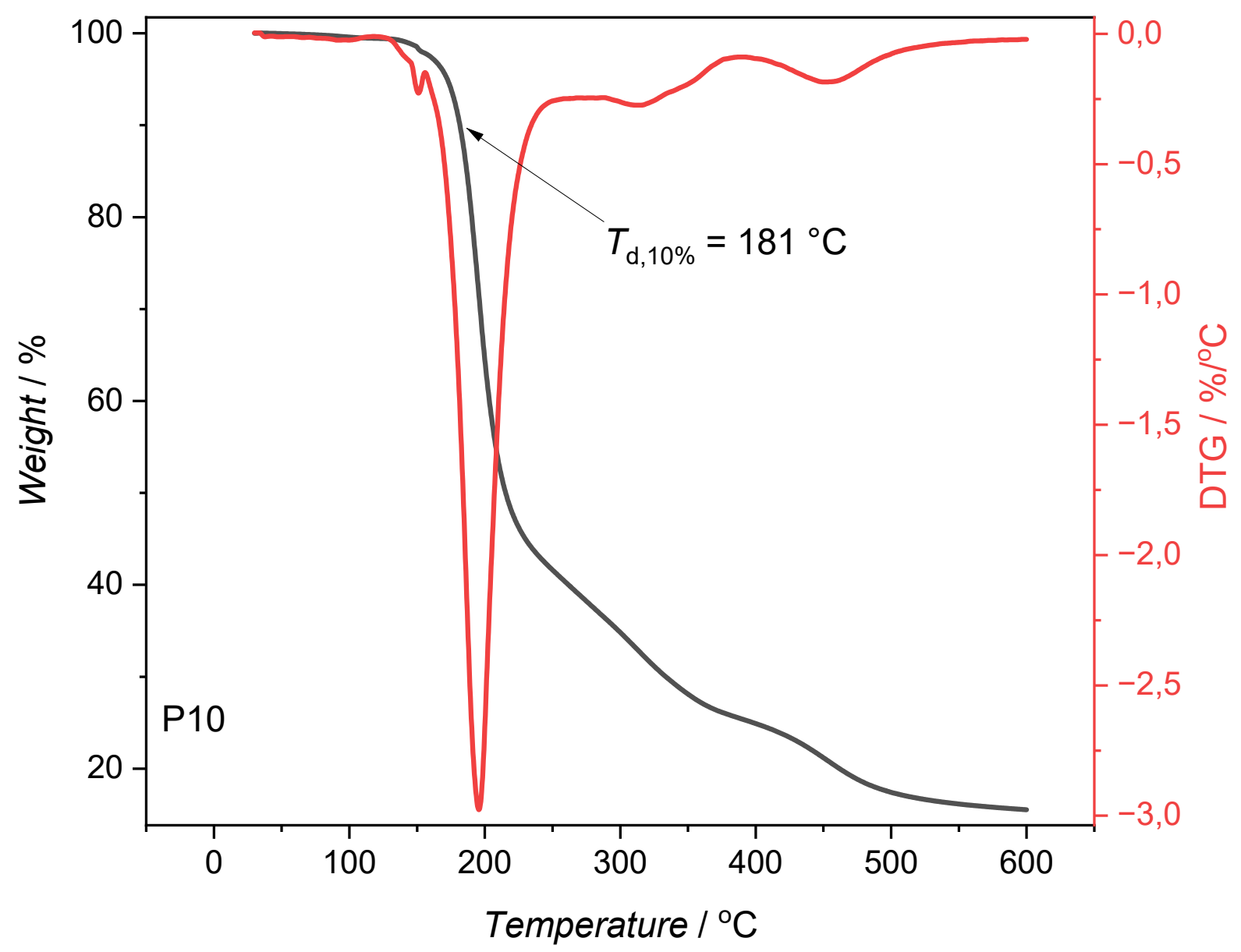

Figure S158. TGA thermogram and corresponding DTG trace of **P10**.

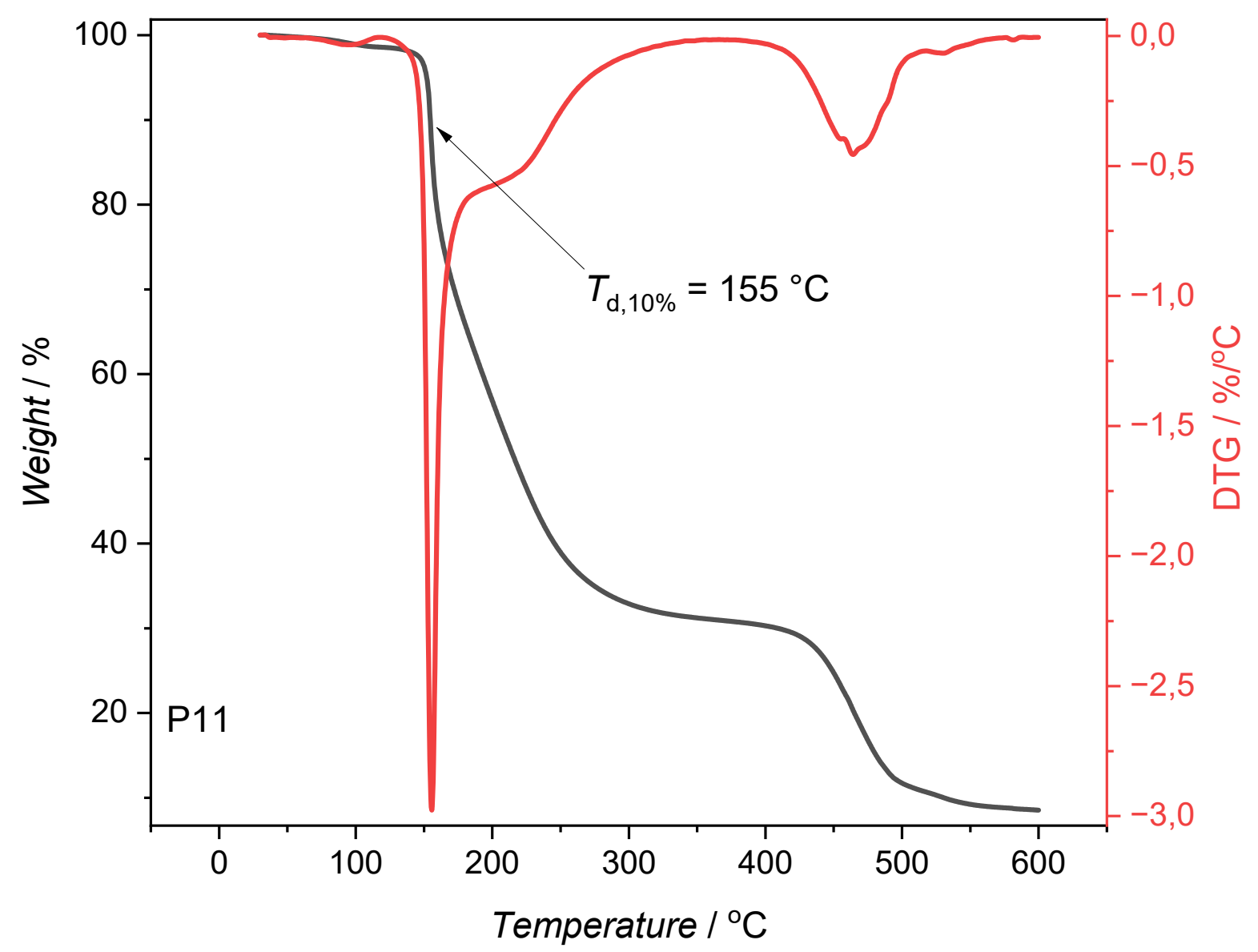

Figure S159. TGA thermogram and corresponding DTG trace of **P11**.

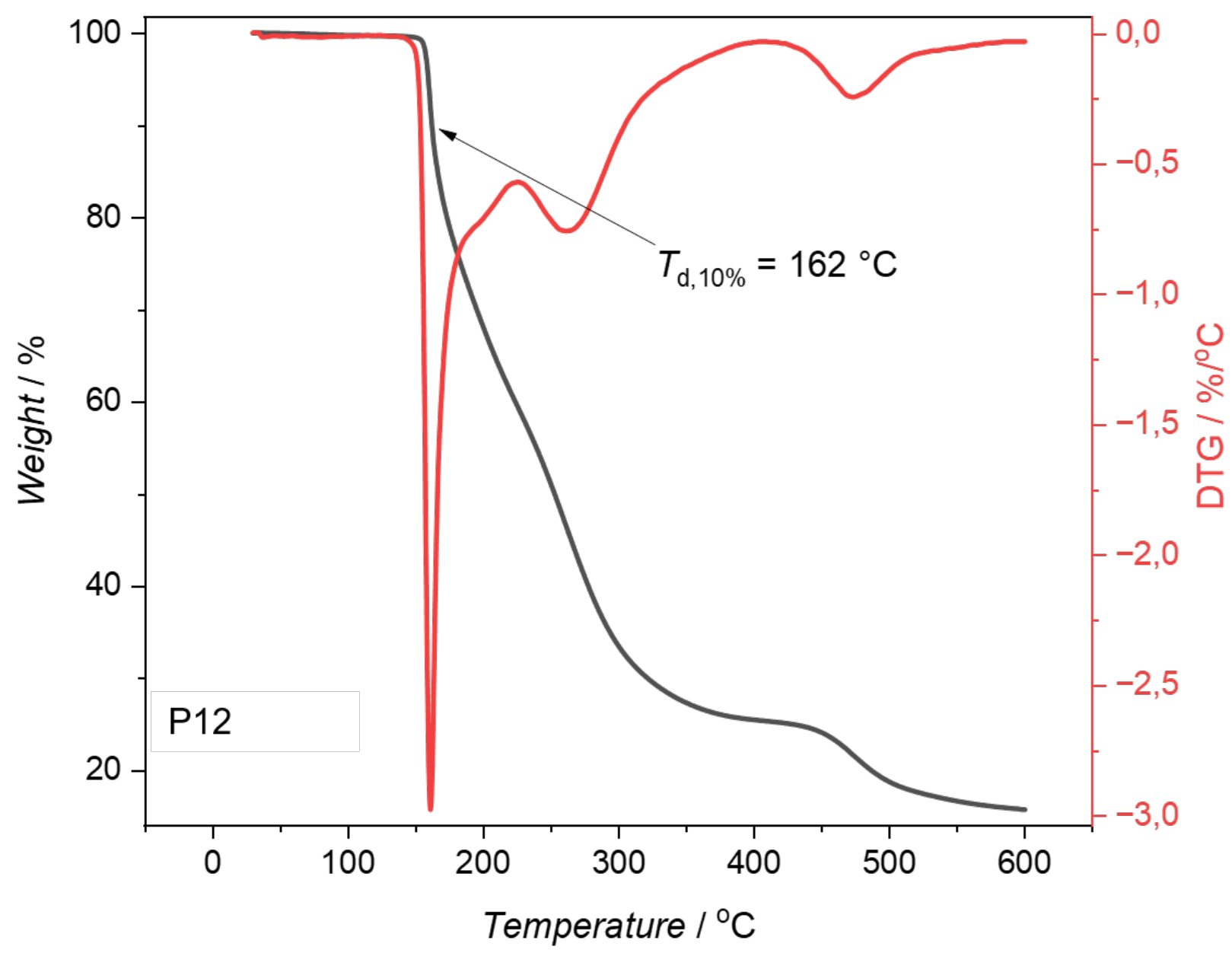

Figure S160. TGA thermogram and corresponding DTG trace of **P12**.

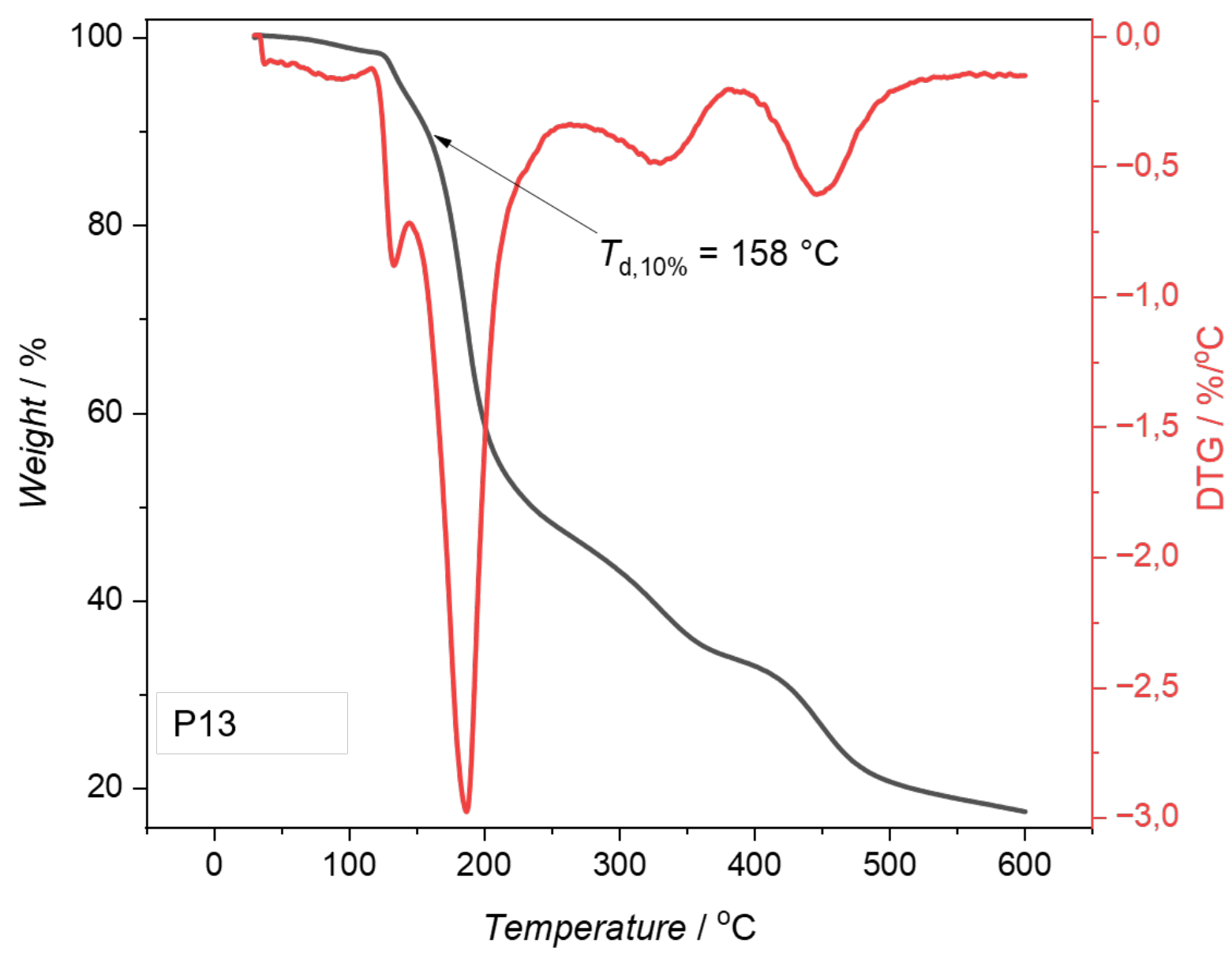

Figure S161. TGA thermogram and corresponding DTG trace of **P13**.

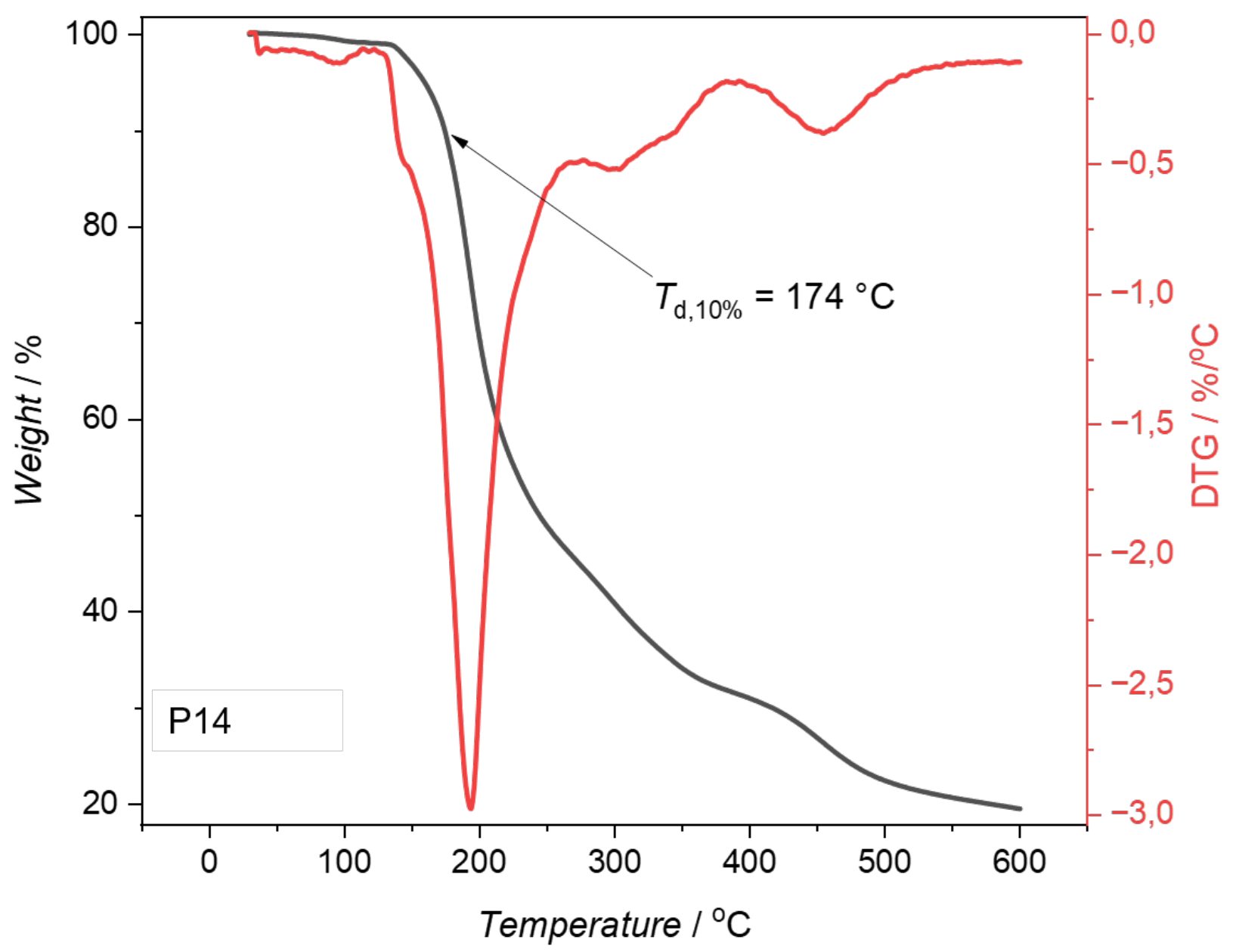

Figure S162. TGA thermogram and corresponding DTG trace of **P14**.

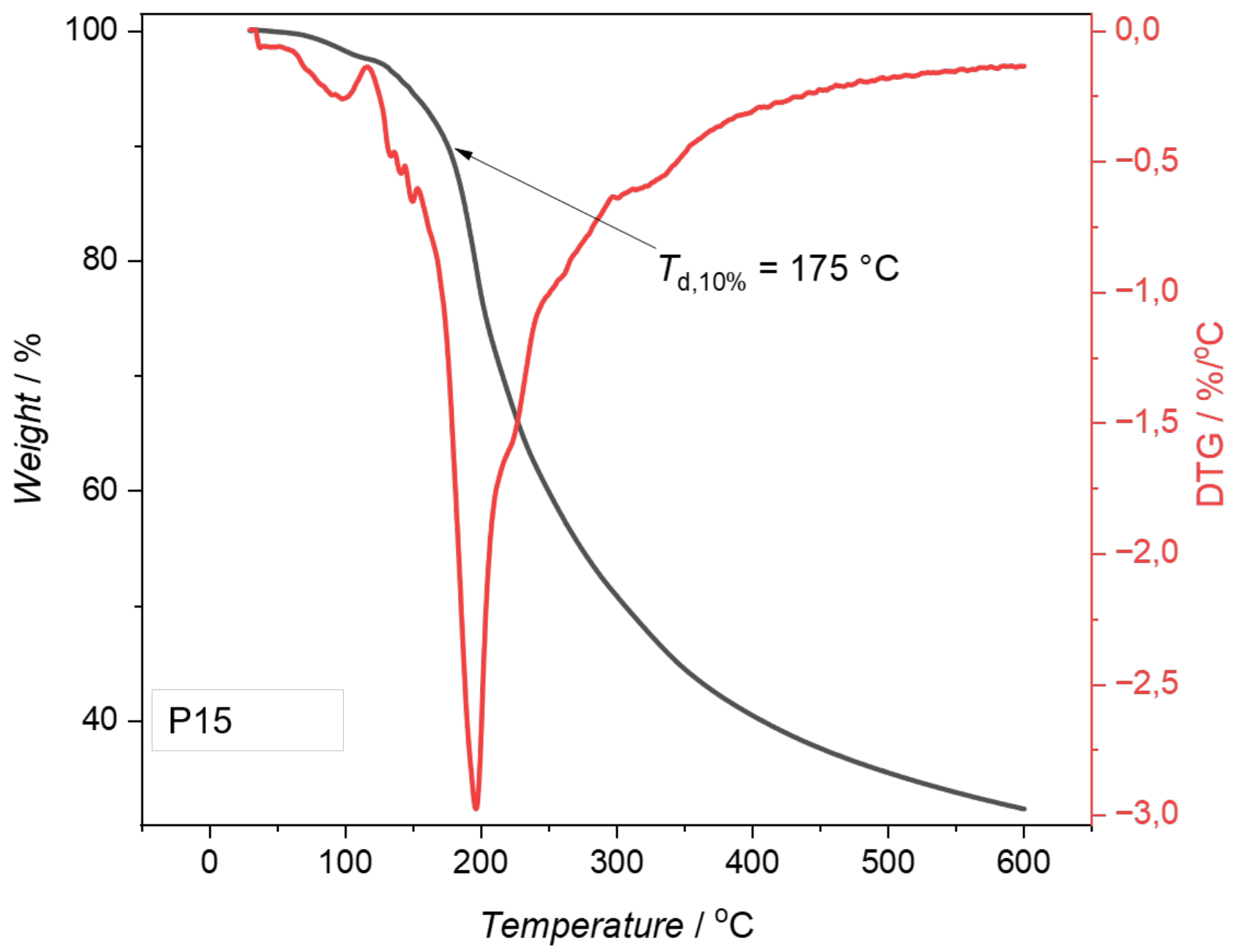

Figure S163. TGA thermogram and corresponding DTG trace of **P15**.

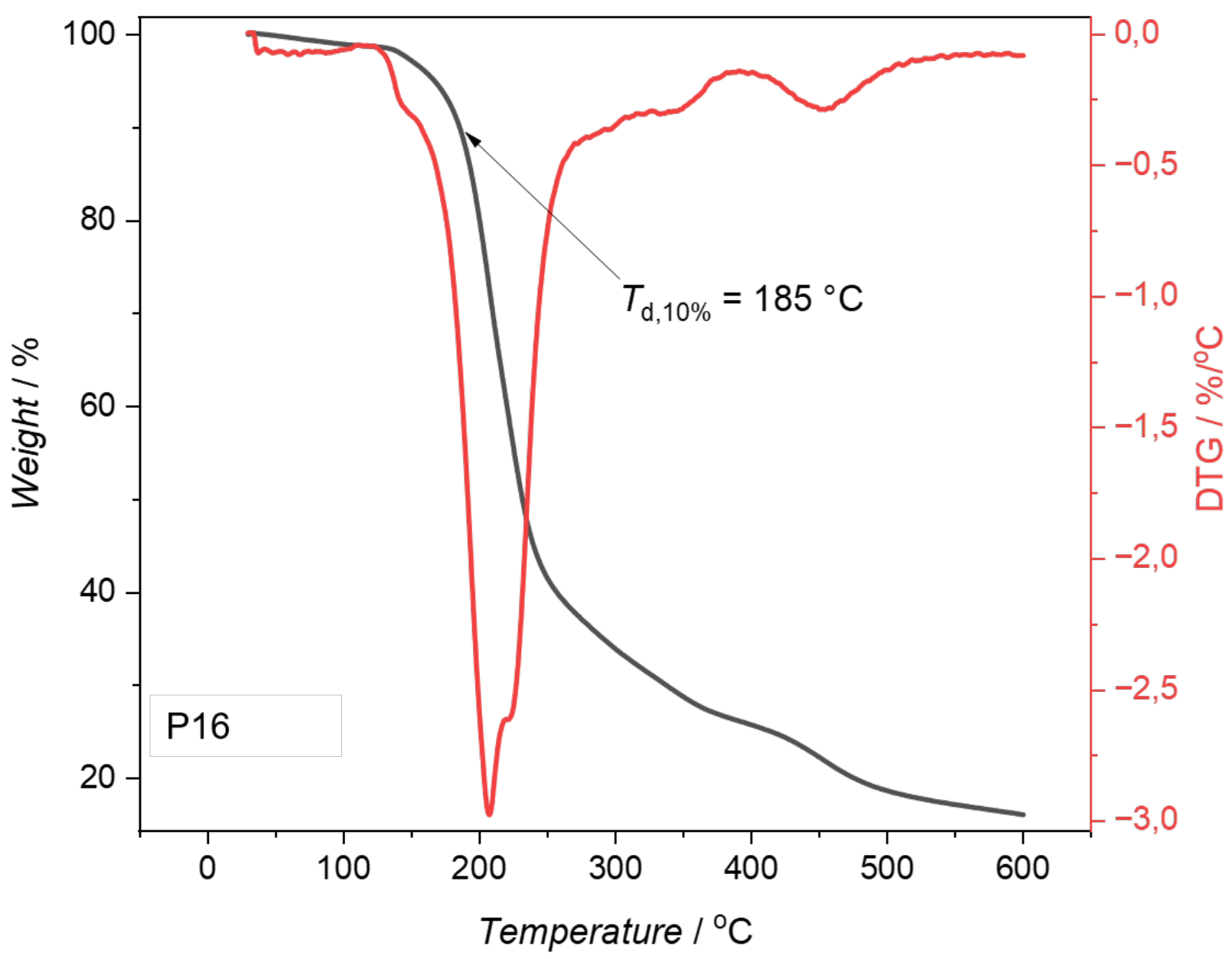

Figure S164. TGA thermogram and corresponding DTG trace of **P16**.

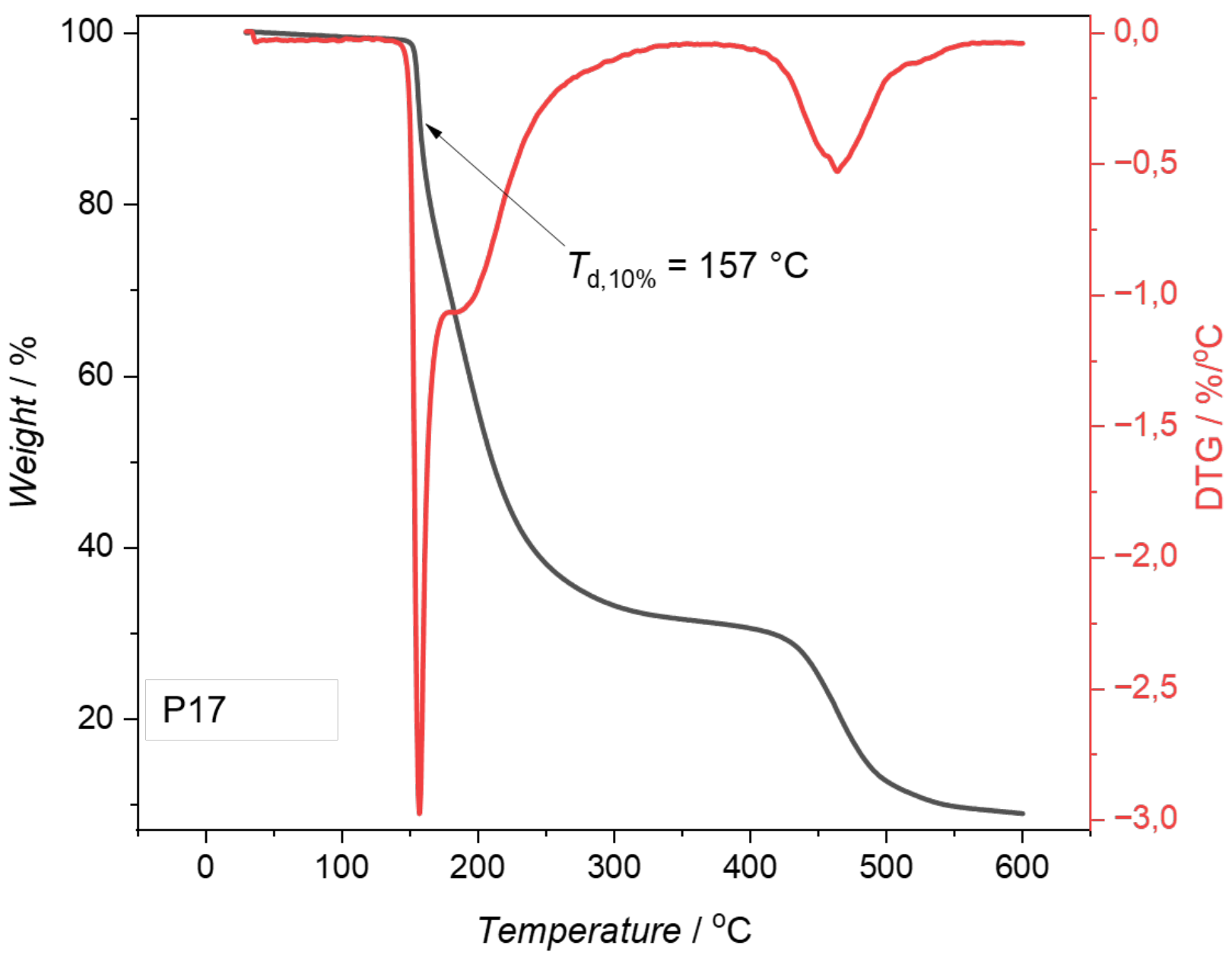

Figure S165. TGA thermogram and corresponding DTG trace of **P17**.

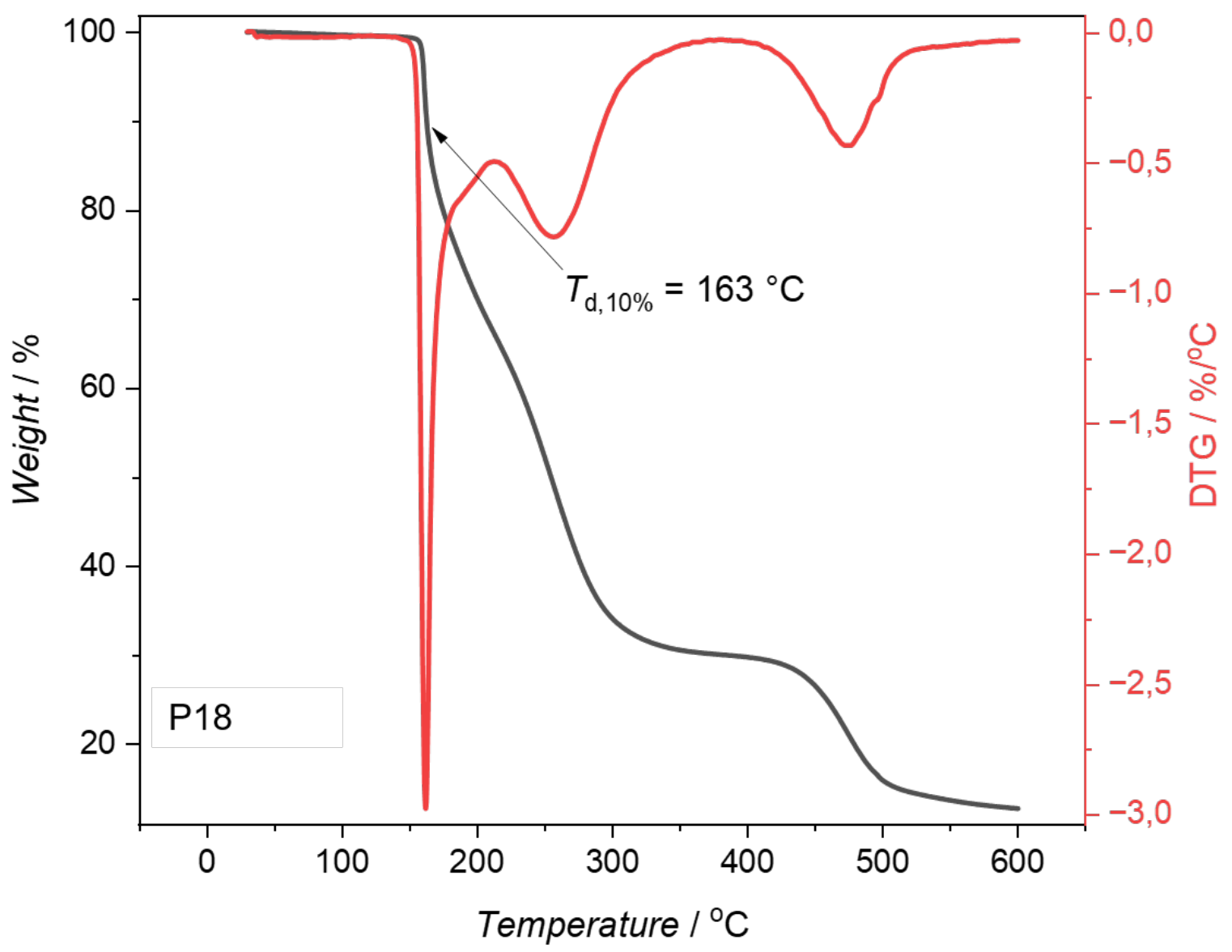

Figure S166. TGA thermogram and corresponding DTG trace of **P18**.

C.6 TEM images

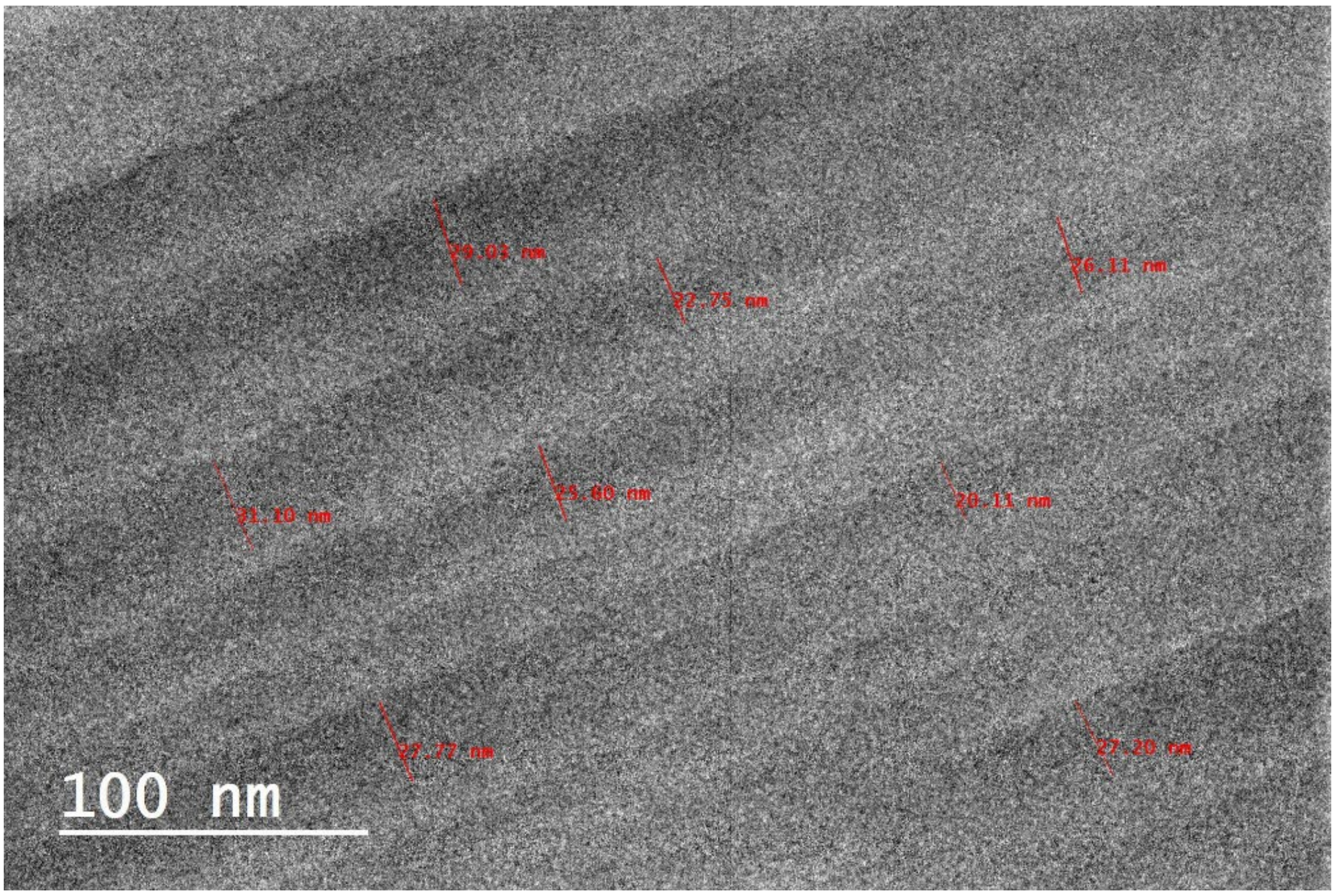

Figure S167. Representative TEM micrograph of **P9** showing lamellar nanoscale contrast with characteristic spacings of approximately 20–30 nm. Representative spacing measurements are indicated.

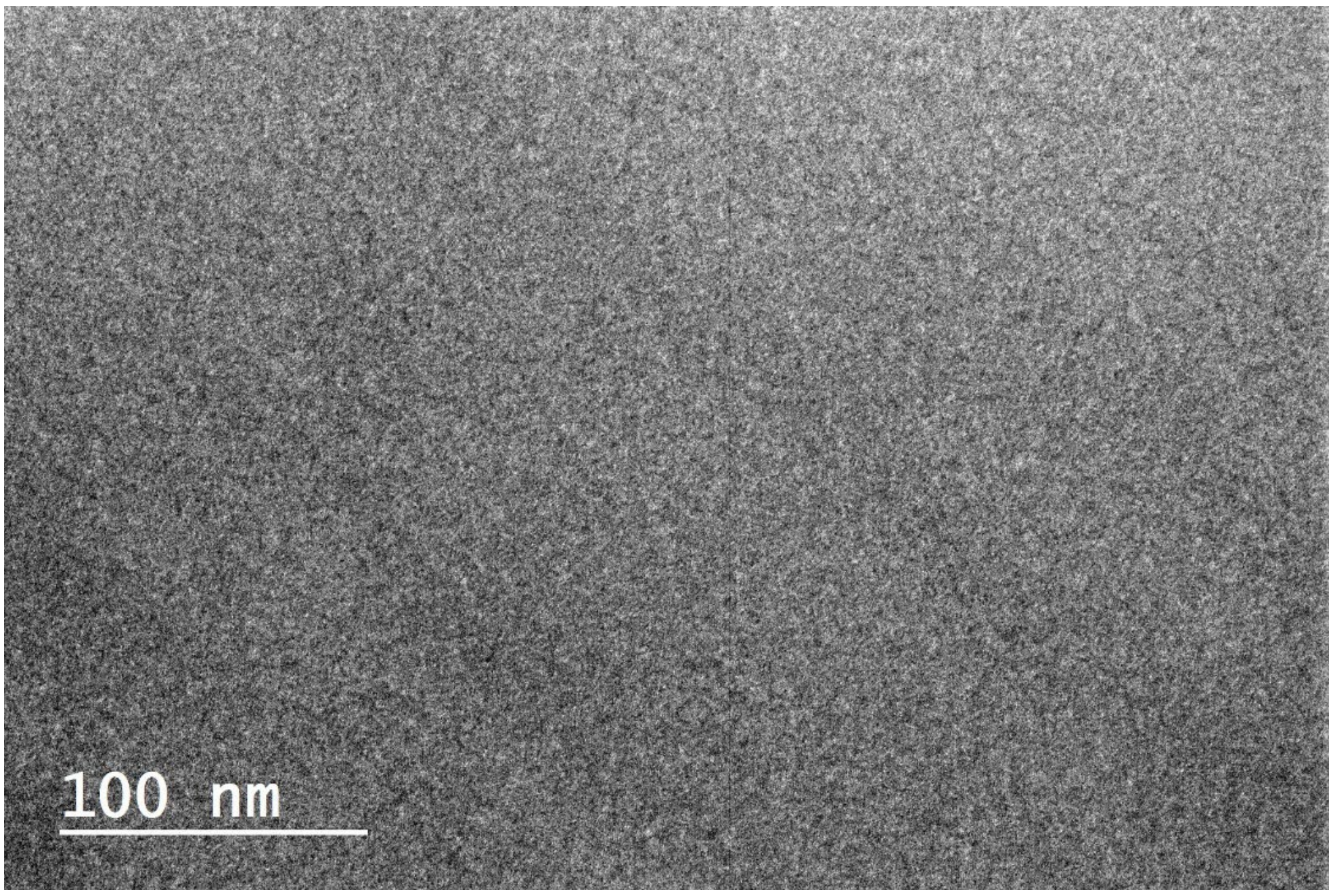

Figure S168. Representative TEM micrograph of **P2**, showing no pronounced nanoscale contrast under the applied imaging conditions.

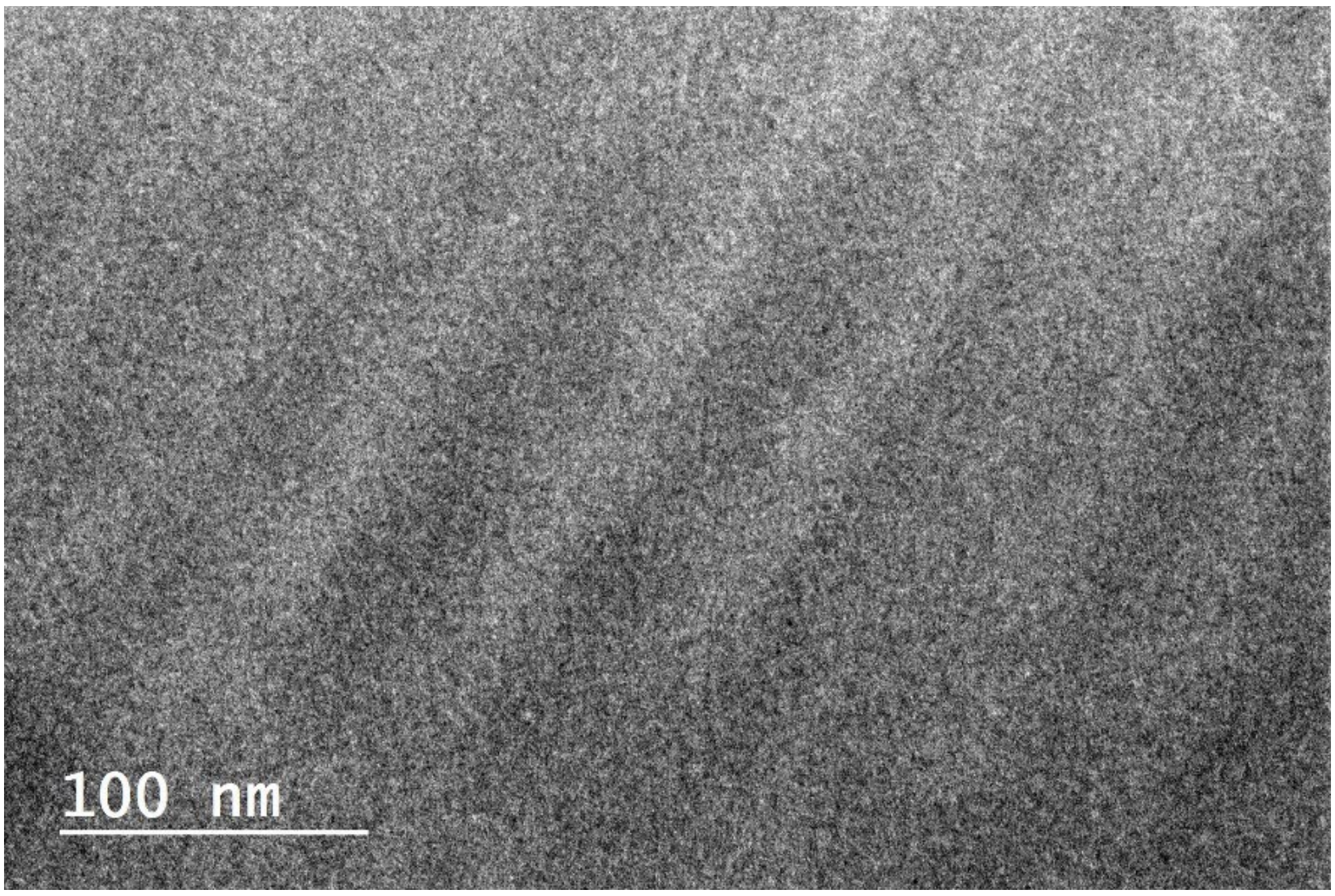

Figure S169. Representative TEM micrograph of **P3**, showing weak nanoscale contrast.

C.7 **AFM image**

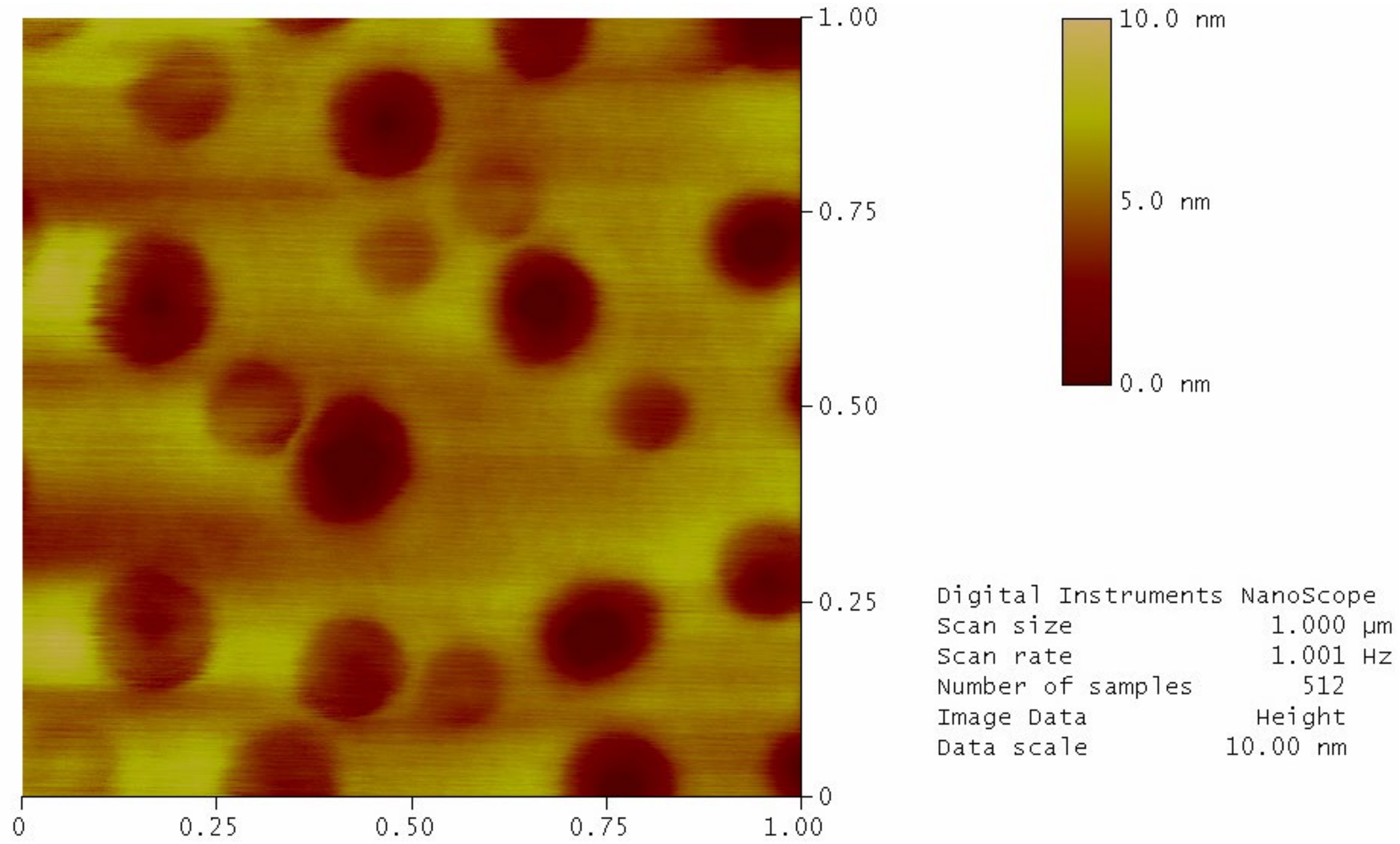

Figure S170. Representative AFM height image of **P9** showing heterogeneous surface morphology (scan area: 1 × 1 μm^2). The mean Feret diameter is 207 ± 66 nm.

C.8 **T1 analysis**

The inversion–recovery experiment monitors the recovery of the longitudinal ^{1}H magnetization following its inversion. The resulting signed signal intensities were fitted using a monoexponential recovery function:

$$I(\tau) = I_\infty + A \exp\left(-\frac{\tau}{T_{1,\text{eff}}}\right)$$

Where $I(\tau)$ is the signal intensity at inversion delay τ, I_∞ is the equilibrium signal intensity, A is the inversion-dependent amplitude, and $T_{1,\text{eff}}$ is the effective longitudinal relaxation time. This expression is the generalized form of the ideal inversion-recovery relationship, $I(\tau) = I_\infty[1 - 2\exp\left(-\frac{\tau}{T_1}\right)]$, in which the amplitude is allowed to vary to accommodate nonideal inversion and experimental scaling.

Fitting was performed using the ExpDec1 function in Origin (**Figure S171 a-c**):

$$y = I_\infty + A_1 \exp\left(-\frac{x}{t_1}\right)$$

with $t_1 = T_{1,\text{eff}}$. The reported uncertainties correspond to the standard errors of the fitted t_1 parameter. Effective relaxation times of 149 ± 1, 172 ± 1, and 262 ± 2 ms were obtained for pristine **PBD**, **P8**, and **P9**, respectively. Because the measurements represent the collective response of the detected proton environments within the polymers, the fitted values are treated as comparative effective relaxation times rather than relaxation times assigned to individual structural domains.

Direct free-induction decay (FID), solid-echo (SE), and magic-sandwich echo (MSE) measurements were additionally compared to evaluate the recovery of rapidly decaying proton signal contributions (**Figure S171 d-f**). In comparatively rigid proton environments, strong residual ^1H–^1H dipolar interactions cause rapid transverse signal decay, such that part of the signal may decay during the receiver dead time of a direct FID measurement. Solid-echo and MSE pulse sequences refocus part of this early-time dephasing and may therefore recover signal that is not fully detected by direct FID acquisition.[2]

For each sample, the FID, SE, and MSE amplitudes were divided by the maximum amplitude of the corresponding FID, assigning the normalized FID maximum a value of unity:

$$A_{k,\mathrm{norm}} = \frac{A_k}{A_{\mathrm{FID,max}}}, \qquad k = \mathrm{FID, SE, MSE}$$

Accordingly,

$$A_{\mathrm{FID,norm}} = 1$$

And the relative echo amplitudes were expressed as

$$R_{\mathrm{SE}} = \frac{A_{\mathrm{SE}}}{A_{\mathrm{FID}}}, R_{\mathrm{MSE}} = \frac{A_{\mathrm{MSE}}}{A_{\mathrm{FID}}}.$$

When the echo-recovered amplitude exceeded the direct-FID amplitude, the apparent fraction of signal not detected in the direct FID was calculated relative to the corresponding echo amplitude:

$$L_k(\%) = 100\left(\frac{A_k - A_{\mathrm{FID}}}{A_k}\right) = 100\left(1 - \frac{1}{R_k}\right), k = \mathrm{SE\ or\ MSE}.$$

Using this treatment, **P8** displayed an apparent FID signal loss of 11% relative to both SE and MSE, whereas **P9** displayed apparent losses of 18% and 23% relative to SE and MSE, respectively. Because the echo amplitudes obtained for pristine **PBD** were slightly lower than its direct-FID amplitude, no positive apparent FID signal loss was assigned. These amplitude ratios and apparent signal-loss values are used as comparative, pulse-sequence-dependent mobility descriptors. They are not interpreted as absolute rigid- or mobile-proton fractions, because the measured amplitudes may additionally depend on experimental factors such as receiver dead time, echo timing, pulse calibration, and the refocusing efficiency of the respective pulse sequences.

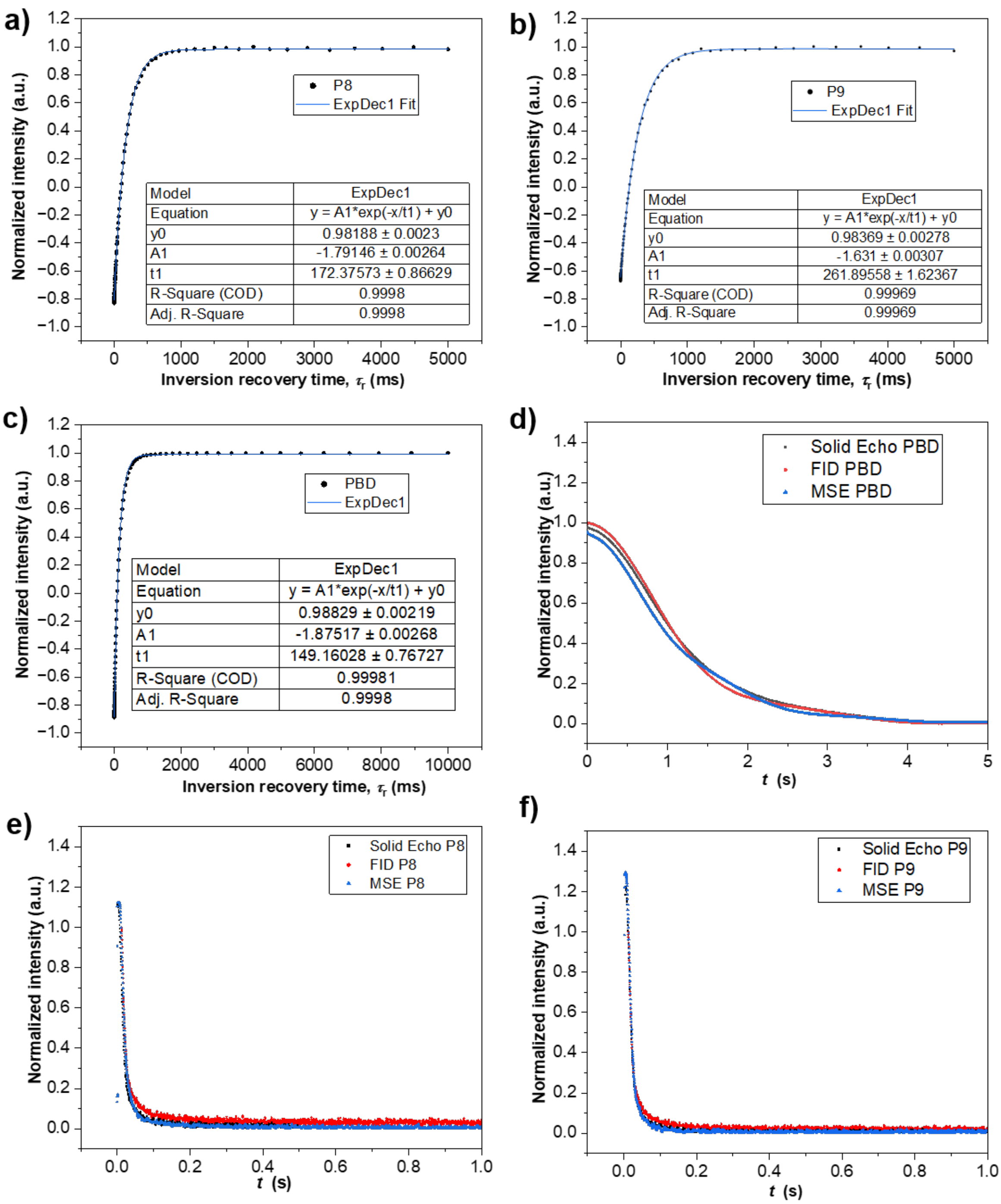

Figure S171. Low-field ^{1}H NMR relaxation analysis of pristine **PBD** and functionalized polymers **P8** and **P9**. (**a**–**c**) Normalized inversion-recovery curves of **P8**, **P9**, and pristine **PBD**, respectively. The solid lines represent the monoexponential fit, yielding effective T_1 relaxation times of 172 ± 1, 262 ± 2, and 149 ± 1 ms for **P8**, **P9**, and pristine **PBD**, respectively. (**d**–**f**) Normalized ^{1}H signal decays recorded using direct free-induction decay (FID), solid-echo, and magic-sandwich echo (MSE) pulse sequences for pristine **PBD**, **P8**, and **P9**, respectively. For each sample, all amplitudes are reported relative to the maximum FID amplitude, which was assigned a value of unity.

Table S17. Summary of the synthesis and characterization data for functionalized polymers **P1**-**P18**, including reaction composition, model-derived polymer-bound bromine content, isolated yield (%), fraction of sulfonamide- or sulfonate ester-bearing repeating units (mol%), SEC-derived number-average molar mass and dispersity (*Đ*) measured in THF, glass transition temperature (T_g), and temperature at 10% mass loss ($T_{d,10\%}$).

Entry	Polymer	Ether/sulfonamide[a]	Isolated yield (%)[b]	Average FD (mol%)[c]	Model-derived Br content (wt%)[d]	M_n[e] (THF)	*Đ*[e] (THF)	T_g[f] (° C)	$T_{d,10\%}$[g] (° C)
1	P1	THF/$MeSO_2NH_2$	74	20	48	17100	1.08	21	159
2	P2	THF/*p*-$TsNH_2$	51	30	39	18200	1.08	56	173
3	P3	THF/*p*-$NsNH_2$	85	34	35	20200	1.18	58	179
4	P4	THF/*p*-CF_3BsNH_2	18	32	35	21300	1.26	51	171
5	P5	THF/MSA	53	25	46	21400	1.93	32	163
6	P6	THF/*p*-TsOH	40	38	36	19000	1.25	43	170
7	P7	2-MeTHF/$MeSO_2NH_2$	47	11	52	15000	1.18	50	167
8	P8	2-MeTHF/*p*-$TsNH_2$	29	17	45	15100	1.19	64	178
9	P9	2-MeTHF/*p*-$NsNH_2$	37	34	34	18600	1.48	58	178
10	P10	2-MeTHF/*p*-CF_3BsNH_2	24	22	40	19400	1.22	53	181
11	P11	2-MeTHF/MSA	29	40	39	14600	1.17	45	155
12	P12	2-MeTHF/*p*TsOH	48	59	28	16000	1.12	67	162
13	P13	THP/$MeSO_2NH_2$	31	17	48	15100	1.65	53	158
14	P14	THP/*p*-$TsNH_2$	28	30	37	14300	1.36	59	174
15	P15	THP/*p*-$NsNH_2$	48	39	33	12500	1.53	50	175
16	P16	THP/*p*-CF_3BsNH_2	8	36	33	18600	1.30	40	185
17	P17	THP/MSA	42	58	33	13300	1.23	67	157
18	P18	THP/*p*-TsOH	20	68	26	15900	1.14	60	163

[a] Reactions were performed using 1.00 equiv of sulfonamide or sulfonic acid nucleophile and 67.0 equiv of cyclic ether relative to **PBD** repeating units. The multicomponent functionalization was initiated at 0 °C using an ice bath and allowed to stir for 4 h while gradually warming to ambient temperature. [b] The calculation of isolated yields is detailed in **Section A.3**. [c] Average functionalization degree estimated by internal-standard quantitative ^{1}H NMR spectroscopy as detailed in **Section A.4**. [d] Model-derived polymer-bound bromine content calculated from the average-composition model described in **Section A.4**, assuming one Br atom per original PBD repeating unit and using the qNMR-derived average FD. The reported values are compositional estimates. [e] Apparent number-average molar mass (M_n) and dispersity (*Đ*) were determined by SEC in THF using linear polystyrene standards. [f] Glass transition temperatures (T_g) were determined from the second heating cycle as the inflection point of the heat-capacity transition. [g] The temperature at 10% mass loss ($T_{d,10\%}$) was determined by TGA.

C.9 **Assessment of molecular-input sensitivity**

To provide a concise description of how the molecular input variables influence the experimentally observed material characteristics, the sensitivity of functionalization degree (FD), glass-transition temperature (T_g), decomposition temperature ($T_{d,10\%}$), molecular mobility, and morphology toward cyclic ether identity and nucleophile identity was assessed. For FD, the analysis uses the ensemble-average incorporation estimates defined in **Section A.4** and does not assume site-resolved or sequence-defined functionalization. For FD, T_g, and $T_{d,10\%}$, measurements covered the complete 18-member, 3×6 molecular design space, enabling a descriptive two-factor decomposition of the observed variation. Accordingly, the analysis describes variation across the experimentally mapped library and is not used to assign statistical significance to pairwise differences between individual polymers. Because the molecular inputs also influence the resulting FD, the individual polymers do not represent compositions in which functionalization level is held constant. The sensitivity analysis is therefore intended to describe the response landscape generated by each molecular-input combination rather than to isolate causal effects at constant FD. Likewise, the analysis does not assume identical molar-mass distributions among the individual library members and is not intended to establish pairwise statistical correlations between polymer dispersity and material properties. Molecular mobility and morphology were investigated for selected representative polymers and were therefore assessed qualitatively.

Quantitative sensitivity analysis for FD, T_g, and $T_{d,10\%}$

For a measured response y_{ij}, where *i* denotes the nucleophile (*i*=1−6) and *j* the cyclic ether (*j*=1−3), the total sum of squares, SS_{total}, was calculated from the squared deviations of the individual measurements from the grand mean, $\bar{y}$:

$$SS_{\text{total}} = \sum_{i=1}^{6}\sum_{j=1}^{3}\left(y_{ij} - \bar{y}\right)^2$$

The sum of squares associated with cyclic ether identity was calculated from the three ether-specific means, $\bar{y}_{.j}$:

$$SS_{\text{ether}} = 6\sum_{j=1}^{3}\left(\bar{y}_{.j} - \bar{y}\right)^2$$

where the factor 6 accounts for the six nucleophiles represented for each cyclic ether.

Analogously, the sum of squares associated with nucleophile identity was calculated from the six nucleophile-specific means, $\bar{y}_{i.}$:

$$SS_{\text{nucleophile}} = 3\sum_{i=1}^{6}\left(\bar{y}_{i.} - \bar{y}\right)^2$$

where the factor 3 accounts for the three cyclic ethers represented for each nucleophile.

The relative contribution of each molecular input variable to the total sum of squares was then calculated according to:

$$C_k = \frac{SS_k}{SS_{\text{total}}} \times 100$$

where *k* denotes either cyclic ether or nucleophile identity. The remaining sum of squares was calculated as:

$$SS_{\text{remaining}} = SS_{\text{total}} - SS_{\text{ether}} - SS_{\text{nucleophile}}$$

corresponding to:

$$C_{\text{remaining}} = 100 - C_{\text{ether}} - C_{\text{nucleophile}}$$

This treatment is mathematically equivalent to a descriptive sums-of-squares decomposition. Since each cyclic ether–nucleophile combination is represented by a single polymer, $SS_{\text{remaining}}$ cannot be independently partitioned into interaction and experimental residual sums of squares. It is therefore reported as remaining/non-additive variation, and the analysis is used descriptively rather than as an inferential ANOVA.

The resulting contributions are summarized in **Table S18**.

Table S18. Summary of resulting contributions.

Response	Cyclic ether contribution (%)	Nucleophile contribution (%)	Remaining/non-additive variation (%)
FD	12.50	67.63	19.87
T_g	23.86	29.05	47.09
$T_{d,10\%}$	0.49	82.63	16.88

For FD, the grand mean across the molecular design space was 33.89 mol%. The mean FD values associated with THF, 2-MeTHF, and THP were 29.83, 30.50, and 41.33 mol%, respectively. The nucleophile-specific mean values ranged from 16.00 to 55.00 mol%. Consequently, nucleophile identity accounted for 67.63% of the total observed variation in FD, whereas cyclic ether identity accounted for 12.50%, identifying nucleophile identity as the dominant systematic molecular variable for the extent of functionalization.

For T_g, the grand mean was 51.50 °C. The mean values associated with THF, 2-MeTHF, and THP were 43.50, 56.17, and 54.83 °C, respectively, while the nucleophile-specific means ranged from 41.33 to 59.67 °C. Cyclic ether and nucleophile identity accounted for 23.86% and 29.05% of the observed variation, respectively. The comparatively large remaining/non-additive contribution of 47.09% indicates that T_g cannot be described primarily by either molecular variable alone; this contribution may reflect non-additive effects of the specific molecular-input combinations and/or experimental residual variation.

For $T_{d,10\%}$, the grand mean was 169.33 °C. The mean values associated with THF, 2-MeTHF, and THP were 169.17, 170.17, and 168.67 °C, respectively, whereas the nucleophile-specific means ranged from 158.33 to 179.00 °C. Accordingly, only 0.49% of the observed variation was associated with cyclic ether identity, compared with 82.63% associated with nucleophile

identity. Within the investigated molecular design space, thermal decomposition behavior is therefore substantially more sensitive to nucleophile identity than to cyclic ether identity.

Sensitivity classification

For visualization in the molecular-input sensitivity summary, the quantitative contributions of each molecular input variable (C_k) were converted into four descriptive sensitivity classes:

$$C_k < 5\% : \mathrm{O}$$

$$5\% \leq C_k < 20\% : +$$

$$20\% \leq C_k < 40\% : ++$$

$$C_k \geq 40\% : +++$$

Here, O, +, ++, and +++ denote negligible, weak, moderate, and strong sensitivity, respectively. *These thresholds are used solely as a compact visualization of relative input sensitivity within the present molecular design space and are not interpreted as universal statistical classifications*.

Qualitative assessment of molecular mobility

Low-field ^{1}H NMR measurements were performed for selected representative polymers. The influence of nucleophile identity could be assessed under constant cyclic-ether conditions by comparing **P8** and **P9**, both synthesized using **2-MeTHF** but differing in their sulfonamide nucleophile. Changing from the *p*-tolyl-substituted sulfonamide in **P8** to the *p*-NO_2-substituted sulfonamide in **P9** increased $T_{1,\mathrm{eff}}$. from 172 ± 1 to 262 ± 2 ms, corresponding to an increase of approximately 52%. Concurrent changes were observed in the normalized solid-echo and magic-sandwich-echo responses: $A_{\mathrm{SE}}/A_{\mathrm{FID}}$ increased from 1.12 to 1.22, whereas $A_{\mathrm{MSE}}/A_{\mathrm{FID}}$ increased from 1.12 to 1.30. The apparent initial signal loss relative to the echo-detected signals likewise increased from approximately 11% for **P8** to 18–23% for **P9**. Together, these measurements reveal a pronounced change in the molecular-mobility fingerprint upon changing nucleophile identity while maintaining the same cyclic ether.

Because this comparison is based on selected representative polymers rather than the complete nucleophile series, nucleophile sensitivity was conservatively classified qualitatively as moderate (++). The effect of cyclic ether identity on molecular mobility was not determined (n.d.).

Qualitative assessment of morphological organization

Morphological organization was examined by TEM for selected polymers representing two nucleophile identities across two cyclic ethers. **P2** and **P3** were synthesized using **THF** and contain the *p*-tolyl- and *p*-NO_2-substituted sulfonamide motifs, respectively, whereas **P8** and **P9** contain the corresponding nucleophiles in the **2-MeTHF**-derived series.

Across both cyclic-ether environments, changing the nucleophile from the *p*-tolyl- to the *p*-NO_2-substituted sulfonamide produced the more pronounced change in nanoscale organization. The *p*-NO_2-containing materials **P3** and particularly **P9** displayed more evident alternating nanoscale contrast, whereas **P2** and **P8** appeared comparatively homogeneous. The persistence of this nucleophile-dependent tendency across both investigated cyclic ethers suggests that nucleophile identity exerts a strong influence on the observed morphological

organization. Cyclic ether identity modified the extent and appearance of the contrast but did not reverse the nucleophile-dependent trend.

Accordingly, the sensitivity of morphology toward nucleophile identity was classified qualitatively as strong (+++), while sensitivity toward cyclic ether identity was classified as weak (+). These assignments are based on selected representative samples and are therefore distinguished from the quantitative variance-derived classifications above.

Overall molecular-input sensitivity summary

The combined quantitative and qualitative assessment gives the following molecular-input sensitivity summary:

Molecular input	FD	Molecular Mobility	Morphology	T_g	$T_{d,10\%}$
Nucleophile identity	+++	++ †	+++ †	++	+++
Cyclic ether identity	+	n.d.	+ †	++	O

† Qualitative classification based on selected representative polymers ; n.d., not determined.

The distinct sensitivity profiles demonstrate that the different material characteristics are not governed uniformly by the molecular input variables. Nucleophile identity strongly influences FD and $T_{d,10\%}$, whereas T_g exhibits a more distributed and non-additive dependence on both molecular inputs. The selected mobility and morphology measurements further indicate that changes in nucleophile identity can translate into substantial differences in molecular dynamics and nanoscale organization. These non-equivalent response patterns support the use of a molecular-library approach rather than the prediction of the complete material response from a single chemical descriptor.

The distinct sensitivity profiles show that the measured material characteristics do not vary uniformly with the molecular input variables. Nucleophile identity is associated with most of the observed variation in FD and $T_{d,10\%}$, whereas T_g exhibits a more distributed and non-additive dependence on both molecular inputs. The selected mobility and morphology measurements further indicate substantial differences associated with nucleophile identity in molecular dynamics and nanoscale organization. These non-equivalent response patterns support a molecular-library approach rather than prediction of the complete material response from a single chemical descriptor.

C.10 **PBD Microstructure**

According to a reported procedure,[3] for microstructure analysis, 150 mg of pristine PBD was dissolved in 0.6 mL of $CDCl_3$, and Cr(acac)$_3$ (6 mg, ca. 29 mM) was added as a paramagnetic relaxation agent. ^{1}H and quantitative ^{13}C NMR spectra were recorded at 25 °C on a Varian MERCURY-300 spectrometer operating at 299.79 MHz for 1H and 75.39 MHz for ^{13}C. The ^{1}H spectrum was acquired using a 10 s relaxation delay, a 45° excitation pulse, and 128 scans. The quantitative ^{13}C spectrum was acquired using a 10 s relaxation delay, a ca. 45° excitation pulse, and 4000 scans under inverse-gated ^{1}H-decoupling conditions, with the proton

decoupler switched off during the relaxation delay and applied during acquisition to suppress NOE contributions.

The relative amounts of 1,2- and 1,4-PBD repeating units were determined from the integrated ^{1}H NMR spectrum of the pristine PBD precursor. The integral of the terminal vinyl CH_2 resonances of the 1,2-units was *A* = 5.26, while the combined integral of the olefinic CH resonances was *B* = 30.69. Since each 1,2-unit contributes two protons to *A* and one olefinic proton to *B*, whereas each 1,4-unit contributes two olefinic protons to *B*, the relative amount of 1,4-units, *X*, was calculated as:

$$X = \frac{B - A/2}{2} = \frac{30.69 - 5.26/2}{2} = 14.03$$

The relative fraction of 1,2-units was therefore:

$$f_{1,2} = \frac{A/2}{A/2 + X} \times 100 = \frac{5.26/2}{5.26/2 + 14.03} = 15.79\%$$

And consequently:

$$f_{1,4} = 100 - f_{1,2} = 84.21\%$$

The *cis*/*trans* ratio within the 1,4-PBD fraction was subsequently determined by quantitative ^{13}C NMR spectroscopy from the diagnostic methylene-carbon resonances at approximately δ= 27.4 ppm (*cis*-1,4) and δ= 32.5 ppm (*trans*-1,4). Integration gave relative contributions of 40.76% *cis*-1,4 and 59.24% *trans*-1,4 within the total 1,4 fraction. Thus:

$$f_{cis-1,4} = 84.21 \times 0.4076 = 34.33\%$$

$$f_{trans-1,4} = 84.21 \times 0.5924 = 49.89\%$$

Accordingly, the pristine PBD precursor consisted of 15.79 mol% 1,2-vinyl, 34.33 mol% *cis*-1,4, and 49.89 mol% *trans*-1,4 repeating units, corresponding to approximately 16, 34, and 50 mol%, respectively. These values describe the microstructure of the pristine PBD precursor. No stereochemical configuration is assigned to stereogenic centers generated during the subsequent multicomponent functionalization.

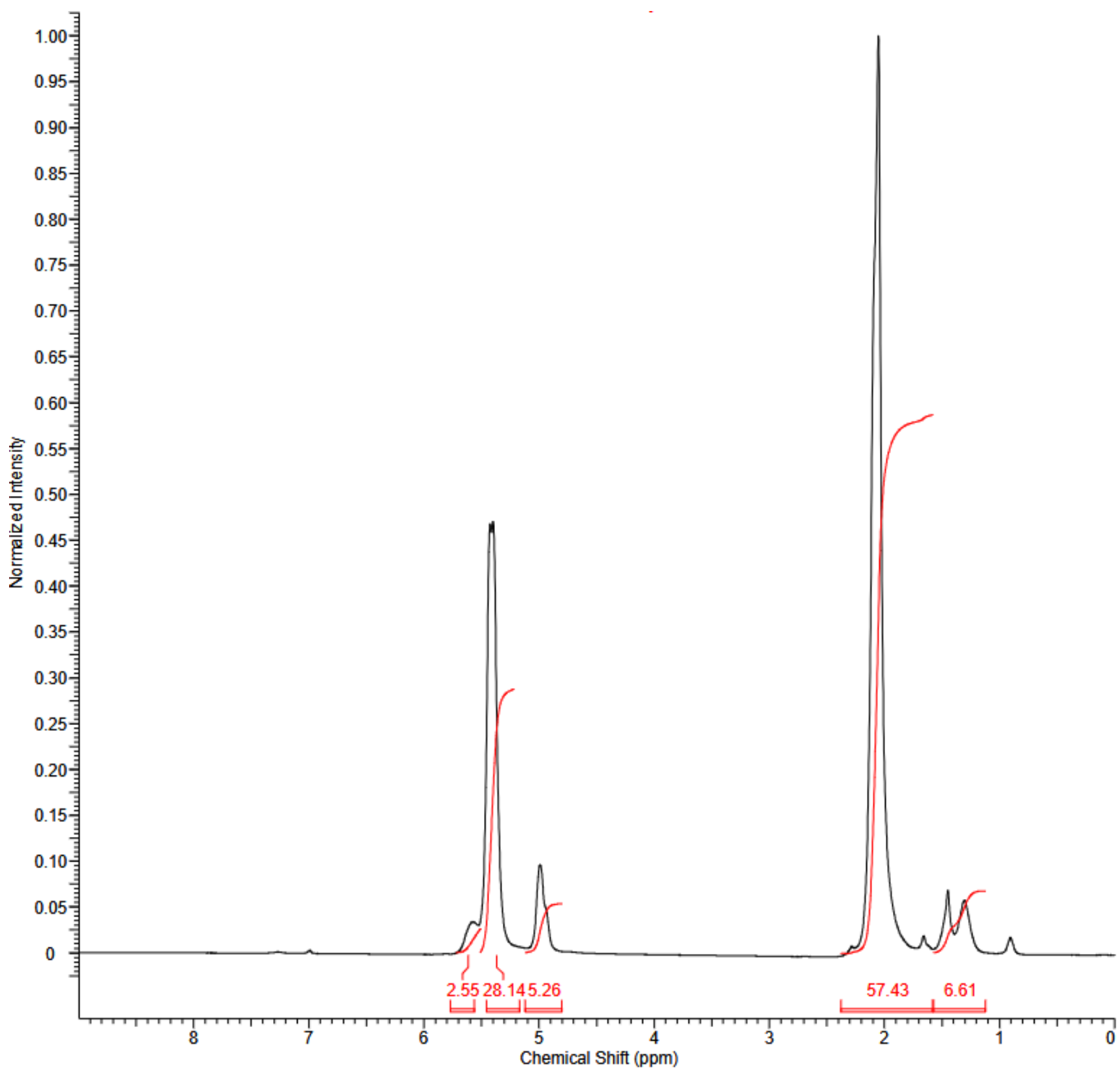

Figure S172. ^{1}H NMR spectrum of pristine PBD recorded in $CDCl_3$ at 300 MHz, showing the integration regions used to determine the relative contents of 1,2-vinyl and total 1,4 repeating units.

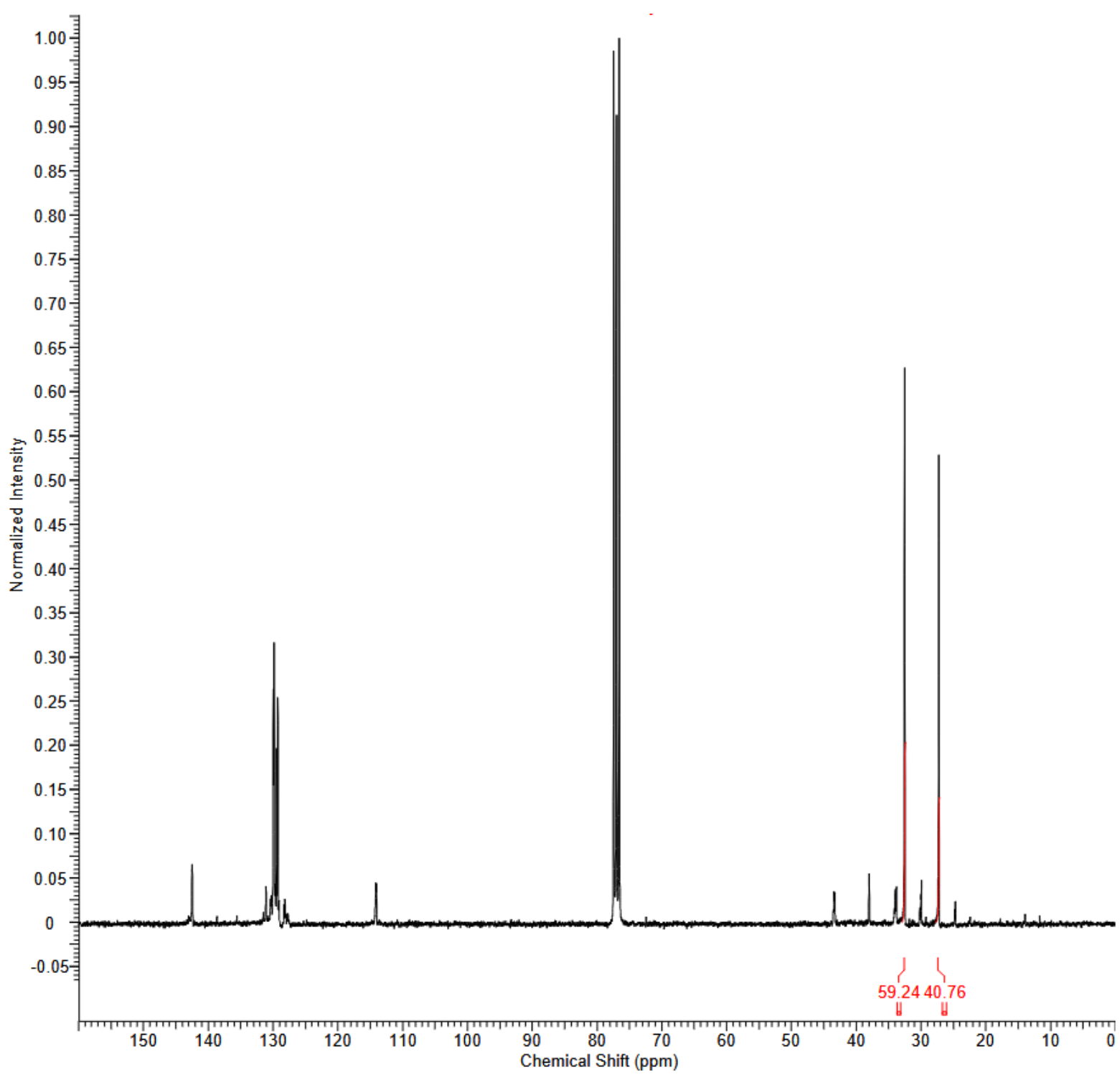

Figure S173. Quantitative ^{13}C NMR spectrum of pristine PBD recorded in $CDCl_3$ at 75 MHz under inverse-gated ^{1}H-decoupling conditions. The integrated methylene-carbon resonances used to determine the *cis*/*trans* ratio within the 1,4-PBD fraction are indicated.

D. **References**

1 J. Jeschke, J. Hobich and H. Mutlu, *Macromolecular Rapid Communications*, 2025, **46**, e00311.
2 A. Maus, C. Hertlein and K. Saalwächter, *Macromolecular Chemistry and Physics*, 2006, **207**, 1150–1158.
3 E. Thorn-Csányi and K. Ruhland, *Macromolecular Chemistry and Physics*, 1999, **200**, 1662–1671.
